# Disparities in COVID-19 mortality amongst the immunosuppressed: A systematic review and meta-analysis for enhanced disease surveillance

**DOI:** 10.1016/j.jinf.2024.01.009

**Published:** 2024-03

**Authors:** Meredith Leston, Willam Elson, Jose M. Ordóñez-Mena, Debasish Kar, Heather Whitaker, Mark Joy, Nia Roberts, F.D. Richard Hobbs, Simon de Lusignan

**Affiliations:** a Nuffield Department of Primary Care Health Sciences, University of Oxford, Oxford OX2 6GG, United Kingdom; b Immunisation and Vaccine Preventable Diseases Division, UK Health Security Agency, 61 Colindale Avenue, London NW9 5EQ, United Kingdom; c Bodleian Health Care Libraries, University of Oxford, Old Campus Road, Old Campus Research Building, Headington, Oxford OX3 7DQ, United Kingdom

**Keywords:** Immunocompromised, Disease surveillance, Digital health, Immunosuppressed, Covid, COVID-19, Vaccination, Vaccines, Personalised medicine, Pandemic preparedness

## Abstract

**Background:**

Effective disease surveillance, including that for COVID-19, is compromised without a standardised method for categorising the immunosuppressed as a clinical risk group.

**Methods:**

We conducted a systematic review and meta-analysis to evaluate whether excess COVID-associated mortality compared to the immunocompetent could meaningfully subdivide the immunosuppressed. Our study adhered to UK *Immunisation against infectious disease* (Green Book) criteria for defining and categorising immunosuppression. Using OVID (EMBASE, MEDLINE, Transplant Library, and Global Health), PubMed, and Google Scholar, we examined relevant literature between the entirety of 2020 and 2022. We selected for cohort studies that provided mortality data for immunosuppressed subgroups and immunocompetent comparators. Meta-analyses, grey literature and any original works that failed to provide comparator data or reported all-cause or paediatric outcomes were excluded.

Odds Ratios (OR) and 95% confidence intervals (CI) of COVID-19 mortality were meta-analysed by immunosuppressed category and subcategory. Subgroup analyses differentiated estimates by effect measure, country income, study setting, level of adjustment, use of matching and publication year. Study screening, extraction and bias assessment were performed blinded and independently by two researchers; conflicts were resolved with the oversight of a third researcher. PROSPERO registration number is CRD42022360755.

**Findings:**

We identified 99 unique studies, incorporating data from 1,542,097 and 56,248,181 unique immunosuppressed and immunocompetent patients with COVID-19 infection, respectively. Compared to immunocompetent people (pooled OR, 95%CI), solid organ transplants (2.12, 1.50-2.99) and malignancy (2.02, 1.69-2.42) patients had a very high risk of COVID-19 mortality. Patients with rheumatological conditions (1.28, 1.13-1.45) and HIV (1.20, 1.05-1.36) had just slightly higher risks than the immunocompetent baseline. Case type, setting income and mortality data matching and adjustment were significant modifiers of excess immunosuppressed mortality for some immunosuppressed subgroups.

**Interpretation:**

Excess COVID-associated mortality among the immunosuppressed compared to the immunocompetent was seen to vary significantly across subgroups. This novel means of subdivision has prospective benefit for targeting patient triage, shielding and vaccination policies during periods of high disease transmission.

## Highlights

• There is no universally accepted means of defining or subdividing the immunosuppressed.
• We investigated if excess COVID mortality could risk-stratify immunosuppression.
• 99 unique studies were reviewed and meta-analysed.
• Mortality was highest in transplants/cancers, lowest in HIV/inflammatory conditions.
• Case type, study setting & data matching and adjustment influenced risk gradients.

## Background

Understanding the heightened vulnerability of some individuals to COVID-19 has been of critical concern.^1^ Globally, researchers have focused on identifying patient groups most at risk of severe health outcomes and those likeliest to experience impaired seroconversion or viral clearance^2^–known contributors to vaccine evasion, pathogenic mutation and onwards resistance.^3^ Leveraging widespread community testing, these efforts delivered dramatic increases in literature that reported infection outcomes amongst the vulnerable.^4^ This has shaped decision-making around the shielding, vaccination, triage and treatment of these groups and the broader social restrictions that have safeguarded their wellbeing.^5^

The immunosuppressed are one such at-risk population.^6^ However, conducting research within the immunosuppressed still presents significant challenges. The range of conditions and treatments this group encompasses affects the generalisability of aggregated findings^7^ while small sample sizes undermine more focused research efforts - especially those targeted towards rare or complex conditions.^8^ Comparative research efforts, meanwhile, are limited by the absence of these patients from clinical development trials^9^ and inconsistencies in how immunosuppression is defined across countries and health contexts more generally.^10^ These disparities in data and definition affect researchers’ ability to establish large scale immunosuppressed cohorts and compare clinical outcomes amongst them accordingly. This is detrimental to the targeted care of these patients.

Identifying such a risk hierarchy would enhance disease surveillance while informing care and resource distribution during periods of elevated transmission. Capitalising on pandemic gains to immunosuppressed literature, the present systematic review and meta-analysis therefore explores whether excess COVID-19 mortality compared to immunocompetent outcomes can successfully subdivide the immunosuppressed spectrum.

## Methods

We conducted this systematic review and meta-analysis in line with the Cochrane Handbook for Systematic Reviews of Interventions (Version 6.3)^11^ and reported results in accordance with Preferred Reporting Items for Systematic Reviews and Meta Analysis (PRISMA) guidelines.^12^ This work has been registered with PROSPERO (registration number: CRD42022360755).^13^

### Search strategy and selection criteria

This review converted all immunosuppressed terminology included within UK *Immunisation against infectious disease* (Green Book) Chapter 14a^14^ into search terms compatible with OVID (EMBASE, MEDLINE, Transplant Library and Global Health inclusive), PubMed and Google Scholar databases. A flow chart depicting this conversion process is available in Appendix 1. These search terms were combined with COVID- and mortality-related language and entered into the databases specified (full syntax is provided in Appendix 2). OVID registries were searched concurrently via multi-file search; duplicate results were automatically removed. English language and date limits were applied (January, 2020 – August, 2022 [date of initial search]).

The use of UK criteria for immunosuppression, provided in Panel 1 below, was justified by its influence over vaccine prioritisation and the expansive yet differentiable entry point into immunosuppressed literature it provides as a condition- and medication-inclusive definition.

**Panel 1**: 'Immunosuppression for individuals aged 16 and over' as defined by Chapter 14a (COVID-19) of *UK immunisation against infectious disease (Green Book)*.

**Table:** 

As specified by our protocol,^15^ it was unclear when planning research whether a narrative review would be attempted or if the immunosuppressed literature base would be expansive enough to support meta-analysis. As such, screening occurred at two phases of stringency. Phase 1 criteria facilitated broad data extraction and identified source material that would be forwards and backwards citation tracked via Google Scholar in Phase 2. The 'relevant articles' function was applied until redundancy during this stage, however, grey literature and trial registries were excluded to prioritise the retention of original and primary data.

Once Phase 1 confirmed the feasibility of meta-analysis, Phase 2 criteria were applied to narrow inclusion scope to just the materials that could support this process. This selected for cohort designs that provided data on both immunosuppressed subgroup and immunocompetent control mortality outcomes via effect size or absolute case and mortality figures. To maximise catchment and keep the search contemporaneous, forward citation tracking was inclusive of the whole year of 2022. 2023 titles were always excluded, however, to mitigate the influence of vaccination and novel therapeutics on our analysis.

**Panel 2:** Search Phase 1 and Phase 2 inclusion and exclusion criteria.

**Table:** 

**Fig. 1:**
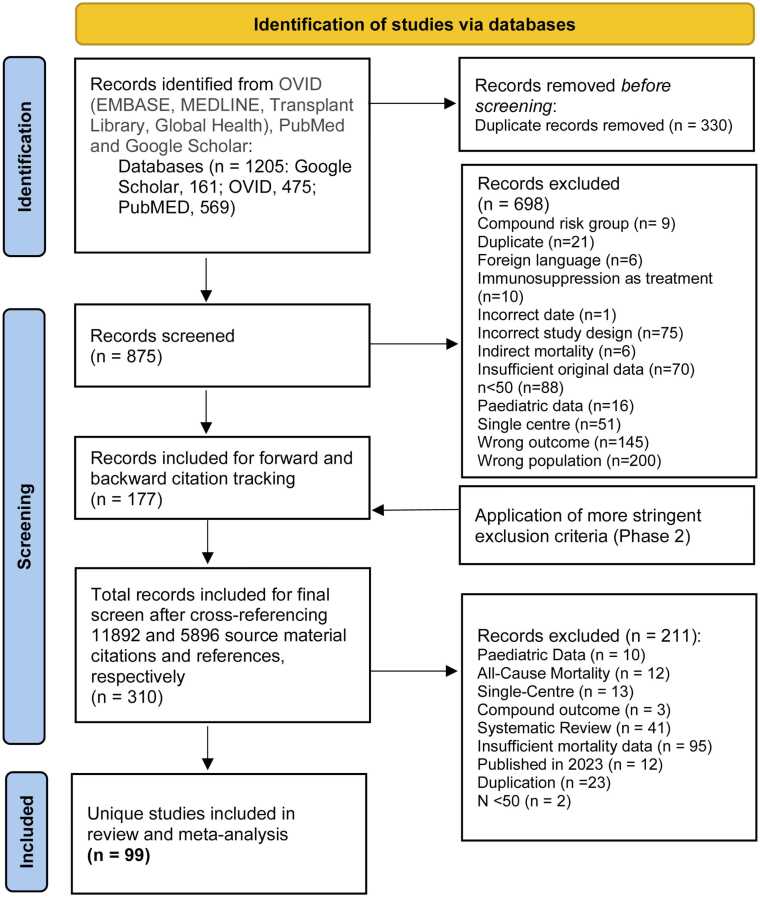
Study selection flow diagram.

These two inclusion and exclusion sets are listed in full within Panel 2; their application is illustrated in Fig. 1.

Blinded screening was performed independently by ML and WE; DK was engaged for conflict resolution. An abridged copy of the study decision-making form that supported this process is included in Appendix 3. The web application, Rayyan,^16^ provided a central portal for reviewers to review determinations and automated deduplication.

Once consensus was reached on the final study set, a configured version of the Newcastle Ottawa Scale^17^ was used to assess and compare levels of bias observed. To better fit with the aims of our investigation, 28 days was considered sufficient follow-up time for outcomes and marital status was not a necessary variable for adjustment. Both ML and WE completed these risk of bias assessments independently, the results of which are included in Appendix 4. DK was available for conflict resolution.

Studies finalised for inclusion were divided into the immunosuppressed categories and subcategories listed in Table 1 below.

**Table 1:** Immunosuppressed categories and their constituent subcategories.

Immunosuppressed categories	Immunosuppressed subcategories
Transplantation	Mixed transplant cohort *(study cohort included outcomes amongst both solid and stem cell/bone marrow transplant recipients)*
	Kidney transplant cohort
	Liver transplant cohort
	Lung/Heart transplant cohort
	
Malignancy	Mixed cancer cohort *(study cohort included outcomes amongst both solid tumour and haematological cancer patients)*
	Haematological cancers
	Solid tumours
	
Immunosuppressive agents	Mixed immunosuppressive cohort (*study cohort included outcomes amongst multiple immunosuppressive types)* Biologics Systemic steroids
	Cancer treatments *(Chemotherapy and radiotherapy)*
	
Rheumatological conditions	Generalised conditions *(general autoimmune disease, general inflammatory conditions, SLE* etc.*)* Conditions affecting the gut *(Irritable Bowel, Ulcerative Colitis, Crohn’s disease* etc.*)* Conditions affecting the joints *(Rheumatoid arthritis* etc.*)* Conditions affecting the skin *(Psoriasis* etc.*)*
	
HIV	HIV/AIDS *(any stage)*

### Data extraction

The form provided in Appendix 5 standardised and blinded the data extraction process. ML and WE independently completed the form for all included studies, alphabetising those that provided viable data points across multiple immunosuppressed groups. DK was available for conflict resolution. Extracted information for each study included title, author(s), publication year, income level (classified by World Bank as 'higher' for high-income countries and 'lower' for low, lower-middle, and upper-middle income^18^), immunosuppression type, case type (All Cases or Hospitalised), time to mortality as well as average age, sample size, number of deaths and/or comparative effect measure type, value, confidence interval (CI) and p-value for both immunosuppressed and immunocompetent cohorts. For studies based in several countries, income level was determined by the World Bank rating of the highest income country listed. Studies that only reported sample sizes and their respective mortality rates or proportions had their effect sizes imputed as odds ratios. Detail was also taken on whether data was sufficiently adjusted according to authors’ minimum criteria (adjusted for age, sex and comorbidities) and whether the values given were based on matched or unmatched cohorts.

To avoid introducing additional heterogeneity into analysis, we did not explore gradations of excess risk within a subgroup (e.g. that conferred by higher dosages, multiple malignancies, multiple transplantations etc.). To mitigate the impact of vaccination or novel therapeutics, we also preferentially extracted the earliest mortality data available from publications that reported multiple time-points. However, to enhance accuracy and capture indications of excess risk, preference was given to the most protracted time-to-mortality data available. Similarly, when choices existed, matched and maximally adjusted mortality data were prioritised over unmatched and unadjusted alternatives.

### Data analysis

R version 4.3.1^19^ was used for all data analysis via meta and metafor packages.^20^, ^21^ Statistical significance was predefined at p ≤ 0.05. To account for observed heterogeneity between studies’ sample sizes, effect measures, settings and case types, meta-analyses were performed for all immunosuppressed categories and subcategories via random effects modelling. Individual effect measures, reported or imputed from the source material, were also standardised into odds ratios for use as a common summary statistic. Although this process can produce slight overestimates of pooled effect sizes, it is justified by Symons and Moore’s^22^ case for the numerical approximation between odds, hazard and risk ratios – especially when evaluating rare diseases or outcomes via short follow-up times.

The weighted average of these odds ratios denoted the pooled intervention effect of the specific form of immunosuppression over COVID-19 mortality. We then explored whether these pooled excess mortality estimates could be presented hierarchically. I^2^ values were also reported as a measure of heterogeneity with values of 0-40%, 40-75% and 75-100% denoted low, moderate and substantial heterogeneity. Subgroup analyses interrogated the influence of effect measure, country income, COVID-19 case type, effect measure, data matching, data adjustment and publication year over these pooled excess mortality estimates - noting instances or patterns of confounding, attenuation or hierarchical flattening. There was insufficient data to attempt this at the subcategory level, however. Reporting included category-specific effect sizes, CIs and p-values.

Meta-regressions for average participant age were also run for each immunosuppressed category to establish whether excess mortality risk was static across the lifespan for these patients or if windows of excess vulnerability existed. Slope values and 95% CIs for the age effect were reported.

## Results

99 unique studies^23^, ^24^, ^25^, ^26^, ^27^, ^28^, ^29^, ^30^, ^31^, ^32^, ^33^, ^34^, ^35^, ^36^, ^37^, ^38^, ^39^, ^40^, ^41^, ^42^, ^43^, ^44^, ^45^, ^46^, ^47^, ^48^, ^49^, ^50^, ^51^, ^52^, ^53^, ^54^, ^55^, ^56^, ^57^, ^58^, ^59^, ^60^, ^61^, ^62^, ^63^, ^64^, ^65^, ^66^, ^67^, ^68^, ^69^, ^70^, ^71^, ^72^, ^73^, ^74^, ^75^, ^76^, ^77^, ^78^, ^79^, ^80^, ^81^, ^82^, ^83^, ^84^, ^85^, ^86^, ^87^, ^88^, ^89^, ^90^, ^91^, ^92^, ^93^, ^94^, ^95^, ^96^, ^97^, ^98^, ^99^, ^100^, ^101^, ^102^, ^103^, ^104^, ^105^, ^106^, ^107^, ^108^, ^109^, ^110^, ^111^, ^112^, ^113^, ^114^, ^115^, ^116^, ^117^, ^118^, ^119^, ^120^, ^121^, ^122^ were carried forward for inclusion in meta-analysis, 18 of which provided mortality data for multiple immunosuppressed subgroups. Overall, data from 1,542,097 immunosuppressed patients and 56,248,181 immunocompetent comparators were analysed.

Table 2 provides a summary of the characteristics of included studies, including the results of Newcastle Ottawa Scale-based bias ratings (AHRQ Standard Fairness Assessment). Further details can be found in Appendix 6. Here, studies that reported both aggregated and subdivided data are flagged to prevent multiple-counting of sample sizes. Likewise, Appendix 6 demarcates when effect size estimates were not directly reported, but were imputed using the available sample sizes and mortality data.

**Table 2:** Summary table of unique studies included in comparative meta-analysis.

Study variable		Number of studies	Percentage
Immunosuppressed category	Transplantation	13	13.13
	Malignancy	26	26.26
	Immunosuppressive agents	4	4.04
	Rheumatological conditions	27	27.27
	HIV	26	26.26
	Multiple	3	3.03
		
AHRQ Standard Fairness Assessment	Good	86	86.86
	Fair	0	0.00
	Poor	13	13.13
		
Year	2020	21	21.21
	2021	29	29.29
	2022	49	49.49
		
Cohort type	Prospective	10	10.1
	Retrospective	89	89.89
		
Income level	Higher	90	90.91
	Lower	9	90.09
		
Case type	All cases	48	48.48
	Hospitalised	51	51.52
		
Matching	Matched	61	61.62
	Unmatched	38	38.38
		
Adjustment	Adjusted	39	39.39
	Unadjusted	60	60.61

Despite being constructed as odds ratios, all data reported in this work uses effect size language. This is in recognition of the variety of effect measures that were extracted and combined from the literature and the significant differences in values that were occasionally detected between them in their unharmonised forms (as listed in Appendix 7).

### Subdividing excess risk for COVID-19 associated mortality amongst immunosuppressed categories and subcategories

**Fig. 2:**
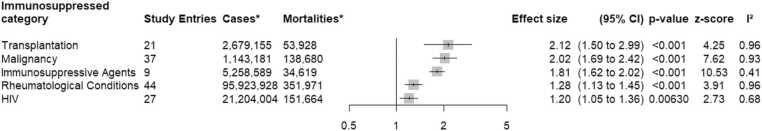
Excess COVID-19 associated mortality by immunosuppressed category. *The column labelled as Cases indicates the total number of participants, immunosuppressed and immunocompetent combined. The column labelled as Mortalities indicates the total number of deaths registered in participants, immunosuppressed and immunocompetent combined. Instances of multiple-counts were not removed.

Fig. 2 depicts the results of meta-analyses run at the immunosuppressed category level as a summary forest plot; they are listed in descending order of pooled effect size and are elaborated in Appendix 8. Our findings suggested that compared to the immunocompetent, transplantation recipients, malignancy patients and those using immunosuppressive agents had greater COVID-19 mortality risk than those with rheumatological conditions or HIV. Except for HIV and Immunosuppressive Agents, within-category heterogeneity was substantial.

**Fig. 3:**
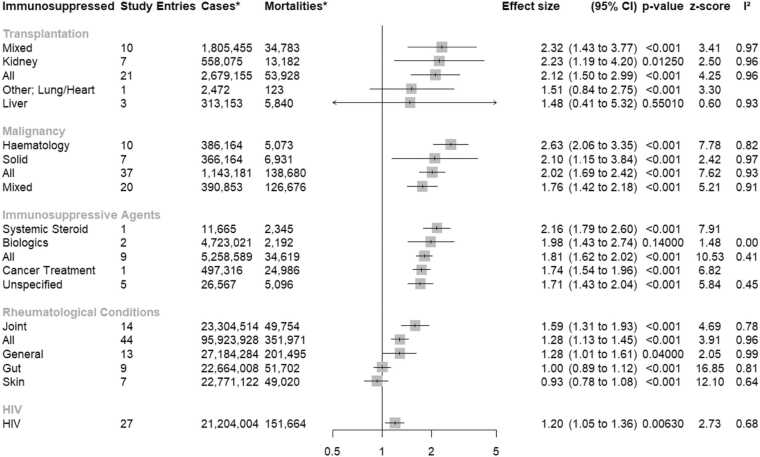
Excess COVID-19 associated mortality by immunosuppressed subcategory. *The column labelled as Cases indicates the total number of participants, immunosuppressed and immunocompetent combined. The column labelled as Mortalities indicates the total number of deaths registered in participants, immunosuppressed and immunocompetent combined. Instances of multiple-counts were not removed.

**Fig. 4:**
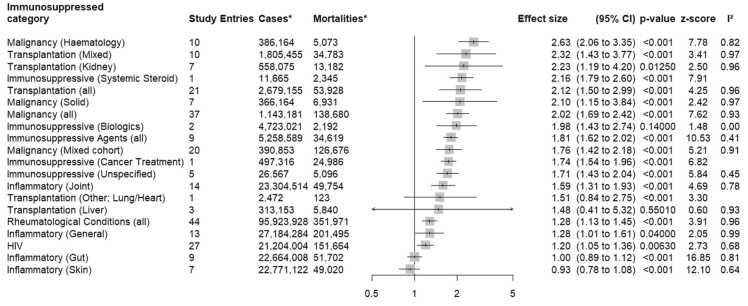
Excess COVID-19 associated mortality by immunosuppressed category and subcategory presented in descending order of effect size. *The column labelled as Cases indicates the total number of participants, immunosuppressed and immunocompetent combined. The column labelled as Mortalities indicates the total number of deaths registered in participants, immunosuppressed and immunocompetent combined. Instances of multiple-counts were not removed.

Results for immunosuppressed subcategories are summarised in Fig. 3, Fig. 4; HIV was not subcategorised, as stage and CD4 count data was not provided consistently across studies. These visuals demonstrate the influence of certain higher-risk subcategories over excess mortality estimates at the categorical level. This was especially pronounced amongst malignancy data where risk estimates were disproportionately high amongst haematological subcategories. Inflammatory joint conditions also returned higher excess mortality estimates than their generalised or gut- or skin-specific counterparts. However, there are notable instances where pooled effect size estimates were underpowered; single and small sample subcategories such as systemic steroids, biologics, cancer treatment, liver and rarer transplant types (lung/heart) may not have reflected the true clinical experience of these patient groups.

### Attenuation of excess COVID-19 associated mortality reported between immunosuppressed categories

Table 3 and Appendix 8 illustrate the impact of country income, case type, level of adjustment, use of matching and publication year on excess COVID-19 mortality in each immunosuppressed category.

**Table 3:** Results of subgroup analyses by country income, case type, level of confounder adjustment, use of matching and publication year.

Immunosuppression	Income	Number of study entries	Effect size	(95% CI)	I^2^	p-value	
*Subgroup analyses by country income*							
Transplantation	Higher	20	2.13	(1.48-3.04)	96.3%	0.82	
	Lower	1	1.90	(0.76-4.73)	-		
Malignancy	Higher	30	1.82	(1.49-2.21)	87.6%	0.007	
	Lower	7	2.96	(2.17-4.05)	97.2%		
Immunosuppressive agents	Higher	9	1.81	(1.62-2.02)	41.2%	NA	
	Lower	0	-	-	-		
Rheumatological conditions	Higher	42	1.27	(1.11-1.44)	96.3%	<0.001	
	Lower	2	1.74	(1.57-1.93)	0%		
HIV	Higher	24	1.13	(1.00-1.28)	57.0%	0.001	
	Lower	3	1.78	(1.39-2.28)	43.6%		

*Subgroup analysis by COVID-19 case type*							
Transplantation	All cases	10	3.36	(1.95-5.78)	95.6%	0.003	
	Hospitalised cases	11	1.37	(1.05-1.77)	79.6%		
Malignancy	All cases	15	2.21	(1.49-2.97)	91.1%	0.76	
	Hospitalised cases	22	1.98	(1.61-2.43)	93.6%		
Immunosuppressive agents	All cases	3	1.63	(1.22-2.17)	46.0%	0.38	
	Hospitalised cases	6	1.88	(1.63-2.18)	47.8%		
Rheumatological conditions	All cases	35	1.31	(1.14-1.51)	96.9%	0.50	
	Hospitalised cases	9	1.18	(0.9-1.55)	87.9%		
HIV	All cases	10	1.32	(1.05-1.65)	80.1%	0.22	
	Hospitalised cases	17	1.11	(0.96-1.29)	43.7%		
			
*Subgroup analysis by level of confounder adjustment*							
Transplantation	Yes	15	1.68	(1.25-2.25)	95.7%	0.0758	
	No	6	3.72	(1.63-8.5)	95.2%		
Malignancy	Yes	19	1.95	(1.49-2.55)	94.2%	0.6992	
	No	18	2.1	(1.7-2.7)	90.1%		
Immunosuppressive agents	Yes	9	1.81	(1.62-2.02)	41.2%	NA	
	No	0	-	-	-		
Rheumatological conditions	Yes	33	1.23	(1.09-1.39)	84.7%	0.4	
	No	11	1.43	(1.03-1.99)	94.5%		
HIV	Yes	14	1.35	(1.12-1.62)	73.4%	0.05	
	No	13	1.06	(0.92-1.22)	53.9%		
	
*Subgroup analysis by matching of immunocompetent and immunocompromised*							
Transplantation	Matched	12	1.36	(1.04-1.79)	72.2%		0.0017
	Unmatched	9	3.55	(2.09-6.04)	95.9%		
Malignancy	Matched	9	1.69	(1.34-2.13)	70.7%		0.1664
	Unmatched	28	2.13	(1.7-2.66)	93.8%		
Immunosuppressive agents	Matched	2	0.97	(0.52-1.84)	0.0%		0.05
	Unmatched	7	1.85	(1.65-2.06)	38.7%		
Rheumatological conditions	Matched	18	1.18	(0.99-1.41)	74.3%		0.28
	Unmatched	26	1.35	(1.14-1.6)	97.6%		
HIV	Matched	7	1.06	(0.79-1.42)	48.3%		0.32
	Unmatched	20	1.24	(1.09-1.43)	66.3%		
	
*Subgroup analysis by publication year*							
Transplantation	2020	6	1.44	(0.84-2.50)	89.5%		0.31
	2021	6	2.27	(1.60-3.21)	92.5%		
	2022	9	2.58	(1.31-5.05)	97.4%		
Malignancy	2020	15	1.68	(1.36-2.07)	82.9%		0.03
	2021	15	1.97	(1.45-2.67)	91.8%		
	2022	7	3.05	(2.02-4.61)	91.9%		
Immunosuppressive agents	2020	0	-	-	-		
	2021	8	1.82	(1.62-2.03)	48.2%		0.74
	2022	1	1.44	(0.38-5.52)	-		
Rheumatological conditions	2020	0	-	-	-		-
	2021	0	-	-	-		
	2022	44	1.28	(1.13-1.45)	0%		
HIV	2020	4	1.12	(0.83-1.51)	0%		
	2021	13	1.24	(1.04-1.48)	76.0%		0.81
	2022	10	1.14	(0.86-1.51)	61.5%		

For Rheumatological Conditions, HIV and Malignancy, reduced effect sizes are seen in studies conducted in higher income countries than those conducted in lower income; in these categories, subgroup differences between higher and lower income pooled estimates reached statistical significance. The comparatively lower heterogeneity seen within HIV studies suggests the influence of income might have been more stable in this category. Finally, while this trend’s apparent reversal amongst transplant patients warrants recognition, it is important to note it was based on single-study reporting only.

Although there is evidence that pooled estimates for excess immunosuppressed COVID-19 mortality were greater in cohorts that did not restrict data collection to hospitalised cases (an instance of Berkson’s Bias^123^), this pattern did not reach statistical significance. Indeed, the influence of case type only reached statistical significance amongst transplant recipients with the reverse observation, though on the basis of a single study estimate.

Discrepancies between immunosuppressed and immunocompetent outcomes were seen to stabilise once mortality data was adjusted for minimum criteria of age, gender and comorbidities or if studies compared matched cohorts. Inflated estimates for excess COVID-19 mortality were given when data was collected in its raw form or comparisons were made between unmatched cohorts. However, these observations for matching-based and adjustment-based attenuation did not meet statistical significance in all circumstances, only for HIV- (reversed trend) and Transplantation- and Immunosuppressive Agent-specific studies respectively.

Subgroup analysis on the basis of publication year only reached significance within malignancy data where there was a clear increase in pooled estimates over time. However, publication year and years of study activity do not correspond in this work; data published in 2022 may assess 2020 COVID-infection outcomes for example. As such it was not possible to interpret this data clinically.

### Modelling for the influence of age over excess COVID-19 associated mortality between immunosuppressed categories

Results of meta-regression on average age of participants are provided in Appendix 9. The results suggested an inverse relationship between increasing age and COVID-19 excess mortality among the immunosuppressed, although none of the analyses were statistically significant.

## Discussion

### Summary of findings

This systematic review and meta-analysis demonstrates that excess COVID-19 mortality can be used to subdivide the immunosuppressed spectrum. The exact nature of this subdivision, and the conditions or medications that might constitute its levels or means of differentiation, still require formal consensus building. However, the application of this evidence to disease surveillance and pharmacological research and development may improve the granularity of insights that can emerge from both.

For example, it is clear that transplant and malignancy patients, especially those with haematological cancers, face heightened vulnerability. Conversely, HIV, rheumatology patients and those under cancer treatment exhibit mortality risks closer to the immunocompetent baseline. These observations add weight to moderate versus severe characterisations of immunosuppression^124^ as opposed to unstructured or list-based alternatives. They also hold clinical significance for COVID-19 triage tools and policies that direct the distribution of therapeutics, booster vaccinations and shielding notices amongst this diverse risk group.

This work also suggests the sensitivity of targeted surveillance to variables such as setting income, case type and data matching and adjustment. Its global vantage produces evidence that less-resourced settings were at a comparative disadvantage for protecting vulnerable populations, particularly patients with malignancies or HIV. The pandemic’s well-established exacerbation of inequalities in medical access, drug-management of pre-existing conditions and eventual vaccine distribution should be considered when interpreting such findings.^125^ Meanwhile, disparities in accessing transplantation, rather than increased clinical risk, might have contributed to the significant excess mortality seen among transplant recipients in higher-income countries – even if only based on a single-study estimate. This procedure is a comparable luxury in lower-income settings and may select for more affluent patients, engendering better outcomes than would otherwise be expected.^126^

Although failing to reach statistical significance, data reported amongst all COVID-19 case types revealed heightened and more differential vulnerability in the immunosuppressed; restricting analysis to hospitalised patients, meanwhile, diminished pooled effect scores and flattened the excess mortality gradient. This is suggestive of an 'equalising' role for inpatient care in COVID-19 mortality. Once sick enough to be admitted, being immunosuppressed in any capacity may add little to the onward likelihood of succumbing to the disease. This instance of Berkson’s Bias^123^ is exacerbated by the differential clinical thresholds by which immunosuppressed versus immunocompetent patients are admitted - triage for the immunosuppressed is accelerated as a precautionary tactic.^127^

Likewise, although statistical significance was not reached universally, this research also highlights the potentially inflated nature of excess COVID-19 mortality estimates that fail to either match immunosuppressed with immunocompetent cohorts or adjust their results for key criteria including age, sex and comorbidities. This was especially pronounced in literature published early on in the pandemic. Here the clinical priority was to establish raw mortality risks for immediate action; experimental designs that delayed publication to match cohorts or fully control for confounders were more commonplace in later years.^128^

Finally, tentative evidence is provided for the influence of age on excess COVID-19 mortality among immunosuppressed categories: this consistently negative relationship is intuitive given the extreme rarity of COVID-19 associated death in younger immunocompetent demographics and aligns with a recent systematic review of infection outcomes in younger demographics.^129^

### Comparison with existing literature

Encouragingly, the results of this work are in broad agreement with preliminary reports of differential hospitalisation, ICU admission and mortality rates made by the INvestigation oF cOvid-19 Risk amongst iMmunocompromised populations (INFORM) study.^130^ Compared to other immunosuppressed groups assessed, here too transplant and haematological malignancy patients were seen to be at heightened risk for critical outcomes. Our findings are also aligned with the narrative efforts of SeyedAlinaghi^131^ and added nuance to aggregated work by Baek et al.^132^ and comprehensiveness to the part-differentiated work of Belsky and colleagues^10^ and the International Severe Acute Respiratory and emerging Infections Consortium (ISARIC) WHO Clinical Characterisation Protocol UK (CCP-UK).^133^

This work could not have been completed without the influx of immunosuppressed literature the pandemic espoused. Indeed, comparable work by Kunisaki and Janoff^134^ was only capable of summarising immunosuppressed outcomes for influenza amongst select subgroups, non-comparatively and at a narrative level.

### Strengths and limitations

To the best of our knowledge, this work is the first of its kind to comprehensively and comparatively appraise studies relating to COVID-19 mortality in immunosuppressed populations. Due to forwards and backwards citation tracking, this review cross-referenced a large amount of relevant literature and presents an authoritative perspective on this topic.

The global scope, nearly 3-year coverage and interrogation into income, case type and other possibly confounding variables are unique to this systematic review. The consistency of immunosuppressed risk gradients, even when attenuated by the influence of income, case type, data adjustment, data matching, or publication year, validated our decision to meta-analyse these dissimilar study sets for the benefit of generating clinical insight.

However, the work presented here can still be improved upon in a number of ways. For example, over the course of the search period our population variable, COVID-19 infection, proved inconsistent. As already specified, infection with COVID-19 in early 2020 was tangibly different to infection early the following year.^135^ Despite best efforts to select for earliest available literature, there was real difficulty controlling for the impacts of viral mutation and the introduction of novel therapeutics and vaccines when appraising excess mortality across the pandemic timeline. Due to discrepancies between publication date and study time periods, subgroup analysis by publication date proved ineffective at gauging how dynamic excess COVID-19 mortality was for the immunosuppressed and if attenuation over time was consistent between subgroups.

Meanwhile, at the exposure level, our inclusion criteria were not compatible with the literature available on treatment-specific forms of immunosuppression. Studies were ordinarily structured to compare medicated immunosuppressed mortality with unmedicated counterparts. Despite being highly informative, the absence of immunocompetent controls precluded these papers from being incorporated into our analysis. Separate systematic review would be beneficial for differentiating the excess mortality risk conferred by various immunosuppressive medications or treatment regimens; subgroup analyses could interrogate the influence of drug dosage and timing. Furthermore, our requirement for sample sizes greater than 50 proved overly ambitious for obtaining mortality data on rarer forms of immunosuppression such as genetically-conferred conditions, stem cell or bone marrow transplant recipients and rarer malignancies (brain tumours etc.). Given past documentation of their extreme vulnerability to COVID-136, 137 our work suffered from their absence. Any go-forward hierarchy of COVID-19 associated risk amongst the immunosuppressed must still incorporate these patients’ experiences. There was also some confusion amongst authors as to whether to include immunosuppressed groups that were in the spirit of UK immunosuppressed definitions, but were not explicitly stated. This included patients with multiple sclerosis, cystic fibrosis and those on haemodialysis – all of which appear in alternative characterisations of the immunosuppressed.^138^ For comprehensiveness, future replications of this work may wish to incorporate these groups into their search terms. Finally, it is possible that paediatric data was still included in studies that did not make their age ranges explicit.

Specifying and extracting comparison data also proved difficult during this investigation. Close inspection of included studies’ immunocompetent controls or comparator groups amongst the general population often revealed instances of diabetes, obesity and other secondarily immunosuppressive conditions – especially when experimental and control cohorts were age, sex or propensity score matched. This may detract from the validity of present reports for excess mortality, possibly proving underestimates of true clinical effect.

The outcome measure utilised by this review, excess COVID-19 associated mortality, was also not without limitation. As indicated, the diversity of effect measures extracted may have inflated summary values once collapsed. Furthermore, the uniquely therapeutic effect of immunosuppressants over critical COVID-19 outcomes^139^ may make data gathered on excess immunosuppressed mortality in this use case, and gradient therein, poorly generalisable to other infections. This warrants investigation. The exclusion of studies that utilised all-cause mortality may also be questioned. However, this was justified by the potentially confounding influence of indirect COVID-19 mortality on our results, something especially pronounced in the immunosuppressed who were disproportionately affected by treatment disruptions, late-stage diagnoses and mortality attributable to pre-existing conditions.^140^, ^141^ By eliminating all-cause mortality data, authors were attempting to control for these factors.

## Conclusion

This large systematic review and meta-analysis has generated insights that are informative for disease surveillance amongst the immunosuppressed. The risk gradient observed may prove beneficial for prioritising scarce medical resources including booster vaccine doses, novel therapeutics and passive forms of immunisation such as convalescent plasma and monoclonal antibody transfusion.

This work is the first of its kind to appraise and compare immunosuppressed literature comprehensively and we invite researchers to test the stability of its findings across international contexts and disease use cases. Our priority is to now translate these findings into a definitive risk hierarchy or binary via clinical consensus building before validating this endpoint in real-world datasets. Doing so will confirm whether the literature-predicted COVID-19 outcomes presented here reflect the lived reality of these vulnerable patients.

## Role of funding source

Funders had no role in study design, data collection, data analysis, data interpretation, or the writing of this report.

## Funding

Supported by EMIS Health and the UK Medical Research Council (Grant number: MR/R015708/1).

## Declaration of Competing Interest

The authors of this manuscript are all either directly employed by or affiliated with the Royal College of General Practitioners’ Research and Surveillance Centre (Oxford-RCGP RSC), the UK primary care sentinel network. This work originated as a direct response to the limitations of existing disease surveillance, both within the RSC and comparable networks, to monitor outcomes within the immunosuppressed with high resolution and fidelity. The findings of this work will now be validated within RSC data flows and amongst RSC collaborator groups. However, none of these stated actors or collaborators had any influence on the approach, analysis, interpretation or write-up of this work.
