# Supplementary figures and images for: Disparities in COVID-19 mortality amongst the immunosuppressed: A systematic review and meta-analysis for enhanced disease surveillance
Source: J Infect. 2024 Mar;88(3):None. doi: 10.1016/j.jinf.2024.01.009 (PMC10943183; doi:10.1016/j.jinf.2024.01.009)

**
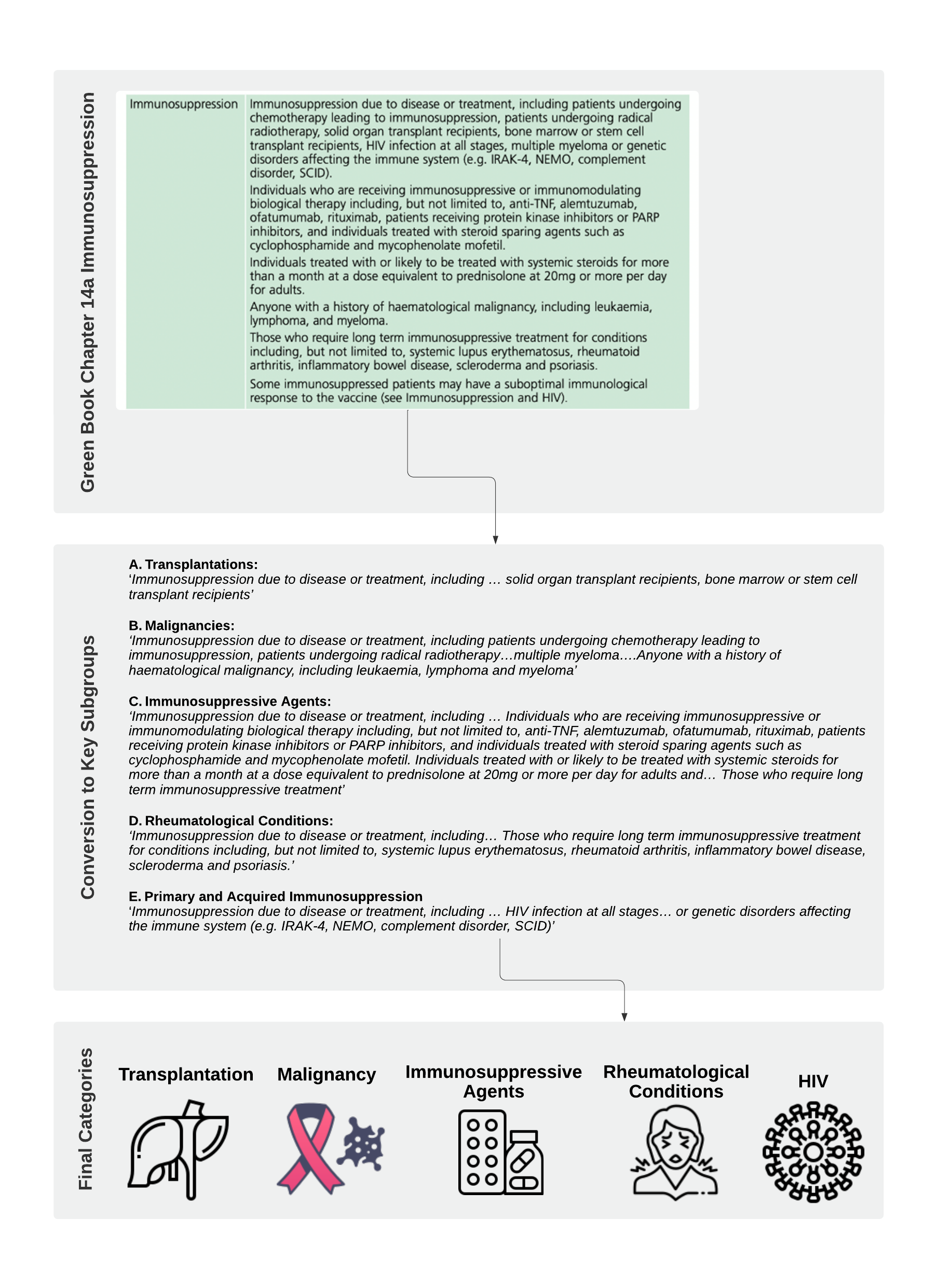
 Appendix 1: Green Book Chapter 14a Immunosuppression Terms**

Supplement: Supplementary file 1 — Supplementary material [file mmc1.docx]
