# Supplementary material for: Disparities in COVID-19 mortality amongst the immunosuppressed: A systematic review and meta-analysis for enhanced disease surveillance
Source: J Infect. 2024 Mar;88(3):None. doi: 10.1016/j.jinf.2024.01.009 (PMC10943183; doi:10.1016/j.jinf.2024.01.009)
Supplement: Supplementary file 2 — Supplementary material [file mmc2.docx]

**Appendix 2: Search terminology**

1. Search terms applied within OVID database, combining Green Book immunosuppression terms with established syntax for COVID-19 and mortality
2. (immunodef* or immunosup* or immunocomprom* or autoimm* or chemo* or radiother* or "biological therap*" or immunomod* or steroid or prednisolone or HIV or transplant* or myeloma or PID or "primary immunodeficiency disorder" or "human immunodeficiency virus" or AIDS or "acquired immunodeficiency syndrome" or "haematological malignancy" or leukaemia or lymphoma or "rheumatoid arthritis" or IBS or "inflammatory bowel disease" or scleroderma or psoriasis or lupus).ti
3. (COVID-19 or "Sars-CoV-2" or COVID*).ti
4. (death or "excess death" or mortality or "excess mortality"). ti

1 AND 2 AND 3

1. Search terms applied within PubMed database, combining Green Book immunosuppression terms with established syntax for COVID-19 and mortality

chemo*[Title] OR radiother*[Title] OR "solid organ transplant*"[Title] OR "bone marrow transplant*"[Title] OR "stem cell transplant*"[Title] OR myeloma[Title] OR complement[Title] “biological therap*”[Title] OR immunothera* [Title] OR immunomod*[Title] OR "primary immunodeficiency disorder"[Title] OR PID[Title] OR HIV[Title] OR “human immunodeficiency virus”[Title] OR AIDS[Title] OR "Acquired immunodeficiency syndrome"[Title] OR steroid*[Title] OR prednisolone[Title] OR "haematological malignancy"[Title] OR leukaemia[Title] OR lymphoma[Title] OR "rheumatoid arthritis"[Title] OR IBS[Title] OR "inflammatory bowel disease"[Title] OR scleroderma[Title] OR psoriasis[Title] OR SLE[Title] OR lupus[Title]

AND

COVID*[Title] OR “COVID-19”[Title] OR “Sars-CoV-2”[Title]

AND

death[Title/Abstract] OR mortality[Title/Abstract] OR “excess death”[Title/Abstract] OR “excess mortality”[Title/Abstract]

1. Search terms applied within Google Scholar database, combining Green Book immunosuppression terms with established syntax for COVID-19 and mortality

COVID-19 immunocompromised "excess mortality" AND chemotherapy OR radiotherapy OR solid OR organ OR transplant OR myeloma OR complement OR biologics OR immunotherapy OR PID OR HIV OR AIDS OR steroid OR prednisolone OR malignancy OR lymphoma OR arthritis OR IBS OR scleroderma OR lupus OR psoriasis
