# Supplementary material for: Disparities in COVID-19 mortality amongst the immunosuppressed: A systematic review and meta-analysis for enhanced disease surveillance
Source: J Infect. 2024 Mar;88(3):None. doi: 10.1016/j.jinf.2024.01.009 (PMC10943183; doi:10.1016/j.jinf.2024.01.009)
Supplement: Supplementary file 3 — Supplementary material [file mmc3.docx]

**Appendix 3: Abridged Study Screening Form, Phase 1 and 2 Compatible**

| **Title** | **Year** | **Journal** | **Issue #** | **Pages** | **Authors** | **URL** | **Abstract** | **DOI** | **Keywords** | **Chapter 14a Subgroup** | **Case study** | **n<50** | **Immunotherapy as candidate COVID treatment** | **Single-centre** | **Compounded risk/immunosuppression/ outcome** | **Paediatric** | **No exposed and unexposed mortality data provided** | **Population statistic** | **All-cause mortality** | **2023 data** | **Meta-analysis** | **Decision and justification** | **Notes** |
| --- | --- | --- | --- | --- | --- | --- | --- | --- | --- | --- | --- | --- | --- | --- | --- | --- | --- | --- | --- | --- | --- | --- | --- |
| Tumor necrosis factor inhibitors are associated with a decreased risk of COVID-19-associated hospitalization in patients with psoriasis-A population-based cohort study | 2021 | Dermatol Ther | 4 | e15003 | Kridin et al | |  |  |  |  |  |  |  |  |  |  |  |  |  |  |  |  |  |
| Characteristics, outcomes, and mortality amongst 133,589 patients with prevalent autoimmune diseases diagnosed with, and 48,418 hospitalised for COVID-19: a multinational distributed network cohort analysis | 2020 | MedRxiv : the Preprint Server for Health Sciences | | 27 | Tan et al |  |  |  |  |  |  |  |  |  |  |  |  |  |  |  |  |  |  |
| Prevalence and Mortality due to COVID-19 in HIV Co-Infected Population: A Systematic Review and Meta-Analysis | 2021 | Infect Dis Ther | 3 | 1267-1285 | Liang et al | |  |  |  |  |  |  |  |  |  |  |  |  |  |  |  |  |  |
| COVID-19 mortality in patients with cancer on chemotherapy or other anticancer treatments: A prospective cohort study | 2020 | The Lancet | | 1919-1926 | Lee et al |  |  |  |  |  |  |  |  |  |  |  |  |  |  |  |  |  |  |
