# Supplementary material for: Disparities in COVID-19 mortality amongst the immunosuppressed: A systematic review and meta-analysis for enhanced disease surveillance
Source: J Infect. 2024 Mar;88(3):None. doi: 10.1016/j.jinf.2024.01.009 (PMC10943183; doi:10.1016/j.jinf.2024.01.009)
Supplement: Supplementary file 4 — Supplementary material [file mmc4.docx]

**Appendix 4: Copy of completed Newcastle Ottawa Bias Assessment Form**

| **Author** | **Title** | **Representativeness of exposed cohort** | **Source of non-exposed cohort** | **Ascertainment of exposure** | **Demonstration that outcome was not present at start of study** | **Comparability of cohorts** | **Assessment of outcome** | **Sufficient follow-up time** | **Completeness of follow-up** | **AHRQ standard fairness assessment** |
| --- | --- | --- | --- | --- | --- | --- | --- | --- | --- | --- |
| Alpert et al | Clinical course of cancer patients with COVID-19: a retrospective cohort study | 1 | 1 | 1 | 1 | 2 | 1 | 1 | 1 | Good quality |
| Attauabi et al | Outcomes and long-term effects of COVID-19 in patients with inflammatory bowel diseases—A Danish prospective population-based cohort study with individual-level data | 1 | 1 | 1 | 1 | 2 | 1 | 1 | 1 | Good quality |
| Belleudi et al | Direct and indirect impact of COVID-19 for patients with immune-mediated inflammatory diseases: a retrospective cohort study | 1 | / | 1 | 1 | 2 | 1 | 1 | 1 | Good quality |
| Bennett et al | Multicenter Study of Outcomes Among Persons With HIV Who Presented to US Emergency Departments With Suspected SARS-CoV-2 | 1 | 1 | 1 | 1 | 2 | 1 | 1 | 1 | Good quality |
| Bertoglio et al | Poor Prognosis of COVID-19 Acute Respiratory Distress Syndrome in Lupus Erythematosus: Nationwide Cross-Sectional Population Study Of 252 119 Patients | 1 | 1 | 1 | 1 | 2 | 1 | 1 | 1 | Good quality |
| Bhaskaran et al | HIV infection and COVID-19 death: a population-based cohort analysis of UK primary care data and linked national death registrations within the OpenSAFELY platform | 1 | 1 | 1 | 1 | 2 | 1 | 1 | 1 | Good quality |
| Boulle | Risk factors for COVID-19 death in a population cohort study from the Western Cape Province, South Africa | 1 | 1 | 1 | 1 | 2 | 1 | 1 | 1 | Good quality |
| Brar et al | COVID-19 severity and outcomes in patients with cancer: A matched cohort study | 1 | 1 | 1 | 1 | 2 | 1 | 1 | 1 | Good quality |
| Bruera et al | Patients with systemic lupus erythematosus have an increased risk of mortality, mechanical ventilation, and hospitalization from COVID-19 | 1 | 1 | 1 | 1 | 2 | 1 | 1 | 1 | Good quality |
| Cabello et al | COVID-19 in people living with HIV: A multicenter case-series study | 1 | / | 1 | 1 | / | 1 | 1 | 1 | Poor quality |
| Caillard et al | Is COVID‐19 infection more severe in kidney transplant recipients? | 1 | / | 1 | 1 | 1 | 1 | 1 | 1 | Good quality |
| Chavaro et al | COVID‐19 severity in kidney transplant recipients is similar to nontransplant patients with similar comorbidities | 1 | / | 1 | 1 | 2 | 1 | 1 | 1 | Good quality |
| Chavez-MacGregor et al | Evaluation of COVID-19 mortality and adverse outcomes in US patients with or without cancer | 1 | 1 | 1 | 1 | 2 | 1 | 1 | 1 | Good quality |
| Chiriboga et al | Risk of COVID-19 Infection and Hospitalization in Patients With Inflammatory Rheumatic Disease Compared With the General Population | 1 | 1 | 1 | 1 | 2 | 1 | 1 | 1 | Good quality |
| Costa et al | Higher severity and risk of in‐hospital mortality for COVID‐19 patients with cancer during the year 2020 in Brazil: A countrywide analysis of secondary data | 1 | 1 | 1 | 1 | 1 | 1 | 1 | 1 | Good quality |
| Curtis et al | Characteristics, comorbidities, and outcomes of SARS-cov-2 infection in patients with autoimmune conditions treated with systemic therapies: a population-based study. | 1 | 1 | 1 | 1 | 2 | 1 | 1 | 1 | Good quality |
| D’Silva et al | COVID‐19 outcomes in patients with systemic autoimmune rheumatic diseases compared to the general population: a US multicenter, comparative cohort study | 1 | 1 | 1 | 1 | 2 | 1 | 1 | 1 | Good quality |
| Dai et al | Patients with Cancer Appear More Vulnerable to SARS-CoV-2: A Multicenter Study during the COVID-19 Outbreak | 1 | 1 | 1 | 1 | 2 | 1 | 1 | 1 | Good quality |
| de Azambuja et al | Impact of solid cancer on in‐hospital mortality overall and among different subgroups of patients with COVID‐19: a nationwide, population‐based analysis | 1 | 1 | 1 | 1 | 2 | 1 | 1 | 1 | Good quality |
| Durstenfeld et al | Impact of HIV Infection on COVID-19 Outcomes Among Hospitalized Adults in the U.S | 1 | 1 | 1 | 1 | 2 | 1 | 1 | 1 | Good quality |
| Fernandez-Cruz et al | Higher mortality of hospitalized haematologic patients with COVID-19 compared to non-haematologic is driven by thrombotic complications and development | 1 | 1 | 1 | 1 | 1 | 1 | 1 | 1 | Good quality |
| Ferri et al | Prevalence and death rate of covid-19 in autoimmune systemic diseases in the first three pandemic waves. relationship with disease subgroups | 1 | / | 1 | 1 | 2 | 1 | 1 | 1 | Good quality |
| Figueroa-Parra et al | Risk of severe COVID-19 outcomes associated with rheumatoid arthritis and phenotypic subgroups: a retrospective, comparative, multicentre cohort study | 1 | 1 | 1 | 1 | 1 | 1 | 1 | 1 | Good quality |
| Fisher et al | Outcomes of COVID‐19 in hospitalized solid organ transplant recipients compared to a matched cohort of non‐transplant patients at a national healthcare system in the United States | 1 | 1 | 1 | 1 | 2 | 1 | 1 | 1 | Good quality |
| Flannery et al | A comparison of COVID-19 inpatients by HIV status | 1 | 1 | 1 | 1 | / | 1 | 1 | 1 | Poor quality |
| Fu et al | COVID‐19 outcomes in hospitalized patients with active cancer: Experiences from a major New York City health care system | 1 | 1 | 1 | 1 | 1 | 1 | 1 | 1 | Good quality |
| Garneau et al | Clinical outcomes of patients previously treated with B-cell depletion therapy hospitalized with COVID-19: results from the Johns Hopkins Crown Registry | 1 | 1 | 1 | 1 | 2 | 1 | 1 | 1 | Good quality |
| Geretti et al | Outcomes of COVID-19 related hospitalization among people with HIV in the ISARIC WHO Clinical Characterization Protocol (UK): a prospective observational study | 1 | 1 | 1 | 1 | 2 | 1 | 1 | 1 | Good quality |
| Gisondi et al | Incidence rates of hospitalization and death from COVID-19 in patients with psoriasis receiving biological treatment: A Northern Italy experience | 1 | / | 1 | 1 | / | 1 | 1 | 1 | Poor quality |
| Hachem et al | Comparing the outcome of COVID-19 in cancer and non-cancer patients: An international multicenter study. | 1 | 1 | 1 | 1 | // | 1 | 1 | 1 | Poor quality |
| Hadi et al | Incidence, outcomes, and impact of COVID-19 on inflammatory bowel disease: propensity matched research network analysis | 1 | 1 | 1 | 1 | / | 1 | 1 | 1 | Poor quality |
| Hadi et al | Characteristics and outcomes of COVID-19 in patients with HIV: a multicentre research network study | 1 | 1 | 1 | 1 | 2 | 1 | 1 | 1 | Good quality |
| Hadi et al | Outcomes of COVID-19 in solid organ transplant recipients: a propensity-matched analysis of a large research network | 1 | 1 | 1 | 1 | 2 | 1 | 1 | 1 | Good quality |
| Hedberg et al | Incidence and severity of COVID-19 in adults with and without HIV diagnosis. | 1 | 1 | 1 | 1 | 2 | 1 | 1 | 1 | Good quality |
| Johannesen et al | COVID-19 in cancer patients, risk factors for disease and adverse outcome, a population-based study from Norway | 1 | 1 | 1 | 1 | 1 | 1 | 1 | 1 | Good quality |
| Kjeldsen et al | Outcome of COVID-19 in hospitalized patients with chronic inflammatory diseases. A population based national register study in Denmark | 1 | 1 | 1 | 1 | 2 | 1 | 1 | 1 | Good quality |
| Kodvanj et al | Inflammatory Bowel Disease Is Associated with an Increased Risk for Covid-19-Related Hospitalization, but Not with Mortality: Croatian Nationwide Cohort Study | 1 | 1 | 1 | 1 | / | 1 | 1 | 1 | Poor quality |
| Kridin et al | Nineteen months into the pandemic, what have we learned about COVID-19-related outcomes in patients with psoriasis? | 1 | 1 | 1 | 1 | 1 | 1 | 1 | 1 | Good quality |
| Kwapong et al | Effect of HIV status on ICU admission and mortality among hospitalized coronavirus disease 2019 (COVID-19) patients | 1 | 1 | 1 | 1 | 2 | 1 | 1 | 1 | Good quality |
| Lee et al | Comparative outcomes in hospital admissions with COVID-19 in people living with HIV and people living without HIV: a retrospective study | 1 | 1 | 1 | 1 | 2 | 1 | 1 | 1 | Good quality |
| Looha et al | The impact of HIV on the risk of COVID-19 death among hospitalized patients | 1 | 1 | 1 | 1 | 2 | 1 | 1 | 1 | Good quality |
| Lunski et al | Multivariate mortality analyses in COVID‐19: comparing patients with cancer and patients without cancer in Louisiana | 1 | 1 | 1 | 1 | 2 | 1 | 1 | 1 | Good quality |
| MacKenna et al | Risk of severe COVID-19 outcomes associated with immune-mediated inflammatory diseases and immune-modifying therapies: a nationwide cohort stud | 1 | 1 | 1 | 1 | 2 | 1 | 1 | 1 | Good quality |
| Mahdavi et al | Factors associated with COVID-19 and its outcome in patients with rheumatoid arthritis | 1 | / | 1 | 1 | / | 1 | 1 | 1 | Poor quality |
| Mangone et al | Cumulative COVID‐19 incidence, mortality and prognosis in cancer survivors: a population‐based study in Reggio Emilia, Northern Italy | 1 | 1 | 1 | 1 | / | 1 | 1 | 1 | Poor quality |
| Mansoor et al | Clinical Characteristics, Hospitalization and Mortality Rates of COVID-19 Among Liver Transplant Patients in the United States: A Multi-Center Research Network Study | 1 | 1 | 1 | 1 | 2 | 1 | 1 | 1 | Good quality |
| Marozoff et al | Severe COVID-19 outcomes among patients with autoimmune rheumatic diseases or transplantation: a population-based matched cohort study | 1 | 1 | 1 | 1 | 1 | 1 | 1 | 1 | Good quality |
| Martinez-Lopez et al | Multiple myeloma and SARS-CoV-2 infection: clinical characteristics and prognostic factors of inpatient mortality | 1 | 1 | 1 | 1 | 2 | 1 | 1 | 1 | Good quality |
| Miyashita et al | Prognosis of coronavirus disease 2019 (COVID-19) in patients with HIV infection in New York City | 1 | 1 | 1 | 1 | / | 1 | 1 | 1 | Poor quality |
| Miyashita et al | Do patients with cancer have a poorer prognosis of COVID-19? An experience in New York City. | 1 | 1 | 1 | 1 | / | 1 | 1 | 1 | Poor quality |
| Molnar et al | Outcomes of critically ill solid organ transplant patients with COVID‐19 in the United States | 1 | 1 | 1 | 1 | 2 | 1 | 1 | 1 | Good quality |
| Moreno-Torres et al | Predictors of in-hospital mortality in HIV-infected patients with COVID-19 | 1 | 1 | 1 | 1 | 2 | 1 | 1 | 1 | Good quality |
| Moreno-Torres et al | Systemic Autoimmune Diseases in Patients Hospitalized with COVID-19 in Spain: A Nation-Wide Registry Study | 1 | 1 | 1 | 1 | 2 | 1 | 1 | 1 | Good quality |
| Myint et al | Routine use of immunosuppressants is associated with mortality in hospitalised patients with COVID-19 | 1 | 1 | 1 | 1 | 2 | 1 | 1 | 1 | Good quality |
| Nair et al | An early experience on the effect of solid organ transplant status on hospitalized COVID-19 patients | 1 | 1 | 1 | 1 | 2 | 1 | 1 | 1 | Good quality |
| Olalla-Sierra et al | Coronavirus disease 2019 hospitalization outcomes in persons with and without HIV in Spain | 1 | 1 | 1 | 1 | 2 | 1 | 1 | 1 | Good quality |
| Osmanodja et al | Undoubtedly, kidney transplant recipients have a higher mortality due to COVID-19 disease compared to the general population | 1 | 1 | 1 | 1 | // | 1 | 1 | 1 | Poor quality |
| Ozturk et al | Mortality analysis of COVID-19 infection in chronic kidney disease, haemodialysis and renal transplant patients compared with patients without kidney disease: a nationwide analysis from Turkey | 1 | 1 | 1 | 1 | 2 | 1 | 1 | 1 | Good quality |
| Pablos et al | Clinical outcomes of hospitalised patients with COVID-19 and chronic inflammatory and autoimmune rheumatic diseases: a multicentric matched cohort study | 1 | 1 | 1 | 1 | 2 | 1 | 1 | 1 | Good quality |
| Pakhchanian et al | COVID-19 outcomes in patients with dermatomyositis: a registry-based cohort analysis | 1 | 1 | 1 | 1 | 2 | 1 | 1 | 1 | Good quality |
| Park | COVID-19 in the largest US HIV cohort. AIDS | 1 | 1 | 1 | 1 | 2 | 1 | 1 | 1 | Good quality |
| Parker et al | Clinical features and outcomes of COVID-19 admissions in a population with a high prevalence of HIV and tuberculosis: a multicentre cohort study | 1 | 1 | 1 | 1 | 2 | 1 | 1 | 1 | Good quality |
| Passamonti et al | Clinical characteristics and risk factors associated with COVID‐19 severity in patients with haematological malignancies in Italy: a retrospective, multicentre, cohort study. | 1 | / | 1 | 1 | 2 | 1 | 1 | 1 | Good quality |
| Patel | Clinical outcomes and inflammatory markers by HIV serostatus and viral suppression in a large cohort of patients hospi- talized with COVID-19 | 1 | 1 | 1 | 1 | 2 | 1 | 1 | 1 | Good quality |
| Peron | Covid-19 presentation and outcomes among cancer patients: A matched case-control study | 1 | 1 | 1 | 1 | 2 | 1 | 1 | 1 | Good quality |
| Qi et al | Clinical outcomes of COVID-19 patients with rheumatic diseases: a retrospective cohort study and synthesis analysis in Wuhan, China | 1 | 1 | 1 | 1 | 2 | 1 | 1 | 1 | Good quality |
| Raad et al | International Multicenter Study Comparing Cancer to Non-Cancer Patients with COVID-19: Impact of Risk Factors and Treatment Modalities on Survivorship | 1 | 1 | 1 | 1 | 2 | 1 | 1 | 1 | Good quality |
| Raez et al | Mortality and prognostic factors in hospitalized COVID-19 patients with cancer: an analysis from a large healthcare system in the United States | 1 | 1 | 1 | 1 | 2 | 1 | 1 | 1 | Good quality |
| Raiker et al | 254 COVID‐19 related outcomes in psoriasis and psoriasis arthritis patients. | 1 | 1 | 1 | 1 | 2 | 1 | 1 | 1 | Good quality |
| Raiker et al | Outcomes of COVID-19 in patients with rheumatoid arthritis: A multicenter research network study in the United States | 1 | 1 | 1 | 1 | 2 | 1 | 1 | 1 | Good quality |
| Ranabothu et al | Outcomes of COVID-19 in solid organ transplants | 1 | 1 | 1 | 1 | / | 1 | 1 | 1 | Poor quality |
| Rasmussen et al | Outcomes following SARS-CoV-2 infection among individuals living with and without HIV; a Danish nationwide cohort study | 1 | / | 1 | 1 | 1 | 1 | 1 | 1 | Good quality |
| Rorat et al | The course of COVID-19 in patients with systemic autoimmune rheumatic diseases | 1 | 1 | 1 | 1 | 2 | 1 | 1 | 1 | Good quality |
| Rosenthal et al | Factors associated with SARS-CoV-2-related hospital outcomes among and between persons living with and without diagnosed HIV infection in New York State | 1 | 1 | 1 | 1 | 2 | 1 | 1 | 1 | Good quality |
| Rugge et al | SARS-CoV-2 infection in the Italian Veneto region: adverse outcomes in patients with cancer. | 1 | 1 | 1 | 1 | 1 | 1 | 1 | 1 | Good quality |
| Rutter et al | COVID-19 infection, admission and death among people with rare autoimmune rheumatic disease in England: results from the RECORDER project | 1 | / | 1 | 1 | 1 | 1 | 1 | 1 | Good quality |
| Sahota et al | Incidence, Risk Factors, and Outcomes of COVID-19 Infection in a Large Cohort of Solid Organ Transplant Recipients | 1 | 1 | 1 | 1 | 2 | 1 | 1 | 1 | Good quality |
| Seyyedsalehi et al | Hospital and post-discharge mortality in COVID-19 patients with a preexisting cancer diagnosis in Iran | 1 | 1 | 1 | 1 | 2 | 1 | 1 | 1 | Good quality |
| Shi et al | Association of Cancer with Risk and Mortality of COVID-19: Results from the UK Biobank | 1 | 1 | 1 | 1 | 1 | 1 | 1 | 1 | Good quality |
| Shin et al | Autoimmune inflammatory rheumatic diseases and COVID-19 out- comes in South Korea: a nationwide cohort study. | 1 | 1 | 1 | 1 | 2 | 1 | 1 | 1 | Good quality |
| Sigel et al | Covid-19 and people with HIV infection: outcomes for hospitalized patients in New York City | 1 | 1 | 1 | 1 | 2 | 1 | 1 | 1 | Good quality |
| Spence et al | COVID-19 Outcomes in a US Cohort of Persons Living with HIV (PLWH) | 1 | 1 | 1 | 1 | 2 | 1 | 1 | 1 | Good quality |
| Suarez-Garcia et al | In-hospital mortality among immunosuppressed patients with COVID-19: Analysis from a national cohort in Spain | 1 | 1 | 1 | 1 | 2 | 1 | 1 | 1 | Good quality |
| Sun et al | COVID-19 HOSPITALIZATION AMONG PEOPLE WITH HIV OR SOLID ORGAN TRANSPLANT IN THE US. | 1 | 1 | 1 | 1 | 2 | 1 | 1 | 1 | Good quality |
| Sun et al | COVID-19 Disease Severity among People with HIV Infection or Solid Organ Transplant in the United States: A Nationally-representative, Multicenter, Observational Cohort Study | 1 | 1 | 1 | 1 | 2 | 1 | 1 | 1 | Good quality |
| Swan et al | Hospitalization and survival of solid organ transplant recipients with coronavirus disease 2019: A propensity matched cohort study | 1 | 1 | 1 | 1 | 2 | 1 | 1 | 1 | Good quality |
| Tang et al | People with HIV have a higher risk of COVID-19 diagnosis but similar outcomes to the general population | 1 | 1 | 1 | 1 | 2 | 1 | 1 | 1 | Good quality |
| Tesoriero et al | COVID-19 Outcomes Among Persons Living With or Without Diagnosed HIV Infection in New York State | 1 | 1 | 1 | 1 | 2 | 1 | 1 | 1 | Good quality |
| Thompson et al | Investigation Into the Effect of COVID-19 Infection on Length of Hospital Stay and Mortality in Patients With Rheumatoid Arthritis | 1 | 1 | 1 | 1 | 2 | 1 | 1 | 1 | Good quality |
| Topless et al | Gout, Rheumatoid Arthritis, and the Risk of Death Related to Coronavirus Disease 2019: An Analysis of the UK Biobank | 1 | 1 | 1 | 1 | 2 | 1 | 1 | 1 | Good quality |
| Udovica et al | High mortality in patients with active malignancy and severe COVID-19: Results from an Austrian multicenter registry during the first period of the COVID-19 … | 1 | 1 | 1 | 1 | 2 | 1 | 1 | 1 | Good quality |
| Ungaro et al | Autoimmune and chronic inflammatory disease patients with COVID‐19. | 1 | 1 | 1 | 1 | 2 | 1 | 1 | 1 | Good quality |
| Webb et al | Liver transplantation does not significantly increase risk of mortality from SARS-CoV-2 infection: International registry data | 1 | / | 1 | 1 | / | 1 | 1 | 1 | Poor quality |
| Westblade et al | SARS-CoV-2 viral load predicts mortality in patients with and without cancer who are hospitalized with COVID-19 | 1 | 1 | 1 | 1 | 1 | 1 | 1 | 1 | Good quality |
| Yang et al | Associations between HIV infection and clinical spectrum of COVID-19: a population level analysis based on US National COVID Cohort Collaborative (N3C) data | 1 | 1 | 1 | 1 | 2 | 1 | 1 | 1 | Good quality |
| Yendewa et al | Clinical Features and Outcomes of Coronavirus Disease 2019 Among People With Human Immunodeficiency Virus in the United States: A Multicenter Study From a Large Global Health Research Network (TriNetX) | 1 | 1 | 1 | 1 | 2 | 1 | 1 | 1 | Good quality |
| Yigenoglu et al | The outcome of COVID-19 in patients with hematological malignancy. | 1 | 1 | 1 | 1 | 2 | 1 | 1 | 1 | Good quality |
| Yousaf et al | Clinical outcomes of COVID‐19 in patients taking tumor necrosis factor inhibitors or methotrexate: a multicenter research network study. | 1 | 1 | 1 | 1 | 2 | 1 | 1 | 1 | Good quality |
| Zanetti et al | Increased COVID-19 mortality in patients with rheumatic diseases: results from the CONTROL-19 study by the Italian Society for Rheumatology | 1 | 1 | 1 | 1 | 1 | 1 | 1 | 1 | Good quality |
