# Supplementary material for: Disparities in COVID-19 mortality amongst the immunosuppressed: A systematic review and meta-analysis for enhanced disease surveillance
Source: J Infect. 2024 Mar;88(3):None. doi: 10.1016/j.jinf.2024.01.009 (PMC10943183; doi:10.1016/j.jinf.2024.01.009)
Supplement: Supplementary file 5 — Supplementary material [file mmc5.docx]

**Appendix 5: Data Extraction Forms**

1. Transplantation data extraction form

| **Authors** | **Title** | **Year** | **Income** | **IS Type** | **Exposed age** | **Unexposed age** | **Case** | **Time to mortality** | **Exposed (n)** | **Unexposed (n)** | **Exposed death** | **Exposed survivors** | **Unexposed death** | **Unexposed survivors** | **Effect Measure** | **Value** | **LB** | **UB** | **p** | **Matched** | **Adjusted criteria met** |
| --- | --- | --- | --- | --- | --- | --- | --- | --- | --- | --- | --- | --- | --- | --- | --- | --- | --- | --- | --- | --- | --- |
| Caillard et al | Is COVID‐19 infection more severe in kidney transplant recipients? | 2021 | H | Kidney | 62 | 63 | In-hospital | 30 day | 273 | 273 | 55 | 218 | 37 | 236 | HR | 1.55 | 1.02 | 2.35 | 0.039 | Yes | Y |
| Chavarot et al | COVID‐19 severity in kidney transplant recipients is similar to nontransplant patients with similar comorbidities | 2022 | H | Kidney | 67.2 | 65.1 | In-hospital | 30 day | 83 | 83 | 31 | 52 | 24 | 59 | sHR | 1.375 | 0.67 | 2.83 | 0.4 | Yes | Y |
| Fisher et al | Outcomes of COVID-19 in hospitalized solid organ transplant recipients compared to a matched cohort of non-transplant pa- tients at a national healthcare system in the United States | 2021 | H | Mixed Cohort | 60 | 60 | In-hospital |  | 128 | 3907 |  |  |  |  | OR | 1.93 | 1.18 | 3.15 | <0.01 | Yes | Y |
| Hadi et al | Outcomes of COVID-19 in solid organ transplant recipients: a propensity-matched analysis of a large research network | 2022a | H | Mixed Cohort | 54.5 | 55.2 | In-hospital | 60 day | 2289 | 2289 | 138 | 2151 | 132 | 2157 | RR | 0.99 | 0.73 | 1.34 |  | Yes | Y |
| Hadi et al | Outcomes of COVID-19 in solid organ transplant recipients: a propensity-matched analysis of a large research network | 2022b | H | Kidney | 52 | 30 | In-hospital | 30 day | 1511 | 2289 | 57 | 1454 | 110 | 2179 |  |  |  |  |  | Yes | Y |
| Hadi et al | Outcomes of COVID-19 in solid organ transplant recipients: a propensity-matched analysis of a large research network | 2022c | H | Other |  |  | In-hospital | 30 day | 183 | 2289 | 13 | 170 | 110 | 2179 |  |  |  |  |  | Yes | Y |
| Mansoor et al | Clinical Characteristics, Hospitalization and Mortality Rates of COVID-19 Among Liver Transplant Patients in the United States: A Multi-Center Research Network Study | 2020 | H | Liver | 57.03 | 59.83 | All Cases |  | 125 | 125 | 10 | 115 | 10 | 115 | RR | 1 | 0.43 | 2.32 | 1 | Yes | Y |
| Marozoff et al | Severe COVID-19 outcomes among patients with autoimmune rheumatic diseases or transplantation: a population-based matched cohort study | 2022 | H | Mixed Cohort | 54.3 | 54 | All Cases |  | 222 | 1106 | 27 | 195 | 24 | 1082 | OR | 5.48 | 2.82 | 10.63 |  | Yes | Y |
| Molnar et al | Outcomes of critically ill solid organ transplant patients with COVID‐19 in the United States | 2020 | H | Mixed Cohort | 58 | 61 | In-hospital | 28 day | 98 | 288 | 39 | 59 | 124 | 164 | RR | 0.92 | 0.7 | 1.22 | 0.58 | Yes | Y |
| Nair et al | An early experience on the effect of solid organ transplant status on hospitalized COVID-19 patients | 2021 | H | Mixed Cohort | 61.8 | 62.7 | In-hospital |  | 82 | 1625 | 26 | 56 | 391 | 1234 | OR | 1.34 | 1.03 | 1.74 | 0.027 | Yes | Y |
| Osmanodja et al | Undoubtedly, kidney transplant recipients have a higher mortality due to COVID-19 disease compared to the general population | 2021 | H | Kidney |  |  | All Cases |  | 114 | 209960 | 10 | 113 | 6002 | 203958 |  |  |  |  |  | No | N |
| Ozturk et al | Mortality analysis of COVID-19 infection in chronic kidney disease, haemodialysis and renal transplant patients compared with patients without kidney disease: a nationwide analysis from Turkey | 2020 | L | Kidney | 48 | 51 | In-hospital | 28 day | 81 | 450 |  |  |  |  | HR | 1.897 | 0.761 | 4.727 | 0.138 | Yes | Y |
| Ranabothu et al | Outcomes of COVID-19 in solid organ transplants | 2020a | H | Mixed Cohort | 55.4 | 47.6 | All Cases | 30 day | 288 | 30285 | 32 | 256 | 1155 | 29130 | OR | 3.15 | 2.17 | 4.57 | <0.0001 | No | Y |
| Ranabothu et al | Outcomes of COVID-19 in solid organ transplants | 2020b | H | Kidney | 55.4 | 47.6 | All Cases | 30 day | 224 | 30285 | 21 | 203 | 1155 | 29130 |  |  |  |  |  | No | N |
| Sahota et al | Incidence, Risk Factors, and Outcomes of COVID-19 Infection in a Large Cohort of Solid Organ Transplant Recipients | 2022a | H | Mixed Cohort | 55.7 | 57.3 | All Cases |  | 600 | 312011 | 88 | 512 | 5615 | 306396 |  |  |  |  |  | No | N |
| Sahota et al | Incidence, Risk Factors, and Outcomes of COVID-19 Infection in a Large Cohort of Solid Organ Transplant Recipients | 2022b | H | Kidney |  |  | All Cases |  | 438 | 312011 | 65 | 373 | 5615 | 306396 |  |  |  |  |  | No | N |
| Sahota et al | Incidence, Risk Factors, and Outcomes of COVID-19 Infection in a Large Cohort of Solid Organ Transplant Recipients | 2022c | H | Liver | 62 | 57.3 | All Cases |  | 114 | 312011 | 10 | 104 | 5615 | 306396 |  |  |  |  |  | No | N |
| Suarez-Garcia et al | In-hospital mortality among immunosuppressed patients with COVID-19: Analysis from a national cohort in Spain | 2021 | H | Mixed Cohort | 63.5 | 66.5 | In-hospital |  | 166 | 11095 | 57 | 109 | 2143 | 8952 | OR | 3.12 | 2.23 | 4.36 | <0.001 | No | Y |
| Sun et al | COVID-19 Disease Severity among People with HIV Infection or Solid Organ Transplant in the United States: A Nationally-representative, Multicenter, Observational Cohort Study | 2021 | H | Mixed Cohort | 57 | 47 | All Cases | 45 day | 11392 | 1426984 | 875 | 10517 | 23831 | 1403153 | OR | 3.38 | 3.35 | 3.41 | <0.01 | No | Y |
| Swan et al | Hospitalization and survival of solid organ transplant recipients with coronavirus disease 2019: A propensity matched cohort study | 2022 | H | Mixed Cohort | 57 | 58 | In-hospital | 60 day | 100 | 500 | 16 | 84 | 70 | 430 | OR | 1.15 | 0.64 | 2.08 | 0.64 | Yes | Y |
| Webb et al | Liver transplantation does not significantly increase risk of mortality from SARS-CoV-2 infection: International registry data | 2020 | H | Liver | 60 | 73 | All Cases |  | 151 | 627 | 28 | 123 | 167 | 460 |  |  |  |  |  | No | N |

1. Malignancy data extraction form

| **Authors** | **Title** | **Year** | **Income** | **IS Type** | **Exposed age** | **Unexposed age** | **Case** | **Time to mortality** | **Exposed (n)** | **Unexposed (n)** | **Exposed death** | **Exposed survivors** | **Unexposed death** | **Unexposed survivors** | **Effect Measure** | **Value** | **LB** | **UB** | **p** | **Matched** | **Adjusted criteria met** |
| --- | --- | --- | --- | --- | --- | --- | --- | --- | --- | --- | --- | --- | --- | --- | --- | --- | --- | --- | --- | --- | --- |
| Alpert et al | Clinical course of cancer patients with COVID-19: a retrospective cohort study | 2021 | H | Mixed cohort |  |  | In-hospital |  | 420 | 840 |  |  |  |  | OR | 1.12 | 0.86 | 1.45 | 1 | Yes | Y |
| Brar et al | COVID-19 severity and outcomes in patients with cancer: A matched cohort study | 2020 | H | Mixed cohort | 72.5 | 71.2 | In-hospital |  | 117 | 468 | 29 | 88 | 100 | 368 | HR | 0.98 | 0.58 | 1.67 |  | Yes | Y |
| Costa et al | Higher severity and risk of in‐hospital mortality for COVID‐19 patients with cancer during the year 2020 in Brazil: A countrywide analysis of secondary data | 2021b | L | Haematology |  |  | In-hospital |  | 944 | 315410 |  |  |  |  | OR | 2.85 | 2.41 | 3.38 | <0.001 | No | N |
| Costa et al | Higher severity and risk of in‐hospital mortality for COVID‐19 patients with cancer during the year 2020 in Brazil: A countrywide analysis of secondary data | 2021a | L | Mixed cohort | 67 | 62 | In-hospital |  | 7406 | 315410 | 4479 | 2927 | 115066 | 200344 | OR | 1.94 | 1.83 | 2.06 | <0.001 | No | N |
| Costa et al | Higher severity and risk of in‐hospital mortality for COVID‐19 patients with cancer during the year 2020 in Brazil: A countrywide analysis of secondary data | 2021c | L | Solid |  |  | In-hospital |  | 6461 | 315410 |  |  |  |  | OR | 1.83 | 1.72 | 1.95 | <0.001 | No | N |
| Dai et al | Patients with Cancer Appear More Vulnerable to SARS-CoV-2: A Multicenter Study during the COVID-19 Outbreak | 2020 | H | Mixed cohort | 64 | 63.5 | In-hospital |  | 105 | 536 | 12 | 93 |  |  | OR | 2.171 | 0.806 | 5.149 | 0.064 | Yes | Y |
| de Azambuja | Impact of solid cancer on in‐hospital mortality overall and among different subgroups of patients with COVID‐19: a nationwide, population‐based analysis | 2020 | H | Solid | 75 | 70 | In-hospital | 30 day | 892 | 9594 | 283 | 609 | 1922 | 7672 | OR | 1.34 | 1.13 | 1.58 | <0.001 | No | Y |
| Fernández-Cruz et al | Higher mortality of hospitalized haematologic patients with COVID-19 compared to non-haematologic is driven by thrombotic complications and development | 2022 | H | Haematology | 70.7 | 69.6 | In-hospital |  | 71 | 142 | 27 | 44 | 26 | 116 |  |  |  |  |  | Yes | N |
| Fu et al | COVID‐19 outcomes in hospitalized patients with active cancer: Experiences from a major New York City health care system | 2021b | H | Haematology |  |  | All Cases |  | 69 | 3953 | 33 | 36 | 683 | 3270 |  |  |  |  |  | No | N |
| Fu et al | COVID‐19 outcomes in hospitalized patients with active cancer: Experiences from a major New York City health care system | 2021a | H | Mixed cohort | 71.2 | 62.2 | All Cases |  | 233 | 3953 | 80 | 153 | 683 | 2777 | OR | 1.89 | 1.33 | 2.67 |  | No | N |
| Fu et al | COVID‐19 outcomes in hospitalized patients with active cancer: Experiences from a major New York City health care system | 2021c | H | Solid |  |  | All Cases |  | 164 | 3953 | 48 | 121 | 158 | 7705 |  |  |  |  |  | No | N |
| Hachem et al | Comparing the outcome of COVID-19 in cancer and non-cancer patients: An international multicenter study. | 2020 | H | Mixed cohort |  |  | All Cases | 30 day | 186 | 385 | 37 | 149 | 42 | 343 |  |  |  |  |  | No | N |
| 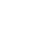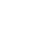  Johannesen et al | COVID-19 in cancer patients, risk factors for disease and adverse outcome, a population-based study from Norway | 2021b | H | Haematology |  |  | All Cases |  | 54 | 7863 | 3 | 51 | 158 | 7705 | OR | 1 | 0.28 | 3.65 |  | No | Y |
| Johannesen et al | COVID-19 in cancer patients, risk factors for disease and adverse outcome, a population-based study from Norway | 2021a | H | Mixed cohort |  |  | All Cases |  | 547 | 7863 | 56 | 491 | 158 | 7705 | OR | 0.99 | 0.68 | 1.42 |  | No | Y |
| Johannesen et al | COVID-19 in cancer patients, risk factors for disease and adverse outcome, a population-based study from Norway | 2021c | H | Solid |  |  | All Cases |  | 493 | 7863 | 53 | 440 | 158 | 7705 | OR | 0.99 | 0.68 | 1.44 |  | No | Y |
| 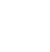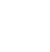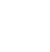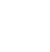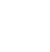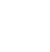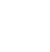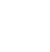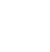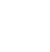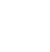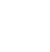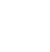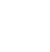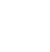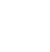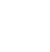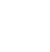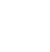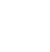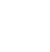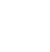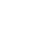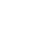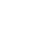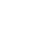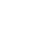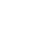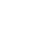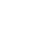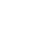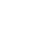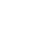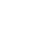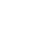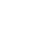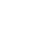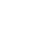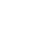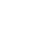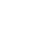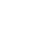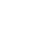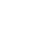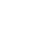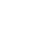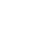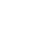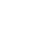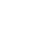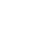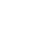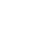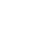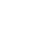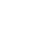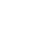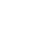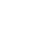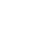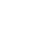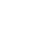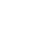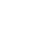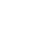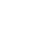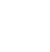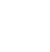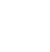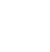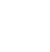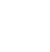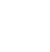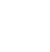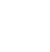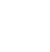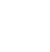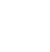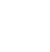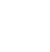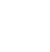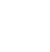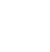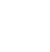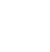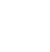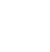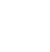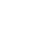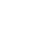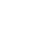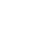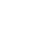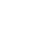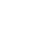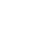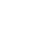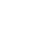  Lunski et al | Multivariate mortality analyses in COVID‐19: comparing patients with cancer and patients without cancer in Louisiana | 2020a | H | Mixed cohort |  |  | All Cases |  | 312 | 4833 | 66 | 246 | 418 | 4415 | OR | 2.03 | 1.44 | 2.87 |  | No | Y |
| Lunski et al | Multivariate mortality analyses in COVID‐19: comparing patients with cancer and patients without cancer in Louisiana | 2020b | H | Mixed cohort |  |  | In-hospital |  | 157 | 1460 | 56 | 101 | 372 | 1088 | OR | 1.36 | 0.89 | 2.08 |  | No | Y |
| Mangone et al | Cumulative COVID‐19 incidence, mortality and prognosis in cancer survivors: a population‐based study in Reggio Emilia, Northern Italy | 2021 | H | Mixed cohort |  |  | All Cases |  | 447 | 4094 | 113 | 334 | 428 | 3666 | OR | 1.54 | 1.18 | 2 |  | No | N |
| Martinez-Lopez et al | Multiple myeloma and SARS-CoV-2 infection: clinical characteristics and prognostic factors of inpatient mortality | 2020 | H | Haematology | 71 | 71 | In-hospital |  | 167 | 167 | 56 | 111 | 38 | 129 |  |  |  |  |  | Yes | N |
| Miyashita et al | Do patients with cancer have a poorer prognosis of COVID-19? An experience in New York City. | 2020 | H | Mixed cohort |  |  | In-hospital |  | 334 | 5354 | 37 | 297 | 518 | 4836 | RR | 1.15 | 0.84 | 1.57 |  | No | N |
| Passamonti et al | Clinical characteristics and risk factors associated with COVID‐19 severity in patients with haematological malignancies in Italy: a retrospective, multicentre, cohort study. | 2020 | H | Haematology | 68 |  | In-hospital |  | 536 | 29282 | 198 | 338 |  |  | SMR | 2.04 | 1.77 | 2.34 |  | No | N |
| Péron et al | Covid-19 presentation and outcomes among cancer patients: A matched case-control study | 2021 | H | Mixed cohort | 76 | 77 | In-hospital | 60 day | 108 | 193 | 43 | 65 | 54 | 139 | OR | 2.00 | 1.20 | 3.40 | 0.0084 | Yes | Y |
| Raad et al | International Multicenter Study Comparing Cancer to Non-Cancer Patients with COVID-19: Impact of Risk Factors and Treatment Modalities on Survivorship | 2022 | H | Mixed cohort | 61 | 50 | All Cases | 30 day | 1115 | 2851 | 122 | 993 | 226 | 2625 | OR | 1.46 | 1.03 | 2.07 | 0.035 | No | N |
| Raez et al | Mortality and prognostic factors in hospitalized COVID-19 patients with cancer: an analysis from a large healthcare system in the United States | 2022 | H | Mixed cohort | 71 | 60 | In-hospital |  | 265 | 4605 | 63 | 202 | 525 | 4080 | OR | 1.48 | 1.08 | 2.01 | 0.014 | No | Y |
| Rugge et al | SARS-CoV-2 infection in the Italian Veneto region: adverse outcomes in patients with cancer. | 2020a | H | Mixed cohort |  |  | All Cases |  | 723 | 8552 | 106 | 617 | 385 | 8167 |  |  |  |  |  | No | N |
| Rugge et al | SARS-CoV-2 infection in the Italian Veneto region: adverse outcomes in patients with cancer. | 2020b | H | Haematology |  |  | All Cases |  | 81 | 8552 | 13 | 68 | 385 | 8167 | OR | 2.39 | 1.24 | 4.58 |  | No | N |
| Seyyedsalehi et al | Hospital and post-discharge mortality in COVID-19 patients with a preexisting cancer diagnosis in Iran | 2022a | L | Mixed cohort | 58.16 | 57.87 | In-hospital | 60 day | 1090 | 5517 | 497 | 593 | 847 | 4670 | OR | 4.40 | 3.80 | 5.20 |  | No | Y |
| Seyyedsalehi et al | Hospital and post-discharge mortality in COVID-19 patients with a preexisting cancer diagnosis in Iran | 2022b | L | Haematology | 51.6 | 57.87 | In-hospital | 60 day | 423 | 5517 | 172 | 251 | 847 | 4670 | OR | 5.07 | 3.95 | 6.51 |  | No | Y |
| Seyyedsalehi et al | Hospital and post-discharge mortality in COVID-19 patients with a preexisting cancer diagnosis in Iran | 2022c | L | Solid | 62.3 | 57.87 | In-hospital | 60 day | 667 | 5517 | 233 | 434 | 847 | 4670 | OR | 4.13 | 3.41 | 5.01 |  | No | Y |
| Shi | Association of Cancer with Risk and Mortality of COVID-19: Results from the UK Biobank | 2020 | H | Mixed cohort | 61.36 | 56.11 | All Cases |  | 256 | 1306 | 46 | 210 | 160 | 1146 | OR | 1.04 | 0.71 | 1.51 | 0.83 | No | N |
| Suarez-Garcia et al | In-hospital mortality among immunosuppressed patients with COVID-19: Analysis from a national cohort in Spain | 2021a | H | Haematology | 71 | 66.5 | In-hospital |  | 358 | 11095 | 139 | 219 | 2143 | 8952 | OR | 2.31 | 1.76 | 3.03 | <0.001 | Yes | Y |
| Suarez-Garcia et al | In-hospital mortality among immunosuppressed patients with COVID-19: Analysis from a national cohort in Spain | 2021b | H | Solid | 73.2 | 66.5 | In-hospital |  | 1081 | 11095 | 343 | 738 | 2143 | 8952 | OR | 1.39 | 1.18 | 1.63 | <0.001 | Yes | Y |
| Sun et al | Rates of COVID-19-related Outcomes in Cancer compared to non-Cancer Patients | 2020 | H | Mixed cohort | 62 | 50 | All Cases | 30 day | 67 | 256 | 9 | 58 | 4 | 252 | OR | 5.67 | 1.49 | 21.59 |  | No | Y |
| Udovica et al | High mortality in patients with active malignancy and severe COVID-19: Results from an Austrian multicenter registry during the first period of the COVID-19 … | 2022 | H | Mixed cohort | 74 | 76 | In-hospital |  | 89 | 5386 | 41 | 48 | 43 | 113 | OR | 4.59 | 2.26 | 9.3 | <0.001 | No | Y |
| Westblade et al | SARS-CoV-2 viral load predicts mortality in patients with and without cancer who are hospitalized with COVID-19 | 2020a | H | Mixed cohort | 72 | 65 | In-hospital |  | 100 | 2914 | 30 | 70 | 725 | 2189 |  |  |  |  |  | No | N |
| Westblade et al | SARS-CoV-2 viral load predicts mortality in patients with and without cancer who are hospitalized with COVID-19 | 2020b | H | Solid |  |  | In-hospital |  | 60 | 2914 | 18 | 42 | 725 | 2189 | OR |  |  |  |  | No | N |
| Yigenoglu et al | The outcome of COVID-19 in patients with hematological malignancy. | 2021 | L | Haematology | 56 | 56 | All Cases |  | 740 | 740 | 102 | 638 | 50 | 690 |  |  |  |  |  | Yes | Y |

1. Immunosuppressive Agents data extraction form

| **Authors** | **Title** | **Year** | **Income** | **IS Type** | **Exposed age** | **Unexposed age** | **Case** | **Time to mortality** | **Exposed (n)** | **Unexposed (n)** | **Exposed death** | **Exposed survivors** | **Unexposed death** | **Unexposed survivors** | **Effect Measure** | **Value** | **LB** | **UB** | **p** | **Matched** | **Adjusted criteria met** |
| --- | --- | --- | --- | --- | --- | --- | --- | --- | --- | --- | --- | --- | --- | --- | --- | --- | --- | --- | --- | --- | --- |
| Belleudi et al | Direct and indirect impact of COVID-19 for patients with immune-mediated inflammatory diseases: a retrospective cohort study | 2021 | H | Biologics | 56 | 51 | All Cases |  | 9176 | 4702567 |  |  |  |  | OR | 2 | 1.11 | 3.63 |  | No | Y |
| Chavez-MacGregor | Evaluation of COVID-19 mortality and adverse outcomes in US patients with or without cancer | 2021 | H | Cancer treatment | 66 | 48 | All Cases | 30 day | 4296 | 493020 | 335 | 3961 | 24651 | 468369 | OR | 1.74 | 1.54 | 1.96 | <0.001 | No | Y |
| Garneau et al | Clinical outcomes of patients previously treated with B-cell depletion therapy hospitalized with COVID-19: results from the Johns Hopkins Crown Registry | 2022 | H | Unspecified | 57.4 | 57.4 | In-hospital | 30 day | 50 | 212 | 3 | 47 | 9 | 203 |  |  |  |  |  | Yes | Y |
| Myint et al | Routine use of immunosuppressants is associated with mortality in hospitalised patients with COVID-19 | 2021 | H | Unspecified | 74 |  | In-hospital |  | 118 | 1066 |  |  |  |  | HR | 1.87 | 1.3 | 2.69 | 0.001 | No | Y |
| Suarez-Garcia et al | In-hospital mortality among immunosuppressed patients with COVID-19: Analysis from a national cohort in Spain | 2021a | H | Unspecified | 71 | 66.5 | In-hospital |  | 2111 | 11095 | 661 | 1450 | 2143 | 8952 | OR | 1.6 | 1.43 | 1.79 | <0.001 | No | Y |
| Suarez-Garcia et al | In-hospital mortality among immunosuppressed patients with COVID-19: Analysis from a national cohort in Spain | 2021b | H | Systemic steroid |  |  | In-hospital |  | 570 | 11095 | 202 | 368 | 2143 | 8952 | OR | 2.16 | 1.8 | 2.61 |  | No | Y |
| Suarez-Garcia et al | In-hospital mortality among immunosuppressed patients with COVID-19: Analysis from a national cohort in Spain | 2021c | H | Biologics |  |  | In-hospital |  | 183 | 11095 | 49 | 134 | 2143 | 8952 | OR | 1.97 | 1.33 | 2.91 |  | No | Y |
| Suarez-Garcia et al | In-hospital mortality among immunosuppressed patients with COVID-19: Analysis from a national cohort in Spain | 2021d | H | Unspecified |  |  | In-hospital |  | 394 | 11095 | 109 | 285 | 2143 | 8952 | OR | 2.06 | 1.64 | 2.6 |  | No | Y |
| Yousaf et al | Clinical outcomes of COVID‐19 in patients taking tumor necrosis factor inhibitors or methotrexate: a multicenter research network study. | 2021 | H | Unspecified | 55.1 | 54.9 | All Cases |  | 213 | 213 | 13 | 200 | 15 | 198 | RR | 0.87 | 0.42 | 1.78 | 0.6958 | Yes | Y |

1. Rheumatological conditions data extraction form

| **Authors** | **Title** | **Year** | **Income** | **IS Type** | **Exposed age** | **Unexposed age** | **Case** | **Time to mortality** | **Exposed (n)** | **Unexposed (n)** | **Exposed death** | **Exposed survivors** | **Unexposed death** | **Unexposed survivors** | **Effect Measure** | **Value** | **LB** | **UB** | **p** | **Matched** | **Adjusted criteria met** |
| --- | --- | --- | --- | --- | --- | --- | --- | --- | --- | --- | --- | --- | --- | --- | --- | --- | --- | --- | --- | --- | --- |
| \| Attauabi et al \| \| --- \| | Outcomes and long-term effects of COVID-19 in patients with inflammatory bowel diseases—A Danish prospective population-based cohort study with individual-level data | 2022a | H | Gut | 48 |  | All Cases |  | 319 | 230087 | 13 | 306 | 2404 | 227683 | RR | 3.9 | 2.89 | 6.65 |  | No | Y |
| Attauabi et al | Outcomes and long-term effects of COVID-19 in patients with inflammatory bowel diseases—A Danish prospective population-based cohort study with individual-level data | 2022b | H | Gut | 44 |  | All Cases |  | 197 | 230087 | 2 | 195 | 2404 | 227683 | RR | 0.97 | 0.24 | 3.86 |  | No | Y |
| Belleudi et al | [Direct and indirect impact of COVID-19 for patients with immune-mediated inflammatory diseases: a retrospective cohort study](https://www.mdpi.com/2077-0383/10/11/2388) | 2022a | H | General | 59 | 51 | All Cases |  | 65230 | 4702567 |  |  |  |  | OR | 1.22 | 0.95 | 1.56 |  | No | Y |
| Belleudi et al | Direct and indirect impact of COVID-19 for patients with immune-mediated inflammatory diseases: a retrospective cohort study | 2022c | H | Gut | 53 | 51 | All Cases |  | 22525 | 4702567 |  |  |  |  | OR | 0.95 | 0.56 | 1.59 |  | No | Y |
| Belleudi et al | [Direct and indirect impact of COVID-19 for patients with immune-mediated inflammatory diseases: a retrospective cohort study](https://www.mdpi.com/2077-0383/10/11/2388) | 2022b | H | Joint | 64 | 51 | All Cases |  | 20299 | 4702567 |  |  |  |  | OR | 1.25 | 0.84 | 1.85 |  | No | Y |
| Belleudi et al | Direct and indirect impact of COVID-19 for patients with immune-mediated inflammatory diseases: a retrospective cohort study | 2022d | H | Skin | 60 | 51 | All Cases |  | 22406 | 4702567 |  |  |  |  | OR | 1.4 | 0.94 | 2.1 |  | No | Y |
| \| Bertoglio et al \| \| --- \| | Poor Prognosis of COVID-19 Acute Respiratory Distress Syndrome in Lupus Erythematosus: Nationwide Cross-Sectional Population Study Of 252 119 Patients | 2022 | L | General | 45.5 | 58.7 | In-hospital |  | 319 | 251800 | 126 | 193 | 82895 | 168905 | RR | 1.738 | 1.557 | 1.914 |  | No | Y |
| Bruera et al | [Patients with systemic lupus erythematosus have an increased risk of mortality, mechanical ventilation, and hospitalization from COVID-19](https://www.mdpi.com/2077-0383/10/11/2388) | 2022 | H | General |  |  | All Cases | 30 day | 687 | 6870 | 25 | 662 | 124 | 6746 | OR | 1.39 | 0.79 | 2.44 |  | Yes | Y |
| \| Chiriboga et al \| \| --- \| | [Risk of COVID-19 Infection and Hospitalization in Patients With Inflammatory Rheumatic Disease Compared With the General Population](https://journals.lww.com/jclinrheum/Fulltext/2022/03000/Risk_of_COVID_19_Infection_and_Hospitalization_in.63.aspx) | 2022 | H | General | 61.8 | 40.2 | In-hospital |  | 470 | 26434 | 63 | 407 | 899 | 25535 | RR | 0.25 | 0.06 | 1 |  | No | N |
| Curtis et al | Characteristics, comorbidities, and outcomes of SARS-cov-2 infection in patients with autoimmune conditions treated with systemic therapies: a population-based study. | 2022c | H | Gut | 52 | 49 | All Cases |  | 811 | 311563 | 17 | 794 | 5226 | 306337 | OR | 1.11 | 0.68 | 1.82 |  | No | Y |
| Curtis et al | Characteristics, comorbidities, and outcomes of SARS-cov-2 infection in patients with autoimmune conditions treated with systemic therapies: a population-based study. | 2022a | H | Joint | 61 | 49 | All Cases |  | 2306 | 311563 | 93 | 2213 | 5226 | 306337 | OR | 1.35 | 1.09 | 1.68 |  | No | Y |
| Curtis et al | Characteristics, comorbidities, and outcomes of SARS-cov-2 infection in patients with autoimmune conditions treated with systemic therapies: a population-based study. | 2022b | H | Skin | 55 | 49 | All Cases |  | 421 | 311563 | 8 | 413 | 5226 | 306337 | OR | 1.24 | 0.53 | 1.94 |  | No | Y |
| D’Silva et al | COVID‐19 outcomes in patients with systemic autoimmune rheumatic diseases compared to the general population: a US multicenter, comparative cohort study | 2022 | H | General | 58 | 58 | All Cases | 30 day | 2379 | 2379 | 93 | 2286 | 79 | 2300 | RR | 1.18 | 0.88 | 1.58 |  | Yes | Y |
| Ferri | Prevalance and Death Rate of COVID-19 in Autoimmune Systemic Diseases in the First Three Pandemic Waves. Relationship with Disease Subgroups and Ongoing Therapies | 2022 | H | General | 59 |  | All Cases |  | 316 | 4051401 | 12 | 304 | 12376 | 4039025 | OR | 1.25 | 0.7 | 2.22 | 0.45 | No | N |
| Figueroa-Parra et al | Risk of severe COVID-19 outcomes associated with rheumatoid arthritis and phenotypic subgroups: a retrospective, comparative, multicentre cohort study | 2022 | H | Joint | 62 | 61 | All Cases |  | 582 | 2875 | 26 | 556 | 59 | 2816 | HR | 1.53 | 0.94 | 2.48 |  | No | N |
| Gisondi et al | Incidence rates of hospitalization and death from COVID-19 in patients with psoriasis receiving biological treatment: A Northern Italy experience | 2022 | H | Skin | 53.4 | 52.3 | All Cases |  | 6481 | 144909 |  |  |  |  | SIR | 0.42 | 0.07 | 1.38 | 0.19 | No | N |
| Hadi et al | Incidence, outcomes, and impact of COVID-19 on inflammatory bowel disease: propensity matched research network analysis | 2022a | H | Gut | 49.72 | 49.79 | All Cases | 30 day | 4310 | 4310 | 90 | 4220 | 95 | 4215 | RR | 0.947 | 0.712 | 1.26 |  | Yes | Y |
| Hadi et al | Incidence, outcomes, and impact of COVID-19 on inflammatory bowel disease: propensity matched research network analysis | 2022b | H | Gut |  |  | All Cases | 30 day | 2082 | 4310 | 41 | 2041 | 95 | 4215 | RR | 0.759 | 0.508 | 1.134 |  | Yes | Y |
| Hadi et al | Incidence, outcomes, and impact of COVID-19 on inflammatory bowel disease: propensity matched research network analysis | 2022c | H | Gut |  |  | All Cases | 30 day | 2190 | 4310 | 46 | 2144 | 95 | 4215 | RR | 1.314 | 0.85 | 2.032 |  | Yes | Y |
| Kjeldsen et al | [Outcome of COVID-19 in hospitalized patients with chronic inflammatory diseases. A population based national register study in Denmark](https://www.sciencedirect.com/science/article/pii/S0896841121000408?casa_token=SM5d56EExREAAAAA:JkzzXCEEichEC8QECJ17BP5-6SEdj00HSlnprtny7CWCSIRVgFihxch1Dr_UnY8b7bd0-FJSbg) | 2022 | H | General | 74 | 69 | In-hospital | 30 day | 132 | 2811 | 28 | 104 | 537 | 2274 | OR | 0.68 | 0.41 | 1.13 |  | No | Y |
| Kodvanj et al | Inflammatory Bowel Disease Is Associated with an Increased Risk for Covid-19-Related Hospitalization, but Not with Mortality: Croatian Nationwide Cohort Study | 2022 | H | Gut |  |  | All Cases |  | 3067 | 433609 |  |  |  |  | RR | 0.85 | 0.6 | 1.19 |  | Yes | Y |
| Kridin et al | Nineteen months into the pandemic, what have we learned about COVID-19-related outcomes in patients with psoriasis? | 2022 | H | Skin | 50 | 50 | All Cases |  | 144304 | 144304 | 232 | 144072 | 234 | 144070 | HR | 0.88 | 0.73 | 1.05 | 0.162 | Yes | Y |
| MacKenna et al | [Risk of severe COVID-19 outcomes associated with immune-mediated inflammatory diseases and immune-modifying therapies: a nationwide cohort stud](https://www.thelancet.com/journals/lanrhe/article/PIIS2665-9913(22)00098-4/fulltext) | 2022a | H | General |  |  | All Cases |  | 1163438 | 16508627 | 4824 | 1158614 | 40453 | 16468174 | HR | 1.15 | 1.11 | 1.18 |  | No | Y |
| MacKenna et al | [Risk of severe COVID-19 outcomes associated with immune-mediated inflammatory diseases and immune-modifying therapies: a nationwide cohort stud](https://www.thelancet.com/journals/lanrhe/article/PIIS2665-9913(22)00098-4/fulltext) | 2022d | H | Gut |  |  | All Cases |  | 199037 | 16508627 | 721 | 198316 | 40453 | 16468174 | HR | 1.07 | 0.99 | 1.15 |  | No | Y |
| MacKenna et al | [Risk of severe COVID-19 outcomes associated with immune-mediated inflammatory diseases and immune-modifying therapies: a nationwide cohort stud](https://www.thelancet.com/journals/lanrhe/article/PIIS2665-9913(22)00098-4/fulltext) | 2022b | H | Joint |  |  | All Cases |  | 272452 | 16508627 | 1856 | 270596 | 40453 | 16468174 | HR | 1.3 | 1.24 | 1.37 |  | No | Y |
| MacKenna et al | [Risk of severe COVID-19 outcomes associated with immune-mediated inflammatory diseases and immune-modifying therapies: a nationwide cohort stud](https://www.thelancet.com/journals/lanrhe/article/PIIS2665-9913(22)00098-4/fulltext) | 2022c | H | Skin |  |  | All Cases |  | 769816 | 16508627 | 2608 | 767208 | 40453 | 16468174 | HR | 1.07 | 1.02 | 1.11 |  | No | Y |
| Mahdavi et al | Factors associated with COVID-19 and its outcome in patients with rheumatoid arthritis | 2022 | L | Joint | 52.3 | 48.4 | All Cases | 30 day | 128 | 92 | 11 | 117 | 5 | 87 |  |  |  |  |  | No | N |
| Marozoff et al | [Severe COVID-19 outcomes among patients with autoimmune rheumatic diseases or transplantation: a population-based matched cohort study](https://bmjopen.bmj.com/content/12/8/e062404.abstract) | 2022 | H | Joint | 54.3 | 53.6 | All Cases |  | 6279 | 31130 | 232 | 6047 | 841 | 30289 | OR | 1.24 | 1.05 | 1.47 |  | Yes | Y |
| Moreno-Torres et al | Systemic Autoimmune Diseases in Patients Hospitalized with COVID-19 in Spain: A Nation-Wide Registry Study | 2022 | H | General | 67.5 | 66.5 | In-hospital |  | 892 | 116802 | 174 | 718 | 18864 | 97938 | OR | 0.93 | 0.78 | 1.11 |  | No | N |
| Pablos et al | Clinical outcomes of hospitalised patients with COVID-19 and chronic inflammatory and autoimmune rheumatic diseases: a multicentric matched cohort study | 2022 | H | Joint | 63 | 63 | In-hospital |  | 228 | 228 | 41 | 187 | 30 | 198 |  |  |  |  |  | Yes | N |
| Pakhchanian et al | [COVID-19 outcomes in patients with dermatomyositis: a registry-based cohort analysis](https://www.sciencedirect.com/science/article/pii/S0049017222000853) | 2022 | H | Skin | 58.45 | 58.95 | All Cases |  | 5574 | 5574 | 112 | 5462 | 147 | 30983 | RR | 0.76 | 0.6 | 0.97 |  | Yes | Y |
| Qi et al | [Clinical outcomes of COVID-19 patients with rheumatic diseases: a retrospective cohort study and synthesis analysis in Wuhan, China](https://link.springer.com/article/10.1007/s10067-022-06086-2) | 2022 | H | General | 62.04 | 62.09 | In-hospital |  | 90 | 347 | 1 | 89 | 12 | 335 | RR | 0.31 | 0.04 | 2.44 |  | Yes | Y |
| Raiker et al | 254 COVID‐19 related outcomes in psoriasis and psoriasis arthritis patients. | 2022 | H | Skin |  |  | All Cases | 45 day | 2288 | 2288 |  |  |  |  | RR | 0.82 | 0.57 | 1.19 |  | Yes | Y |
| Raiker et al | Outcomes of COVID-19 in patients with rheumatoid arthritis: A multicenter research network study in the United States | 2022 | H | Joint | 61.1 | 61.4 | In-hospital |  | 9730 | 9730 | 357 | 9373 | 328 | 9402 | RR | 1.09 | 0.94 | 1.26 | 0.2593 | Yes | Y |
| Rorat et al | The course of COVID-19 in patients with systemic autoimmune rheumatic diseases | 2022 | H | General | 69 | 64 | In-hospital | 28 day | 185 | 8035 | 42 | 143 | 1155 | 6880 |  |  |  |  |  | No | N |
| Rutter et al | COVID-19 infection, admission and death among people with rare autoimmune rheumatic disease in England: results from the RECORDER project | 2022 | H | General | 61.7 |  | All Cases | 28 days | 1874 | 261348 | 574 | 1300 | 36658 | 224690 | RR | 2.7 | 2.56 | 2.84 |  | No | N |
| Shin et al | Autoimmune inflammatory rheumatic diseases and COVID-19 out- comes in South Korea: a nationwide cohort study. | 2022a | H | Joint |  |  | All Cases |  | 365 | 891 | 24 | 341 | 40 | 851 | OR | 1.69 | 1.01 | 2.84 | 0.046 | Yes | Y |
| \| Shin et al \| \| --- \| | Autoimmune inflammatory rheumatic diseases and COVID-19 out- comes in South Korea: a nationwide cohort study. | 2022b | H | Joint |  |  | All Cases |  | 84 | 188 | 7 | 77 | 11 | 177 | OR | 1.87 | 0.71 | 4.85 | 0.031 | Yes | Y |
| Shin et al | Autoimmune inflammatory rheumatic diseases and COVID-19 out- comes in South Korea: a nationwide cohort study. | 2022c | H | Joint |  |  | All Cases |  | 327 | 796 | 20 | 307 | 32 | 764 | OR | 1.81 | 1.02 | 3.18 | 0.04 | Yes | Y |
| Thompson et al | Investigation Into the Effect of COVID-19 Infection on Length of Hospital Stay and Mortality in Patients With Rheumatoid Arthritis | 2022 | H | Joint | 58 | 58 | In-hospital |  | 159 | 14021 |  |  |  |  | OR | 1.65 | 1.07 | 2.53 | 0.02 | No | Y |
| Topless et al | Gout, Rheumatoid Arthritis, and the Risk of Death Related to Coronavirus Disease 2019: An Analysis of the UK Biobank | 2022a | H | General |  |  | All Cases |  | 117 | 1942 |  |  |  |  | OR | 1.71 | 1.23 | 2.38 | 1.4 × 10−3 | Yes | Y |
| \| Topless et al \| \| --- \| | Gout, Rheumatoid Arthritis, and the Risk of Death Related to Coronavirus Disease 2019: An Analysis of the UK Biobank | 2022b | H | Joint |  |  | All Cases |  | 61 | 13063 |  |  |  |  | OR | 3.23 | 2.07 | 5.04 | 2.2 × 10−7 | Yes | Y |
| Ungaro et al | Autoimmune and chronic inflammatory disease patients with COVID‐19. | 2022 | H | General | 63 | 62 | All Cases |  | 159 | 6633 | 36 | 123 | 1445 | 5188 |  |  |  |  |  | No | N |
| Zanetti et al | Increased COVID-19 mortality in patients with rheumatic diseases: results from the CONTROL-19 study by the Italian Society for Rheumatology | 2022 | H | Joint | 58.4 |  | All Cases |  | 668 | 1395075 | 62 | 55803 |  |  | OR | 3.1 | 2.29 | 4.12 |  | No | N |

1. HIV data extraction form

| **Authors** | **Title** | **Year** | **Income** | **IS Type** | **Exposed age** | **Unexposed age** | **Case** | **Time to mortality** | **Exposed (n)** | **Unexposed (n)** | **Exposed death** | **Exposed survivors** | **Unexposed death** | **Unexposed survivors** | **Effect Measure** | **Value** | **LB** | **UB** | **p** | **Matched** | **Adjusted criteria met** |
| --- | --- | --- | --- | --- | --- | --- | --- | --- | --- | --- | --- | --- | --- | --- | --- | --- | --- | --- | --- | --- | --- |
| Bennett et al | Multicenter Study of Outcomes Among Persons With HIV Who Presented to US Emergency Departments With Suspected SARS-CoV-2 | 2021 | H | HIV | 57 | 59 | In-hospital |  | 201 | 13236 | 28 | 173 | 1995 | 11241 |  |  |  |  |  | No | N |
| Bhaskaran et al | HIV infection and COVID-19 death: a population-based cohort analysis of UK primary care data and linked national death registrations within the OpenSAFELY platform | 2021 | H | HIV | 48 | 49 | All Cases |  | 27480 | 17255425 | 25 | 27455 | 14857 | 17240568 | HR | 2.3 | 1.55 | 3.41 | <0.0001 | No | Y |
| Boulle | Risk factors for COVID-19 death in a population HIV cohort study from the Western Cape Province, South Africa | 2021 | L | HIV |  |  | All Cases |  | 3978 | 18330 | 115 | 3863 | 510 | 17820 | HR | 2.14 | 1.7 | 2.7 |  | No | Y |
| Cabello et al | COVID-19 in people living with HIV: A multicenter case-series study | 2021 | H | HIV | 46 |  | In-hospital |  | 63 | 18790 | 2 | 61 | 1315 | 17475 |  |  |  |  |  | No | N |
| Durstenfield et al | Impact of HIV Infection on COVID-19 Outcomes Among Hospitalized Adults in the U.S. | 2021 | H | HIV | 56 | 62.3 | In-hospital |  | 220 | 21308 | 36 | 184 | 3290 | 18018 | OR | 1.13 | 0.77 | 1.6 | 0.54 | No | N |
| Flannery et al | A comparison of COVID-19 inpatients by HIV status. | 2021 | H | HIV | 58.3 | 64.32 | In-hospital |  | 99 | 10202 | 25 | 74 | 2703 | 7499 |  |  |  |  |  | No | N |
| Geretti et al | Outcomes of COVID-19 related hospitalization among people with HIV in the ISARIC WHO Clinical Characterization Protocol (UK): a prospective observational study | 2021 | H | HIV | 56 | 74 | In-hospital | 28 days | 122 | 47470 | 30 | 92 | 13969 | 33501 | HR | 1.5 | 1.02 | 2.22 | 0.04 | No | Y |
| Hadi et al | Characteristics and outcomes of COVID-19 in patients with HIV: a multicentre research network study | 2020 | H | HIV | 48.18 | 47.75 | All Cases | 30 days | 404 | 404 | 20 | 384 | 15 | 389 | RR | 1.33 | 0.69 | 2.57 |  | Yes | Y |
| Hedberg et al | Incidence and severity of COVID-19 in adults with and without diagnosis. | 2022 | H | HIV | 50 | 50 | All Cases | 30 days | 364 | 3587 | 6 | 358 | 44 | 3543 |  |  |  |  |  | No | N |
| Kwapong et al | Effect of status on ICU admission and mortality among hospitalized coronavirus disease 2019 (COVID-19) patients | 2022 | H | HIV | 52.7 | 52.7 | In-hospital |  | 151 | 185 |  |  |  |  | OR | 0.97 | 0.32 | 2.88 |  | Yes | Y |
| Lee et al | Comparative outcomes in hospital admissions with COVID-19 in people living with and people living without : a retrospective study | 2020 | H | HIV | 57 | 56 | In-hospital | 28 days | 68 | 181 | 13 | 55 | 35 | 146 | HR |  |  |  |  | No | Y |
| Looha et al | The impact of on the risk of COVID-19 death among hospitalized patients | 2022 | L | HIV | 58 | 50 | In-hospital |  | 127 | 325925 | 18 | 109 | 21595 | 304330 | OR | 1.49 | 0.99 | 2.25 |  | No | Y |
| Miyashita et al | Prognosis of coronavirus disease 2019 (COVID-19) in patients with infection in New York City | 2021 | H | HIV |  |  | In-hospital |  | 161 | 8751 | 23 | 138 | 1235 | 7516 | RR | 1.01 | 0.69 | 1.48 |  | No | N |
| Moreno-Torres et al | Predictors of in-hospital mortality in HIV-infected patients with COVID-19 | 2022 | H | HIV | 53.2 | 66.5 | In-hospital |  | 234 | 117460 | 22 | 212 | 18836 | 98624 | OR | 1.08 | 0.68 | 1.7 | 0.704 | No | N |
| Olalla-Sierra et al | Coronavirus disease 2019 hospitalization outcomes in persons with and without in Spain | 2022 | H | HIV | 57.9 | 67.4 | In-hospital |  | 98 | 16465 | 12 | 86 | 3507 | 12958 |  |  |  |  |  | No | N |
| Park et al | COVID-19 in the largest US cohort. AIDS | 2020 | H | HIV |  |  | All Cases |  | 253 | 504 | 24 | 165 | 56 | 324 | HR | 1.08 | 0.66 | 1.75 |  | No | N |
| Parker et al | Clinical features and outcomes of COVID-19 admissions in a population with a high prevalence of and tuberculosis: a multicentre cohort study | 2022 | L | HIV | 46 | 54 | In-hospital |  | 270 | 1164 | 71 | 199 | 296 | 868 | OR | 1.56 | 1.11 | 2.2 | 0.011 | No | Y |
| Patel et al | Clinical outcomes and inflammatory markers by HIV serostatus and viral suppression in a large cohort of patients hospi- talized with COVID-19 | 2021 | H | HIV | 63 | 65 | In-hospital |  | 100 | 4513 | 22 | 78 | 1104 | 3409 | HR | 1.2 | 0.78 | 1.83 | 0.41 | No | Y |
| Rasmussen et al | Outcomes following SARS-CoV-2 infection among individuals living with and without HIV; a Danish nationwide cohort study | 2022 | H | HIV | 51.7 | 51.7 | All Cases | 90 days | 5276 | 42208 | 4 | 2037 | 41 | 19721 | IRR | 0.7 | 0.3 | 2 |  | Yes | Y |
| Rosenthal et al | Factors associated with SARS-CoV-2-related hospital outcomes among and between persons living with and without diagnosed HIV infection in New York State | 2022 | H | HIV |  |  | In-hospital |  | 853 | 1621 | 200 | 653 | 414 | 1207 | RR | 0.94 | 0.82 | 1.08 |  | Yes | N |
| Sigel et al | Covid-19 and people with HIV New York City | 2020 | H | HIV | 61 | 60 | In-hospital |  | 88 | 405 | 18 | 70 | 81 | 324 | SHR | 1.13 | 0.62 | 2.08 |  | Yes | N |
| Spence et al | COVID-19 Outcomes in a US Cohort of Persons Living with HIV (PLWH) | 2022 | H | HIV | 51.5 | 51.2 | In-hospital |  | 281 | 1124 | 18 | 263 | 33 | 1091 |  |  |  |  |  | Yes | N |
| Sun et al | COVID-19 Disease Severity among People with HIV Infection or Solid Organ Transplant in the United States: A Nationally-representative, Multicenter, Observational Cohort Study | 2021 | H | HIV | 50 | 47 | All Cases | 45 days | 8270 | 1426984 | 196 | 8074 | 23831 | 1403153 | OR | 1.2 | 1.19 | 1.2 | <0.01 | No | Y |
| Tang et al | People with have a higher risk of COVID-19 diagnosis but similar outcomes to the general population | 2022 | H | HIV | 45 | 50 | In-hospital |  | 88 | 399 | 1 | 87 | 7 | 392 | IRR | 0.729 | 0.078 | 6.813 |  | No | Y |
| Tesoriero et al | COVID-19 Outcomes Among Persons Living With or Without Diagnosed HIV Infection in New York State | 2021 | H | HIV | 54 |  | All Cases |  | 2988 | 375260 | 207 | 2781 | 14522 | 360738 | RR | 1.3 | 1.13 | 1.48 |  | No | N |
| Yang et al | Associations between HIV infection and clinical spectrum of COVID-19: a population level analysis based on US National COVID Cohort Collaborative (N3C) data | 2021 | H | HIV | 49 | 47 | All Cases |  | 13170 | 1423452 | 445 | 12725 | 25685 | 1397767 | OR | 1.29 | 1.16 | 1.44 |  | No | Y |
| Yendewa et al | Clinical Features and Outcomes of Coronavirus Disease 2019 Among People With Human Immunodeficiency Virus in the United States: A Multicenter Study From a Large Global Health Research Network (TriNetX) | 2021 | H | HIV | 48.34 | 49.12 | All Cases | 30 days | 1635 | 1609 | 46 | 1589 | 61 | 46 | OR | 0.74 | 0.5 | 1.08 | 0.145 | Yes | Y |
