# Supplementary material for: Disparities in COVID-19 mortality amongst the immunosuppressed: A systematic review and meta-analysis for enhanced disease surveillance
Source: J Infect. 2024 Mar;88(3):None. doi: 10.1016/j.jinf.2024.01.009 (PMC10943183; doi:10.1016/j.jinf.2024.01.009)
Supplement: Supplementary file 6 — Supplementary material [file mmc6.docx]

**Appendix 6:** Characteristics of all studies included in comparative meta-analysis

|  |  |  |  |  |  |  |  |  |  |  |
| --- | --- | --- | --- | --- | --- | --- | --- | --- | --- | --- |
| **Subgroup** | **Authors** | **Title** | **Date** | **Design** | **Country** | **Immunosuppressed (n)** | **Control (n)** | **Average Immunosuppressed Age** | **Average Control Age** | **Effect Size (95% CI)** |
| Malignancy | Alpert et al | [Clinical course of cancer patients with COVID-19: a retrospective cohort study](https://academic.oup.com/jncics/article-abstract/5/1/pkaa085/5951182) | 2021 | Retrospective cohort study | USA | 420 | 840 | NA | NA | 1.12 (0.86-1.45) |
| Rheumatological Conditions | Attauabi et al | Outcomes and long-term effects of COVID-19 in patients with inflammatory bowel diseases—A Danish prospective population-based cohort study with individual-level data | 2022a | Prospective cohort study | Denmark | 319 | 230087 | 48 |  | 3.9 (2.89-6.65) |
| Rheumatological Conditions | Attauabi et al | “ | 2022b | Prospective cohort study | Denmark | 197 | 230087* | 44 |  | 0.97 (0.24-3.86) |
| Immunosuppressive Agents | Belleudi et al | [Direct and indirect impact of COVID-19 for patients with immune-mediated inflammatory diseases: a retrospective cohort study](https://www.mdpi.com/2077-0383/10/11/2388) | 2021 | Retrospective cohort study | Italy | 9176 | 4702567 | 56 | 51 | 2 (1.11-3.63) |
| Rheumatological Conditions | Belleudi et al | [Direct and indirect impact of COVID-19 for patients with immune-mediated inflammatory diseases: a retrospective cohort study](https://www.mdpi.com/2077-0383/10/11/2388) | 2022a | Retrospective cohort study | Italy | 65230 | 4702567 | 59 | 51 | 1.22 (0.95-1.56) |
| Rheumatological Conditions | Belleudi et al | “ | 2022b | Retrospective cohort study | Italy | 20299* | 4702567* | 64 | 51 | 1.25 (0.84-1.85) |
| Rheumatological Conditions | Belleudi et al | “ | 2022c | Retrospective cohort study | Italy | 22525* | 4702567* | 53 | 51 | 0.95 (0.56-1.59) |
| Rheumatological Conditions | Belleudi et al | “ | 2022d | Retrospective cohort study | Italy | 22406* | 4702567* | 60 | 51 | 1.4 (0.94-2.10) |
| HIV | Bennett et al | Multicenter Study of Outcomes Among Persons With HIV Who Presented to US Emergency Departments With Suspected SARS-CoV-2 | 2021 | Retrospective cohort study | USA | 201 | 13236 | 57 | 59 | 0.91 (0.61-1.36)** |
| Rheumatological Conditions | Bertoglio et al | Poor Prognosis of COVID-19 Acute Respiratory Distress Syndrome in Lupus Erythematosus: Nationwide Cross-Sectional Population Study Of 252 119 Patients | 2022 | Cross-Sectional study | Brazil | 319 | 251800 | 45.5 | 58.7 | 1.738 (1.56-1.91) |
| HIV | Bhaskaran et al | HIV infection and COVID-19 death: a population-based cohort analysis of UK primary care data and linked national death registrations within the OpenSAFELY platform | 2021 | Retrospective cohort study | UK | 27480 | 17255425 | 48 | 49 | 2.3 (1.55-3.41) |
| HIV | Boulle | Risk factors for COVID-19 death in a population cohort study from the Western Cape Province, South Africa | 2021 | Retrospective cohort study | South Africa | 3978 | 18330 | NA | NA | 2.14 (1.7-2.7) |
| Malignancy | Brar et al | COVID-19 severity and outcomes in patients with cancer: A matched cohort study | 2020 | Retrospective cohort study | USA | 117 | 468 | 72.5 | 71.2 | 0.98 (0.58-1.67) |
| Rheumatological Conditions | Bruera et al | Patients with systemic lupus erythematosus have an increased risk of mortality, mechanical ventilation, and hospitalization from COVID-19 | 2022 | Retrospective cohort study | USA | 687 | 6870 | NA | NA | 1.39 (0.79-2.44) |
| HIV | Cabello et al | COVID-19 in people living with HIV: A multicenter case-series study | 2021 | Retrospective cohort study | Spain | 63 | 18790 | 46 | NA | 0.43 (0.11-1.78)** |
| Transplantation | Caillard et al | Is COVID‐19 infection more severe in kidney transplant recipients? | 2021 | Retrospective cohort study | France | 273 | 273 | 62 | 63 | 1.55 (1.02-2.35) |
| Transplantation | Chavarot et al | COVID‐19 severity in kidney transplant recipients is similar to nontransplant patients with similar comorbidities | 2022 | Retrospective cohort study | France | 83 | 83 | 67.2 | 65.1 | 1.36 (0.67-2.83) |
| Immunosuppressive Agents | Chavez-MacGregor et al | [Evaluation of COVID-19 mortality and adverse outcomes in US patients with or without cancer](https://jamanetwork.com/journals/jamaoncology/article-abstract/2785677) | 2021 | Retrospective cohort study | USA | 4296 | 493020 | 66 | 48 | 1.74 (1.54-1.96) |
| Rheumatological Conditions | Chiriboga et al | [Risk of COVID-19 Infection and Hospitalization in Patients With Inflammatory Rheumatic Disease Compared With the General Population](https://journals.lww.com/jclinrheum/Fulltext/2022/03000/Risk_of_COVID_19_Infection_and_Hospitalization_in.63.aspx) | 2022 | Retrospective cohort study | USA | 470 | 26434 | 61.8 | 40.2 | 0.25 (0.06-1.00) |
| Malignancy | Costa et al | “ | 2021b | Retrospective cohort study | Brazil | 944* | 315410* | NA | NA | 2.85 (2.41-3.38) |
| Malignancy | Costa et al | “ | 2021c | Retrospective cohort study | Brazil | 6461* | 315410* | NA | NA | 1.83 (1.72-1.95) |
| Malignancy | Costa et al | [Higher severity and risk of in‐hospital mortality for COVID‐19 patients with cancer during the year 2020 in Brazil: A countrywide analysis of secondary data](https://acsjournals.onlinelibrary.wiley.com/doi/abs/10.1002/cncr.33832) | 2021a | Retrospective cohort study | Brazil | 7406 | 315410 | 67 | 62 | 1.94 (1.83-2.06) |
| Rheumatological Conditions | Curtis et al | Characteristics, comorbidities, and outcomes of SARS-cov-2 infection in patients with autoimmune conditions treated with systemic therapies: a population-based study. | 2022a | Retrospective cohort study | USA | 2306 | 311563* | 61 | 49 | 1.35 (1.09-1.68) |
| Rheumatological Conditions | Curtis et al | “ | 2022b | Retrospective cohort study | USA | 421 | 311563* | 55 | 49 | 1.24 (0.53-1.94) |
| Rheumatological Conditions | Curtis et al | “ | 2022c | Retrospective cohort study | USA | 811 | 311563 | 52 | 49 | 1.11 (0.68-1.82) |
| Rheumatological Conditions | D’Silva et al | COVID‐19 outcomes in patients with systemic autoimmune rheumatic diseases compared to the general population: a US multicenter, comparative cohort study | 2022 | Retrospective cohort study | USA | 2379 | 2379 | 58 | 58 | 1.18 (0.88-1.58) |
| Malignancy | Dai et al | Patients with Cancer Appear More Vulnerable to SARS-CoV-2: A Multicenter Study during the COVID-19 Outbreak | 2020 | Retrospective cohort study | China | 105 | 536 | 64 | 63.5 | 2.17 (0.81-5.15) |
| Malignancy | de Azambuja et al | Impact of solid cancer on in‐hospital mortality overall and among different subgroups of patients with COVID‐19: a nationwide, population‐based analysis | 2020 | Retrospective cohort study | Belgium | 892 | 9594 | 75 | 70 | 1.34(1.13-1.58) |
| HIV | Durstenfeld et al | Impact of HIV Infection on COVID-19 Outcomes Among Hospitalized Adults in the U.S. | 2021 | Retrospective cohort study | USA | 220 | 21308 | 56 | 62.3 | 1.13 (0.77-1.6) |
| Malignancy | Fernández-Cruz et al | [Higher mortality of hospitalized haematologic patients with COVID-19 compared to non-haematologic is driven by thrombotic complications and development](https://www.sciencedirect.com/science/article/pii/S259017022200005X) | [2022](https://www.cell.com/cancer-cell/pdf/S1535-6108(20)30481-5.pdf) | Retrospective cohort study | Spain | 71 | 142 | 70.7 | 69.6 | 2.74 (1.44-5.20)** |
| Rheumatological Conditions | Ferri | Prevalance and Death Rate of COVID-19 in Autoimmune Systemic Diseases in the First Three Pandemic Waves. Relationship with Disease Subgroups and Ongoing Therapies | 2022 | Prospective cohort study | Italy | 316 | 4051401 | 59 |  | 1.25 (0.7-2.22)** |
| Rheumatological Conditions | Figueroa-Parra et al | Risk of severe COVID-19 outcomes associated with rheumatoid arthritis and phenotypic subgroups: a retrospective, comparative, multicentre cohort study | 2022 | Retrospective cohort study | USA | 582 | 2875 | 62 | 61 | 1.53 (0.94-2.48) |
| Transplantation | Fisher et al | Outcomes of COVID-19 in hospitalized solid organ transplant recipients compared to a matched cohort of non-transplant pa- tients at a national healthcare system in the United States | 2021 | Retrospective cohort study | USA | 128 | 3907 | 60 | 60 | 1.93 (1.18-3.15) |
| HIV | Flannery et al | A comparison of COVID-19 inpatients by HIV status. | 2021 | Retrospective cohort study | USA | 99 | 10202 | 58.3 | 64.32 | 0.94 (0.59-1.48)** |
| Malignancy | Fu et al | [COVID‐19 outcomes in hospitalized patients with active cancer: Experiences from a major New York City health care system](https://acsjournals.onlinelibrary.wiley.com/doi/abs/10.1002/cncr.33657) | 2021a | Retrospective cohort study | USA | 233 | 3953 | 71.2 | 62.2 | 1.89 (1.33-2.67) |
| Malignancy | Fu et al | “ | 2021b | Retrospective cohort study | USA | 69* | 3953* | NA | NA | 4.39 (2.72-7.09)** |
| Malignancy | \| Fu et al \| \| --- \| | “ | 2021c | Retrospective cohort study | USA | 164* | 3953* | NA | NA | 19.35 (13.37-27.99)** |
| Immunosuppressive Agents | Garneau et al | Clinical outcomes of patients previously treated with B-cell depletion therapy hospitalized with COVID-19: results from the Johns Hopkins Crown Registry | 2022 | Retrospective cohort study | USA | 50 | 212 | 57.4 | 57.4 | 1.44 (0.38-5.52)** |
| HIV | Geretti et al | Outcomes of COVID-19 related hospitalization among people with HIV in the ISARIC WHO Clinical Characterization Protocol (UK): a prospective observational study | 2021 | Prospective cohort study | UK | 122 | 47470 | 56 | 74 | 1.5 (1.02-2.22) |
| Rheumatological Conditions | Gisondi et al | Incidence rates of hospitalization and death from COVID-19 in patients with psoriasis receiving biological treatment: A Northern Italy experience | 2022 | Retrospective cohort study | Italy | 6481 | 144909 | 53.4 | 52.3 | 0.42 (0.07-1.38) |
| Malignancy | Hachem et al | Comparing the outcome of COVID-19 in cancer and non-cancer patients: An international multicenter study. | 2020 | Retrospective cohort study | International | 186 | 385 | NA | NA | 2.03 (1.25-3.28)** |
| HIV | Hadi et al | Characteristics and outcomes of COVID-19 in patients with HIV: a multicentre research network study | 2020 | Retrospective cohort study | USA | 404 | 404 | 48.18 | 47.75 | 1.33 (0.69-2.57) |
| Rheumatological Conditions | Hadi et al | Incidence, outcomes, and impact of COVID-19 on inflammatory bowel disease: propensity matched research network analysis | 2022a | Retrospective cohort study | USA | 4310 | 4310 | 49.72 | 49.79 | 0.947 (0.71-1.26) |
| Rheumatological Conditions | Hadi et al | “ | 2022b | Retrospective cohort study | USA | 2082* | 4310* | NA | NA | 0.759 (0.51-1.13) |
| Rheumatological Conditions | Hadi et al | “ | 2022c | Retrospective cohort study | USA | 2190* | 4310* | NA | NA | 1.314 (0.85-2.03) |
| Transplantation | Hadi et al | Outcomes of COVID-19 in solid organ transplant recipients: a propensity-matched analysis of a large research network | 2022a | Retrospective cohort study | USA | 2289 | 2289 | 54.5 | 55.2 | 0.99 (0.73-1.34) |
| Transplantation | Hadi et al | “ | 2022b | Retrospective cohort study | USA | 1511* | 2289* | 52 | 30 | 0.78 (0.56-1.08) |
| Transplantation | Hadi et al | “ | 2022c | Retrospective cohort study | USA | 183* | 2289* | NA | NA | 3.88 (2.2-6.81) |
| HIV | Hedberg et al | Incidence and severity of COVID-19 in adults with and without HIV diagnosis. | 2022 | Retrospective cohort study | Sweden | 364 | 3587 | 50 | 50 | 1.35 (0.57-3.19)** |
| Malignancy | Johannesen et al | [COVID-19 in cancer patients, risk factors for disease and adverse outcome, a population-based study from Norway](https://www.frontiersin.org/articles/10.3389/fonc.2021.652535/full) | 2021a | Retrospective cohort study | Norway | 547 | 7863 | NA | NA | 0.99 (0.68-1.42) |
| Malignancy | Johannesen et al | “ | 2021b | Retrospective cohort study | Norway | 54* | 7863* | NA | NA | 1 (0.28-3.65) |
| Malignancy | Johannesen et al | “ | 2021c | Retrospective cohort study | Norway | 493* | 7863* | NA | NA | 0.99 (0.68-1.44) |
| Rheumatological Conditions | Kjeldsen et al | [Outcome of COVID-19 in hospitalized patients with chronic inflammatory diseases. A population based national register study in Denmark](https://www.sciencedirect.com/science/article/pii/S0896841121000408?casa_token=SM5d56EExREAAAAA:JkzzXCEEichEC8QECJ17BP5-6SEdj00HSlnprtny7CWCSIRVgFihxch1Dr_UnY8b7bd0-FJSbg) | 2022 | Retrospective cohort study | Denmark | 132 | 2811 | 74 | 69 | 0.68 (0.41-1.13) |
| Rheumatological Conditions | Kodvanj et al | Inflammatory Bowel Disease Is Associated with an Increased Risk for Covid-19-Related Hospitalization, but Not with Mortality: Croatian Nationwide Cohort Study | 2022 | Retrospective cohort study | Croatia | 3067 | 433609 | NA | NA | 0.85 (0.6-1.19) |
| Rheumatological Conditions | Kridin et al | Nineteen months into the pandemic, what have we learned about COVID-19-related outcomes in patients with psoriasis? | 2022 | Retrospective cohort study | Israel | 144304 | 144304 | 50 | 50 | 0.88 (0.73-1.05) |
| HIV | Kwapong et al | Effect of HIV status on ICU admission and mortality among hospitalized coronavirus disease 2019 (COVID-19) patients | 2022 | Retrospective cohort study | USA | 151 | 185 | 52.7 | 52.7 | 0.97 (0.32-2.88) |
| HIV | Lee et al | Comparative outcomes in hospital admissions with COVID-19 in people living with HIV and people living without HIV: a retrospective study | 2020 | Retrospective cohort study | UK | 68 | 181 | 57 | 56 | 0.99 (0.49-2.00)** |
| HIV | Looha et al | [The impact of HIV on the risk of COVID-19 death among hospitalized patients](https://content.iospress.com/articles/human-antibodies/hab220011) | 2022 | Retrospective cohort study | Iran | 127 | 325925 | 58 | 50 | 1.49 (0.99-2.25) |
| Malignancy | Lunski et al | Multivariate mortality analyses in COVID‐19: comparing patients with cancer and patients without cancer in Louisiana | 2020a | Retrospective cohort study | USA | 312 | 4833 | NA | NA | 2.03 (1.44-2.87) |
| Malignancy | Lunski et al | “ | 2020b | Retrospective cohort study | USA | 157 | 1460 | NA | NA | 1.36 (0.89-2.08) |
| Rheumatological Conditions | MacKenna et al | [Risk of severe COVID-19 outcomes associated with immune-mediated inflammatory diseases and immune-modifying therapies: a nationwide cohort stud](https://www.thelancet.com/journals/lanrhe/article/PIIS2665-9913(22)00098-4/fulltext)y | 2022a | Retrospective cohort study | UK | 1163438 | 16508627 | NA | NA | 1.15 (1.11-1.18) |
| Rheumatological Conditions | MacKenna et al | [“](https://www.thelancet.com/journals/lanrhe/article/PIIS2665-9913(22)00098-4/fulltext) | 2022b | Retrospective cohort study | UK | 272452* | 16508627* | NA | NA | 1.3 (1.24-1.37) |
| Rheumatological Conditions | MacKenna et al | [“](https://www.thelancet.com/journals/lanrhe/article/PIIS2665-9913(22)00098-4/fulltext) | 2022c | Retrospective cohort study | UK | 769816* | 16508627* | NA | NA | 1.07 (1.02-1.11) |
| Rheumatological Conditions | MacKenna et al | [“](https://www.thelancet.com/journals/lanrhe/article/PIIS2665-9913(22)00098-4/fulltext) | 2022d | Retrospective cohort study | UK | 199037* | 16508627* | NA | NA | 1.07 (0.99-1.15) |
| Rheumatological Conditions | Mahdavi et al | Factors associated with COVID-19 and its outcome in patients with rheumatoid arthritis | 2022 | Prospective cohort study | Iran | 128 | 92 | 52.3 | 48.4 | 1.64 (0.55-4.88)** |
| Malignancy | Mangone et al | Cumulative COVID‐19 incidence, mortality and prognosis in cancer survivors: a population‐based study in Reggio Emilia, Northern Italy | 2021 | Prospective cohort study | Italy | 447 | 4094 | NA | NA | 1.54 (1.18-2) |
| Transplantation | Mansoor et al | Clinical Characteristics, Hospitalization and Mortality Rates of COVID-19 Among Liver Transplant Patients in the United States: A Multi-Center Research Network Study | 2020 | Retrospective cohort study | USA | 125 | 125 | 57.03 | 59.83 | 1 (0.43-2.32) |
| Rheumatological Conditions | Marozoff et al | [Severe COVID-19 outcomes among patients with autoimmune rheumatic diseases or transplantation: a population-based matched cohort study](https://bmjopen.bmj.com/content/12/8/e062404.abstract) | 2022 | Retrospective cohort study | Canada | 6279 | 31130 | 54.3 | 53.6 | 1.24 (1.05-1.47) |
| Transplantation | \| Marozoff et al \| \| --- \| | [Severe COVID-19 outcomes among patients with autoimmune rheumatic diseases or transplantation: a population-based matched cohort study](https://bmjopen.bmj.com/content/12/8/e062404.abstract) | 2022 | Retrospective cohort study | Canada | 222 | 1106 | 54.3 | 54 | 5.48 (2.82-10.63) |
| Malignancy | Martinez-Lopez et al | Multiple myeloma and SARS-CoV-2 infection: clinical characteristics and prognostic factors of inpatient mortality | 2020 | Retrospective cohort study | Spain | 167 | 167 | 71 | 71 | 1.71 (1.06-2.78)** |
| HIV | Miyashita et al | Prognosis of coronavirus disease 2019 (COVID-19) in patients with HIV infection in New York City | 2021 | Retrospective cohort study | USA | 161 | 8751 | NA | NA | 1.01 (0.69-1.48) |
| Malignancy | Miyashita et al | Do patients with cancer have a poorer prognosis of COVID-19? An experience in New York City. | 2020 | Retrospective cohort study | Italy | 334 | 5354 | NA | NA | 1.15 (0.84-1.57) |
| Transplantation | Molnar et al | Outcomes of critically ill solid organ transplant patients with COVID‐19 in the United States | 2020 | Prospective cohort study | USA | 98 | 288 | 58 | 61 | 0.92 (0.7-1.22) |
| HIV | Moreno-Torres et al | Predictors of in-hospital mortality in HIV-infected patients with COVID-19 | 2022 | Retrospective cohort study | Spain | 234 | 117460 | 53.2 | 66.5 | 1.08 (0.68-1.7) |
| Rheumatological Conditions | Moreno-Torres et al | Systemic Autoimmune Diseases in Patients Hospitalized with COVID-19 in Spain: A Nation-Wide Registry Study | 2022 | Retrospective cohort study | Spain | 892 | 116802 | 67.5 | 66.5 | 0.93 (0.78-1.11) |
| Immunosuppressive Agents | Myint et al | Routine use of immunosuppressants is associated with mortality in hospitalised patients with COVID-19 | 2021 | Prospective cohort study | International | 118 | 1066 | 74 | NA | 1.87 (1.30-2.69) |
| Transplantation | Nair et al | An early experience on the effect of solid organ transplant status on hospitalized COVID-19 patients | 2021 | Retrospective cohort study | USA | 82 | 1625 | 61.8 | 62.7 | 1.34 (1.03-1.74) |
| HIV | Olalla-Sierra et al | Coronavirus disease 2019 hospitalization outcomes in persons with and without HIV in Spain | 2022 | Retrospective cohort study | Spain | 98 | 16465 | 57.9 | 67.4 | 0.51 (0.28-0.94)** |
| Transplantation | [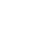](https://onlinelibrary.wiley.com/action/getFTRLinkout?url=https%3A%2F%2Fct.prod.getft.io%2Fd2lsZXksZWxzZXZpZXIsaHR0cHM6Ly93d3cuc2NpZW5jZWRpcmVjdC5jb20vc2NpZW5jZS9hcnRpY2xlL3BpaS9TMDE0MDY3MzYyMDMwNTY2Mz9wZXM9dm9y.yip_QfI1Lv3QmaNPnxJGX39_wBSciulfQT29CMJS3_s&doi=10.1111%2Fapt.16730&doiOfLink=10.1016%2FS0140-6736%2820%2930566-3&linkType=VIEW_FULL_ACCESS&linkLocation=Reference&linkSource=FULL_TEXT)  Osmanodja et al | Undoubtedly, kidney transplant recipients have a higher mortality due to COVID-19 disease compared to the general population | 2021 | Retrospective cohort study | Germany | 114 | 209960 | NA | NA | 3.27 (1.71-6.26) |
| Transplantation | Ozturk et al | Mortality analysis of COVID-19 infection in chronic kidney disease, haemodialysis and renal transplant patients compared with patients without kidney disease: a nationwide analysis from Turkey | 2020 | Retrospective cohort study | Turkey | 81 | 450 | 48 | 51 | 1.897 (0.76-4.73) |
| Rheumatological Conditions | Pablos et al | Clinical outcomes of hospitalised patients with COVID-19 and chronic inflammatory and autoimmune rheumatic diseases: a multicentric matched cohort study | 2022 | Retrospective cohort study | Spain | 228 | 228 | 63 | 63 | 1.44 (0.87-2.41)** |
| Rheumatological Conditions | Pakhchanian et al | [COVID-19 outcomes in patients with dermatomyositis: a registry-based cohort analysis](https://www.sciencedirect.com/science/article/pii/S0049017222000853) | 2022 | Retrospective cohort study | USA | 5574 | 5574 | 58.45 | 58.95 | 0.76 (0.6-0.97) |
| HIV | Park et al | COVID-19 in the largest US HIV cohort | 2020 | Retrospective cohort study | USA | 253 | 504 | NA | NA | 1.08 (0.66-1.75) |
| HIV | Parker et al | Clinical features and outcomes of COVID-19 admissions in a population with a high prevalence of HIV and tuberculosis: a multicentre cohort study | 2022 | Retrospective cohort study | South Africa | 270 | 1164 | 46 | 54 | 1.56 (1.11-2.2) |
| Malignancy | Passamonti et al | Clinical characteristics and risk factors associated with COVID‐19 severity in patients with haematological malignancies in Italy: a retrospective, multicentre, cohort study | 2020 | Retrospective cohort study | Italy | 536 | 29282 | 68 |  | 2.04 (1.77-2.34) |
| HIV | Patel et al | Clinical outcomes and inflammatory markers by HIV serostatus and viral suppression in a large cohort of patients hospitalized with COVID-19 | 2021 | Retrospective cohort study | USA | 100 | 4513 | 63 | 65 | 1.2 (0.78-1.83) |
| Malignancy | Péron et al | [Covid-19 presentation and outcomes among cancer patients: A matched case-control study](https://www.mdpi.com/2072-6694/13/21/5283) | 2021 | Retrospective cohort study | France | 108 | 193 | 76 | 77 | 2 (1.20-3.40) |
| Rheumatological Conditions | Qi et al | [Clinical outcomes of COVID-19 patients with rheumatic diseases: a retrospective cohort study and synthesis analysis in Wuhan, China](https://link.springer.com/article/10.1007/s10067-022-06086-2) | 2022 | Retrospective cohort study | China | 90 | 347 | 62.04 | 62.09 | 0.31 (0.04-2.44) |
| Malignancy | Raad et al | [International Multicenter Study Comparing Cancer to Non-Cancer Patients with COVID-19: Impact of Risk Factors and Treatment Modalities on Survivorship](https://www.medrxiv.org/content/10.1101/2022.08.25.22279181.abstract) | 2022 | Retrospective cohort study | International | 1115 | 2851 | 61 | 50 | 1.46 (1.03-2.07) |
| Malignancy | Raez et al | [Mortality and prognostic factors in hospitalized COVID-19 patients with cancer: an analysis from a large healthcare system in the United States](https://www.ncbi.nlm.nih.gov/pmc/articles/PMC9652524/) | 2022 | Retrospective cohort study | USA | 265 | 4605 | 71 | 60 | 1.48 (1.08-2.01) |
| Rheumatological Conditions | Raiker et al | 254 COVID‐19 related outcomes in psoriasis and psoriasis arthritis patients. | 2022 | Retrospective cohort study | USA | 2288 | 2288 | NA | NA | 0.82 (0.57-1.19) |
| Rheumatological Conditions | Raiker et al | Outcomes of COVID-19 in patients with rheumatoid arthritis: A multicenter research network study in the United States | 2022 | Retrospective cohort study | USA | 9730 | 9730 | 61.1 | 61.4 | 1.09 (0.94-1.26) |
| Transplantation | Ranabothu et al | Outcomes of COVID-19 in solid organ transplants | 2020a | Retrospective cohort study | USA | 288 | 30285 | 55.4 | 47.6 | 3.15 (2.17-4.57) |
| Transplantation | Ranabothu et al | “ | 2020b | Retrospective cohort study | USA | 224* | 30285* | 55.4 | 47.6 | 2.61 (1.66-4.10) |
| HIV | Rasmussen et al | Outcomes following SARS-CoV-2 infection among individuals living with and without HIV; a Danish nationwide cohort study | 2022 | Retrospective cohort study | Denmark | 5276 | 42208 | 51.7 | 51.7 | 0.7(0.3-2.0) |
| Rheumatological Conditions | Rorat et al | The course of COVID-19 in patients with systemic autoimmune rheumatic diseases | 2022 | Retrospective cohort study | Poland | 185 | 8035 | 69 | 64 | 1.75 (1.23-2.48) |
| HIV | Rosenthal et al | Factors associated with SARS-CoV-2-related hospital outcomes among and between persons living with and without diagnosed HIV infection in New York State | 2022 | Retrospective cohort study | USA | 853 | 1621 | NA | NA | 0.94 (0.82-1.08) |
| Malignancy | Rugge et al | SARS-CoV-2 infection in the Italian Veneto region: adverse outcomes in patients with cancer. | 2020a | Retrospective cohort study | Italy | 723 | 8552 | NA | NA | 3.64 (2.9-4.59)** |
| Malignancy | Rugge et al | “ | 2020b | Retrospective cohort study | Italy | 81* | 8552* | NA | NA | 2.39 (1.24-4.58) |
| Rheumatological Conditions | Rutter et al | COVID-19 infection, admission and death among people with rare autoimmune rheumatic disease in England: results from the RECORDER project | 2022 | Retrospective cohort study | UK | 1874 | 261348 | 61.7 |  | 2.7 (2.56-2.84) |
| Transplantation | Sahota et al | Incidence, Risk Factors, and Outcomes of COVID-19 Infection in a Large Cohort of Solid Organ Transplant Recipients | 2022a | Retrospective cohort study | USA | 600 | 312011 | 55.7 | 57.3 | 9.38 (7.47-11.78) |
| Transplantation | Sahota et al | “ | 2022b | Retrospective cohort study | USA | 438* | 312011* | NA | NA | 9.51 (7.28-12.39) |
| Transplantation | Sahota et al | “ | 2022c | Retrospective cohort study | USA | 114* | 312011* | 62 | 57.3 | 5.25 (2.74-10.05) |
| Malignancy | Seyyedsalehi et al | [Hospital and post-discharge mortality in COVID-19 patients with a preexisting cancer diagnosis in Iran](https://www.researchsquare.com/article/rs-2259495/latest) | 2022a | Prospective cohort study | Iran | 1090 | 5517 | 58.16 | 57.87 | 4.4 (3.80-5.20) |
| Malignancy | Seyyedsalehi et al | “ | 2022b | Prospective cohort study | Iran | 423* | 5517* | 51.6 | 57.87 | 5.07 (3.95-6.51) |
| Malignancy | Seyyedsalehi et al | “ | 2022c | Prospective cohort study | Iran | 667* | 5517* | 62.3 | 57.87 | 4.13 (3.41-5.01) |
| Malignancy | Shi et al | [Association of Cancer with Risk and Mortality of COVID-19: Results from the UK Biobank](https://www.medrxiv.org/content/10.1101/2020.07.10.20151076.abstract) | 2020 | Prospective cohort study | UK | 256 | 1306 | 61.36 | 56.11 | 1.04 (0.71-1.51) |
| Rheumatological Conditions | Shin et al | Autoimmune inflammatory rheumatic diseases and COVID-19 out- comes in South Korea: a nationwide cohort study. | 2022a | Prospective cohort study | South Korea | 365 | 891 | NA | NA | 1.69 (1.01-2.84) |
| Rheumatological Conditions | Shin et al | “ | 2022b | Prospective cohort study | South Korea | 84 | 188 | NA | NA | 1.87 (0.71-4.85) |
| Rheumatological Conditions | Shin et al | “ | 2022c | Prospective cohort study | South Korea | 327 | 796 | NA | NA | 1.81 (1.02-3.18) |
| HIV | Sigel et al | Covid-19 and people with HIV infection: outcomes for hospitalized patients in New York City | 2020 | Retrospective cohort study | USA | 88 | 405 | 61 | 60 | 1.13 (0.62-2.08) |
| HIV | Spence et al | [COVID-19 Outcomes in a US Cohort of Persons Living with HIV (PLWH)](https://www.mdpi.com/2571-841X/5/4/41) | 2022 | Retrospective cohort study | USA | 281 | 1124 | 51.5 | 51.2 | 2.26 (1.25-4.08) |
| Immunosuppressive Agents | Suarez-Garcia et al | In-hospital mortality among immunosuppressed patients with COVID-19: Analysis from a national cohort in Spain | 2021a | Retrospective cohort study | Spain | 2111 | 11095* | 71 | 66.5 | 1.6 (1.43-1.79) |
| Immunosuppressive Agents | Suarez-Garcia et al | In-hospital mortality among immunosuppressed patients with COVID-19: Analysis from a national cohort in Spain | 2021b | Retrospective cohort study | Spain | 570* | 11095* | NA | NA | 2.16 (1.8-2.61) |
| Immunosuppressive Agents | Suarez-Garcia et al | In-hospital mortality among immunosuppressed patients with COVID-19: Analysis from a national cohort in Spain | 2021c | Retrospective cohort study | Spain | 183* | 11095* | NA | NA | 1.97 (1.33-2.91) |
| Malignancy | Suarez-Garcia et al | In-hospital mortality among immunosuppressed patients with COVID-19: Analysis from a national cohort in Spain | 2021a | Retrospective cohort study | Spain | 358 | 11095* | 71 | 66.5 | 2.31 (1.76-3.03) |
| Malignancy | Suarez-Garcia et al | “ | 2021b | Retrospective cohort study | Spain | 1081 | 11095* | 73.2 | 66.5 | 1.39 (1.18-1.63) |
| Transplantation | Suarez-Garcia et al | “ | 2021 | Retrospective cohort study | Spain | 166 | 11095 | 63.5 | 66.5 | 3.1 (2.23-4.26) |
| Immunosuppressive Agents | Suarez-Garcia et al | “ | 2021d | Retrospective cohort study | Spain | 394* | 11095* | NA | NA | 2.06 (1.64-2.6) |
| HIV | Sun et al | COVID-19 Disease Severity among People with HIV Infection or Solid Organ Transplant in the United States: A Nationally-representative, Multicenter, Observational Cohort Study | 2021 | Retrospective cohort study | USA | 8270 | 1426984 | 50 | 47 | 1.2 (1.19-1.2) |
| Malignancy | Sun et al | [Rates of COVID-19-related Outcomes in Cancer compared to non-Cancer Patients](https://www.scienceopen.com/document_file/d3a59a27-bf41-44b6-a7c3-fb162d42e6f3/PubMedCentral/d3a59a27-bf41-44b6-a7c3-fb162d42e6f3.pdf) | 2020 | Retrospective cohort study | USA | 67 | 256 | 62 | 50 | 5.67 (1.49-21.59) |
| Transplantation | Sun et al | COVID-19 Disease Severity among People with HIV Infection or Solid Organ Transplant in the United States: A Nationally-representative, Multicenter, Observational Cohort Study | 2021 | Retrospective cohort study | USA | 11392 | 1426984* | 57 | 47 | 3.38 (3.35-3.41) |
| Transplantation | Swan et al | [Hospitalization and survival of solid organ transplant recipients with coronavirus disease 2019: A propensity matched cohort study](https://journals.plos.org/plosone/article?id=10.1371/journal.pone.0278781) | 2022 | Retrospective cohort study | USA | 100 | 500 | 57 | 58 | 1.15 (0.64-2.08) |
| HIV | Tang et al | People with HIV have a higher risk of COVID-19 diagnosis but similar outcomes to the general population | 2022 | Retrospective cohort study | USA | 88 | 399 | 45 | 50 | 0.729 (0.078-6.81) |
| HIV | Tesoriero et al | COVID-19 Outcomes Among Persons Living With or Without Diagnosed HIV Infection in New York State | 2021 | Retrospective cohort study | USA | 2988 | 375260 | 54 | NA | 1.3 (1.13-1.48) |
| Rheumatological Conditions | Thompson et al | Investigation Into the Effect of COVID-19 Infection on Length of Hospital Stay and Mortality in Patients With Rheumatoid Arthritis | 2022 | Retrospective cohort study | USA | 159 | 14021 | 58 | 58 | 1.65 (1.07-2.53) |
| Rheumatological Conditions | Topless et al | Gout, Rheumatoid Arthritis, and the Risk of Death Related to Coronavirus Disease 2019: An Analysis of the UK Biobank | 2022a | Retrospective cohort study | UK | 117 | 1942 | NA | NA | 1.71 (1.23-2.38) |
| Rheumatological Conditions | Topless et al | “ | 2022b | Retrospective cohort study | UK | 61 | 13063 | NA | NA | 3.23 (2.07-5.04) |
| Malignancy | Udovico et al | [High mortality in patients with active malignancy and severe COVID-19: Results from an Austrian multicenter registry during the first period of the COVID-19 …](https://link.springer.com/article/10.1007/s12254-022-00814-9) | 2022 | Retrospective cohort study | Austria | 89 | 5386 | 74 | 76 | 4.59 (2.26-9.3) |
| Rheumatological Conditions | Ungaro et al | Autoimmune and chronic inflammatory disease patients with COVID‐19. | 2022 | Retrospective cohort study | USA | 159 | 6633 | 63 | 62 | 1.05 (0.72-1.53) |
| Transplantation | Webb et al | Liver transplantation does not significantly increase risk of mortality from SARS-CoV-2 infection: International registry data | 2020 | Retrospective cohort study | UK | 151 | 627 | 60 | 73 | 0.63 (0.4-0.98) |
| Malignancy | Westblade et al | [SARS-CoV-2 viral load predicts mortality in patients with and without cancer who are hospitalized with COVID-19](https://www.cell.com/cancer-cell/pdf/S1535-6108(20)30481-5.pdf) | [2020a](https://www.frontiersin.org/articles/10.3389/fonc.2021.652535/full) | Retrospective cohort study | USA | 100 | 2914 | 72 | 65 | 1.29 (0.84-2.0)** |
| Malignancy | Westblade et al | [“](https://www.cell.com/cancer-cell/pdf/S1535-6108(20)30481-5.pdf) | [2020b](https://www.frontiersin.org/articles/10.3389/fonc.2021.652535/full) | Retrospective cohort study | USA | 60* | 2914* | NA | NA | 1.29 (0.74-2.26)** |
| HIV | Yang et al | Associations between HIV infection and clinical spectrum of COVID-19: a population level analysis based on US National COVID Cohort Collaborative (N3C) data | 2021 | Retrospective cohort study | USA | 13170 | 1423452 | 49 | 47 | 1.29 (1.16-1.44) |
| HIV | Yendewa et al | Clinical Features and Outcomes of Coronavirus Disease 2019 Among People With Human Immunodeficiency Virus in the United States: A Multicenter Study From a Large Global Health Research Network (TriNetX) | 2021 | Retrospective cohort study | USA | 1635 | 1609 | 48.34 | 49.12 | 0.74 (0.5-1.08) |
| Malignancy | Yigenoglu et al | The outcome of COVID-19 in patients with hematological malignancy. | 2021 | Retrospective cohort study | Turkey | 740 | 740 | 56 | 56 | 2.21 (1.55-3.15) |
| Immunosuppressive Agents | Yousaf et al | Clinical outcomes of COVID‐19 in patients taking tumor necrosis factor inhibitors or methotrexate: a multicenter research network study. | 2021 | Retrospective cohort study | International | 213 | 213 | 55.1 | 54.9 | 0.87 (0.42-1.78) |
| Rheumatological Conditions | Zanetti et al | Increased COVID-19 mortality in patients with rheumatic diseases: results from the CONTROL-19 study by the Italian Society for Rheumatology | 2022 | Retrospective cohort study | Italy | 668 | 1395075 | 58.4 |  | 3.1 (2.29-4.12) |
|  |  |  |  |  |  |  |  |  |  |  |
|  |  |  |  |  |  |  |  |  |  |  |
| **TOTAL** |  |  |  |  |  | **1542097** | **56248181** |  |  |  |

*Figures excluded from totals to prevent multiple counts from same cohorts

** Imputed figures
