# Supplementary material for: Disparities in COVID-19 mortality amongst the immunosuppressed: A systematic review and meta-analysis for enhanced disease surveillance
Source: J Infect. 2024 Mar;88(3):None. doi: 10.1016/j.jinf.2024.01.009 (PMC10943183; doi:10.1016/j.jinf.2024.01.009)
Supplement: Supplementary file 7 — Supplementary material [file mmc7.docx]

**Appendix 7: The impact of effect measure type on pooled estimation of effect size**

| **Immunosuppression** | **Number of Study Entries** | **Effect Measure Type** | **Effect Size** | **Lower Bound** | **Upper Bound** | **p-value** | **I2** |
| --- | --- | --- | --- | --- | --- | --- | --- |
| Transplantation | 2 | HR | 1.61 | 1.1 | 2.35 | <0.001 | 0% |
|  | 1 | sHR | 1.38 | 0.67 | 2.83 | " | / |
|  | 15 | OR | 2.6 | 1.7 | 3.97 | " | 96.00% |
|  | 3 | RR | 0.95 | 0.78 | 1.16 | " | 0% |
| Malignancy | 34 | OR | 2.1 | 1.74 | 2.54 | 0.0005 | 93.00% |
|  | 1 | HR | 0.98 | 0.58 | 1.66 | " | / |
|  | 1 | RR | 1.15 | 0.84 | 1.57 | " | / |
|  | 1 | SMR | 2.04 | 1.77 | 2.35 | " | / |
| Immunosuppressive agents | 7 | OR | 1.84 | 1.63 | 2.08 | 0.13 | 38.80% |
|  | 1 | HR | 1.87 | 1.3 | 2.69 | " | / |
|  | 1 | RR | 0.87 | 0.42 | 1.79 | " | / |
| Rheumatological conditions | 14 | RR | 1.15 | 0.84 | 1.57 | 0.049 | 96.70% |
|  | 23 | OR | 1.42 | 1.21 | 1.65 | " | 73.50% |
|  | 6 | HR | 1.12 | 1.01 | 1.24 | " | 89.50% |
|  | 1 | SIR | 0.42 | 0.09 | 1.86 | " | / |
| HIV | 15 | OR | 1.12 | 0.96 | 1.31 | 0.15 | 51.30% |
|  | 5 | HR | 1.62 | 1.21 | 2.17 | " | 67.00% |
|  | 4 | RR | 1.1 | 0.9 | 1.36 | " | 73.70% |
|  | 2 | IRR | 0.7 | 0.29 | 1.69 | " | 0% |
|  | 1 | SHR | 1.13 | 0.62 | 2.07 | " | / |
|  |  |  |  |  |  |  |  |
