# Supplementary material for: Disparities in COVID-19 mortality amongst the immunosuppressed: A systematic review and meta-analysis for enhanced disease surveillance
Source: J Infect. 2024 Mar;88(3):None. doi: 10.1016/j.jinf.2024.01.009 (PMC10943183; doi:10.1016/j.jinf.2024.01.009)

**Appendix 8: Forest plots of all meta-analyses conducted**

1. Excess COVID-19 associated mortality between immunosuppressed categories
2. Forest Plot of excess COVID-19 associated mortality amongst transplant recipients


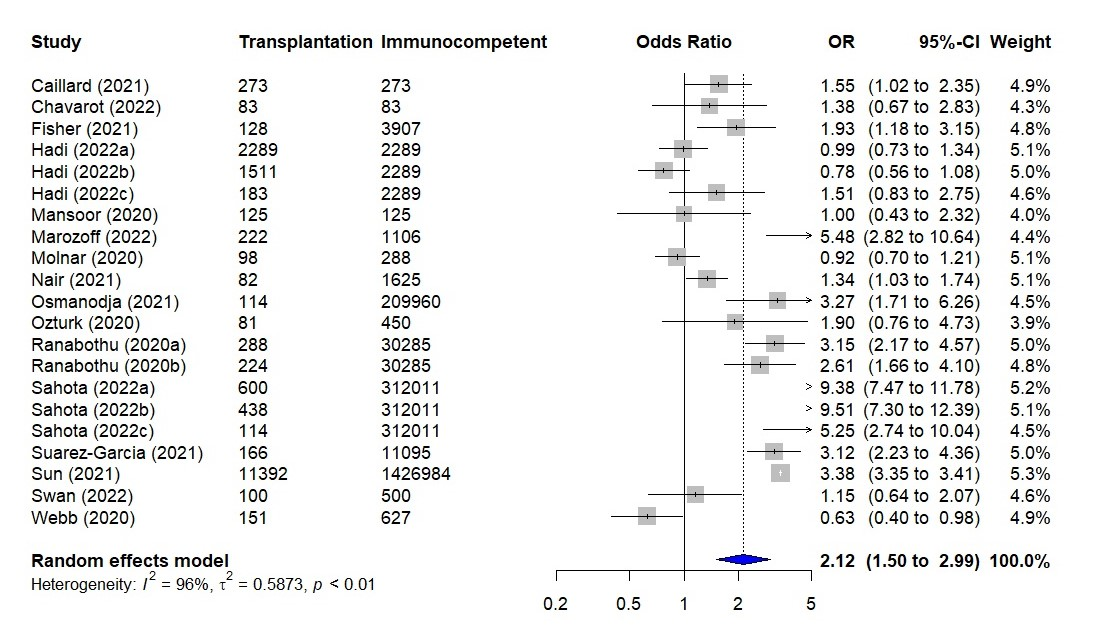


1. Forest Plot of excess COVID-19 associated mortality amongst patients with malignancies as compared to immunocompetent controls


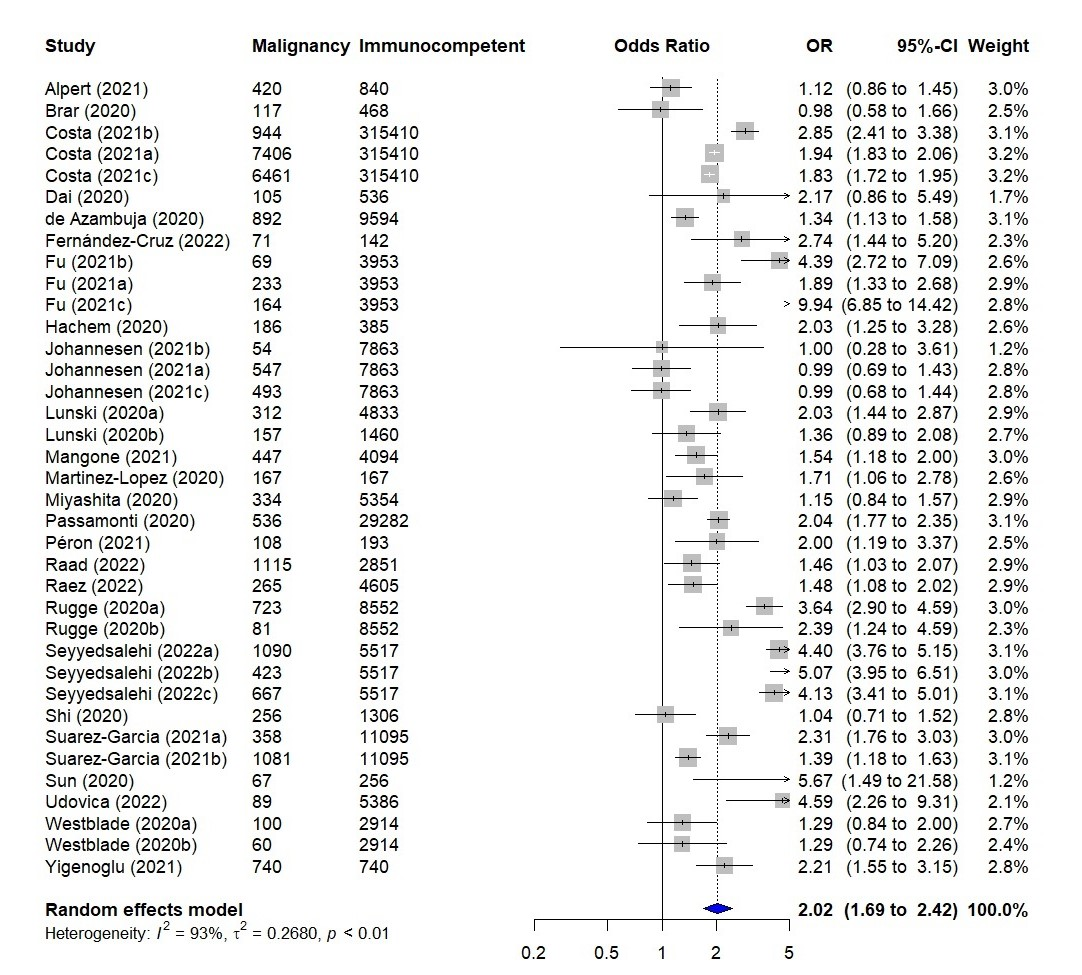


1. Forest Plot of excess COVID-19 associated mortality amongst recipients of immunosuppressive agents as compared to immunocompetent controls


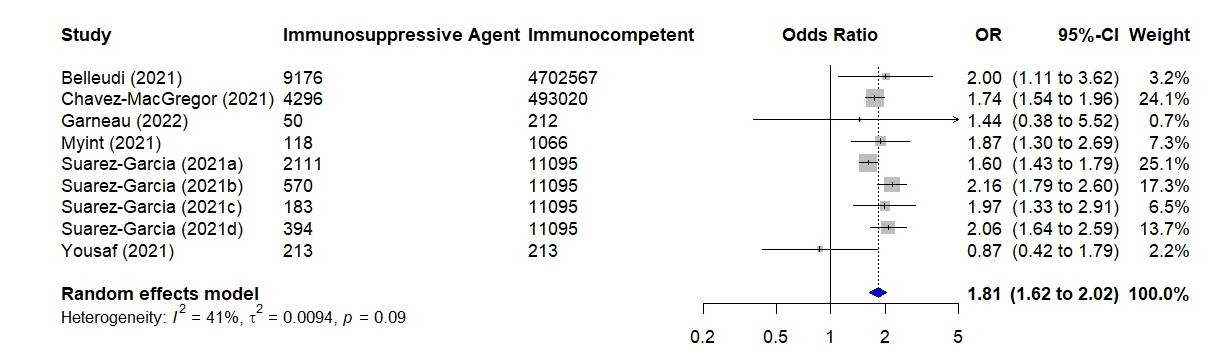


1. Forest Plot of excess COVID-19 associated mortality amongst rheumatology patients as compared to immunocompetent controls


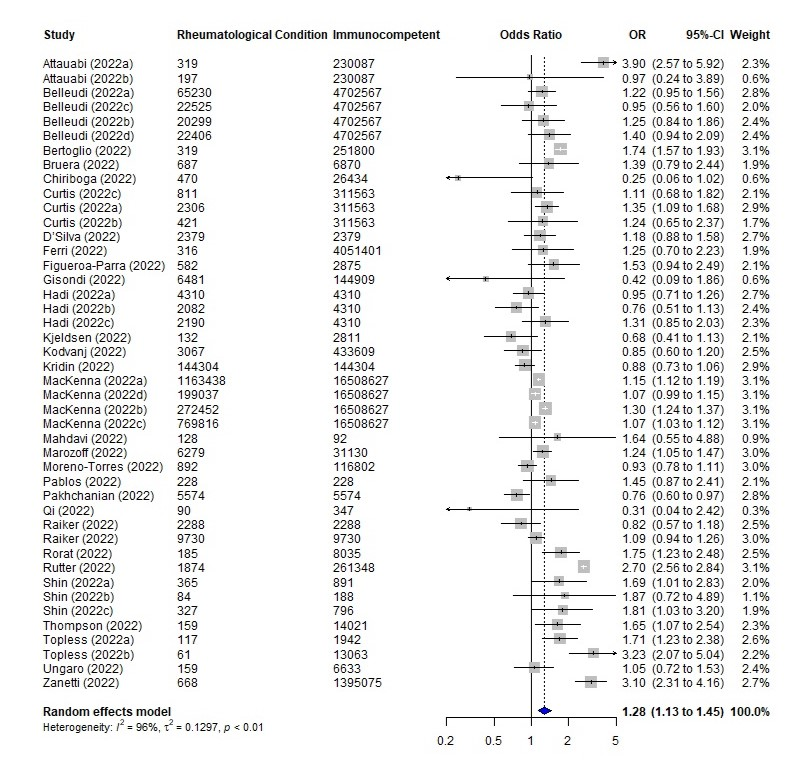


1. Forest Plot of excess COVID-19 associated mortality amongst HIV patients as compared to immunocompetent controls


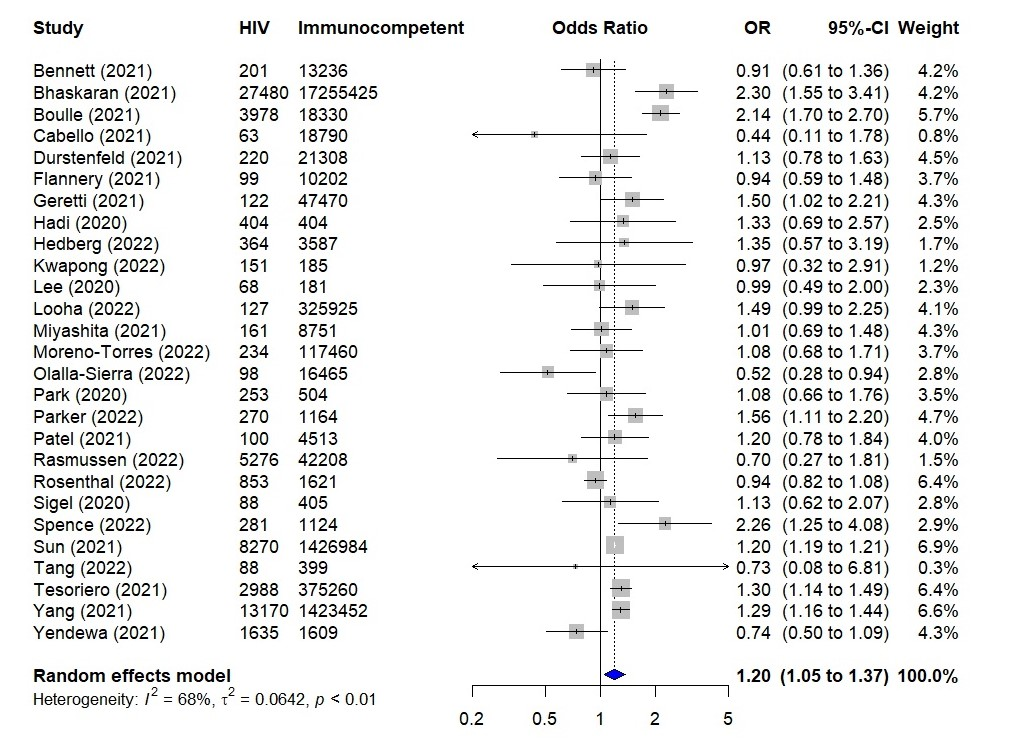


1. Excess COVID-19 associated mortality between immunosuppressed subcategories
2. Forest Plot of excess COVID-19 associated mortality amongst subcategories of transplant patients as compared to immunocompetent controls


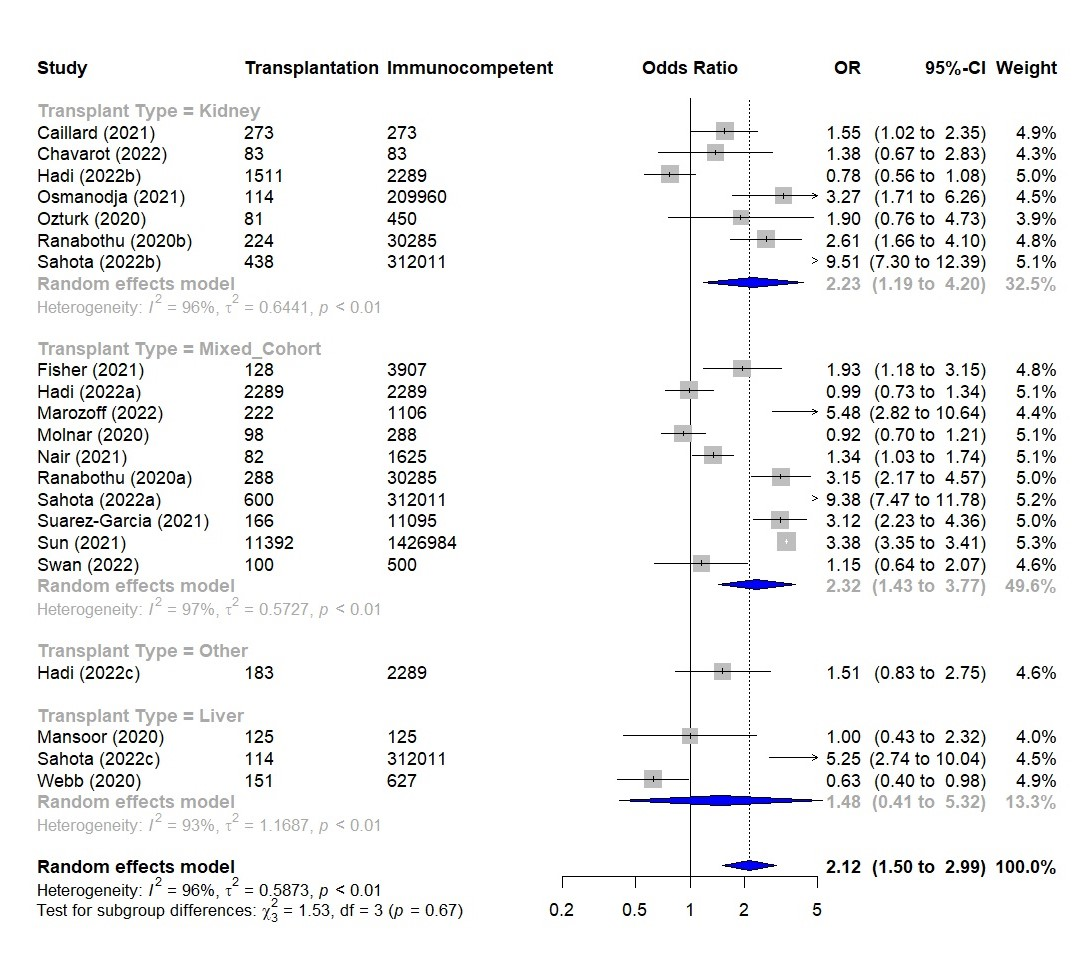


1. Forest Plot of excess COVID-19 associated mortality amongst subcategories of malignancy patients as compared to immunocompetent controls


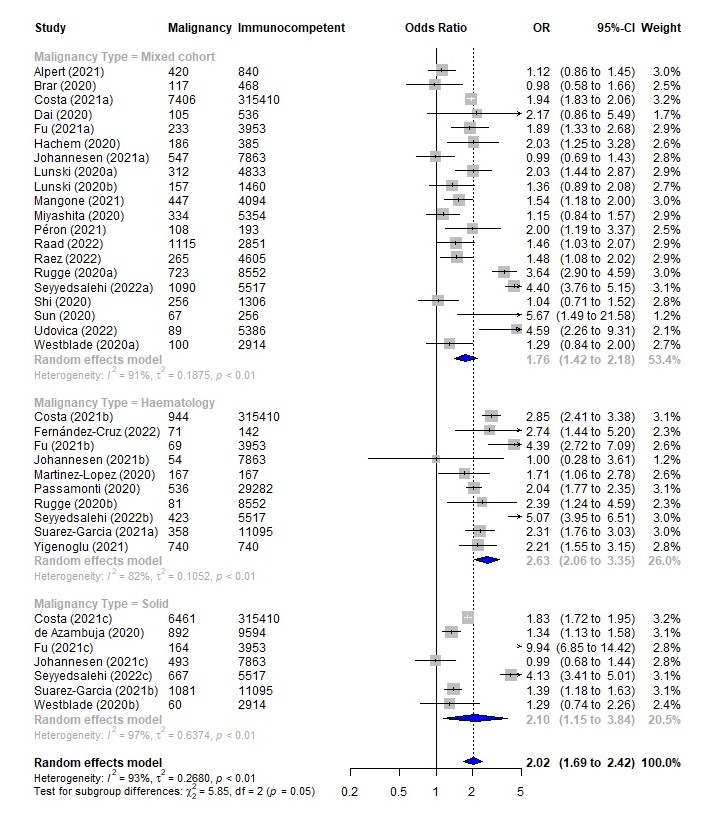


1. Forest Plot of excess COVID-19 associated mortality amongst subcategories of patients receiving immunosuppressive agents as compared to immunocompetent controls


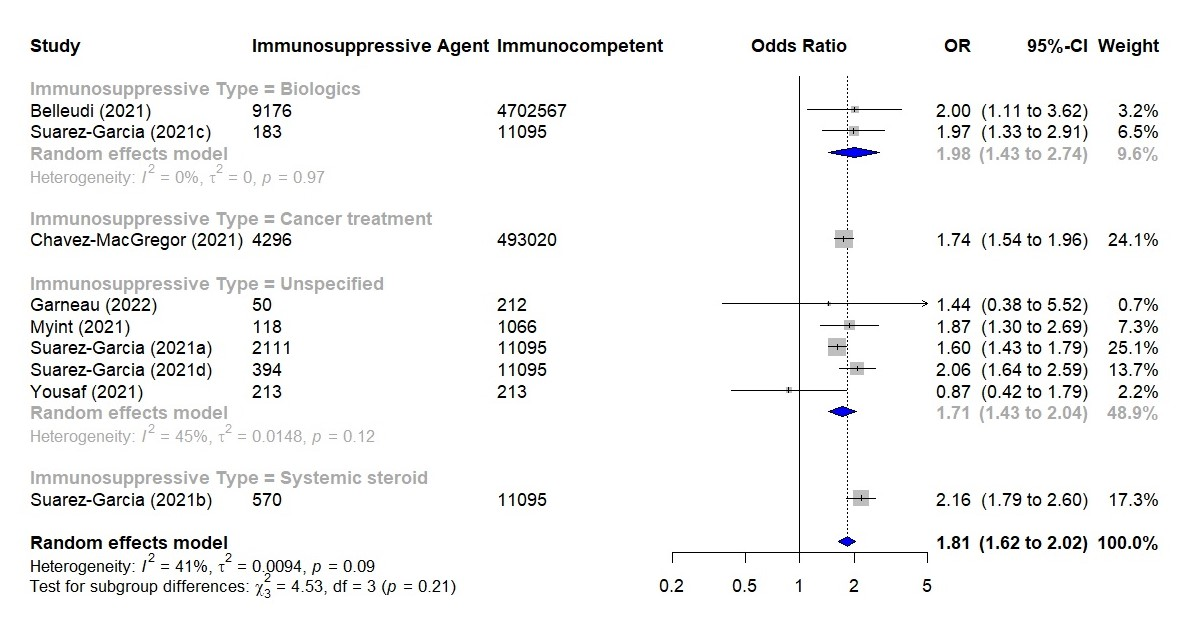


1. Forest Plot of excess COVID-19 associated mortality amongst subcategories of rheumatology patients as compared to immunocompetent controls


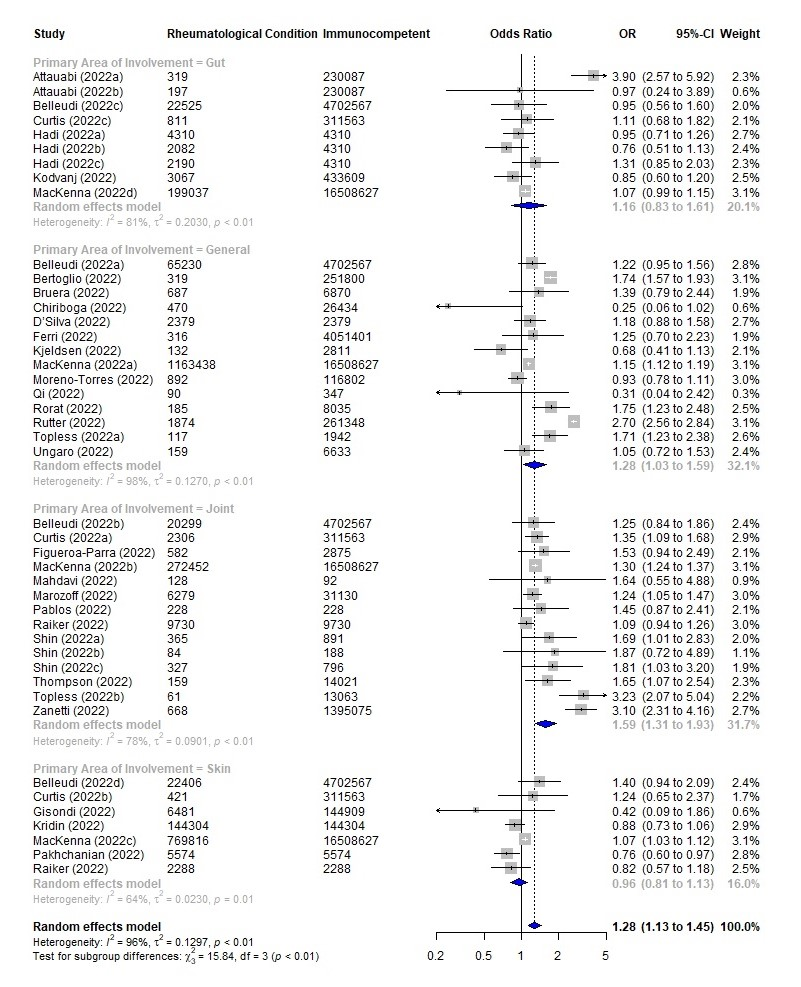


1. Attenuation of excess COVID-19 mortality by income category of study country
2. Subgroup analysis of COVID-19 associated mortality in transplant patients versus immunocompetent controls by income of study country


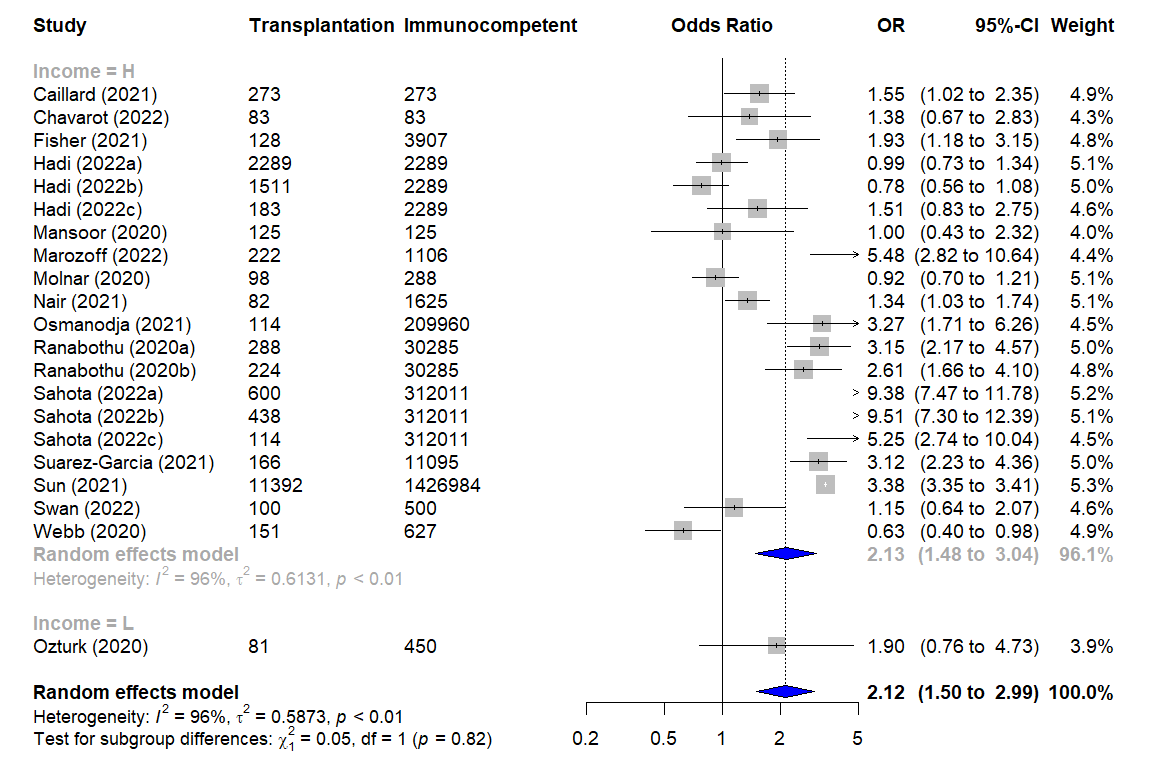


1. Subgroup analysis of COVID-19 associated mortality in malignancy patients versus immunocompetent controls by income of study country


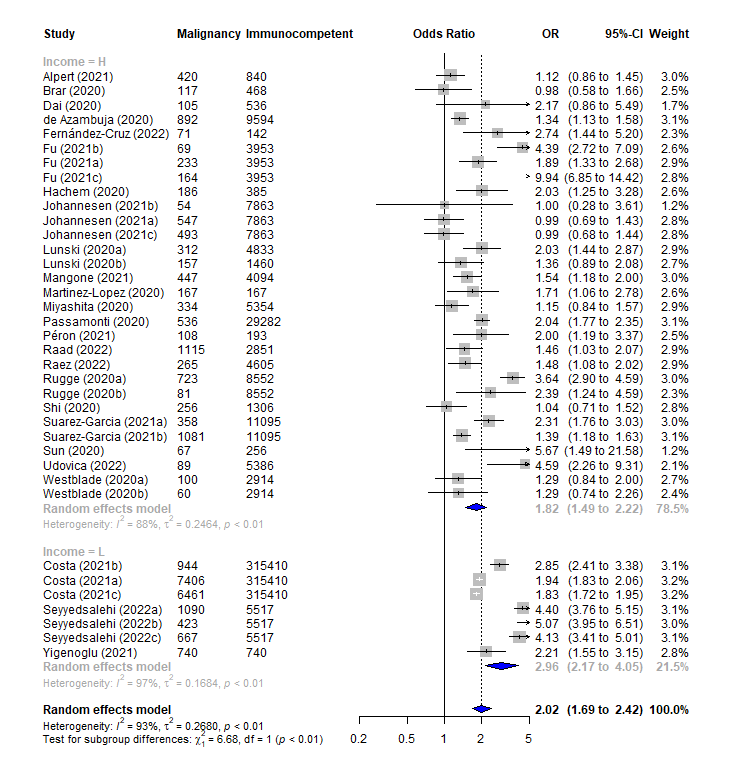


1. Subgroup analysis of COVID-19 associated mortality in immunosuppressive agent patients versus immunocompetent controls by income of study country


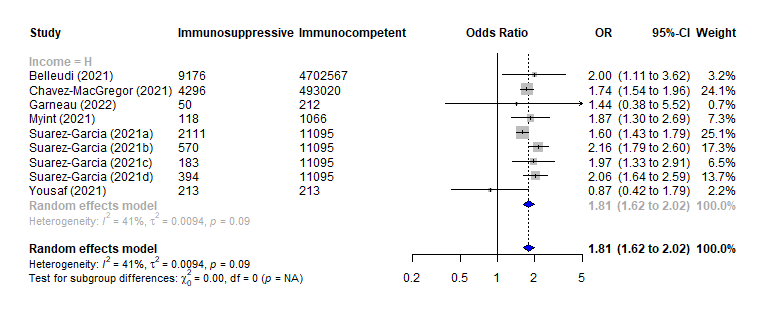


1. Subgroup analysis of COVID-19 associated mortality in rheumatological patients versus immunocompetent controls by income of study country


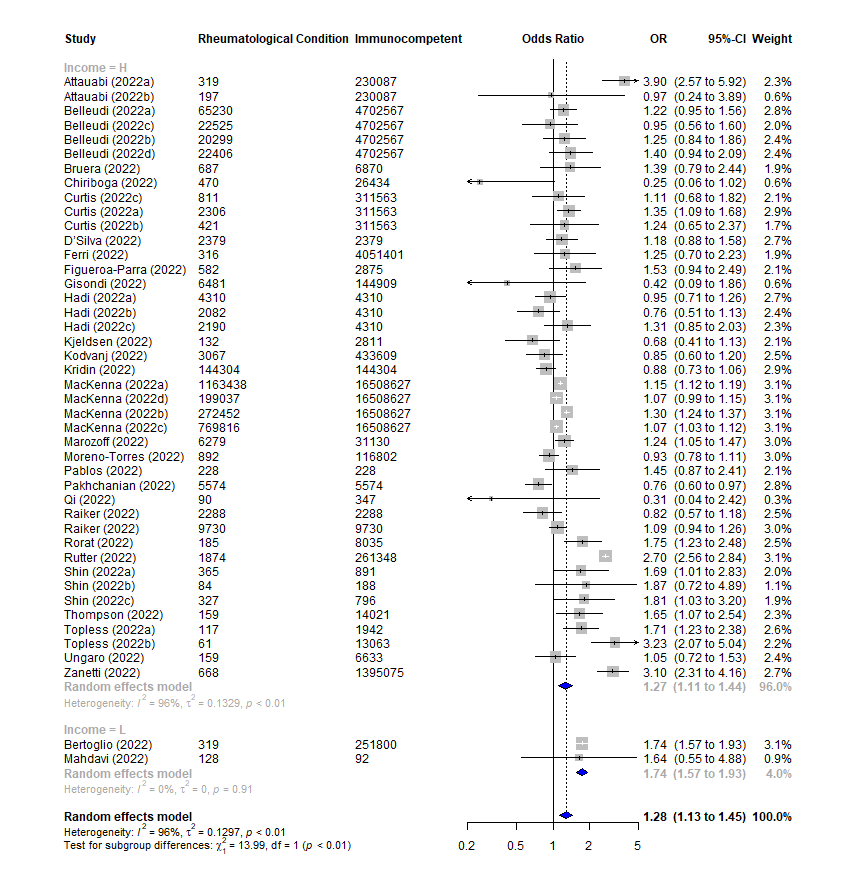


1. Subgroup analysis of COVID-19 associated mortality in HIV patients versus immunocompetent controls by income of study country


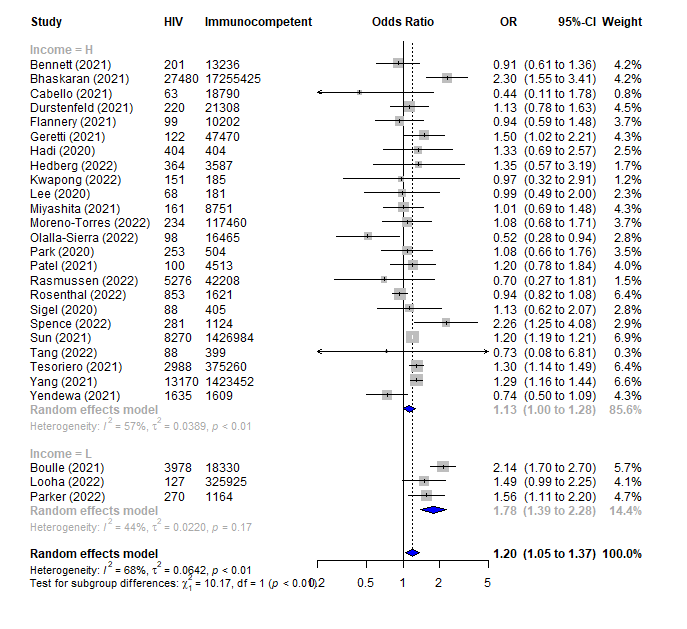


1. Attenuation of excess COVID-19 mortality by COVID-19 case type
2. Subgroup analysis of COVID-19 associated mortality in transplant patients versus immunocompetent controls by case type


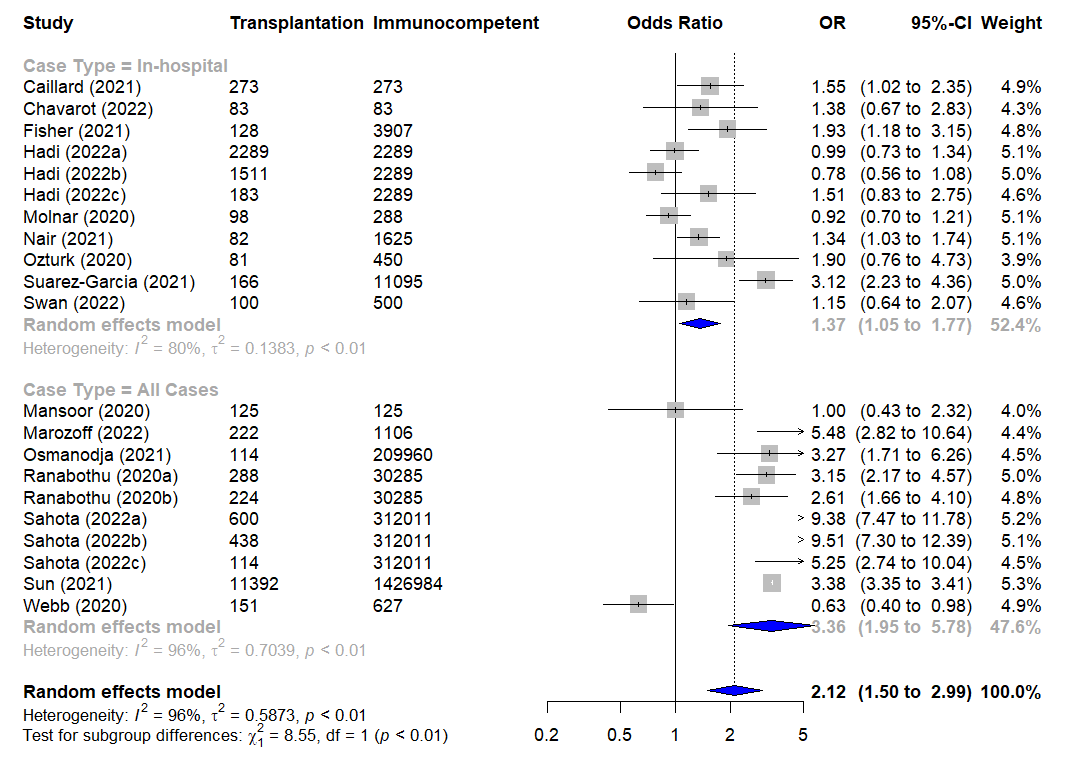


1. Subgroup analysis of COVID-19 associated mortality in patients with malignancies versus immunocompetent controls by case type


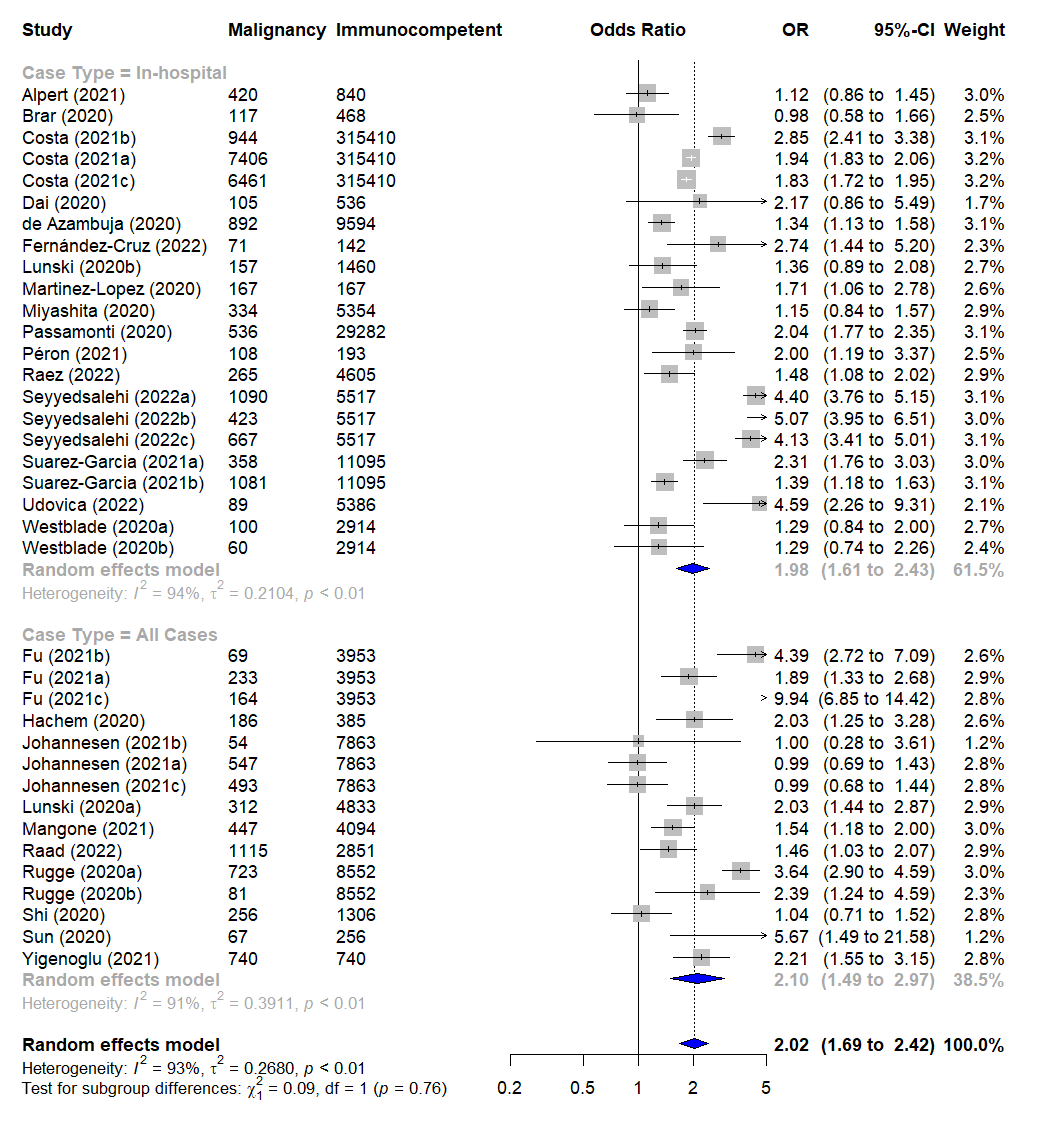


1. Subgroup analysis of COVID-19 associated mortality in patients on immunosuppressives versus immunocompetent controls by case type


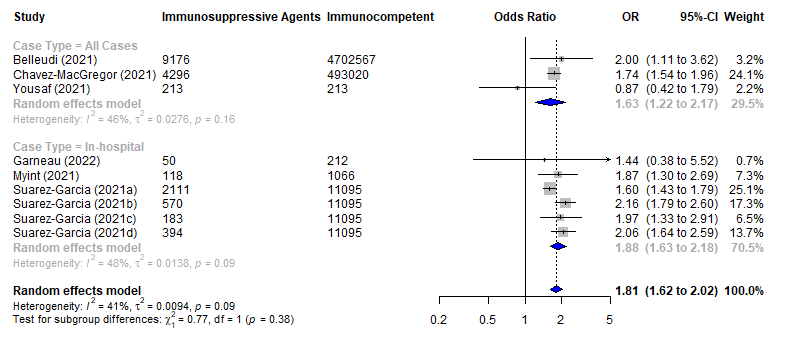


1. Subgroup analysis of COVID-19 associated mortality in rheumatology patients versus immunocompetent controls by case type


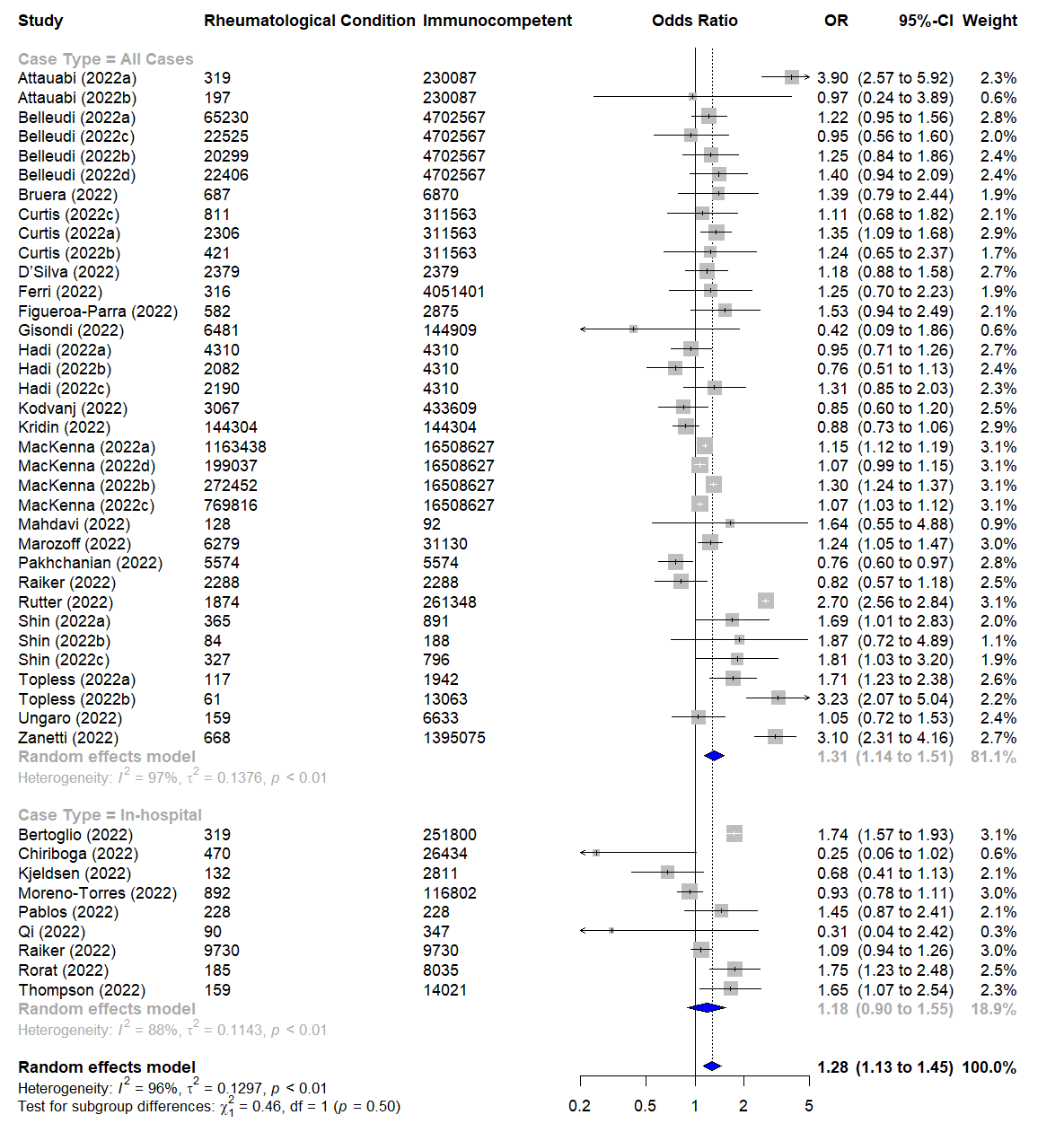


1. Subgroup analysis of COVID-19 associated mortality in HIV patients versus immunocompetent controls by case type


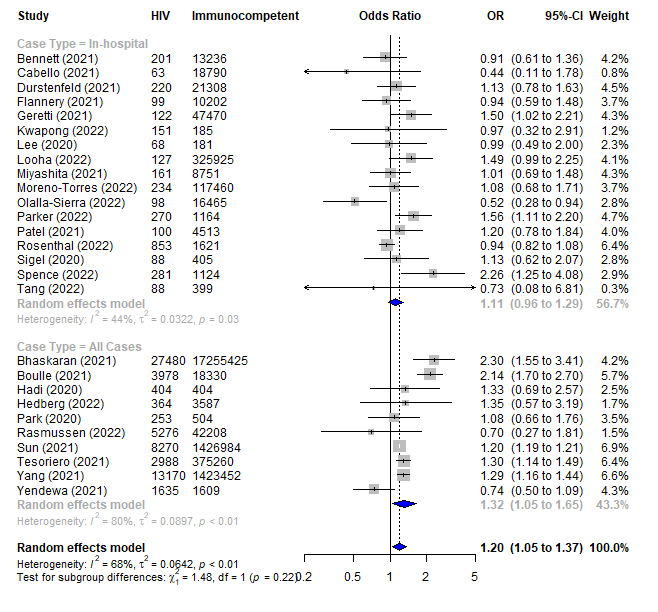


1. Attenuation of excess COVID-19 mortality by level of data adjustment
2. Subgroup analysis of COVID-19 associated mortality in transplant patients versus immunocompetent controls by data adjustment


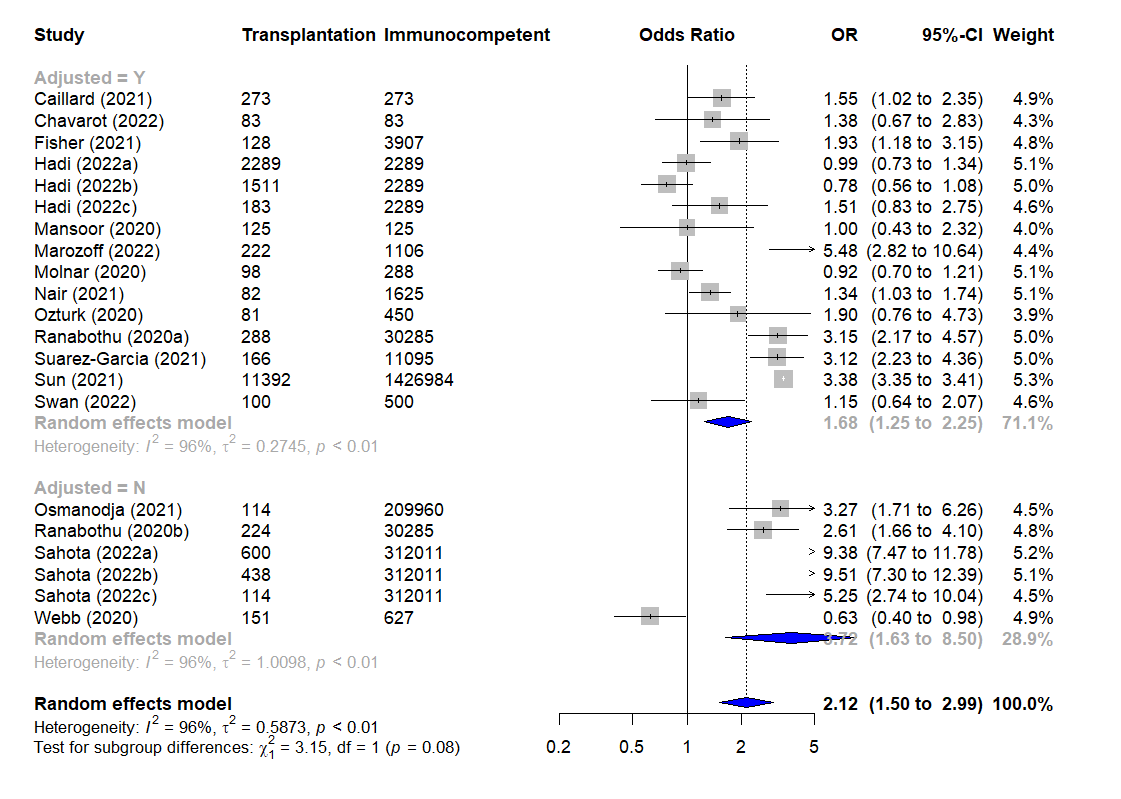


1. Subgroup analysis of COVID-19 associated mortality in malignancy patients versus immunocompetent controls by data adjustment


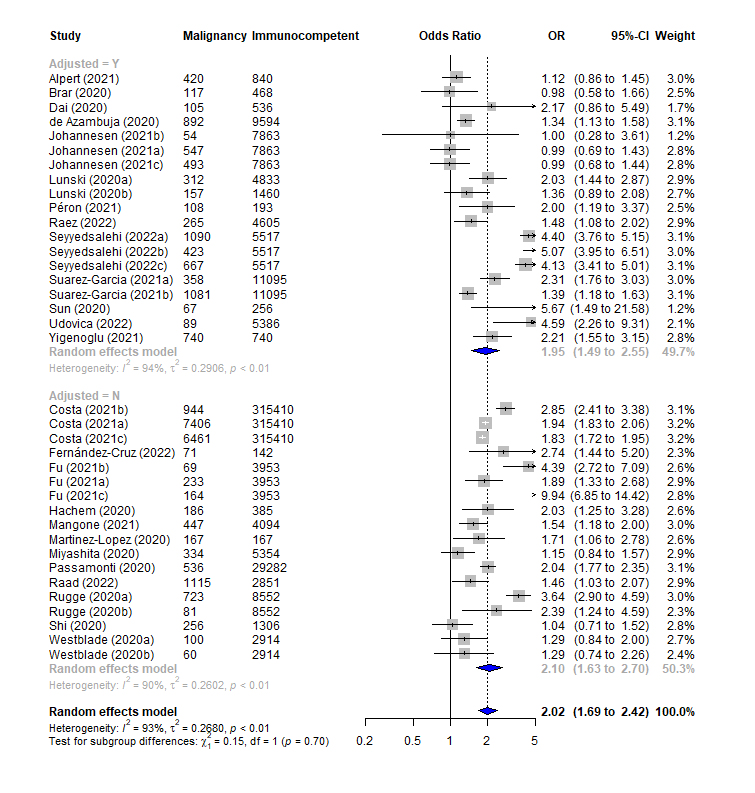


1. Subgroup analysis of COVID-19 associated mortality in immunosuppressive agent patients versus immunocompetent controls by data adjustment


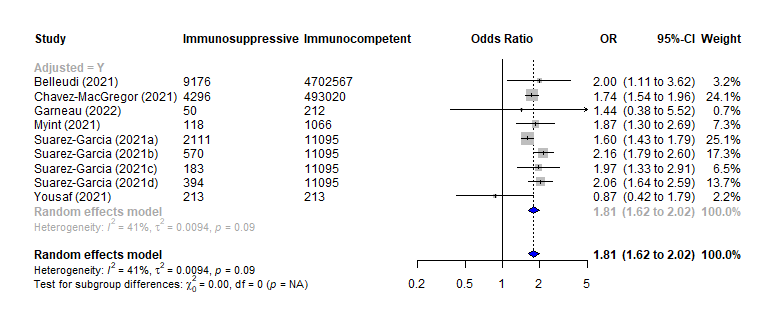


1. Subgroup analysis of COVID-19 associated mortality in rheumatological patients versus immunocompetent controls by data adjustment


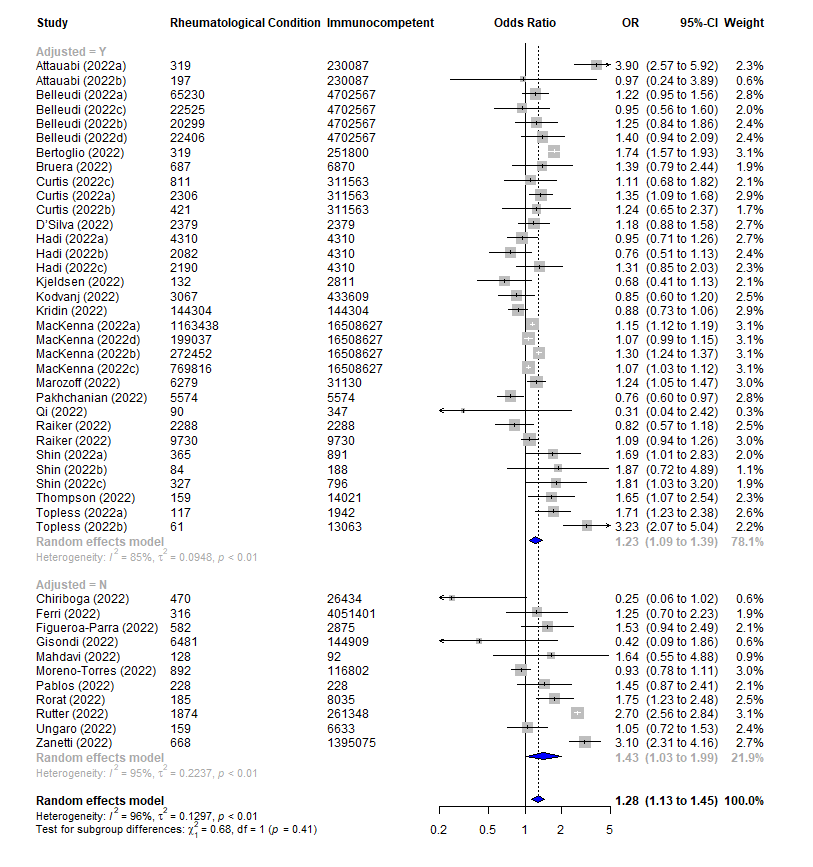


1. Subgroup analysis of COVID-19 associated mortality in HIV patients versus immunocompetent controls by data adjustment


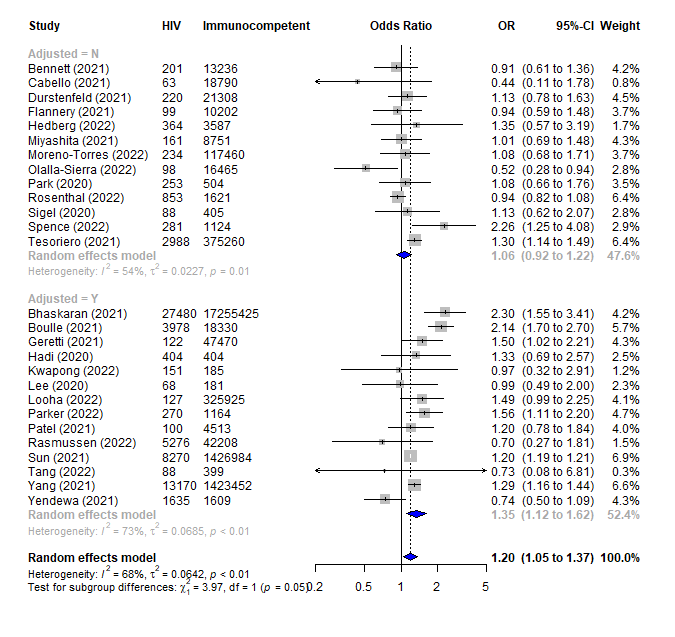


1. Attenuation of excess COVID-19 mortality by level of data matching
2. Subgroup analysis of COVID-19 associated mortality in transplant patients versus immunocompetent controls by data matching


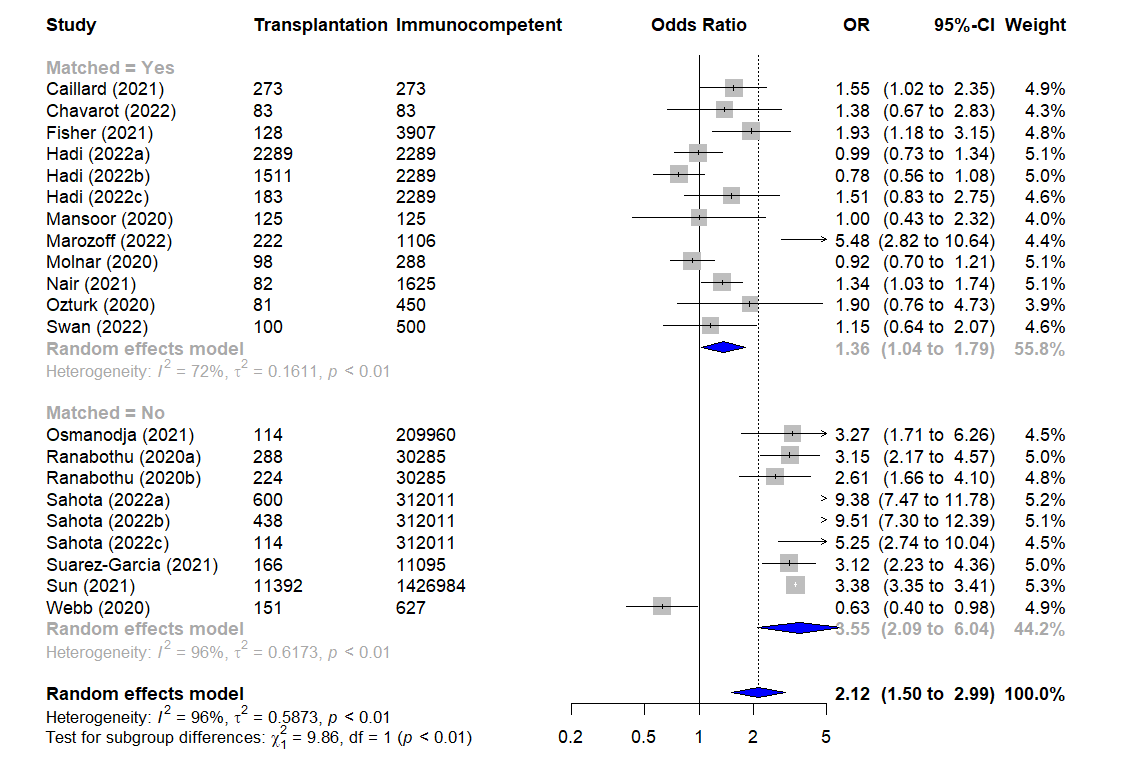


1. Subgroup analysis of COVID-19 associated mortality in malignancy patients versus immunocompetent controls by data matching


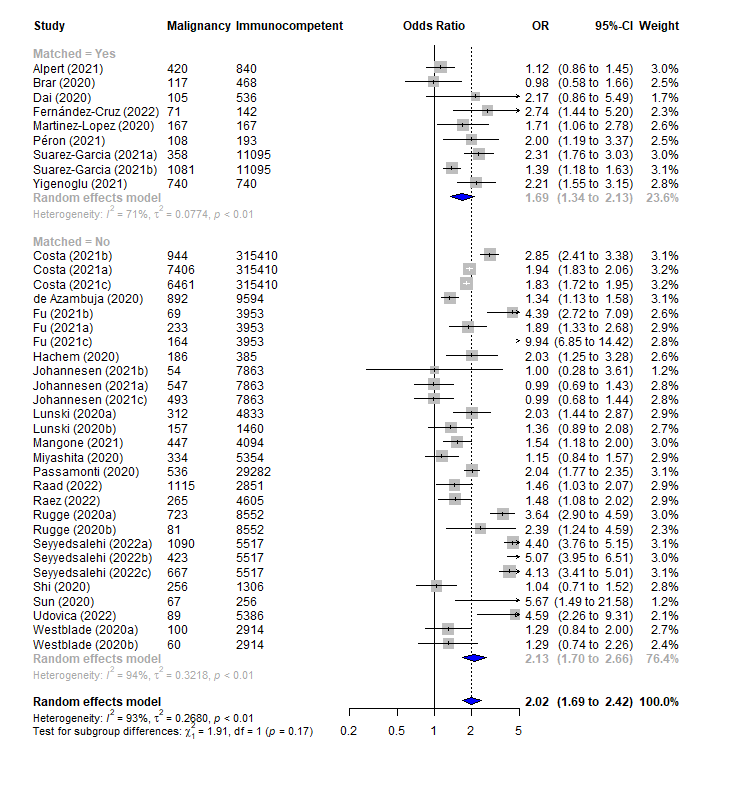


1. Subgroup analysis of COVID-19 associated mortality in immunosuppressive agent patients versus immunocompetent controls by data matching


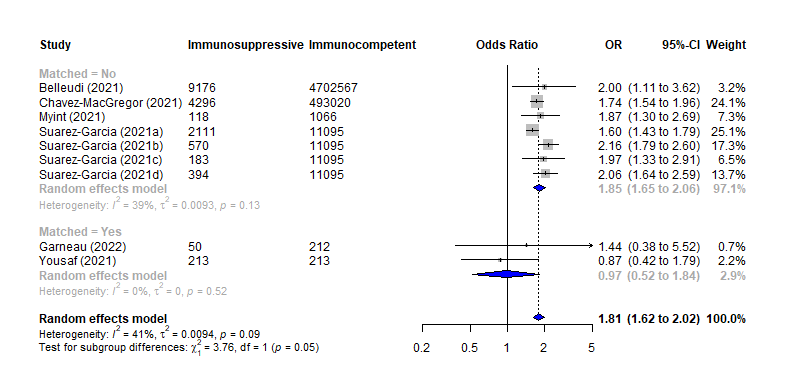


1. Subgroup analysis of COVID-19 associated mortality in rheumatological patients versus immunocompetent controls by data matching


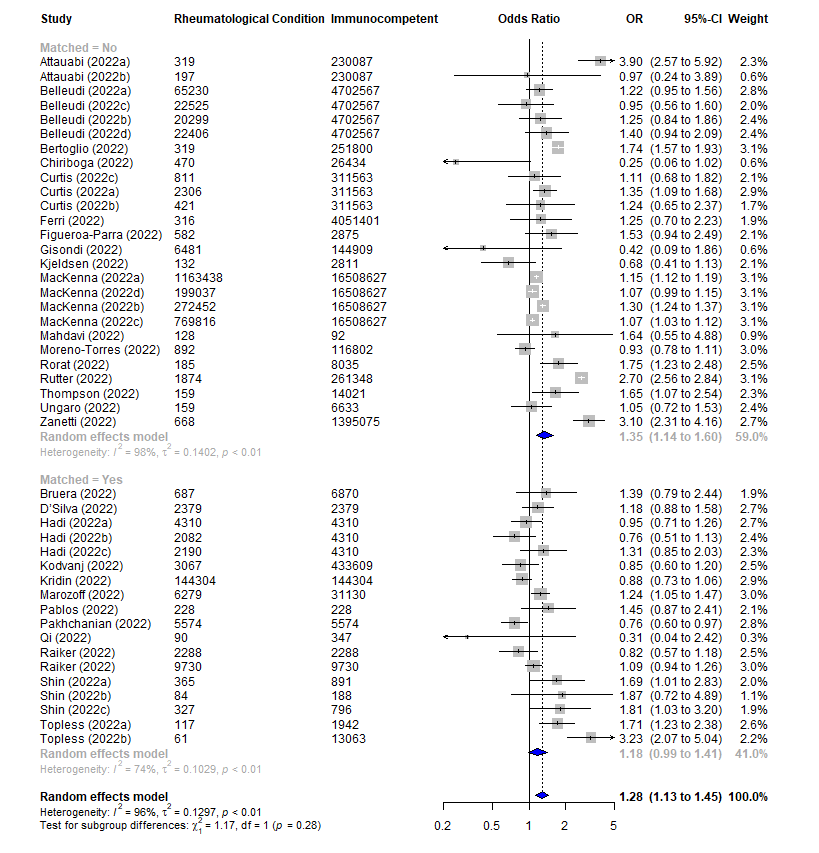


1. Subgroup analysis of COVID-19 associated mortality in HIV patients versus immunocompetent controls by data matching


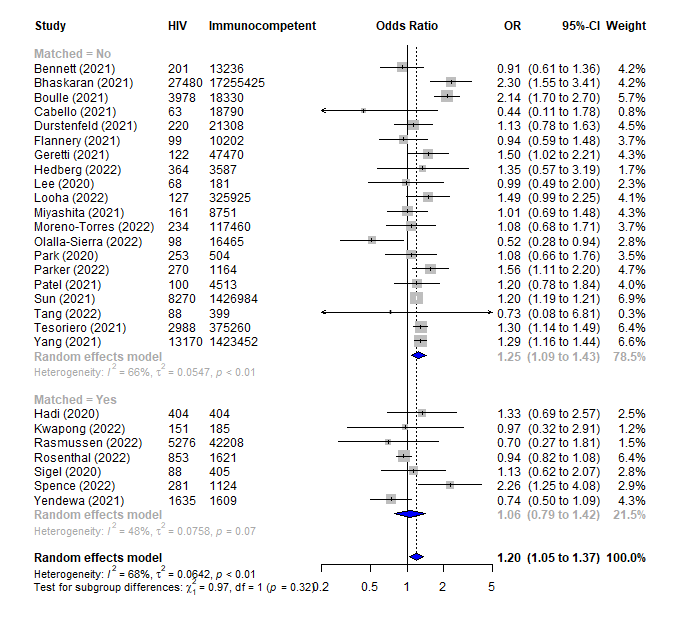


1. Attenuation of excess COVID-19 mortality by effect measure type
2. Subgroup analysis of COVID-19 associated mortality in transplant patients versus immunocompetent controls by effect measure type


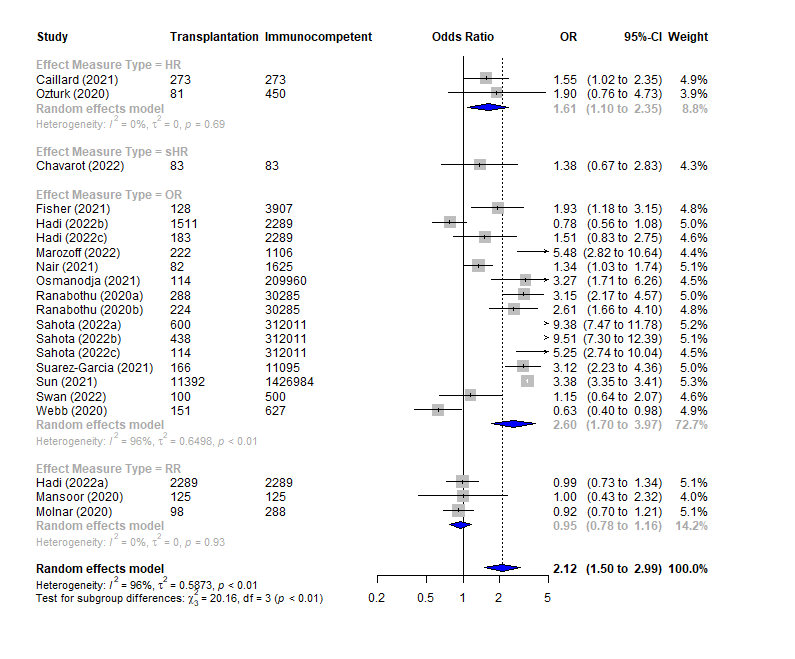


1. Subgroup analysis of COVID-19 associated mortality in malignancy patients versus immunocompetent controls by effect measure type


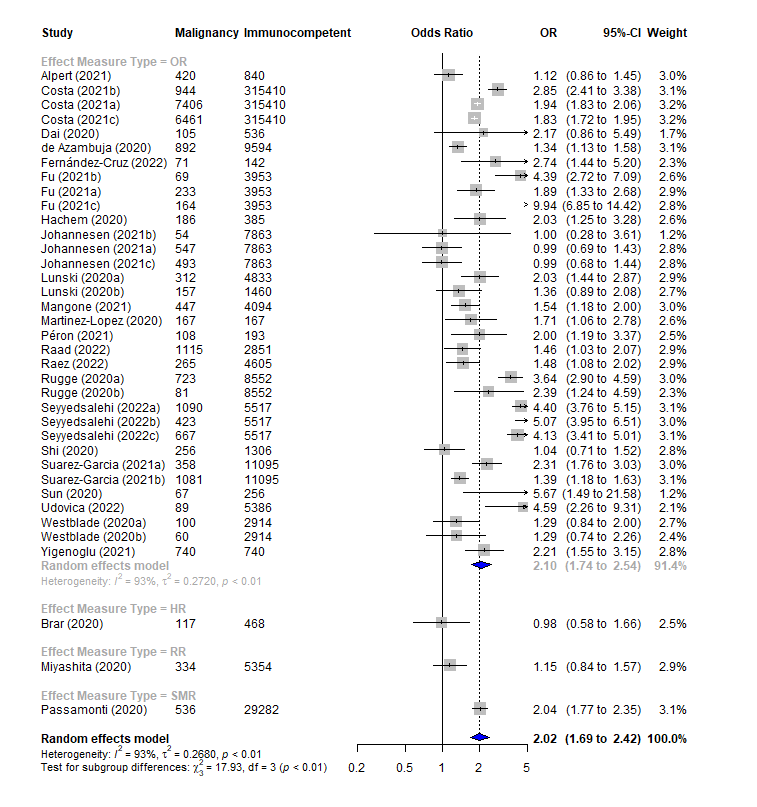


1. Subgroup analysis of COVID-19 associated mortality in immunosuppressive agent patients versus immunocompetent controls by effect measure type


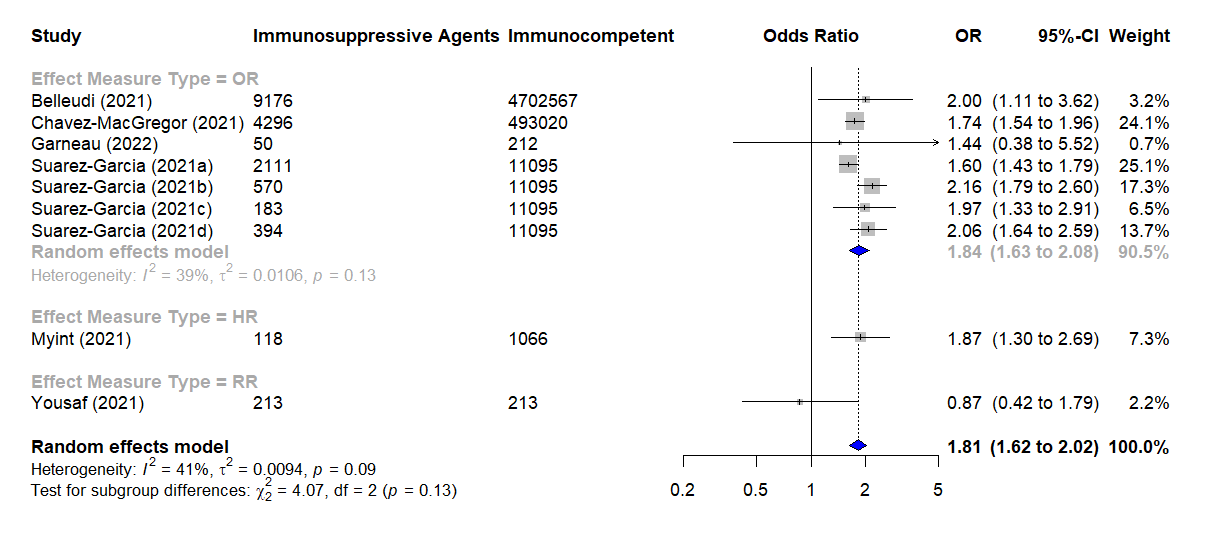


1. Subgroup analysis of COVID-19 associated mortality in rheumatological patients versus immunocompetent controls by effect measure type


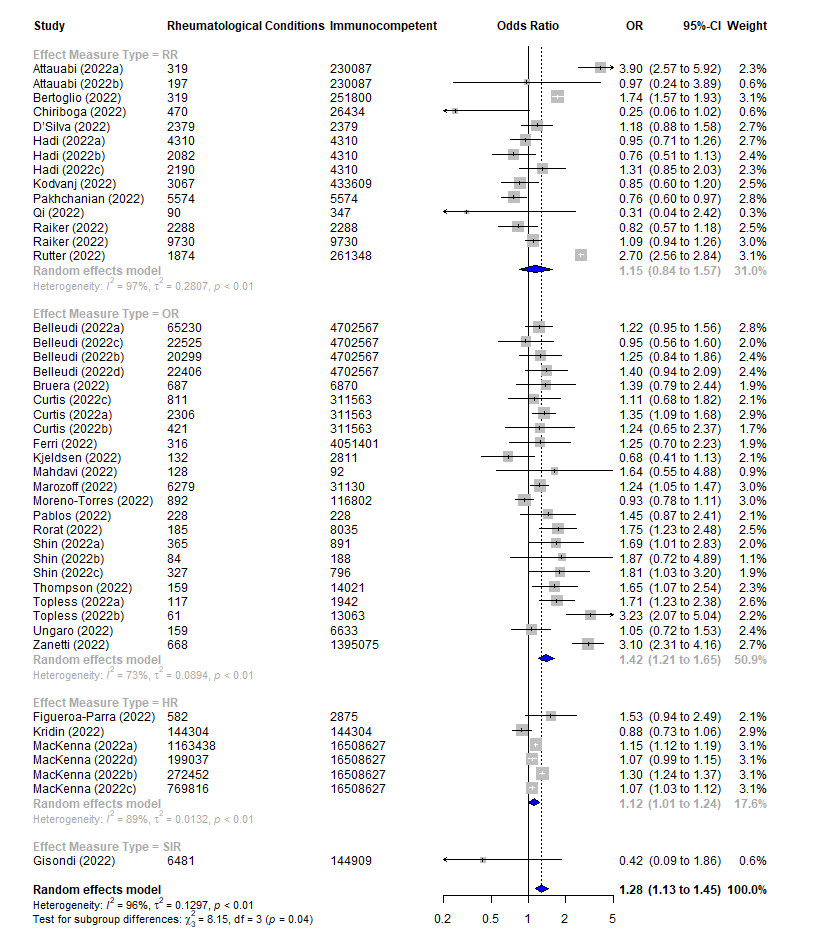


1. Subgroup analysis of COVID-19 associated mortality in HIV patients versus immunocompetent controls by effect measure type


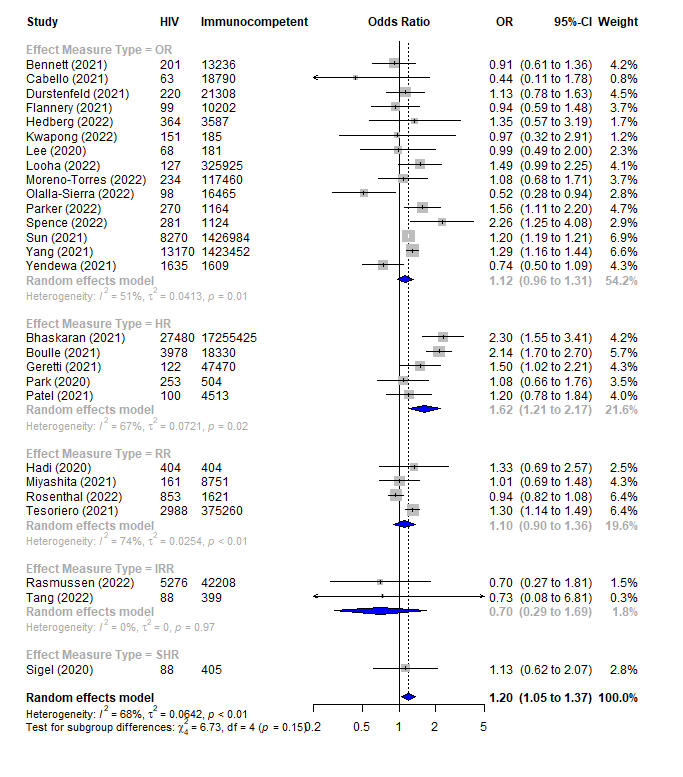

Supplement: Supplementary file 8 — Supplementary material [file mmc8.docx]
