# Supplementary material for: Disparities in COVID-19 mortality amongst the immunosuppressed: A systematic review and meta-analysis for enhanced disease surveillance
Source: J Infect. 2024 Mar;88(3):None. doi: 10.1016/j.jinf.2024.01.009 (PMC10943183; doi:10.1016/j.jinf.2024.01.009)

**Appendix 9: Modelling for the influence of age over excess COVID-19 associated mortality between immunosuppressed categories**

**Table 1:** Summary of meta-regression outputs between average age of study participants and excess COVID-19 associated mortality across immunosuppressed categories

| **Immunosuppression** | **Estimate** | **Standard Error** | **p-value** | **Lower slope** | **Upper slope** |
| --- | --- | --- | --- | --- | --- |
| Transplantation | -0.01 | 0.0029 | 0.72 | -0.07 | 0.05 |
| Malignancy | -0.03 | 0.02 | 0.09 | -0.06 | 0.09 |
| Immunosuppressive Agents | -0.0029 | 0.0067 | 0.66 | -0.02 | 0.01 |
| Rheumatological conditions | -0.005 | 0.01 | 0.71 | -0.03 | 0.02 |
| HIV | -0.01 | 0.113 | 0.24 | -0.035 | 0.009 |

1. Bubble Plot of impact of average age of study participants over excess COVID associated mortality amongst transplant recipients


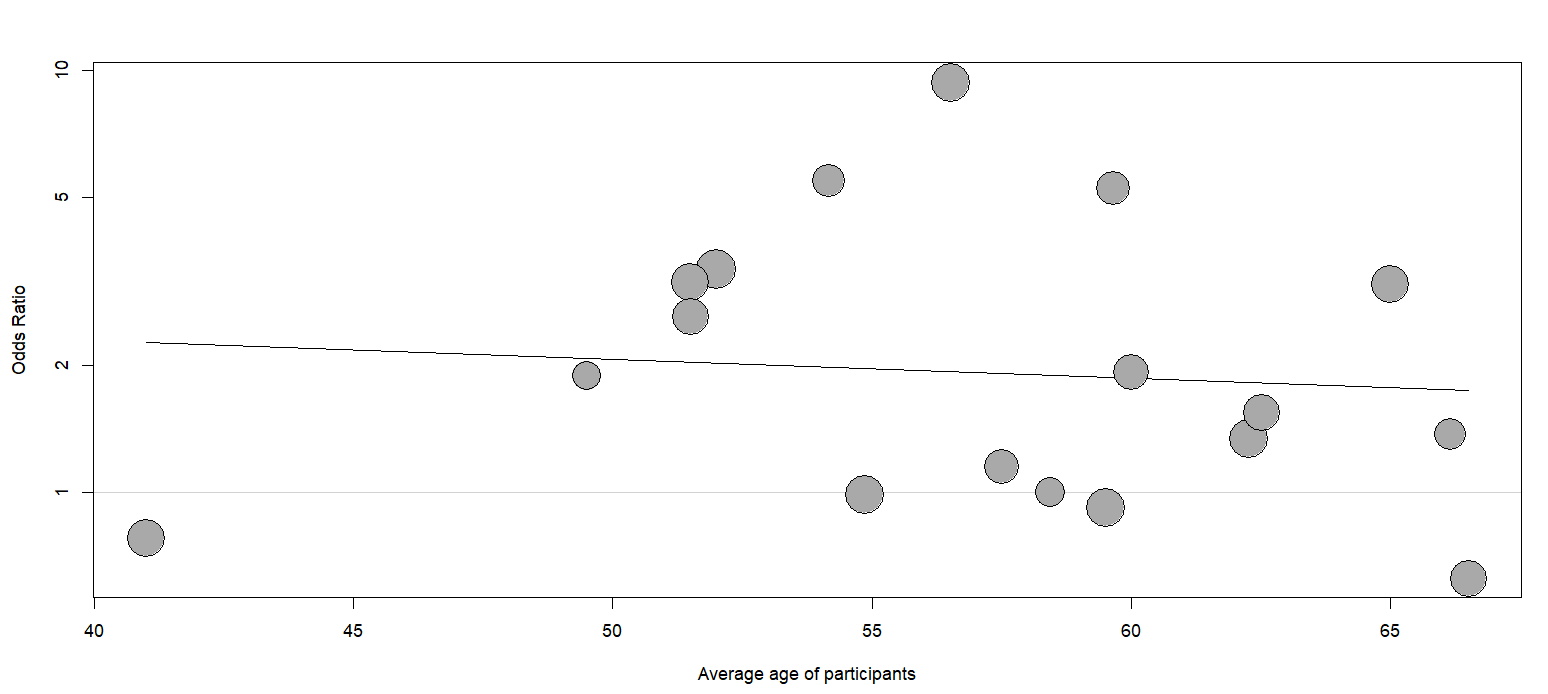


1. Bubble Plot of impact of average age of study participants over excess COVID associated mortality amongst patients with malignancies


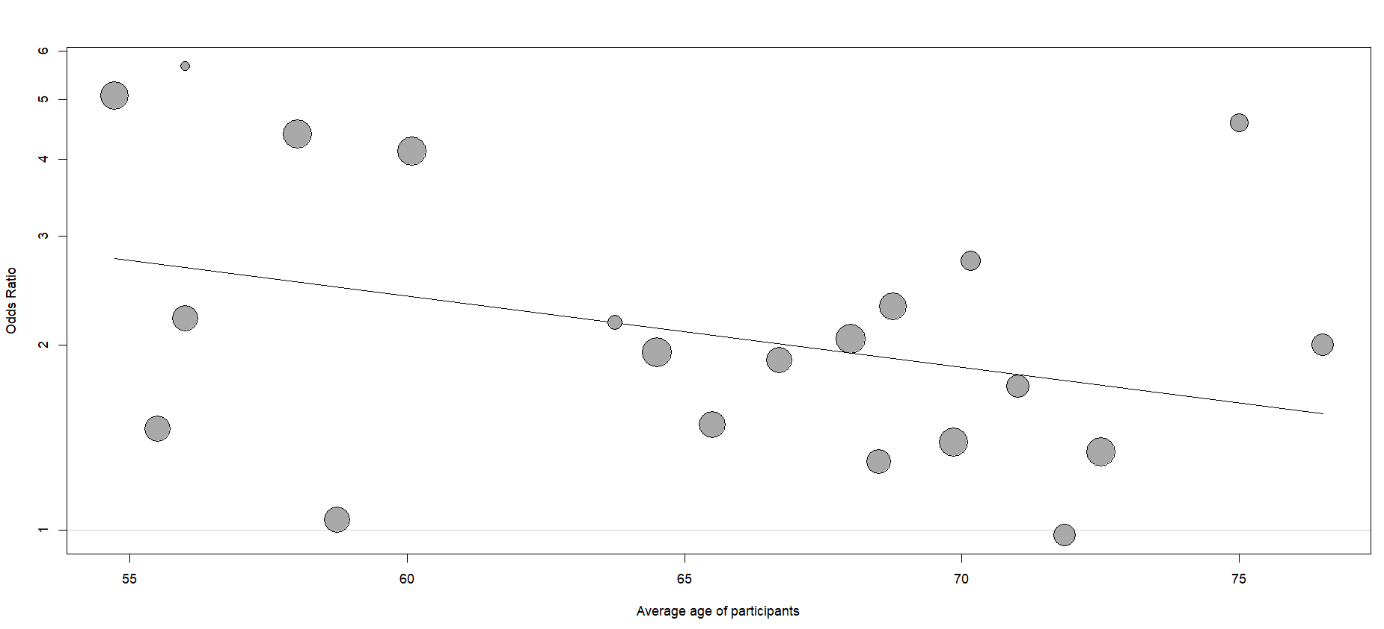


1. Bubble Plot of impact of average age of study participants over excess COVID associated mortality amongst immunosuppressive recipients


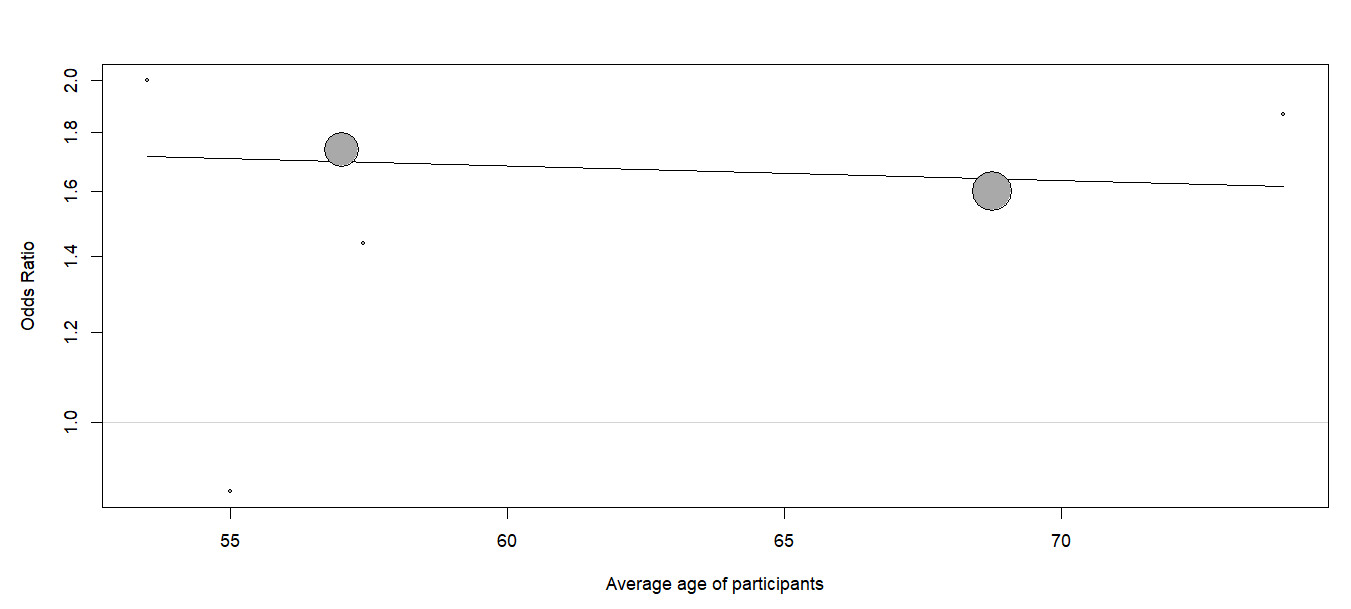


1. Bubble Plot of impact of average age of study participants over excess COVID associated mortality amongst rheumatology patients


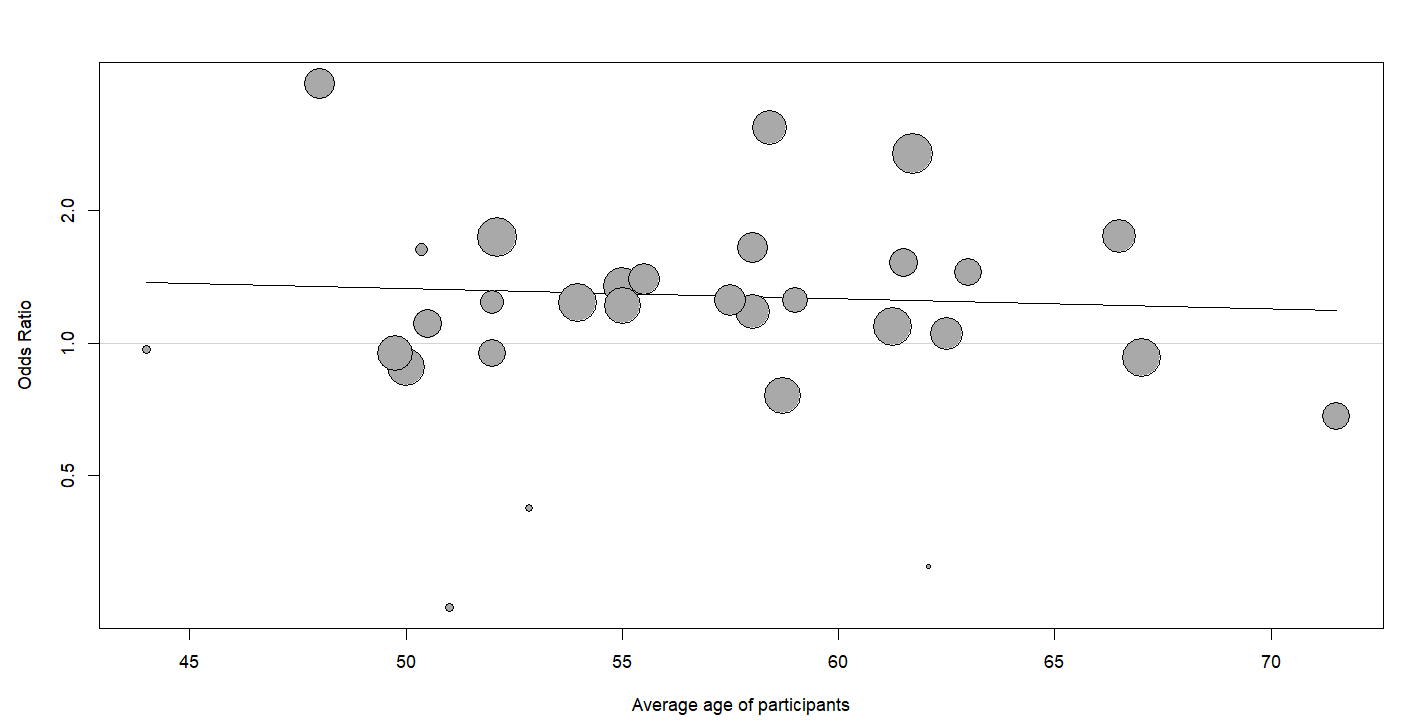


1. Bubble Plot of impact of average age of study participants over excess COVID associated mortality amongst HIV patients


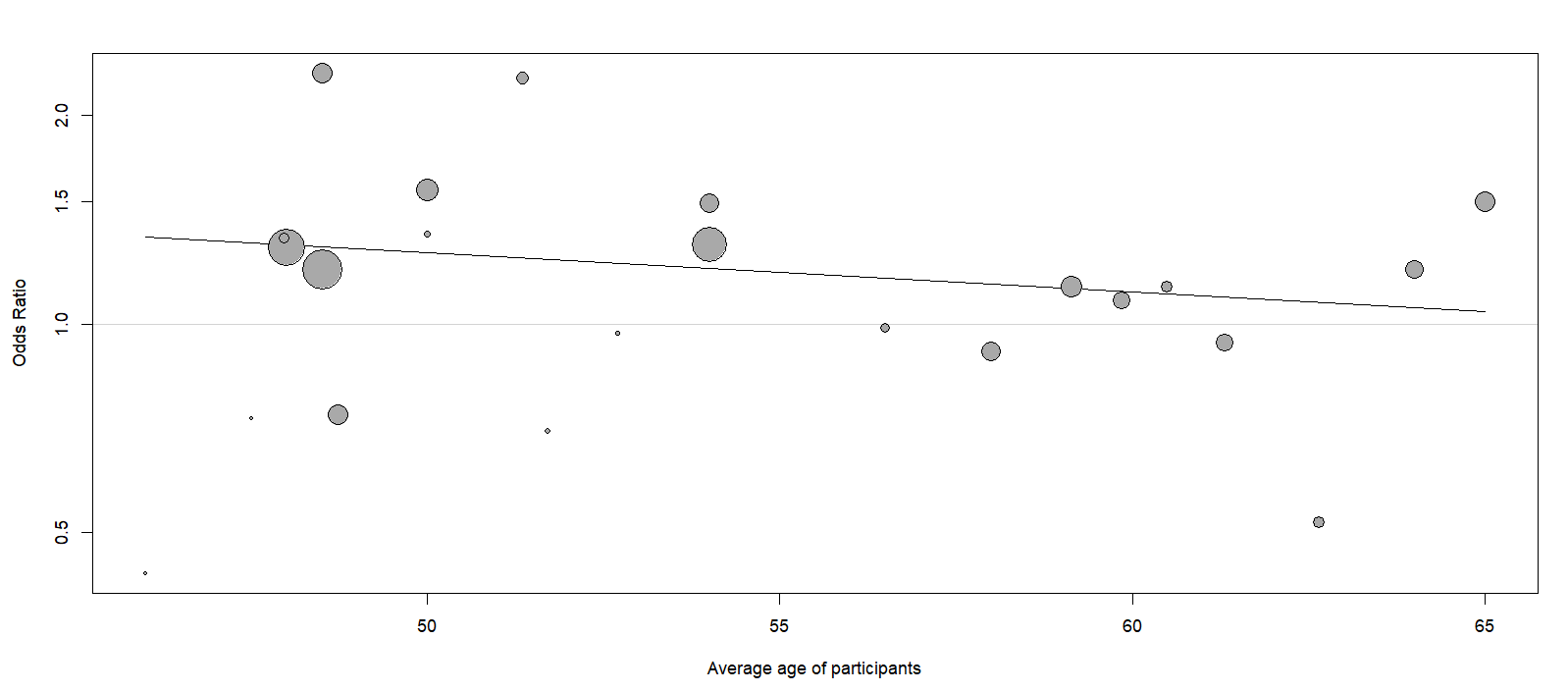

Supplement: Supplementary file 9 — Supplementary material [file mmc9.docx]
