# Supplementary material for: Disparities in COVID-19 mortality amongst the immunosuppressed: A systematic review and meta-analysis for enhanced disease surveillance
Source: J Infect. 2024 Mar;88(3):None. doi: 10.1016/j.jinf.2024.01.009 (PMC10943183; doi:10.1016/j.jinf.2024.01.009)
Supplement: Supplementary file 10 — Supplementary material [file mmc10.docx]

**Appendix 10: Exclusion Justifications at Phase 1 and Phase 2**

1. **Phase 1 Exclusion Justifications**

| **Authors** | **Title** | **Year** | **Exclusion Factor** |
| --- | --- | --- | --- |
| Abate, H. K. and Mekonnen, C. K. and Ferede, Y. M. | Depression Among HIV-Positive Pregnant Women at Northwest Amhara Referral Hospitals During COVID-19 Pandemic | 2021 | Wrong outcome |
| Abdulbasit, M. and Salameh, O. K. and Dauleh, M. M. and Zebi, A. M. and Verma, N. and Ghahramani, N. | Mortality among ESKD patients with COVID-19: comparison between kidney transplant and hemodialysis | 2021 | Insufficient original data |
| Abelenda-Alonso, G. and Rombauts, A. and Gudiol, C. and Oriol, I. and Simonetti, A. and Coloma, A. and RodrÃ­guez-Molinero, A. and Izquierdo, E. and DÃ­az-Brito, V. and SanmartÃ­, M. and PadullÃ©s, A. and Grau, I. and Ras, M. and Bergas, A. and Guillem, L. and Blanco-ArÃ©valo, A. and Alvarez-Pouso, C. and PallarÃ©s, N. and Videla, S. and TebÃ©, C. and CarratalÃ , J. | Immunomodulatory therapy, risk factors and outcomes of hospital-acquired bloodstream infection in patients with severe COVID-19 pneumonia: a Spanish case-control matched multicentre study (BACTCOVID) | 2021 | Wrong outcome |
| Abernathy, M. E. and Dam, K. A. and Esswein, S. R. and Jette, C. A. and Bjorkman, P. J. | How Antibodies Recognize Pathogenic Viruses: Structural Correlates of Antibody Neutralization of HIV-1, SARS-CoV-2, and Zika | 2021 | Wrong outcome |
| Ademas, A. and Adane, M. and Keleb, A. and Berihun, G. and Lingerew, M. and Sisay, T. and Hassen, S. and Getachew, M. and Tesfaw, G. and Getaneh Feleke, D. and Addisu, E. and Berhanu, L. and Abebe, M. and Gizeyatu, A. and Abate, H. and Derso, A. | COVID-19 Prevention Practices and Associated Factors among Diabetes and HIV/AIDS Clients in South-Wollo Zone, Ethiopia: A Health Facility-Based Cross-Sectional Study | 2021 | Wrong outcome |
| Adugna, A. and Azanaw, J. and Sharew Melaku, M. | The Effect of COVID-19 on Routine HIV Care Services from Health Facilities in Northwest Ethiopia | 2021 | Wrong outcome |
| Aggarwal, A. and Akerman, A. and Milogiannakis, V. and Silva, M. R. and Walker, G. and Stella, A. O. and Kindinger, A. and Angelovich, T. and Waring, E. and Amatayakul-Chantler, S. and Roth, N. and Manni, S. and Hauser, T. and Barnes, T. and Condylios, A. and Yeang, M. and Wong, M. and Jean, T. and Foster, C. S. P. and Christ, D. and Hoppe, A. C. and Munier, M. L. and Darley, D. and Churchill, M. and Stark, D. J. and Matthews, G. and Rawlinson, W. D. and Kelleher, A. D. and Turville, S. G. | SARS-CoV-2 Omicron BA.5: Evolving tropism and evasion of potent humoral responses and resistance to clinical immunotherapeutics relative to viral variants of concern | 2022 | Wrong outcome |
| Aggarwal, R. and Agrawal, A. and Gurtoo, A. and Suman, V. and Meena, S. and Prakash, A. | High Mortality Unaffected by Age, Gender and Steroid Use is the Hallmark of COVID-19 in Diabetes: Observations from a Retrospective Analysis during Peak of 2020 Pandemic in India | 2021 | Wrong population |
| Agrawal, Narendra and Singh, Reema and Sharma, Sanjeev Kumar and Naithani, Rahul and Bhargava, Rahul and Choudhary, Dharma and Jeyaraman, Preethi and Bansal, Sachin and Doval, Divya and Kh and elwal, Vipin | Outcomes of COVID-19 in Hematopoietic Stem Cell Transplant Recipients: Multicenter Retrospective Analysis | 2022 | n<50 |
| Aguolu, O. G. and Malik, A. A. and Ahmed, N. and Omer, S. B. | Overcoming Vaccine Hesitancy for Future COVID-19 and HIV Vaccines: Lessons from Measles and HPV Vaccines | 2022 | Wrong population |
| Ahanchian, H. and Moazzen, N. and Saeidinia, A. and Joghatayi, S. H. and Khoshkhui, M. and Aelami, M. H. and Haghi, N. S. M. and Rezaei, N. | Death Due to COVID-19 in an Infant with Combined Immunodeficiencies | 2021 | Paediatric data |
| Ahearn, A. J. and Maw, T. T. and Mehta, R. and Emamaullee, J. and Kim, J. and Blodget, E. and Kahn, J. and Sher, L. and Genyk, Y. | A Programmatic Response, Including Bamlanivimab or Casirivimab-Imdevimab Administration, Reduces Hospitalization and Death in COVID-19 Positive Abdominal Transplant Recipients | 2022 | Wrong population |
| Ahmed, N. S. and Nega, S. S. and Deyessa, N. and Gebremariam, T. H. and Ahmed, H. Y. and Etissa, E. K. and Huluka, D. K. | Characteristics and outcomes of COVID-19 among people living with HIV at Eka Kotebe General Hospital, Addis Ababa, Ethiopia | 2022 | Wrong population |
| Ahmed, Sakir and Gasparyan, Armen Yuri and Zimba, Olena | Comorbidities in rheumatic diseases need special consideration during the COVID-19 pandemic | 2021 | Wrong outcome |
| Ajami, M. and Nazari, M. and Mahmoodzadeh, H. and Moazzeni, S. M. | Recombinant CD137-Fc, its synthesis, and applications for improving the immune system functions, such as tumor immunotherapy and to reduce the inflammation due to the novel coronavirus | 2021 | Wrong population |
| Akama-Garren, E. H. and Li, J. X. | Prior immunosuppressive therapy is associated with mortality in COVID-19 patients: A retrospective study of 835 patients | 2021 | Single centre |
| Akelew, Y. and Andualem, H. and Ebrahim, E. and Atnaf, A. and Hailemichael, W. | Immunomodulation of COVID-19 severity by helminth co-infection: Implications for COVID-19 vaccine efficacy | 2022 | Incorrect study design |
| Akhtar, H. and Khalid, S. and Rahman, F. U. and Ali, S. and Afridi, M. and Khader, Y. S. and Hassan, F. and Akhtar, N. and Khan, M. M. and Ikram, A. | Delayed admissions and efficacy of steroid use in patients with critical and severe COVID-19: an apprehensive approach | 2021 | Indirect mortality |
| Akyala, A. I. and Iwu, C. J. | Novel severe acute respiratory syndrome coronavirus 2 (SARS-CoV-2) co-infection with HIV: clinical case series analysis in North Central Nigeria | 2020 | n<50 |
| Al Azzi, Y. and Loarte, P. and Pynadath, C. and Liriano, L. and Ajaimy, M. and Kapoor, S. and Yaffe, H. and Le, M. and Graham, J. and Rocca, J. and Greenstein, S. and Kinkhabwala, M. and Akalin, E. | Decreased Mortality from Sars-CoV-2 Infection in Kidney Transplant Recipients Over the Course of the Pandemic | 2022 | Duplicate |
| Al Azzi, Y. and Pynadath, C. and Loarte, P. and Alani, O. and Liriano-Ward, L. and Ajaimy, M. and Bartash, R. and Graham, J. and Le, M. and Yaffe, H. and Greenstein, S. and Rocca, J. and Kinkhabwala, M. and Akalin, E. | Variation of mortality from sars-cov-2 infection in kidney transplant recipients over the course of the pandemic | 2021 | Single centre |
| Alakeel, Y. S. and Alharbi, E. F. and Alhaidal, H. A. and Jumaa, A. M. and Albaiahy, L. K. and Alsagami, N. S. and Alshahrani, S. A. | The effects of the antecedent use of inhaled steroid on the clinical course of COVID-19: A retrospective study of asthmatic patients | 2022 | Immunosuppression as treatment |
| Alasfar, Sami and Avery, Robin K | The impact of COVID-19 on kidney transplantation | 2020 | Indirect mortality |
| Alberca, Ricardo Wesley and Alberca, Gabriela Gama Freire and Netto, Lucas Chaves and Orfali, Raquel LeÃ£o and Gozzi-Silva, Sarah Cristina and da Silva Duarte, Alberto JosÃ© and Aoki, Valeria and Sato, Maria Notomi and Benard, Gil | COVID-19 Severity and Mortality in Solid Organ Transplantation: Differences between Liver, Heart, and Kidney Recipients | 2021 | Single centre |
| Aldea, D. and Martinez, T. and Miller, S. and Miklin, D. and Depasquale, E. and Vaidya, A. and Wolfson, A. | Transplant Recipient Characteristics in COVID vs Non COVID Cause of Death | 2022 | Insufficient original data |
| Alfishawy, M. and Elbendary, A. and Mohamed, M. and Nassar, M. | COVID-19 Mortality in Transplant Recipients | 2020 | Incorrect study design |
| Al-Hajeri, H. and Baroun, F. and Abutiban, F. and Al-Mutairi, M. and Ali, Y. and Alawadhi, A. and Albasri, A. and Aldei, A. and AlEnizi, A. and Alhadhood, N. and Al-Herz, A. and Alkadi, A. and Alk and eri, W. and Almathkoori, A. and Almutairi, N. and Alsayegh, S. and Alturki, A. and Bahbahani, H. and Dehrab, A. and Ghanem, A. and Haji Hasan, E. and Hayat, S. and Saleh, K. and Tarakmeh, H. | Therapeutic role of immunomodulators during the COVID-19 pandemic- a narrative review | 2022 | Immunosuppression as treatment |
| Ali Malekhosseini, S. and Nikoupour, H. and Gholami, S. and Shamsaeefar, A. and Arasteh, P. and Kazemi, K. and Dehghani, M. and Eghlimi, H. and Raeisi Shahraki, H. and Roozbeh, J. and Rezaianzadeh, A. and Nikeghbalian, S. | A Report of 85 Cases of COVID-19 and Abdominal Transplantation From a Single Center: What Are the Associated Factors With Death Among Organ Transplantation Patients | 2021 | Single centre |
| Ali, Tariq and Al-Ali, Ali and Fajji, Layal and Hammad, Ehab and Nazmi, Ahmed and Alahmadi, Ibrahim and Aleid, Hassan and Ullah, Asad and Shah, Yaser and Broering, Dieter | Coronavirus disease-19: disease severity and outcomes of solid organ transplant recipients: different spectrums of disease in different populations? | 2021 | Single centre |
| Alunno, A. and Najm, A. and Machado, P. M. and Bertheussen, H. and Burmester, G. R. and Carubbi, F. and De Marco, G. and Giacomelli, R. and Hermine, O. and Isaacs, J. D. and KonÃ©-Paut, I. and Magro-Checa, C. and McInnes, I. and Meroni, P. L. and Quartuccio, L. and Ramanan, A. V. and Ramos-Casals, M. and RodrÃ­guez Carrio, J. and Schulze-Koops, H. and Stamm, T. A. and Tas, S. W. and Terrier, B. and McGonagle, D. G. and Mariette, X. | EULAR points to consider on pathophysiology and use of immunomodulatory therapies in COVID-19 | 2021 | Immunosuppression as treatment |
| Alunno, A. and Najm, A. and Mariette, X. and De Marco, G. and Emmel, J. and Mason, L. and McGonagle, D. G. and Machado, P. M. | Immunomodulatory therapies for SARS-CoV-2 infection: a systematic literature review to inform EULAR points to consider | 2021 | Immunosuppression as treatment |
| Amrouche, T. and Chikindas, M. L. | Probiotics for immunomodulation in prevention against respiratory viral infections with special emphasis on COVID-19 | 2022 | Wrong population |
| Anaya-Albinagorta, A. V. and Balcazar-Aniceto, K. X. and Murga-Mogollon, C. | Mortality in patients with kidney transplantation and SARS-CoV-2 infection. [Spanish] | 2022 | Insufficient original data |
| Anggraeni, A. T. and Soedarsono, S. and Soeprijanto, B. | Concurrent COVID-19 and Pneumocystis jirovecii pneumonia: The importance of radiological diagnostic and HIV testing | 2021 | Incorrect study design |
| Anikhindi, S. A. and Kumar, A. and Arora, A. | COVID-19 in patients with inflammatory bowel disease | 2020 | Insufficient original data |
| Anwar, M. M. | Immunotherapies and COVID-19 related Neurological manifestations: A Comprehensive Review Article | 2020 | Wrong population |
| Arazi, H. and Falahati, A. and Suzuki, K. | Moderate Intensity Aerobic Exercise Potential Favorable Effect Against COVID-19: The Role of Renin-Angiotensin System and Immunomodulatory Effects | 2021 | Wrong outcome |
| Artigas, C. and Lemort, M. and Mestrez, F. and Gil, T. and Flamen, P. | COVID-19 Pneumonia Mimicking Immunotherapy-Induced Pneumonitis on 18F-FDG PET/CT in a Patient Under Treatment With Nivolumab | 2020 | Incorrect study design |
| Ashare, R. L. and Bernstein, S. L. and Schnoll, R. and Gross, R. and Catz, S. L. and Cioe, P. and Crothers, K. and Hitsman, B. and Marhefka, S. L. and McClure, J. B. and Pacek, L. R. and Vidrine, D. J. and Vilardaga, R. and Kaufman, A. and Edelman, E. J. | The United States National Cancer Institute's Coordinated Research Effort on Tobacco Use as a Major Cause of Morbidity and Mortality among People with HIV | 2021 | Wrong population |
| Athale, J. and Gallagher, J. and Busch, L. M. | Management of Severe and Critical COVID-19 Infection with Immunotherapies | 2022 | Wrong population |
| Azoulay, Elie and Fartoukh, Muriel and Darmon, Michael and Geri, Guillaume and Voiriot, Guillaume and Dupont, Thibault and Zafrani, Lara and Girodias, Lola and Labbe, Vincent and Dres, Martin | Increased mortality in patients with severe SARS-CoV-2 infection admitted within seven days of disease onset | 2020 | Wrong population |
| Bacca, E. and Digaetano, M. and Meschiari, M. and Franceschini, E. and Menozzi, M. and Cuomo, G. and Mussini, C. | Immunomodulation for the management of severe SARS-CoV2 infections. State of the art and review of the literature | 2021 | Immunosuppression as treatment |
| Bachanas, P. J. and Chun, H. M. and Mehta, N. and Aberle-Grasse, J. and Parris, K. and Sherlock, M. W. and Lloyd, S. and Zeh, C. and Makwepa, D. K. and Kap and a, M. L. and Dokubo, E. K. and Bonono, L. and Balach and ra, S. and Ehui, E. and Fonjungo, P. and Nkoso, A. M. and Mazibuko, S. and Okello, V. N. and Tefera, F. and Getachew, M. and Katiku, E. M. and Mulwa, A. and Asiimwe, F. M. and Tarumbiswa, T. F. and Auld, A. F. and Nyirenda, R. and Dos Santos De Louvado, A. P. and Gaspar, I. and Hong, S. Y. and Ashipala, L. and Obanubi, C. and Ikpeazu, A. and Musoni, C. and Yoboka, E. and Mthethwa, S. and Pinini, Z. and Bunga, S. and Rumunu, J. and Magesa, D. J. and Mutayoba, B. and Nelson, L. J. and Katureebe, C. and Agolory, S. and Mulenga, L. B. and Nyika, P. and Mugurungi, O. and Ellerbrock, T. and Mitruka, K. | Protecting the gains: analysis of HIV treatment and service delivery programme data and interventions implemented in 19 African countries during COVID-19 | 2022 | Wrong outcome |
| Bachar, S. C. and Mazumder, K. and Bachar, R. and Aktar, A. and Al Mahtab, M. | A Review of Medicinal Plants with Antiviral Activity Available in Bangladesh and Mechanistic Insight Into Their Bioactive Metabolites on SARS-CoV-2, HIV and HBV | 2021 | Wrong population |
| Badawy, A. A. | Immunotherapy of COVID-19 with poly (ADP-ribose) polymerase inhibitors: starting with nicotinamide | 2020 | Wrong population |
| Bagri, N. K. and Khan, M. and P and ey, R. M. and Lodha, R. and Kabra, S. K. | Initial Immunomodulation and Outcome of Children with Multisystem Inflammatory Syndrome Related to COVID-19: A Multisite Study from India | 2022 | Wrong population |
| Bakouny, Z. and Labaki, C. and Grover, P. and Awosika, J. and Gulati, S. and Hsu, C. Y. and Alimohamed, S. I. and Bashir, B. and Berg, S. and Bilen, M. A. and Bowles, D. and Castellano, C. and Desai, A. and Elkrief, A. and Eton, O. E. and Fecher, L. A. and Flora, D. and Galsky, M. D. and Gatti-Mays, M. E. and Gesenhues, A. and Glover, M. J. and Gopalakrishnan, D. and Gupta, S. and Halfdanarson, T. R. and Hayes-Lattin, B. and Hendawi, M. and Hsu, E. and Hwang, C. and J and arov, R. and Jani, C. and Johnson, D. B. and Joshi, M. and Khan, H. and Khan, S. A. and Knox, N. and Koshkin, V. S. and Kulkarni, A. A. and Kwon, D. H. and Matar, S. and McKay, R. R. and Mishra, S. and Moria, F. A. and Nizam, A. and Nock, N. L. and Nonato, T. K. and Panasci, J. and Pomerantz, L. and Portuguese, A. J. and Provenzano, D. and Puc, M. and Rao, Y. J. and Rhodes, T. D. and Riely, G. J. and Ripp, J. J. and Rivera, A. V. and Ruiz-Garcia, E. and Schmidt, A. L. and Schoenfeld, A. J. and Schwartz, G. K. and Shah, S. A. and Shaya, J. and Subbiah, S. and Tachiki, L. M. and Tucker, M. D. and Valdez-Reyes, M. and Weissmann, L. B. and Wotman, M. T. and Wulff-Burchfield, E. M. and Xie, Z. and Yang, Y. J. and Thompson, M. A. and Shah, D. P. and Warner, J. L. and Shyr, Y. and Choueiri, T. K. and Wise-Draper, T. M. | Interplay of Immunosuppression and Immunotherapy Among Patients With Cancer and COVID-19 | 2022 | Wrong outcome |
| Banerjee, Amitava and Pasea, Laura and Harris, Steve and Gonzalez-Izquierdo, Arturo and Torralbo, Ana and Shallcross, Laura and Noursadeghi, Mahdad and Pillay, Deenan and Pagel, Christina and Wong, Wai Keong | Estimating excess 1-year mortality from COVID-19 according to underlying conditions and age in England: a rapid analysis using NHS health records in 3.8 million adults | 2020 | Wrong outcome |
| Banerjee, Amitava and Pasea, Laura and Harris, Steve and Gonzalez-Izquierdo, Arturo and Torralbo, Ana and Shallcross, Laura and Noursadeghi, Mahdad and Pillay, Deenan and Sebire, Neil and Holmes, Chris | Estimating excess 1-year mortality associated with the COVID-19 pandemic according to underlying conditions and age: a population-based cohort study | 2020 | Wrong outcome |
| Barat, M. and Duran, J. M. and Sung, K. and Brown, M. and Lin, A. Y. and King, K. R. and Adler, E. D. and Aslam, S. | Breakthrough Infections and Low Mortality Observed in Heart Transplant Recipients Infected with COVID-19 at UC San Diego | 2022 | n<50 |
| Barrera-LÃ³pez, P. and PÃ©rez-Riveros, E. D. and Moreno-Montoya, J. and Ballesteros, S. M. and Valencia, S. A. and De la Hoz-Valle, J. A. | Coinfection of other respiratory pathogens and HIV in COVID-19 patients: Is there a pattern? | 2021 | Insufficient original data |
| Basic-Jukic, N. and Racki, S. and Tolj, I. and Aleckovic, M. and Babovic, B. and Juric, I. and Furic-Cunko, V. and Katalinic, L. and Mihaljevic, D. and Vujic, S. and Mesic, E. and Jelakovic, B. and Kastelan, Z. | Hospitalization and death after recovery from acute COVID-19 among renal transplant recipients | 2022 | Duplicate |
| Basu-Ray, I. and Metri, K. and Khanra, D. and Revankar, R. and Chinnaiyan, K. M. and Raghuram, N. and Mishra, M. C. and Patwardhan, B. and Sharma, M. and Basavaraddi, I. V. and An and , A. and Reddy, S. and Deepak, K. K. and Levy, M. and Theus, S. and Levine, G. N. and Cramer, H. and Fricchione, G. L. and Hongas and ra, N. R. | A narrative review on yoga: a potential intervention for augmenting immunomodulation and mental health in COVID-19 | 2022 | Wrong population |
| BatÄ±rel, A. and Demirhan, R. and Eser, N. and KÃ¶rlÃ¼, E. and Tezcan, M. E. | Pulse steroid treatment for hospitalized adults with COVID-19 | 2021 | Incorrect study design |
| Bazgir, N. and Taghinezhad, F. and Nourmohammadi, H. and Azami, G. and Ahmadi, I. and Mozafari, A. | Comparing the COVID-19 Mortality Rate in Cancer Patients with and Without a History of Chemotherapy | 2022 | Single centre |
| Beaney, Thomas and Clarke, Jonathan M and Jain, Vageesh and Golestaneh, Amelia Kataria and Lyons, Gemma and Salman, David and Majeed, Azeem | Excess mortality: the gold standard in measuring the impact of COVID-19 worldwide? | 2020 | Insufficient original data |
| Bell, D. and Schultz Hansen, K. | Relative Burdens of the COVID-19, Malaria, Tuberculosis, and HIV/AIDS Epidemics in Sub-Saharan Africa | 2021 | Wrong population |
| Bell, N. and Bracchi, M. and Dalla Pria, A. and Nelson, M. and Boffito, M. | Indirect Human Immunodeficiency Virus Morbidity and Mortality Due to Coronavirus Disease 2019 | 2021 | Wrong outcome |
| Bell, N. and Longley, J. and Falconer, J. and Sutcliffe, L. and Dalla Pria, A. and Garvey, L. and Nelson, M. and Boffito, M. and Bracchi, M. | The indirect effects of COVID-19 on the morbidity and mortality of people living with HIV | 2021 | Single centre |
| Bell, Samira and Campbell, Jacqueline and McDonald, Jackie and Oâ€™Neill, Martin and Watters, Chrissie and Buck, Katharine and Cousl and , Zoe and Findlay, Mark and Lone, Nazir I and Metcalfe, Wendy | COVID-19 in patients undergoing chronic kidney replacement therapy and kidney transplant recipients in Scotland: findings and experience from the Scottish renal registry | 2020 | n<50 |
| Belluomini, L. and Caldart, A. and Avancini, A. and Dodi, A. and Trestini, I. and Kadrija, D. and Sposito, M. and Tregnago, D. and Casali, M. and Riva, S. T. and Sartori, G. and Menis, J. and Milella, M. and Pilotto, S. | Infections and Immunotherapy in Lung Cancer: A Bad Relationship? | 2020 | Incorrect study design |
| Belsky, Jennifer A and Tullius, Brian P and Lamb, Margaret G and Sayegh, Rouba and Stanek, Joseph R and Auletta, Jeffery J | COVID-19 in immunocompromised patients: a systematic review of cancer, hematopoietic cell and solid organ transplant patients | 2021 | Paediatric data |
| Ben Moftah, M. and Eswayah, A. | Repurposing of Hydroxyurea Against COVID-19: A Promising Immunomodulatory Role | 2022 | Wrong population |
| Benade, M. and Long, L. and Rosen, S. and Meyer-Rath, G. and Tucker, J. M. and Miot, J. | Reduction in initiations of HIV treatment in South Africa during the COVID pandemic | 2022 | Indirect mortality |
| Benchimol, E. I. and Carroll, M. W. and Geist, R. and Griffiths, A. M. and Huang, J. G. and Mack, D. R. and Bernstein, C. N. and Bitton, A. and Jones, J. L. and Kaplan, G. G. and Kuenzig, M. E. and Lee, K. and Mukhtar, M. S. and Murthy, S. K. and T and on, P. and Targownik, L. E. and Windsor, J. W. and Seow, C. H. | Crohn's and Colitis Canada's 2021 Impact of COVID-19 and Inflammatory Bowel Disease in Canada: Children and Expectant Mothers With Inflammatory Bowel Disease | 2021 | Wrong population |
| Benotmane, I. and Perrin, P. and Gautier Vargas, G. and Bass and , X. and Keller, N. and Lavaux, T. and Ohana, M. and Bedo, D. and Baldacini, C. and Sagnard, M. and Bozman, D. F. and Della Chiesa, M. and Cognard, N. and Olagne, J. and Delagreverie, H. and Marx, D. and Heibel, F. and Braun, L. and Moulin, B. and Fafi Kremer, S. and Caillard, S. | Biomarkers of Cytokine Release Syndrome Predict Disease Severity and Mortality From COVID-19 in Kidney Transplant Recipients | 2020 | n<50 |
| Berger, B. and Hazzan, M. and Kamar, N. and Francois, H. and Matignon, M. and Greze, C. and Gatault, P. and Frimat, L. and Westeel, P. F. and Goutaudier, V. and Snanoudj, R. and Colosio, C. and Sicard, A. and Bertr and , D. and Mousson, C. and Bamoulid, J. and Thierry, A. and Anglicheau, D. and Couzi, L. and Chemouny, J. M. and Duveau, A. and Moal, V. and Le Meur, Y. and Blancho, G. and Tourret, J. and Malvezzi, P. and Mariat, C. and Rerolle, J. P. and Bouvier, N. and Caillard, S. and Thaunat, O. | Changing of therapeutic trends between the 1st and 2nd wave did not reduce COVID-19 related mortality of renal transplant recipients: A national registry study | 2021 | Duplicate |
| Bernini, R. and Velotti, F. | Natural Polyphenols as Immunomodulators to Rescue Immune Response Homeostasis: Quercetin as a Research Model against Severe COVID-19 | 2021 | Immunosuppression as treatment |
| Bernstein, C. N. and Singh, H. and Murthy, S. K. and Nguyen, G. C. and Benchimol, E. I. and Bitton, A. and Kuenzig, M. E. and Huang, J. G. and Jones, J. L. and Lee, K. and Targownik, L. E. and Windsor, J. W. and Mukhtar, M. S. and T and on, P. and Kaplan, G. G. | Crohn's and Colitis Canada's 2021 Impact of COVID-19 and Inflammatory Bowel Disease in Canada: Seniors With IBD | 2021 | Duplicate |
| Bertini, Christopher D and Khawaja, Fareed and Sheshadri, Ajay | COVID-19 in the Immunocompromised Host | 2022 | Duplicate |
| Bhargava, Ashish and Szpunar, Susanna M and Sharma, Mamta and Fukushima, Elisa Akagi and Hoshi, Sami and Levine, Miriam and G and hi, Nikhil and Zhao, Wei and Michael, Somero and Tanveer, Farah | Clinical features and risk factors for in-hospital mortality from COVID-19 infection at a tertiary care medical center, at the onset of the US COVID-19 pandemic | 2021 | Wrong outcome |
| Bhatti, A. B. H. and Riyaz, S. and Akhtar, A. | In-hospital Mortality after Liver Transplantation due to COVID-19 | 2020 | Incorrect study design |
| BobircÄƒ, A. and BobircÄƒ, F. and AncuÈ›a, I. and Florescu, A. and BojincÄƒ, M. and MuscÄƒ, A. and Florescu, D. N. and Florescu, L. M. and Sima, R. M. and Florescu, A. and MuÈ™etescu, A. E. | COVID-19-A Trigger Factor for Severe Immune-Mediated Thrombocytopenia in Active Rheumatoid Arthritis | 2022 | Wrong outcome |
| Boffito, Marta and Waters, Laura | More evidence for worse COVID-19 outcomes in people with HIV | 2021 | Insufficient original data |
| Bongomin, F. and Sereke, S. G. and Okot, J. and Katsigazi, R. and K and ole, T. K. and Oriekot, A. and Olum, R. and Atukunda, A. and Baluku, J. B. and Nakwagala, F. | COVID-19, HIV-Associated Cryptococcal Meningitis, Disseminated Tuberculosis and Acute Ischaemic Stroke: A Fatal Foursome | 2021 | Incorrect study design |
| Bonuomo, V. and Ferrarini, I. and Dell'Eva, M. and SbisÃ , E. and Krampera, M. and Visco, C. | COVID-19 (SARS-CoV-2 infection) in lymphoma patients: A review | 2021 | Incorrect study design |
| Booton, R. D. and Fu, G. and MacGregor, L. and Li, J. and Ong, J. J. and Tucker, J. D. and Turner, K. M. and Tang, W. and Vickerman, P. and Mitchell, K. M. | The impact of disruptions due to COVID-19 on HIV transmission and control among men who have sex with men in China | 2021 | Wrong outcome |
| Booton, R. D. and Fu, G. and MacGregor, L. and Li, J. and Ong, J. J. and Tucker, J. D. and Turner, K. M. E. and Tang, W. and Vickerman, P. and Mitchell, K. M. | Estimating the impact of disruptions due to COVID-19 on HIV transmission and control among men who have sex with men in China | 2020 | Wrong outcome |
| Boppana, T. K. and Mittal, S. and Madan, K. and Mohan, A. and Hadda, V. and Tiwari, P. and Guleria, R. | Steroid therapy for COVID-19: A systematic review and meta-analysis of randomized controlled trials | 2021 | Wrong population |
| Borin, A. and Coimbra, L. D. and Bispo-Dos-Santos, K. and Naciuk, F. F. and Fontoura, M. and Simeoni, C. L. and Gomes, G. V. and Amorim, M. R. and Gravina, H. D. and Shimizu, J. F. and Passos, A. S. C. and de Oliveira, I. M. and de Carvalho, A. C. and Cardoso, A. C. and Parise, P. L. and Toledo-Teixeira, D. A. and Sotorilli, G. E. and Persinoti, G. F. and Claro, I. M. and Sabino, E. C. and Alborghetti, M. R. and Rocco, S. A. and Franchini, K. G. and de Souza, W. M. and Oliveira, P. S. L. and Cunha, T. M. and Granja, F. and ProenÃ§a-MÃ³dena, J. L. and Trivella, D. B. B. and Bruder, M. and Cordeiro, A. T. and Marques, R. E. | Identification and characterization of the anti-SARS-CoV-2 activity of cationic amphiphilic steroidal compounds | 2022 | Wrong outcome |
| Borshoff, D. C. and Sadleir, P. | Nonoperating room anaesthesia: safety, monitoring, cognitive aids and severe acute respiratory syndrome coronavirus 2 | 2020 | Wrong outcome |
| Bosworth, Matthew L and Ayoubkhani, Daniel and Nafilyan, VahÃ© and Foubert, Josephine and Glickman, Myer and Davey, Calum and Kuper, Hannah | Deaths involving COVID-19 by disability status: a retrospective analysis of 29 million adults during the first two waves of the Coronavirus pandemic in England | 2021 | Wrong population |
| Bouleftour, W. and Bonjean, P. and Grangeon, K. and MagnÃ©, N. | COVID-19 Vaccine-Related Adverse Events in Solid Cancer Patients Treated with Immunotherapy | 2022 | Wrong population |
| Brannigan, L and Botha, J | An argument for a rational and balanced risk approach to transplantation during the COVID-19 pandemic | 2020 | Insufficient original data |
| Brown, A. E. and Croxford, S. E. and Nash, S. and Khawam, J. and Kirwan, P. and Kall, M. and Bradshaw, D. and Sabin, C. and Miller, R. F. and Post, F. A. and Harding, R. and Collins, S. and Waters, L. and Asboe, D. and Chadwick, D. R. and Delpech, V. and Sullivan, A. K. | COVID-19 mortality among people with diagnosed HIV compared to those without during the first wave of the COVID-19 pandemic in England | 2022 | Single centre |
| Brown, L. B. and Spinelli, M. A. and G and hi, M. | The interplay between HIV and COVID-19: summary of the data and responses to date | 2021 | Insufficient original data |
| Brownstone, N. D. and Thibodeaux, Q. G. and Reddy, V. D. and Myers, B. A. and Chan, S. Y. and Bhutani, T. and Liao, W. | Novel Coronavirus Disease (COVID-19) and Biologic Therapy in Psoriasis: Infection Risk and Patient Counseling in Uncertain Times | 2020 | Wrong outcome |
| Bruce, E. and Barlow-Pay, F. and Short, R. and Vilches-Moraga, A. and Price, A. and McGovern, A. and Braude, P. and Stechman, M. J. and Moug, S. and McCarthy, K. and Hewitt, J. and Carter, B. and Myint, P. K. | Prior Routine Use of Non-Steroidal Anti-Inflammatory Drugs (NSAIDs) and Important Outcomes in Hospitalised Patients with COVID-19 | 2020 | Wrong population |
| Bsteh, G. and Assar, H. and Hegen, H. and Heschl, B. and Leutmezer, F. and Di Pauli, F. and Gradl, C. and Traxler, G. and Zulehner, G. and Rommer, P. and Wipfler, P. and Guger, M. and Enzinger, C. and Berger, T. | COVID-19 severity and mortality in multiple sclerosis are not associated with immunotherapy: Insights from a nation-wide Austrian registry | 2021 | Wrong outcome |
| Buchbinder, S. and Liu, A. | CROI 2021: Epidemiologic Trends in the HIV and SARS-CoV-2 Pandemics and HIV Prevention Research | 2021 | Wrong outcome |
| Bukhari, M. | A deeper dive into rare autoimmune diseases, death and COVID-19 in the first wave of the pandemic | 2021 | Incorrect study design |
| Bulbuloglu, S. and Kapikiran, G. | The effect of immunosuppressive therapy after liver transplantation on activities of daily living and fear of death during the COVID-19 pandemic | 2021 | Wrong outcome |
| Bulut, Ã– and GÃœrsel, Ä° | Mesenchymal stem cell derived extracellular vesicles: promising immunomodulators against autoimmune, autoinflammatory disorders and SARS-CoV-2 infection | 2020 | Wrong population |
| Burns, S. M. and Woodworth, T. S. and Icten, Z. and Honda, T. and Manjourides, J. | A Machine Learning Approach to Identify Predictors of Severe COVID-19 Outcome in Patients With Rheumatoid Arthritis | 2022 | Wrong outcome |
| Calis, H. and Karabulut, Z. and Guler, Y. and Sengul, S. | Idiopathic granulomatous mastitis and steroid use during the pandemic of COVID-19 | 2022 | Wrong outcome |
| Camargo, Jose F and Mendoza, Maria A and Lin, Rick and Moroz, Ilona V and Anderson, Anthony D and Morris, Michelle I and Natori, Yoichiro and Natori, Akina and Raja, Mohammed and Lekakis, Lazaros | Clinical presentation and outcomes of COVID-19 following hematopoietic cell transplantation and cellular therapy | 2021 | Single centre |
| Campbell, H. M. and Murata, A. E. and Conner, T. A. and Fotieo, G. | Chronic use of non-steroidal anti-inflammatory drugs (NSAIDs) or acetaminophen and relationship with mortality among United States Veterans after testing positive for COVID-19 | 2022 | Wrong population |
| Cantarelli, Chiara and Angeletti, Andrea and Perin, Laura and Russo, Luis Sanchez and Sabiu, Gianmarco and PodestÃ , Manuel Alfredo and Cravedi, Paolo | Immune responses to SARS-CoV-2 in dialysis and kidney transplantation | 2022 | Wrong outcome |
| Cassin, R. and Rampi, N. and C, F. and Muscatello, A. and Mariani, B. and Noto, A. and Rossi, F. G. and Baldini, L. | Reply to "successful early use of anti-SARS-CoV-2 monoclonal neutralizing antibodies in SARS-CoV-2 infected hematological patients-A Czech multicenter experience": A case series of SARS-CoV-2 Omicron infection and aggressive lymphoma in the Sotrovimab era | 2022 | Incorrect study design |
| Ceballos, M. E. and Ross, P. and Lasso, M. and Dominguez, I. and Puente, M. and Valenzuela, P. and Enberg, M. and Serri, M. and MuÃ±oz, R. and Pinos, Y. and Silva, M. and Noguera, M. and Dominguez, A. and Zamora, F. | Clinical characteristics and outcomes of people living with HIV hospitalized with COVID-19: a nationwide experience | 2021 | n<50 |
| Cesaro, S. and Ljungman, P. and Mikulska, M. and Hirsch, H. H. and von Lilienfeld-Toal, M. and Cordonnier, C. and Meylan, S. and Mehra, V. and Styczynski, J. and Marchesi, F. and Besson, C. and Baldanti, F. and Masculano, R. C. and Beutel, G. and Einsele, H. and Azoulay, E. and Maertens, J. and de la Camara, R. and Pagano, L. | Recommendations for the management of COVID-19 in patients with haematological malignancies or haematopoietic cell transplantation, from the 2021 European Conference on Infections in Leukaemia (ECIL 9) | 2022 | Insufficient original data |
| Ch and a, D. and Minchella, P. A. and Kampamba, D. and Itoh, M. and Hines, J. Z. and Fwoloshi, S. and Boyd, M. A. and Hamusonde, K. and Chirwa, L. and Nikoi, K. and Chirwa, R. and Siwingwa, M. and Sivile, S. and Zyambo, K. D. and Mweemba, A. and Mbewe, N. and Mutengo, K. H. and Malama, K. and Agolory, S. and Mulenga, L. B. | COVID-19 Severity and COVID-19-Associated Deaths Among Hospitalized Patients with HIV Infection - Zambia, March-December 2020 | 2021 | Paediatric data |
| Chakravarthy, K. and Str and , N. and Frosch, A. and Sayed, D. and Narra, L. R. and Chaturvedi, R. and Grewal, P. K. and Pope, J. and Schatman, M. E. and Deer, T. | Recommendations and Guidance for Steroid Injection Therapy and COVID-19 Vaccine Administration from the American Society of Pain and Neuroscience (ASPN) | 2021 | Insufficient original data |
| Chambers, C. and Samji, H. and Cooper, C. L. and Costiniuk, C. T. and Janjua, N. Z. and Kroch, A. E. and Arbess, G. and Benoit, A. C. and Buchan, S. A. and Chung, H. and Kendall, C. E. and Kwong, J. C. and Langlois, M. A. and Lee, S. M. and Mbuagbaw, L. and McCullagh, J. and Moineddin, R. and Nambiar, D. and Walmsley, S. and Anis, A. H. and Burchell, A. N. | Coronavirus disease 2019 vaccine effectiveness among a population-based cohort of people living with HIV | 2022 | Wrong outcome |
| Chastain, D. B. and Stitt, T. M. and Ly, P. T. and Henao-MartÃ­nez, A. F. and Franco-Paredes, C. and Osae, S. P. | Countermeasures to Coronavirus Disease 2019: Are Immunomodulators Rational Treatment Options-A Critical Review of the Evidence | 2020 | Wrong population |
| Chatterjee, K. and Wu, C. P. and Bhardwaj, A. and Siuba, M. | Steroids in COVID-19: An overview | 2020 | Wrong population |
| Chedid, Georges and Abdessamad, Hilal and Costanian, Christy and Maamari, Julian and Al Nakib, Mostafa and Maatook, Ismael and Farra, Anna and Hussni, Roula and Mokhbat, Jacques | COVID-19 in Persons Living With HIV: A Cross-Sectional Study From Lebanon | 2020 | Insufficient original data |
| Chen, L. and Hu, K. and Cheng, C. and Hu, Q. and Zhang, L. and An, T. and Guo, Y. and Chen, S. and Duan, G. | Risk of adverse outcomes in inflammatory bowel disease patients infected with SARS-CoV-2: a systematic review and meta-analysis | 2022 | Paediatric data |
| Chen, X. and Guo, H. and Qiu, L. and Zhang, C. and Deng, Q. and Leng, Q. | Immunomodulatory and Antiviral Activity of Metformin and Its Potential Implications in Treating Coronavirus Disease 2019 and Lung Injury | 2020 | Wrong population |
| Cheng, J. and Tao, J. and Li, B. and Shi, Y. and Liu, H. | Coinfection with PEDV and BVDV induces inflammatory bowel disease pathway highly enriched in PK-15 cells | 2022 | Wrong population |
| Chenneville, T. and Gabbidon, K. and Hanson, P. and Holyfield, C. | The Impact of COVID-19 on HIV Treatment and Research: A Call to Action | 2020 | Insufficient original data |
| Chiappelli, F. and Khakshooy, A. and Greenberg, G. | CoViD-19 Immunopathology and Immunotherapy | 2020 | Insufficient original data |
| Chilot, D. and Woldeamanuel, Y. and Manyazewal, T. | COVID-19 Burden on HIV Patients Attending Antiretroviral Therapy in Addis Ababa, Ethiopia: A Multicenter Cross-Sectional Study | 2022 | Wrong outcome |
| Choudhary, D. and Kenwar, D. and Sharma, A. and Bhalla, A. and Singh, S. and Singh, M. P. and Kumar, V. and Sharma, A. | Risk factors for mortality in kidney transplant recipients with COVID-19: a single centre experience and case-control study | 2022 | Single centre |
| Choudhury, A. and Mukherjee, G. and Mukherjee, S. | Chemotherapy vs. Immunotherapy in combating nCOVID19: An update | 2021 | Wrong population |
| Christie, M. J. and Irving, A. T. and Forster, S. C. and Marsl and , B. J. and Hansbro, P. M. and Hertzog, P. J. and Nold-Petry, C. A. and Nold, M. F. | Of bats and men: Immunomodulatory treatment options for COVID-19 guided by the immunopathology of SARS-CoV-2 infection | 2021 | Wrong population |
| Chun, H. M. and Milligan, K. and Agyemang, E. and Ford, N. and Rangaraj, A. and Desai, S. and Wilder-Smith, A. and Vitoria, M. and Zulu, I. | A Systematic Review of COVID-19 Vaccine Antibody Responses in People With HIV | 2022 | Wrong outcome |
| Clarke, J. A. and Wiemken, T. L. and Korenblat, K. M. | Excess Mortality Among Solid Organ Transplant Recipients in the United States During the COVID-19 Pandemic | 2022 | Wrong outcome |
| Coll, Elisabeth and FernÃ¡ndezâ€Ruiz, Mario and SÃ¡nchezâ€Ãlvarez, J Emilio and MartÃ­nezâ€FernÃ¡ndez, JosÃ© R and Crespo, Marta and Gayoso, Jorge and Badaâ€Bosch, Teresa and Oppenheimer, Federico and Moreso, Francesc and LÃ³pezâ€Oliva, MarÃ­a O | COVID-19 in transplant recipients: the Spanish experience | 2021 | Paediatric data |
| Collaboration, Antiretroviral Therapy Cohort | Mortality of HIV-infected patients starting potent antiretroviral therapy: comparison with the general population in nine industrialized countries | 2009 | Wrong outcome |
| Collora, J. A. and Liu, R. and Albrecht, K. and Ho, Y. C. | The single-cell landscape of immunological responses of CD4+ T cells in HIV versus severe acute respiratory syndrome coronavirus 2 | 2021 | Wrong population |
| Conforti, Claudio and Giuffrida, Roberta and Di Meo, Nicola and Zalaudek, Iris | Management of advanced melanoma in the COVID-19 era | 2020 | Insufficient original data |
| Conforti, Claudio and Giuffrida, Roberta and Dianzani, Caterina and Di Meo, Nicola and Zalaudek, Iris | Biologic therapy for psoriasis during the COVID-19 outbreak: the choice is to weigh risks and benefits | 2020 | Insufficient original data |
| Conforti, Claudio and Giuffrida, Roberta and Dianzani, Caterina and Di Meo, Nicola and Zalaudek, Iris | COVID-19 and psoriasis: is it time to limit treatment with immunosuppressants? A call for action | 2020 | Insufficient original data |
| Constans, M. and Santiago, R. and Jimenez, L. and Motllo, C. and Lopez, R. and Trape, J. and Reverter, J. C. and Altes, A. | Lupus anticoagulant is an independent risk factor for non-thrombotic in-hospital mortality in COVID-19 patients | 2021 | Wrong population |
| Conway, Richard and Grimshaw, Alyssa A and Konig, Maximilian F and Putman, Michael and Duarteâ€GarcÃ­a, AlÃ­ and Tseng, Leslie Yingzhijie and Cabrera, Diego M and Chock, Yu Pei Eugenia and Degirmenci, Huseyin Berk and Duff, Eimear | SARSâ€“CoVâ€2 Infection and COVID-19 Outcomes in Rheumatic Diseases: A Systematic Literature Review and Metaâ€Analysis | 2022 | Paediatric data |
| Copertino, D. C., Jr. and Casado Lima, B. C. and Duarte, R. R. R. and Powell, T. R. and Ormsby, C. E. and Wilkin, T. and Gulick, R. M. and de Mulder Rougvie, M. and Nixon, D. F. | Antiretroviral drug activity and potential for pre-exposure prophylaxis against COVID-19 and HIV infection | 2022 | Wrong outcome |
| Cordtz, RenÃ© and Lindhardsen, Jesper and Soussi, Bolette G and Vela, Jonathan and Uhrenholt, Line and Westermann, Rasmus and Kristensen, Salome and Nielsen, Henrik and Torp-Pedersen, Christian and Dreyer, Lene | Incidence and severeness of COVID-19 hospitalization in patients with inflammatory rheumatic disease: a nationwide cohort study from Denmark | 2021 | n<50 |
| Couzin-Frankel, J. | Will COVID-19 change science? Past pandemics offer clues from the Black Death to AIDS, outbreaks can spur scientists to rethink how they study disease and protect public health | 2021 | Wrong outcome |
| Creemers, R. H. and Rezazadeh Ardabili, A. and Jonkers, D. M. and Leers, M. P. G. and Romberg-Camps, M. J. and Pierik, M. J. and van Bodegraven, A. A. | Severe COVID-19 in inflammatory bowel disease patients in a population-based setting | 2021 | n<50 |
| Croxford, S. and Brown, A. and Nash, S. and Khawam, J. and Kall, M. and Bradshaw, D. and Sabin, C. and Miller, R. and Post, F. and Harding, R. and Lucas, S. and Collins, S. and Waters, L. and Asboe, D. and Chadwick, D. and Delpech, V. and Sullivan, A. | COVID-19 mortality among people with HIV compared to the general population during the first wave of the epidemic in England | 2021 | Wrong outcome |
| Cutolo, M. and Smith, V. and Paolino, S. | Editorial: Understanding immune effects of oestrogens to explain the reduced morbidity and mortality in female versus male COVID-19 patients. Comparisons with autoimmunity and vaccination | 2020 | Wrong outcome |
| Dâ€™Souza, Gypsyamber and Springer, Gayle and Gustafson, Deborah and Kassaye, Seble and Alcaide, Maria L and Ramirez, Catalina and Sharma, Anjali and Palella, Frank J and Tien, Phyllis C and Detels, Roger | COVID-19 symptoms and SARS-CoV-2 infection among people living with HIV in the US: the MACS/WIHS combined cohort study | 2020 | Wrong outcome |
| DÃ­ez, C. and Del Romero-Raposo, J. and Mican, R. and LÃ³pez, J. C. and Blanco, J. R. and Calzado, S. and Samperiz, G. and Portilla, J. and GarcÃ­a-Fraile, L. J. and GutiÃ©rrez, F. and GÃ³mez-Sirvent, J. L. and SuÃ¡rez-GarcÃ­a, I. and Amador, C. and Novella, M. and Arribas, J. R. and Moreno, S. and GonzÃ¡lez-GarcÃ­a, J. and JarrÃ­n, I. and Berenguer, J. | COVID-19 in hospitalized HIV-positive and HIV-negative patients: A matched study | 2021 | n<50 |
| Dahmane, R. and Sahtout, W. and Azzabi, A. and Thabet, N. and Hadhri, A. and kallel, W. and ben aicha, N. and Mrabet, S. and Fradi, A. and boukadida, R. and Zellama, D. and fodha, I. and trabelsi, A. and Guedri, Y. and Achour, A. | Pos-789 Death in Kidney Transplant Recipients with Covid-19 Experience of Nephrology Department of Sahloul Hospital | 2022 | n<50 |
| Danwang, C. and Noubiap, J. J. and Robert, A. and Yombi, J. C. | Outcomes of patients with HIV and COVID-19 co-infection: a systematic review and meta-analysis | 2022 | Paediatric data |
| Danzigerâ€Isakov, Lara and Blumberg, Emily A and Manuel, Oriol and Sester, Martina | Impact of COVID-19 in solid organ transplant recipients | 2021 | Incorrect study design |
| Das, S. and Rastogi, A. and Harikumar, K. V. S. and Dutta, D. and Sahay, R. and Kalra, S. and Ghosh, S. and Gupta, S. K. and P and it, K. and Jabbar, P. K. and Damodaran, S. and Nagesh, V. S. and Sheikh, S. and Madhu, S. V. and Bantwal, G. | Diagnosis and Management Considerations in Steroid-Related Hyperglycemia in COVID-19: A Position Statement from the Endocrine Society of India | 2021 | Incorrect study design |
| Davidson, Jennifer and Warren-Gash, Charlotte and Mcdonald, Helen and Banerjee, Ami and Gayle, Alicia and Strongman, Helen and Evans, Dai and Clay, Simon and Forbes, Harriet and Mansfield, Kathryn E | Codelists for:" Factors associated with excess mortality in the first wave of COVID-19 pandemic in the UK: a cohort analysis using the Clinical Practice Research Databank" | 2021 | Incorrect study design |
| De Brab and er, J. and Duijvelaar, E. and Schippers, J. R. and Smeele, P. J. and Duitman, J. and Aman, J. and Bogaard, H. J. and Van Der Poll, T. and Bos, L. D. J. | Oral Imatinib Reduces Mortality in Hospitalized COVID-19 Patients Through Endothelial Barrier Protection and Immunomodulation | 2022 | Wrong population |
| de Brab and er, J. and Duijvelaar, E. and Schippers, J. R. and Smeele, P. J. and Peters-Sengers, H. and Duitman, J. W. and Aman, J. and Bogaard, H. J. and van der Poll, T. and Bos, L. D. J. | Immunomodulation and endothelial barrier protection mediate the association between oral imatinib and mortality in hospitalised COVID-19 patients | 2022 | Wrong population |
| de Lazzari, E. and MartÃ­nez-Mimbrero, A. and Chivite, I. and GonzÃ¡lez-CordÃ³n, A. and Mosquera, M. M. and Laguno, M. and Costa, J. and Bosch, J. and Blanco, J. L. and Ãlvarez-Martinez, M. and Ugarte, A. and Inciarte, A. and de la Mora, L. and Torres, B. and MartÃ­nez-Rebollar, M. and Ambrosioni, J. and FernaÃ¡ndez, E. and Hurtado, J. C. and Mallolas, J. and MirÃ³, J. M. and Marcos, M. A. and MartÃ­nez, E. | Impact of coronavirus disease 2019 epidemics on prevention and care for HIV and other sexually transmitted infections | 2022 | Wrong outcome |
| De Lima-Karagiannis, A. and Juillerat, P. and Sebastian, S. and Pedersen, N. and Bar-Gil Shitrit, A. and van der Woude, C. J. | Management of Pregnant Inflammatory Bowel Disease Patients During the COVID-19 Pandemic | 2020 | Wrong population |
| De Meester, Johan and De Bacquer, Dirk and Naesens, Maarten and Meijers, Bjorn and Couttenye, Marie M and De Vriese, An S | Incidence, characteristics, and outcome of COVID-19 in adults on kidney replacement therapy: a regionwide registry study | 2021 | Wrong outcome |
| De Sirkar, S. and Yu, M. | Predictors of all-cause mortality in heart transplant recipients with covid-19 | 2021 | n<50 |
| De Waard, L. and Langenegger, E. and Erasmus, K. and Van der Merwe, T. and Olivier, S. E. and Du Toit, N. and Paulsen, C. and Nkangana, N. and Van Niekerk, M. and Moodley, A. and Schell, S. and Taljaard, J. and Botha, M. H. and Dramowski, A. and Cluver, C. A. and Bekker, A. | Maternal and neonatal outcomes of COVID-19 in a high-risk pregnant cohort with and without HIV | 2021 | Wrong population |
| Dejani, N. N. and Elshabrawy, H. A. and Bezerra Filho, Cdsm and de Sousa, D. P. | Anticoronavirus and Immunomodulatory Phenolic Compounds: Opportunities and Pharmacotherapeutic Perspectives | 2021 | Wrong outcome |
| Della Pia, A. and Zhao, C. and J and ir, P. and Gupta, A. and Batistick, M. and Kim, G. Y. G. and Xia, Y. and Ahn, J. and Magarelli, G. and Lukasik, B. and Leslie, L. A. and Goy, A. H. and Ip, A. and Feldman, T. A. | Improved Survival of Lymphoma Patients with COVID-19 in the Modern Treatment and Vaccination Era | 2022 | Single centre |
| Dell'Edera, A. and Borghesan, F. and Favero, E. and Rattazzi, M. and Scarpa, R. and Tartaglia, L. and Agostini, C. and Cinetto, F. | Venom immunotherapy during COVID-19 pandemic: Experience from a University Allergy Center in Northern Italy | 2020 | Wrong population |
| Deputy, M. and Sahnan, K. and Worley, G. and Patel, K. and Balinskaite, V. and Bottle, A. and Aylin, P. and Burns, E. M. and Hart, A. and Faiz, O. | The use of, and outcomes for, inflammatory bowel disease services during the Covid-19 pandemic: a nationwide observational study | 2022 | Wrong population |
| Devresse, Arnaud and De Greef, Julien and Yombi, Jean Cyr and Belkhir, Leila and Goffin, Eric and Kanaan, Nada | Immunosuppression and SARS-CoV-2 infection in kidney transplant recipients | 2022 | Incorrect study design |
| Dh and , Abhay and Razonable, Raymund R | COVID-19 and solid organ transplantation: role of anti-SARS-CoV-2 monoclonal antibodies | 2022 | Incorrect study design |
| DomÃ­nguez-Rojas, J. A. and Rojas-Soto, N. and VÃ¡squez-Hoyos, P. and Coronado Munoz, A. J. | Difficult acute lymphoblastic leukaemia diagnosis in a paediatric patient with mixed presentation of COVID-19 acute respiratory failure and multisystemic inflammatory syndrome | 2022 | n<50 |
| Domjanovic, J. and Matetic, A. and Bakovic Kramaric, D. and Domjanovic Skopinic, T. and Boric Skaro, D. and Delic, N. and Runjic, F. and Jelicic, I. | Association of the novel CROW-65 risk score and mortality in hospitalized kidney transplant recipients with COVID-19 : A retrospective observational study | 2022 | n<50 |
| Dong, Y. and Li, Z. and Ding, S. and Liu, S. and Tang, Z. and Jia, L. and Liu, J. and Liu, Y. | HIV infection and risk of COVID-19 mortality: A meta-analysis | 2021 | Paediatric data |
| Dowd, J. B. and Andriano, L. and Brazel, D. M. and Rotondi, V. and Block, P. and Ding, X. and Liu, Y. and Mills, M. C. | Demographic science aids in understanding the spread and fatality rates of COVID-19 | 2020 | Wrong population |
| Drake, T. M. and Fairfield, C. J. and Pius, R. and Knight, S. R. and Norman, L. and Girvan, M. and Hardwick, H. E. and Docherty, A. B. and Thwaites, R. S. and Openshaw, P. J. M. and Baillie, J. K. and Harrison, E. M. and Semple, M. G. | Non-steroidal anti-inflammatory drug use and outcomes of COVID-19 in the ISARIC Clinical Characterisation Protocol UK cohort: a matched, prospective cohort study | 2021 | Wrong population |
| Dravid, A. and Kashiva, R. and Khan, Z. and Memon, D. and Kodre, A. and Potdar, P. and Mane, M. and Borse, R. and Pawar, V. and Patil, D. and Banerjee, D. and Bhoite, K. and Phar and e, R. and Kalyani, S. and Raut, P. and Bapte, M. and Mehta, A. and Reddy, M. S. and Bhayani, K. and Laxmi, S. S. and Vishnu, P. D. and Srivastava, S. and Kh and elwal, S. and More, S. and Shinde, R. and Pawar, M. and Harshe, A. and Kadam, S. and Mahajan, U. and Joshi, G. and Mane, D. | Combination therapy of Tocilizumab and steroid for management of COVID-19 associated cytokine release syndrome: A single center experience from Pune, Western India | 2021 | Wrong population |
| D'Silva, K. M. and Wallace, Z. S. | COVID-19 and rheumatoid arthritis | 2021 | Incorrect study design |
| Duran, J. M. and Barat, M. and Lin, A. Y. and King, K. R. and Greenberg, B. and Adler, E. D. and Aslam, S. | Low mortality in SARS-CoV-2 infected heart transplant recipients at a single center | 2022 | n<50 |
| Durstenfeld, M. S. and Sun, K. and Ma, Y. and Rodriguez, F. and Secemsky, E. A. and Parikh, R. V. and Hsue, P. Y. | Association of HIV infection with outcomes among adults hospitalized with COVID-19 | 2022 | Wrong outcome |
| Dutschke, A. and Wejse, C. and Nanque, J. P. and Medina, C. and HÃ¸nge, B. L. and Jespersen, S. | SARS-CoV-2 seroprevalence among people living with HIV in Guinea-Bissau | 2022 | Wrong outcome |
| Dzinamarira, T. and Murewanhema, G. and Chitungo, I. and Ngara, B. and Nkambule, S. J. and Madziva, R. and Herrera, H. and Mukwenha, S. and Cuadros, D. F. and Iradukunda, P. G. and Mashora, M. and Tungwarara, N. and Rwibasira, G. N. and Musuka, G. | Risk of mortality in HIV-infected COVID-19 patients: A systematic review and meta-analysis | 2022 | Paediatric data |
| Ebrahimi, A. and Sayad, B. and Rahimi, Z. | COVID-19 and psoriasis: biologic treatment and challenges | 2022 | Duplicate |
| Edara, L. and Suvvari, T. K. and Kutikuppala, L. V. S. | High Dose Steroid Therapy to Prevent Severe Hypoxia in COVID-19 Patients: A Potential Solution for Low Resource Clinical Setting | 2020 | Wrong population |
| Efimov, S. V. and Matsiyeuskaya, N. V. and Boytsova, O. V. and Akhieva, L. Y. and Kuntsevich, E. V. and Troshina, A. A. and Kvasova, E. I. and Tikhonov, A. A. and Khomyakova, N. F. and Harrison, F. and Rossi, J. F. and Hardman, T. C. | Open-label use of an aliphatic polyamine immunomodulator in patients hospitalized with COVID-19 | 2022 | Wrong population |
| Eiting, M. M. and Phillips, K. and Smith, L. | The Evolution of Mortality in Kidney Transplant Recipients at a Single Center in the Era of Covid | 2022 | Insufficient original data |
| El Karoui, Khalil and De Vriese, An S | COVID-19 in dialysis: clinical impact, immune response, prevention and treatment | 2022 | Incorrect study design |
| Elaiw, A. M. and Al Agha, A. D. and Azoz, S. A. and Ramadan, E. | Global analysis of within-host SARS-CoV-2/HIV coinfection model with latency | 2022 | Wrong outcome |
| Elemam, N. M. and Maghazachi, A. A. and Hannawi, S. | COVID-19 infection and rheumatoid arthritis: mutual outburst cytokines and remedies | 2021 | Wrong outcome |
| ElGohary, Ghada M and Hashmi, Shahrukh and Styczynski, Jan and Kharfan-Dabaja, Mohamed A and Alblooshi, Rehab M and de la CÃ¡mara, Rafael and Mohmed, Sherif and Alshaibani, Alfadel and Cesaro, Simone and Abd El-Aziz, Nashwa | The risk and prognosis of COVID-19 infection in cancer patients: A systematic review and meta-analysis | 2020 | n<50 |
| Elhadi, M. and Momen, A. A. and Abdulhadi, Omas and Msherghi, A. | Multi-organ failure after acute kidney injury in patient with HIV and COVID-19 | 2020 | Incorrect study design |
| El-Malky, A. M. and YA, S. Albalawi and Alanazi, S. M. and Saed Albalawi, M. A. and Althobaiti, A. N. and ZA, A. Kassarah and HA, I. Alzahrani and AS, A. Al-Balawi | Severe acute respiratory syndrome coronavirus 2 and risk of inhospital mortality among end-stage renal disease patients with rheumatoid arthritis: A scientific perspective | 2021 | Compound risk group |
| El-Qushayri, A. E. and Nardone, B. | Psoriasis exacerbation after COVID-19 vaccines: A brief report of the reported cases | 2022 | Wrong outcome |
| Emmi, Giacomo and Bettiol, Aless and ra and Mattioli, Irene and Silvestri, Elena and Di Scala, Gerardo and Urban, Maria Letizia and Vaglio, Augusto and Prisco, Domenico | SARS-CoV-2 infection among patients with systemic autoimmune diseases | 2020 | Incorrect study design |
| ErkoÃ§, M. and Ã–ztÃ¼rk, BÃ– and Mungan, D. and Ã–ztuna, D. and Bavbek, S. and Demirel, Y. S. and AydÄ±n, Ã– and Sin, B. A. | Allergen-specific immunotherapy practices and course of coronavirus disease 2019 (COVID-19) in patients during COVID-19 | 2022 | Wrong population |
| Esmaeilzadeh, A. and Rostami, S. and Yeganeh, P. M. and Tahmasebi, S. and Ahmadi, M. | Recent advances in antibody-based immunotherapy strategies for COVID-19 | 2021 | Wrong population |
| Eybpoosh, S. and Afshari, M. and Haghdoost, A. A. and Afsar Kazerooni, P. and Gouya, M. M. and Tayeri, K. | Severity and mortality of COVID-19 infection in HIV-infected individuals: Preliminary findings from Iran | 2021 | n<50 |
| FacciolÃ , A. and D'Amato, S. and Calimeri, S. and Giudice, D. L. and Micali, C. and Russotto, Y. and Venanzi Rullo, E. and Nunnari, G. and Squeri, R. and PellicanÃ², G. F. | Efficacy of COVID-19 Vaccination in People Living with HIV: A Public Health Fundamental Tool for the Protection of Patients and the Correct Management of Infection | 2022 | Wrong outcome |
| Fathi, M. and Vakili, K. and Jazi, K. and Sadeghi, M. A. and Hajiesmaeili, M. and Mohamadkhani, A. and Rezaei-Tavirani, M. and Tavasol, A. | Challenges of cancer immunotherapy and chemotherapy during the COVID-19 pandemic | 2022 | Duplicate |
| Favalli, Ennio Giulio and Bugatti, Serena and Klersy, Catherine and Biggioggero, Martina and Rossi, Silvia and De Lucia, Orazio and Bobbio-Pallavicini, Francesca and Murgo, Antonella and Balduzzi, Silvia and Caporali, Roberto | Impact of corticosteroids and immunosuppressive therapies on symptomatic SARS-CoV-2 infection in a large cohort of patients with chronic inflammatory arthritis | 2020 | Wrong outcome |
| Fehr, D. and LebouchÃ©, B. and Ruppenthal, L. and Brownc, M. and Obasc, N. and BourbonniÃ¨re, E. and Girouard, J. and Massicotte, A. and Jenabian, M. A. and Almomen, A. A. and Frenette, C. and de Pokom and y, A. and Cox, J. and Giannakis, A. and Cheng, M. and Kronfli, N. and Tsoukas, C. and Zahedi, N. and Szabo, J. and Dehghani, K. and Brouillette, M. J. and Falutz, J. and Turner, H. and Hamel, A. and Duchesneau, C. and Lanthier-Brun, J. and Klein, M. and Routy, J. P. and Costiniuk, C. T. | Characterization of people living with HIV in a Montreal-based tertiary care center with COVID-19 during the first wave of the pandemic | 2022 | n<50 |
| Feitosa, M. R. and Parra, R. S. and de Camargo, H. P. and Ferreira, S. D. C. and Troncon, L. E. A. and da Rocha, J. J. R. and FÃ©res, O. | COVID-19 quarantine measures are associated with negative social impacts and compromised follow-up care in patients with inflammatory bowel disease in Brazil | 2021 | Wrong outcome |
| Fenig, Y. and Santeusanio, A. and Menon, M. and Liu, C. and Rana, M. and Shapiro, R. | Influence of immunosuppressant management on mortality in kidney transplant recipients hospitalized with covid-19 | 2021 | n<50 |
| Ferraccioli, E. S. and Gremese, E. and Ferraccioli, G. | Morbidity and Mortality From COVID-19 Are Not Increased Among Children or Patients With Autoimmune Rheumatic Disease-Possible Immunologic Rationale: Comment on the Article by Henderson et al | 2020 | Wrong population |
| Ferri, C. and Raimondo, V. and Gragnani, L. and Giuggioli, D. and Dagna, L. and Tavoni, A. and Ursini, F. and L'Andolina, M. and Caso, F. and Ruscitti, P. and Caminiti, M. and Foti, R. and Riccieri, V. and Guiducci, S. and Pellegrini, R. and Zanatta, E. and Varcasia, G. and Olivo, D. and Gigliotti, P. and Cuomo, G. and Murdaca, G. and Cecchetti, R. and De Angelis, R. and Romeo, N. and Ingegnoli, F. and Cozzi, F. and Codullo, V. and Cavazzana, I. and Colaci, M. and Abignano, G. and De Santis, M. and Lubrano, E. and Fusaro, E. and Spinella, A. and Lumetti, F. and De Luca, G. and Bell and o-R and one, S. and Visalli, E. and Dal Bosco, Y. and Amato, G. and Giannini, D. and Bilia, S. and Masini, F. and Pellegrino, G. and Pigatto, E. and Generali, E. and Mariano, G. P. and Pettiti, G. and Zanframundo, G. and Brittelli, R. and Aiello, V. and Caminiti, R. and Scorpiniti, D. and Ferrari, T. and Campochiaro, C. and Brusi, V. and Fredi, M. and Moschetti, L. and Cacciapaglia, F. and Paparo, S. R. and Ragusa, F. and Mazzi, V. and Elia, G. and Ferrari, S. M. and Di Cola, I. and Vadacca, M. and Lorusso, S. and Monti, M. and Lorini, S. and Aprile, M. L. and Tasso, M. and Miccoli, M. and Bosello, S. and D'Angelo, S. and Doria, A. and Franceschini, F. and Meliconi, R. and Matucci-Cerinic, M. and Iannone, F. and Giacomelli, R. and Salvarani, C. and Zignego, A. L. and Fallahi, P. and Antonelli, A. | Prevalence and Death Rate of COVID-19 in Autoimmune Systemic Diseases in the First Three Pandemic Waves. Relationship with Disease Subgroups and Ongoing Therapies | 2022 | Duplicate |
| Feuillet, V. and Canard, B. and Trautmann, A. | Combining Antivirals and Immunomodulators to Fight COVID-19 | 2021 | Wrong population |
| Fisher, M. C. and Fazzari, M. J. and Hanna, D. B. and Patel, V. V. and Felsen, U. R. and Alahiri, E. and Byju, A. and Akiyama, M. J. and Ginsberg, M. S. and Anastos, K. and Ross, M. J. | Brief Report: Acute Kidney Injury in People Living With HIV Hospitalized With Coronavirus Disease 2019: Clinical Characteristics and Outcomes | 2021 | Wrong outcome |
| Fishman, Jay A | The immunocompromised transplant recipient and SARS-CoV-2 infection | 2020 | Incorrect study design |
| Flanagan, C. F. and McCann, N. and Stover, J. and Freedberg, K. A. and Ciaranello, A. L. | Do not forget the children: a model-based analysis on the potential impact of COVID-19-associated interruptions in paediatric HIV prevention and care | 2022 | Wrong population |
| Fleischer, B. and Olum, R. and Nakwagala, F. N. and Nassozi, D. R. and Pitua, I. and Paintsil, E. and Baluku, J. B. and Bongomin, F. | Higher intensive care unit consultations for COVID-19 patients living with HIV compared to those without HIV coinfection in Uganda | 2022 | n<50 |
| Fox, T. A. and Troy-Barnes, E. and Kirkwood, A. A. and Chan, W. Y. and Day, J. W. and Chavda, S. J. and Kumar, E. A. and David, K. and Tomkins, O. and Sanchez, E. and Scully, M. and Khwaja, A. and Lambert, J. and Singer, M. and Roddie, C. and Morris, E. C. and Yong, K. L. and Thomson, K. J. and Ardeshna, K. M. | Response to 'Impact of immunosuppression on mortality in critically ill COVID-19 patients' | 2020 | Wrong outcome |
| Fox, T. A. and Troy-Barnes, E. and Kirkwood, A. A. and Chan, W. Y. and Day, J. W. and Chavda, S. J. and Kumar, E. A. and David, K. and Tomkins, O. and Sanchez, E. and Scully, M. and Khwaja, A. and Lambert, J. and Singer, M. and Roddie, C. and Morris, E. C. and Yong, K. L. and Thomson, K. J. and Ardeshna, K. M. | Clinical outcomes and risk factors for severe COVID-19 in patients with haematological disorders receiving chemo- or immunotherapy | 2020 | Single centre |
| Franke, K. | COVID-19 after liver transplant: Mortality is comparable with other patient groups. [German] | 2020 | Foreign language |
| Franquet, T. and Domingo, P. | Pulmonary Infections in People Living with HIV | 2022 | Wrong outcome |
| Fung, Monica and Chiu, Charles Y and DeVoe, Catherine and Doernberg, Sarah B and Schwartz, Brian S and Langelier, Charles and Henrich, Timothy J and Yokoe, Deborah and Davis, John and Hays, Steven R | Clinical outcomes and serologic response in solid organ transplant recipients with COVID-19: a case series from the United States | 2020 | n<50 |
| GÃ¡lvez-Romero, J. L. and Palmeros-Rojas, O. and Real-RamÃ­rez, F. A. and SÃ¡nchez-Romero, S. and Tome-Maxil, R. and RamÃ­rez-S and oval, M. P. and Olivos-RodrÃ­guez, R. and Flores-EncarnaciÃ³n, S. E. and Cabrera-Estrada, A. A. and Ãvila-Morales, J. and CortÃ©s-SÃ¡nchez, V. and Sarmiento-Padilla, G. and Tezmol-RamÃ­rez, S. E. and Aparicio-HernÃ¡ndez, D. and Urbina-SÃ¡nchez, M. I. and GÃ³mez-Pluma, MÃ and Cisneros-MÃ©ndez, S. and RodrÃ­guez-Rivas, D. I. and Reyes-Inurrigarro, S. and CortÃ©s-DÃ­az, G. and Cruz-Delgado, C. and Navarro-GonzÃ¡lez, J. and Deveaux-Homs, J. and Pedraza-SÃ¡nchez, S. | Cyclosporine A plus low-dose steroid treatment in COVID-19 improves clinical outcomes in patients with moderate to severe disease: A pilot study | 2021 | Wrong population |
| Gaitzsch, Erik and Passerini, Verena and Khatamzas, Elham and Strobl, Carolin D and Muenchhoff, Maximilian and Scherer, Clemens and Osterman, Andreas and Heide, Michael and Reischer, Anna and Subklewe, Marion | COVID-19 in patients receiving CD20-depleting immunochemotherapy for B-cell lymphoma | 2021 | n<50 |
| Gamboa, E. and Duran, M. and Gathe, J. C., Jr. and Surani, S. and Varon, J. | COVID-19 Coexisting With the Human Immunodeficiency Virus: A Case Report | 2020 | n<50 |
| Gapud, E. J. and Kronbichler, A. and Gauckler, P. and Geetha, D. | Immunotherapy for ANCA-associated vasculitis during the COVID-19 pandemic | 2020 | Insufficient original data |
| Gasmi, A. and Tippairote, T. and Mujawdiya, P. K. and Peana, M. and Menzel, A. and Dadar, M. and Gasmi Benahmed, A. and BjÃ¸rklund, G. | Micronutrients as immunomodulatory tools for COVID-19 management | 2020 | Wrong population |
| Gavriilidis, P. and Pai, M. | The Impact of COVID-19 Global Pandemic on Morbidity and Mortality of Liver Transplant Recipients Children and Adults: A Systematic Review of Case Series | 2020 | Wrong outcome |
| Gaziano, R. and Pistoia, E. S. and Campione, E. and Fontana, C. and Marino, D. and Favaro, M. and Pica, F. and Di Francesco, P. | Immunomodulatory agents as potential therapeutic or preventive strategies for COVID-19 | 2021 | Wrong outcome |
| Gazzaruso, C. and Mariani, G. and Ravetto, C. and Malinverni, L. and Tondelli, E. and Cerrone, M. and Sala, V. and Bevilacqua, L. and Altavilla, T. and Coppola, A. and Gallotti, P. | Lupus anticoagulant and mortality in patients hospitalized for COVID-19 | 2021 | Wrong population |
| Gendron, N. and Dragon-Durey, M. A. and Chocron, R. and Darnige, L. and Jourdi, G. and Philippe, A. and Chenevier-Gobeaux, C. and Hadjadj, J. and Duchemin, J. and Khider, L. and Yatim, N. and Goudot, G. and Krzisch, D. and Debuc, B. and Mauge, L. and Levavasseur, F. and Pene, F. and Boussier, J. and Sourdeau, E. and Brichet, J. and Ochat, N. and Goulvestre, C. and Peronino, C. and Szwebel, T. A. and Pages, F. and Gaussem, P. and Samama, C. M. and Cheurfa, C. and Planquette, B. and Sanchez, O. and Diehl, J. L. and Mirault, T. and Fontenay, M. and Terrier, B. and Smadja, D. M. | Lupus Anticoagulant Single Positivity During the Acute Phase of COVID-19 Is Not Associated With Venous Thromboembolism or In-Hospital Mortality | 2021 | Wrong population |
| Gendron, N. and Dragon-Durey, M. A. and Chocron, R. and Darnige, L. and Jourdi, G. and Philippe, A. and Hadjadj, J. and Duchemin, J. and Khider, L. and Yatim, N. and Mauge, L. and Gaussem, P. and Samama, C. M. and Planquette, B. and Sanchez, O. and Diehl, J. L. and Mirault, T. and Fontenay, M. and Terrier, B. and Smadja, D. M. | Lupus anticoagulant positivity is not associated with venous thromboembolism or in-hospital mortality in COVID-19 | 2021 | Wrong population |
| Geretti, Anna Maria and Stockdale, Alex and er J and Kelly, Sophie H and Cevik, Muge and Collins, Simon and Waters, Laura and Villa, Giovanni and Docherty, Annemarie and Harrison, Ewen M and Turtle, Lance | Outcomes of coronavirus disease 2019 (COVID-19) related hospitalization among people with human immunodeficiency virus (HIV) in the ISARIC World Health Organization (WHO) clinical characterization protocol (UK): a prospective observational study | 2021 | Duplicate |
| Ghosh, N. and Tirpack, A. and Chan, K. K. and Bass, A. R. | Impact of COVID-19 on patients with rheumatic complications of cancer immunotherapy: results of a registry survey | 2020 | Wrong outcome |
| Gilissen, L. P. L. and Heinen, S. G. H. and Rijpma-Jacobs, L. and Schoon, E. and Schreuder, R. M. and Wensing, A. M. and van der Ende-van Loon, M. C. M. and Bloemen, J. G. and Stapelbroek, J. M. and Stronkhorst, A. | Neither inflammatory bowel disease nor immunosuppressants are associated with an increased risk of severe COVID-19: an observational Dutch cohort study | 2022 | Single centre |
| Ginzberg, D. and Pierce, K. and Kreiger-Benson, E. and Graves, M. and Neumann, H. and Ali, N. and Gidea, C. and Park, J. and Mehta, S. | Clinical signs predictive of covid-19 mortality among transplant recipients | 2021 | Single centre |
| Gisondi, P. and Bellinato, F. and Chiricozzi, A. and Girolomoni, G. | The Risk of COVID-19 Pandemic in Patients with Moderate to Severe Plaque Psoriasis Receiving Systemic Treatments | 2020 | Insufficient original data |
| Gisondi, P. and Zaza, G. and Del Giglio, M. and Rossi, M. and Iacono, V. and Girolomoni, G. | Risk of hospitalization and death from COVID-19 infection in patients with chronic plaque psoriasis receiving a biological treatment and renal transplanted recipients in maintenance immunosuppressive treatment | 2020 | Incorrect study design |
| Goldman, J. D. and Gonzalez, M. A. and RÃ¼thrich, M. M. and Sharon, E. and von Lilienfeld-Toal, M. | COVID-19 and Cancer: Special Considerations for Patients Receiving Immunotherapy and Immunosuppressive Cancer Therapies | 2022 | Insufficient original data |
| Goldman, J. D. and Robinson, P. C. and Uldrick, T. S. and Ljungman, P. | COVID-19 in immunocompromised populations: implications for prognosis and repurposing of immunotherapies | 2021 | Incorrect study design |
| Golin, R. and Godfrey, C. and Firth, J. and Lee, L. and Minior, T. and Phelps, B. R. and Raizes, E. G. and Ake, J. A. and Siberry, G. K. | PEPFAR's response to the convergence of the HIV and COVID-19 pandemics in Sub-Saharan Africa | 2020 | Wrong outcome |
| Gottlieb, R. L. and Askar, M. and Chen, L. and Ch and ak, A. and Mozaffari, E. and Thrun, M. and Haubrich, R. | Clinical Outcomes in a Large Transplant Cohort Hospitalized with COVID-19: Mycophenolate Mofetil (MMF) Utilization and Mortality Trends | 2022 | Insufficient original data |
| Govender, R. and Moodley, J. and Naicker, T. | The COVID-19 Pandemic: an Appraisal of its Impact on Human Immunodeficiency Virus Infection and Pre-Eclampsia | 2021 | Wrong population |
| Goyal, A. and Joshi, A. and Saigal, S. and Brahmam, D. and Niwariya, Y. and Khurana, A. and Khadanga, S. and Mitra, A. | Whether Early Steroid dose is associated with lower mortality in COVID-19 critically ill Patients- An exploratory chart review | 2022 | Wrong population |
| Graff, L. A. and Fowler, S. and Jones, J. L. and Benchimol, E. I. and Bitton, A. and Huang, J. G. and Kuenzig, M. E. and Kaplan, G. G. and Lee, K. and Mukhtar, M. S. and T and on, P. and Targownik, L. E. and Windsor, J. W. and Bernstein, C. N. | Crohn's and Colitis Canada's 2021 Impact of COVID-19 and Inflammatory Bowel Disease in Canada: Mental Health and Quality of Life | 2021 | Wrong outcome |
| Grange, Lucile and Guilpain, Philippe and Truchetet, Marie-Elise and Cracowski, Jean-Luc and Pharmacology, French Society of and Therapeutics | Challenges of autoimmune rheumatic disease treatment during the COVID-19 pandemic: a review | 2020 | Incorrect study design |
| Granger, Camille and Guedeney, Paul and Arnaud, Camille and Guendouz, Soulef and Cimadevilla, Claire and Kerneis, Mathieu and Kerneis, Caroline and Zeitouni, Michel and Verdonk, Constance and Legeai, Camille | Clinical manifestations and outcomes of coronavirus diseaseâ€19 in heart transplant recipients: a multicentre case series with a systematic review and metaâ€analysis | 2021 | n<50 |
| Granholm, A. and Munch, M. W. and Andersen-Ranberg, N. and Myatra, S. N. and Vijayaraghavan, B. K. T. and Venkatesh, B. and Jha, V. and Wahlin, R. R. and Jakob, S. M. and Cioccari, L. and MÃ¸ller, M. H. and Perner, A. | Heterogeneous treatment effects of dexamethasone 12â€‰mg versus 6â€‰mg in patients with COVID-19 and severe hypoxaemia-Post hoc exploratory analyses of the COVID STEROID 2 trial | 2022 | Wrong population |
| Granholm, A. and Munch, M. W. and Myatra, S. N. and Vijayaraghavan, B. K. T. and Cronhjort, M. and Wahlin, R. R. and Jakob, S. M. and Cioccari, L. and KjÃ¦r, M. N. and Vesterlund, G. K. and Meyhoff, T. S. and Helleberg, M. and MÃ¸ller, M. H. and Benfield, T. and Venkatesh, B. and Hammond, N. E. and Micallef, S. and Bassi, A. and John, O. and Jha, V. and Kristiansen, K. T. and Ulrik, C. S. and JÃ¸rgensen, V. L. and Smitt, M. and Bestle, M. H. and Andreasen, A. S. and Poulsen, L. M. and Rasmussen, B. S. and BrÃ¸chner, A. C. and StrÃ¸m, T. and MÃ¸ller, A. and Khan, M. S. and Padmanaban, A. and Divatia, J. V. and Saseedharan, S. and Borawake, K. and Kapadia, F. and Dixit, S. and Chawla, R. and Shukla, U. and Amin, P. and Chew, M. S. and Wamberg, C. A. and Gluud, C. and Lange, T. and Perner, A. | Dexamethasone 12Â mg versus 6Â mg for patients with COVID-19 and severe hypoxaemia: a pre-planned, secondary Bayesian analysis of the COVID STEROID 2 trial | 2022 | Wrong population |
| Granholm, A. and Munch, M. W. and Myatra, S. N. and Vijayaraghavan, B. K. T. and Cronhjort, M. and Wahlin, R. R. and Jakob, S. M. and Cioccari, L. and Kjaer, M. N. and Vesterlund, G. K. and Meyhoff, T. S. and Helleberg, M. and MÃ¸ller, M. H. and Benfield, T. and Venkatesh, B. and Hammond, N. and Micallef, S. and Bassi, A. and John, O. and Jha, V. and Kristiansen, K. T. and Ulrik, C. S. and JÃ¸rgensen, V. L. and Smitt, M. and Bestle, M. H. and Andreasen, A. S. and Poulsen, L. M. and Rasmussen, B. S. and BrÃ¸chner, A. C. and StrÃ¸m, T. and MÃ¸ller, A. and Khan, M. S. and Padmanaban, A. and Divatia, J. V. and Saseedharan, S. and Borawake, K. and Kapadia, F. and Dixit, S. and Chawla, R. and Shukla, U. and Amin, P. and Chew, M. S. and Gluud, C. and Lange, T. and Perner, A. | Higher vs Lower Doses of Dexamethasone in Patients with COVID-19 and Severe Hypoxia (COVID STEROID 2) trial: Protocol for a secondary Bayesian analysis | 2021 | Incorrect study design |
| Gravett, R. M. and Marrazzo, J. M. | HIV and COVID-19: Lessons From HIV and STI Harm Reduction Strategies | 2021 | Wrong outcome |
| Grawert, S. | COVID-19 mortality in patients with rheumatoid arthritis. [German] | 2021 | Foreign language |
| Gudipati, S. and Brar, I. and Murray, S. and McKinnon, J. E. and Yared, N. and Markowitz, N. | Descriptive Analysis of Patients Living With HIV Affected by COVID-19 | 2020 | n<50 |
| Guisado-Vasco, P. and Valderas-Ortega, S. and CarralÃ³n-GonzÃ¡lez, M. M. and Roda-Santacruz, A. and GonzÃ¡lez-Cortijo, L. and Sotres-FernÃ¡ndez, G. and MartÃ­-Ballesteros, E. M. and Luque-Pinilla, J. M. and Almagro-Casado, E. and La Coma-Lanuza, F. J. and Barrena-Puertas, R. and Malo-Benages, E. J. and Monforte-GÃ³mez, M. J. and Diez-Munar, R. and Merino-Lanza, E. and Comeche-Casanova, L. and Remirez-de-Esparza-Otero, M. and Correyero-Plaza, M. and Recio-RodrÃ­guez, M. and RodrÃ­guez-LÃ³pez, M. and SÃ¡nchez-Manzano, M. D. and Andreu-VÃ¡zquez, C. and Thuissard-Vasallo, I. J. and MarÃ­a-TomÃ©, J. M. E. and Carnevali-Ruiz, D. | Clinical characteristics and outcomes among hospitalized adults with severe COVID-19 admitted to a tertiary medical center and receiving antiviral, antimalarials, glucocorticoids, or immunomodulation with tocilizumab or cyclosporine: A retrospective observational study (COQUIMA cohort) | 2020 | Immunosuppression as treatment |
| Gunaratne, S. H. and Tieu, H. V. and Wilkin, T. J. and Taylor, B. S. | CROI 2021: Advances in Antiretroviral Therapy for HIV and Antiviral Therapy for COVID-19 | 2021 | Wrong outcome |
| Gundogdu, O. and Demir, B. and Coskun, C. O. and Ersan, I. | Efficacy of pulse steroid therapy in patients critically ill with COVID-19 | 2021 | Wrong outcome |
| Gupta, A. and Shivaji, K. and Kadam, S. and Gupta, M. and Rodriguez, H. C. and Potty, A. G. and El-Amin, S. F., 3rd and Maffulli, N. | Immunomodulatory extracellular vesicles: an alternative to cell therapy for COVID-19 | 2021 | Wrong population |
| Gupta, T. and Debele, T. A. and Wei, Y. F. and Gupta, A. and Murtaza, M. and Su, W. P. | Synergistic Action of Immunotherapy and Nanotherapy against Cancer Patients Infected with SARS-CoV-2 and the Use of Artificial Intelligence | 2022 | Wrong outcome |
| Gurion, R. and Rozovski, U. and Itchaki, G. and Gafter-Gvili, A. and Leibovitch, C. and Raanani, P. and Ben-Zvi, H. and Szwarcwort, M. and Taylor-Abigadol, M. and Dann, E. J. and Horesh, N. and Inbar, T. and Tzoran, I. and Lavi, N. and Fineman, R. and Ringelstein-Harlev, S. and Horowitz, N. A. | Humoral serological response to the BNT162b2 vaccine is abrogated in lymphoma patients within the first 12 months following treatment with anti-CD2O antibodies | 2022 | Wrong outcome |
| Gutierrez, M. D. M. and Mur, I. and Mateo, M. G. and Vidal, F. and Domingo, P. | Pharmacological considerations for the treatment of COVID-19 in people living with HIV (PLWH) | 2021 | Wrong outcome |
| Gyebi, G. A. and Ogunyemi, O. M. and Ibrahim, I. M. and Afolabi, S. O. and Adebayo, J. O. | Dual targeting of cytokine storm and viral replication in COVID-19 by plant-derived steroidal pregnanes: An in silico perspective | 2021 | Incorrect study design |
| HÃ¤rter, G. and Spinner, C. D. and Roider, J. and Bickel, M. and Krznaric, I. and Grunwald, S. and Schabaz, F. and Gillor, D. and Postel, N. and Mueller, M. C. and MÃ¼ller, M. and RÃ¶mer, K. and Schewe, K. and Hoffmann, C. | COVID-19 in people living with human immunodeficiency virus: a case series of 33 patients | 2020 | n<50 |
| Hage, RenÃ© and Steinack, Carolin and Benden, Christian and Schuurmans, MacÃ© M | COVID-19 in patients with solid organ transplantation: a systematic review | 2020 | n<50 |
| Haller, M. J. and Jacobsen, L. M. and Posgai, A. L. and Schatz, D. A. | How Do We Move Type 1 Diabetes Immunotherapies Forward During the Current COVID-19 Pandemic? | 2021 | Wrong outcome |
| Hamiduzzaman, A. and Reddy, U. | Improved Mortality in COVID-19 Kidney Transplant Recipients Treated with Bamlanivimab | 2022 | Single centre |
| Hamza, M. and Alhujaily, M. and Alosaimi, B. and El Bakkouri, K. and AlDughaim, M. S. and Alonazi, M. and Alanazi, M. A. and Abbass, B. and Alshehri, A. and Al-Shouli, S. T. and Alturaiki, W. and Awadalla, M. | Association between inflammatory cytokines/chemokines, clinical laboratory parameters, disease severity and in-hospital mortality in critical and mild COVID-19 patients without comorbidities or immune-mediated diseases | 2022 | Wrong population |
| Han, X. and Yu, X. and Han, Y. and Fang, Q. and Shen, C. and Liu, H. and Wang, P. and Wang, Y. and Li, X. | Safety and Immunogenicity of Inactivated COVID-19 Vaccines Among People Living with HIV in China | 2022 | Wrong population |
| Hanif, F. and Satiti, S. and Subagya, S. and Retnowulan, H. and Subronto, Y. W. and Mulya, D. P. and Ar Rochmah, M. | Progressive Worsening of Neurological Manifestations in HIV-Associated Opportunistic Central Nervous System (CNS) Infection Patients After COVID-19 Vaccinations: A Possible Co-Incidence Causality | 2022 | Wrong outcome |
| Hannan, T. B. and Paul, S. and Barai, L. and Alam, M. R. and Chowdhury, F. R. | Rhino-Orbital Mucormycosis After COVID-19 Infection in a Patient With Non-Hodgkin's Lymphoma | 2022 | n<50 |
| Hardgrave, H. and Wells, A. and Nigh, J. and Klutts, G. and Krinock, D. and Osborn, T. and Bhusal, S. and Rude, M. K. and Burdine, L. and Giorgakis, E. | COVID-19 Mortality in Vaccinated vs. Unvaccinated Liver & Kidney Transplant Recipients: A Single-Center United States Propensity Score Matching Study on Historical Data | 2022 | Insufficient original data |
| Hardy, Y. O. and Amenuke, D. A. and Hutton-Mensah, K. A. and Chadwick, D. R. and Larsen-Reindorf, R. | Presentation and outcome of COVID-19 in HIV patients with high viral loads and opportunistic infections: a case series | 2020 | n<50 |
| Hariyanto, T. I. and Putri, C. and Frinka, P. and Louisa, J. and Lugito, N. P. H. and Kurniawan, A. | Human Immunodeficiency Virus (HIV) and outcomes from coronavirus disease 2019 (COVID-19) pneumonia: A Meta-Analysis and Meta-Regression | 2021 | Incorrect study design |
| Harthan, A. A. and Nadiger, M. and McGarvey, J. S. and Hanson, K. and Gharpure, V. P. and Bjornstad, E. C. and Chiotos, K. and Miller, A. S. and Reikoff, R. A. and Gajic, O. and Kumar, V. and Walkey, A. J. and Kashyap, R. and Tripathi, S. | Early combination therapy with immunoglobulin and steroids is associated with shorter ICU length of stay in Multisystem Inflammatory Syndrome in Children (MIS-C) associated with COVID-19: A retrospective cohort analysis from 28 U.S. Hospitals | 2022 | Wrong population |
| Hassan, A. T. and Elmoniem, A. E. A. and Abdelrady, M. M. and Mohamed, M. E. and Mokhtar, M. A. and Elsherif, A. A. and Saied, G. M. and Kasem, S. M. | Challenges in Steroid and Anticoagulant Therapy in Severe COVID-19 Pneumonia: A Prospective Study | 2021 | Wrong population |
| Hejazian, S. S. and Hejazian, S. M. and Farnood, F. and Abedi Azar, S. | Dysregulation of immunity in COVID-19 and SLE | 2022 | Insufficient original data |
| Heldman, M. R. and Kates, O. S. and Safa, K. and Kotton, C. N. and Multani, A. and Georgia, S. J. and Steinbrink, J. M. and Alex and er, B. D. and Blumberg, E. A. and Haydel, B. and Hemmige, V. and Hemmersbach-Miller, M. and La Hoz, R. M. and Moni, L. and Condor, Y. and Flores, S. and Munoz, C. G. and Guitierrez, J. and Diaz, E. I. and Diaz, D. and Vianna, R. and Guerra, G. and Loebe, M. and Yabu, J. M. and Kramer, K. H. and Tanna, S. D. and Ison, M. G. and Rakita, R. M. and Malinis, M. and Azar, M. M. and McCort, M. E. and Singh, P. P. and Velioglu, A. and Mehta, S. A. and van Duin, D. and Goldman, J. D. and Lease, E. D. and Wald, A. and Limaye, A. P. and Fisher, C. E. and Team, U. W. Covid-19 SOT Study | Delayed mortality among solid organ transplant recipients hospitalized for COVID-19 | 2022 | Duplicate |
| Heldman, M. R. and Rakita, R. M. and Lease, E. D. and Fisher, C. E. and Limaye, A. P. | Apples to apples: The challenges of studying COVID-19 mortality in solid organ transplant recipients | 2022 | Incorrect study design |
| Heldman, Madeleine R and Kates, Olivia S and Fisher, Cynthia E and Limaye, Ajit P | Immunosuppression in solid organ transplant recipients with COVID-19: More data, but still complicated | 2021 | Incorrect study design |
| Heldman, Madeleine R and Kates, Olivia S and Safa, Kassem and Kotton, Camille N and Georgia, Sarah J and Steinbrink, Julie M and Alex and er, Barbara D and Hemmersbachâ€Miller, Marion and Blumberg, Emily A and Crespo, Maria M | COVID-19 in hospitalized lung and nonâ€lung solid organ transplant recipients: a comparative analysis from a multicenter study | 2021 | Duplicate |
| Hendrickx, Rodinde and Jellingso, Mads and Sommer, Morten OA | Kidney patients remain at increased risk for succumbing to COVID-19 | 2022 | Wrong outcome |
| Ho, M. and Zanwar, S. and Buadi, F. K. and Ailawadhi, S. and Larsen, J. and Bergsagel, L. and Binder, M. and Chanan-Khan, A. and Dingli, D. and Dispenzieri, A. and Fonseca, R. and Gertz, M. A. and Gonsalves, W. and Go, R. S. and Hayman, S. and Kapoor, P. and Kourelis, T. and Lacy, M. Q. and Leung, N. and Lin, Y. and Muchtar, E. and Roy, V. and Sher, T. and Warsame, R. and Fonder, A. and Hobbs, M. and Hwa, Y. L. and Kyle, R. A. and Rajkumar, S. V. and Kumar, S. | Risk factors for severe infection and mortality In patients with COVID-19 in patients with multiple myeloma and AL amyloidosis | 2022 | Single centre |
| Hoffman, J. R. H. and Higa, K. C. and Lin, Y. and Reece, T. B. and Clevel and , J. C. and Aftab, M. and Rove, J. Y. | Noteworthy Cardiac Literature From 2021: Coronary Guideline Change Without New Data, Heart Transplant Donation After Cardiac Death, Covid Effects on Global Cardiac Surgery, and Attempt to Improve Dissection Remodeling | 2022 | Indirect mortality |
| Hoffmann, M. S. and Ganguly, S. | Delayed COVID-19 Respiratory Failure in Patients with Lymphoma on Rituximab-based Chemoimmunotherapy | 2021 | n<50 |
| Holtgrave, D. R. and Valdiserri, R. O. and Kalichman, S. C. and Del Rio, C. and Thompson, M. | Core Elements of a National COVID-19 Strategy: Lessons Learned from the US National HIV/AIDS Strategy | 2020 | Insufficient original data |
| Hornuss, D. and Giesen, R. and Biever, P. and Kern, W. V. | [Clinical benefit of Tocilizumab and other immunomodulating agents for treatment of COVID-19] | 2021 | Foreign language |
| Houle, B. and Kabudula, C. W. and Tilstra, A. M. and Mojola, S. A. and Schatz, E. and Clark, S. J. and Angotti, N. and GÃ³mez-OlivÃ©, F. X. and Menken, J. | Twin epidemics: the effects of HIV and systolic blood pressure on mortality risk in rural South Africa, 2010-2019 | 2022 | Wrong outcome |
| Hu, H. and Tang, N. and Zhang, F. and Li, L. and Li, L. | Bioinformatics and System Biology Approach to Identify the Influences of COVID-19 on Rheumatoid Arthritis | 2022 | Wrong outcome |
| Huang, D. and Zunong, J. and Li, M. and Li, D. and Gong, J. and Vermund, S. H. and Hu, Y. | COVID-19 Clinical Presentation Among HIV-Infected Persons in China: A Systematic Review | 2022 | n<50 |
| Huang, X. and Yu, M. and Fu, G. and Lan, G. and Li, L. and Yang, J. and Qiao, Y. and Zhao, J. and Qian, H. Z. and Zhang, X. and Liu, X. and Jin, X. and Chen, G. and Jiang, H. and Tang, W. and Wang, Z. and Xu, J. | Willingness to Receive COVID-19 Vaccination Among People Living With HIV and AIDS in China: Nationwide Cross-sectional Online Survey | 2021 | Wrong outcome |
| Hudzik, B. and Nowak, J. and Zubelewicz-Szkodzinska, B. | Consideration of immunomodulatory actions of morphine in COVID-19 - Short report | 2020 | Incorrect study design |
| Hugo, C. and Vehreschild, J. and Stecher, M. | Response to Invited Commentary "Undoubtedly, kidney transplant recipients have a higher mortality due to COVID-19 disease compared to the general population" | 2021 | Incorrect study design |
| Hyder Pottoo, F. and Abu-Izneid, T. and Mohammad Ibrahim, A. and Noushad Javed, M. and AlHajri, N. and Hamrouni, A. M. | Immune system response during viral Infections:Â Immunomodulators, cytokine storm (CS) and Immunotherapeutics in COVID-19 | 2021 | Incorrect study design |
| Hyrich, Kimme L and Machado, Pedro M | Rheumatic disease and COVID-19: epidemiology and outcomes | 2021 | Incorrect study design |
| Imai, R. and Ro, S. and Tomishima, Y. and Nishimura, N. | Steroid resistance and rebound phenomena in patients with COVID-19 | 2021 | Wrong population |
| Inciarte, A. and Gonzalez-Cordon, A. and Rojas, J. and Torres, B. and de Lazzari, E. and de la Mora, L. and Martinez-Rebollar, M. and Laguno, M. and Callau, P. and Gonzalez-Navarro, A. and Leal, L. and Garcia, F. and Mallolas, J. and Mosquera, M. and Marcos, M. A. and Ambrosioni, J. and Miro, J. M. and Martinez, E. and Blanco, J. L. | Clinical characteristics, risk factors, and incidence of symptomatic coronavirus disease 2019 in a large cohort of adults living with HIV: a single-center, prospective observational study | 2020 | Single centre |
| Ioannidis, John PA and Axfors, Cathrine and Contopoulos-Ioannidis, Despina G | Population-level COVID-19 mortality risk for non-elderly individuals overall and for non-elderly individuals without underlying diseases in pandemic epicenters | 2020 | Wrong population |
| Iovino, L. and Thur, L. A. and Gnjatic, S. and Chapuis, A. and Milano, F. and Hill, J. A. | Shared inflammatory pathways and therapeutic strategies in COVID-19 and cancer immunotherapy | 2021 | Insufficient original data |
| Iyer, A. and Shah, J. and Shah, R. | The burden and characteristics of HIV-infected COVID-19 patients at a tertiary care hospital in sub-Saharan Africa-A retrospective cohort study | 2022 | Single centre |
| Izudi, J. and Kiragga, A. N. and Kalyesubula, P. and Okoboi, S. and Castelnuovo, B. | Effect of the COVID-19 pandemic restrictions on outcomes of HIV care among adults in Uganda | 2022 | Wrong outcome |
| J and aghi, P. and Hosseini, Z. and Chilibeck, P. and Hanley, A. J. and Deguire, J. R. and B and y, B. and Pahwa, P. and Vatanparast, H. | The Role of Immunomodulatory Nutrients in Alleviating Complications Related to SARS-CoV-2: A Scoping Review | 2021 | Wrong population |
| Jakharia, N. and Subramanian, A. K. and Shapiro, A. E. | COVID-19 in the Immunocompromised Host, Including People with Human Immunodeficiency Virus | 2022 | Insufficient original data |
| Jamil, Z. and Almajhdi, F. N. and Khalid, S. and Asghar, M. and Ahmed, J. and Waheed, Y. | Comparison of Low-Versus High-Dose Steroids in the Clinical Outcome of Hospitalized COVID-19 Patients | 2021 | Wrong population |
| Janapala, R. N. and Patel, J. and Belfaqeeh, O. and Alhashmi, A. and Pourm and , A. | Letter to the Editor Regarding Combination of Tocilizumab and Steroids to Improve Mortality in Patients with Severe COVID-19 Infection: A Spanish, Multicenter, Cohort Study | 2021 | Wrong population |
| Jassat, W. and Cohen, C. and Tempia, S. and Masha, M. and Goldstein, S. and Kufa, T. and Murang and i, P. and Savulescu, D. and Walaza, S. and Bam, J. L. and Davies, M. A. and Prozesky, H. W. and Naude, J. and Mnguni, A. T. and Lawrence, C. A. and Mathema, H. T. and Zamparini, J. and Black, J. and Mehta, R. and Parker, A. and Chikobvu, P. and Dawood, H. and Muvhango, N. and Strydom, R. and Adelekan, T. and Mdlovu, B. and Moodley, N. and Namavh and u, E. L. and Rheeder, P. and Venturas, J. and Magula, N. and Blumberg, L. | Risk factors for COVID-19-related in-hospital mortality in a high HIV and tuberculosis prevalence setting in South Africa: a cohort study | 2021 | Single centre |
| Jee, J. and Stonestrom, A. J. and Devlin, S. and Nguyentran, T. and Wills, B. and Narendra, V. and Foote, M. B. and Lumish, M. and Vardhana, S. A. and Pastores, S. M. and Korde, N. and Patel, D. and Horwitz, S. and Scordo, M. and Daniyan, A. F. | Oncologic immunomodulatory agents in patients with cancer and COVID-19 | 2021 | Single centre |
| Jefferis, K. and Avalos, A. and Phillips, H. and Mmelesi, M. and Ramaabya, D. and Nkomo, B. and Muthoga, C. and Jarvis, J. N. and Ratladi, S. and Selato, R. and Stover, J. | Five years after Treat All implementation: Botswana's HIV response and future directions in the era of COVID-19 | 2021 | Wrong population |
| Jefferson, Tom and Spencer, Elizabeth A and Rosca, Elena Cecilia and Maltoni, Susanna and Brassey, Jon and Onakpoya, Igho and Pluddemann, Annette and Evans, David H and Conly, John M and Heneghan, Carl | Viral cultures, Polymerase Chain Reaction Cycle Threshold Values and Viral Load Estimation for SARS-CoV-2 Infectious Potential Assessment in Hematopoietic Stem Cell and Solid Organ Transplant Patients: A Systematic Review | 2022 | Wrong outcome |
| Jewell, B. L. and Mudimu, E. and Stover, J. and Ten Brink, D. and Phillips, A. N. and Smith, J. A. and Martin-Hughes, R. and Teng, Y. and Glaubius, R. and Mahiane, S. G. and Bansi-Matharu, L. and Taramusi, I. and Chagoma, N. and Morrison, M. and Doherty, M. and Marsh, K. and Bershteyn, A. and Hallett, T. B. and Kelly, S. L. | Potential effects of disruption to HIV programmes in sub-Saharan Africa caused by COVID-19: results from multiple mathematical models | 2020 | Wrong outcome |
| Jewell, B. L. and Smith, J. A. and Hallett, T. B. | Understanding the impact of interruptions to HIV services during the COVID-19 pandemic: A modelling study | 2020 | Wrong outcome |
| Jin, Y. H. and Jeon, S. and Lee, J. and Kim, S. and Jang, M. S. and Park, C. M. and Song, J. H. and Kim, H. R. and Kwon, S. | Broad Spectrum Antiviral Properties of Cardiotonic Steroids Used as Potential Therapeutics for Emerging Coronavirus Infections | 2021 | Wrong population |
| Jodele, S. and KÃ¶hl, J. | Tackling COVID-19 infection through complement-targeted immunotherapy | 2021 | Wrong population |
| John, B. V. and Deng, Y. and Khakoo, N. S. and Taddei, T. H. and Kaplan, D. E. and Dahman, B. | COVID-19 Vaccination Is Associated with Reduced SARS CoV2 Infection and Death in Liver Transplant Recipients | 2021 | Incorrect study design |
| John, N. A. and John, J. E. | Implication of COVID-19 in patients of HIV with hepatitis C | 2022 | Compound risk group |
| Jones, Jessica M and Faruqi, Aiman J and Sullivan, James K and Calabrese, Cass and ra and Calabrese, Leonard H | COVID-19 outcomes in patients undergoing B cell depletion therapy and those with humoral immunodeficiency states: A scoping review | 2021 | Incorrect study design |
| Jones, Rachael and Nelson, Mark and Bracchi, Margherita and Asboe, David and Boffito, Marta | COVID-19 in patients with HIV | 2020 | Incorrect study design |
| Jou, E. and Zhou, A. K. and Ho, J. S. Y. and Thahir, A. | Perioperative use of intra-articular steroids during the COVID-19 pandemic | 2022 | Wrong population |
| Jou, J. and Gupta, M. | A Single-Center Report of Mortality Comparison of COVID-19 Vaccinated Kidney Transplant Recipients | 2022 | Wrong outcome |
| Jung, C. and Wernly, B. and FjÃ¸lner, J. and Bruno, R. R. and Dudzinski, D. and Artigas, A. and Bollen Pinto, B. and Schefold, J. C. and Wolff, G. and Kelm, M. and Beil, M. and Sigal, S. and van Heerden, P. V. and Szczeklik, W. and Czuczwar, M. and Elhadi, M. and Joannidis, M. and Oeyen, S. and Zafeiridis, T. and Marsh, B. and Andersen, F. H. and Moreno, R. and Cecconi, M. and Leaver, S. and Boumendil, A. and De Lange, D. W. and Guidet, B. and Flaatten, H. | Steroid use in elderly critically ill COVID-19 patients | 2021 | Wrong population |
| Jung, Y. and Kwon, M. and Choi, H. G. | Association between previous rheumatoid arthritis and COVID-19 and its severity: a nationwide cohort study in South Korea | 2021 | n<50 |
| Junker, A. | COVID-19 patients with bronchial tumors: Chemotherapy leads to a higher mortality risk. [German] | 2020 | Foreign language |
| Kaddam, L. and Babiker, R. and Ali, S. and Satti, S. and Ali, N. and Elamin, M. and Mukhtar, M. and Elnimeiri, M. and Saeed, A. | Potential Role of Acacia Senegal (Gum Arabic) as Immunomodulatory Agent among newly diagnosed COVID 19 Patients: A structured summary of a protocol for a randomised, controlled, clinical trial | 2020 | Wrong population |
| Kajova, M. and KekÃ¤lÃ¤inen, E. and Anttila, V. J. and Paajanen, J. | Successful treatment with a short course of remdesivir in a case of prolonged COVID-19 in a lymphoma patient | 2022 | n<50 |
| Kalidhindi, R. S. R. and Borkar, N. A. and Ambhore, N. S. and Pabelick, C. M. and Prakash, Y. S. and Sathish, V. | Sex steroids skew ACE2 expression in human airway: a contributing factor to sex differences in COVID-19? | 2020 | Wrong population |
| Kalil, A. C. and Florescu, D. F. | Mortality in solid organ transplant recipients hospitalized for COVID-19 | 2021 | Incorrect study design |
| Kalil, A. C. and Stebbing, J. | Baricitinib: the first immunomodulatory treatment to reduce COVID-19 mortality in a placebo-controlled trial | 2021 | Wrong population |
| Kamath, C. and Brenner, E. J. | The safe use of inflammatory bowel disease therapies during the COVID-19 pandemic | 2022 | Duplicate |
| Kaplan, G. G. and Windsor, J. W. | The four epidemiological stages in the global evolution of inflammatory bowel disease | 2021 | Wrong outcome |
| Kara Polat, A. and Oguz Topal, I. and Karadag, A. S. and Aksoy, H. and Koku Aksu, A. E. and Ozkur, E. and Ozkok Akbulut, T. and Topaloglu Demir, F. and Engin, B. and Uzuncakmak, T. K. and KÄ±vanc Altunay, I. | The impact of COVID-19 in patients with psoriasis: A multicenter study in Istanbul | 2021 | Insufficient original data |
| Karakike, E. and Dalekos, G. N. and Koutsodimitropoulos, I. and Saridaki, M. and Pourzitaki, C. and Papathanakos, G. and Kotsaki, A. and Chalvatzis, S. and Dimakopoulou, V. and Vechlidis, N. and Paramythiotou, E. and Avgoustou, C. and Ioakeimidou, A. and Kouriannidi, E. and Komnos, A. and Neou, E. and Rovina, N. and Stefanatou, E. and Milionis, H. and Nikolaidis, G. and Koutsoukou, A. and Damoraki, G. and Dimopoulos, G. and Zoumpos, V. and Eugen-Olsen, J. and Akinosoglou, K. and Gatselis, N. K. and Koulouras, V. and Gkeka, E. and Markou, N. and Netea, M. G. and Giamarellos-Bourboulis, E. J. | ESCAPE: An Open-Label Trial of Personalized Immunotherapy in Critically lll COVID-19 Patients | 2022 | Wrong population |
| Karasneh, Reema A and Khassawneh, Basheer Y and Al-Azzam, Sayer and Al-Mistarehi, Abdel-Hameed and Lattyak, William J and Aldiab, Motasem and Kabbaha, Suad and Hasan, Syed Shahzad and Conway, Barbara R and Aldeyab, Mamoon A | Risk Factors Associated with Mortality in COVID-19 Hospitalized Patients: Data from the Middle East | 2022 | Wrong population |
| Karcher, D. S. | From HIV to Coronavirus Disease 2019 (COVID-19) | 2022 | Incorrect study design |
| Karmen-Tuohy, S. and Carlucci, P. M. and Zervou, F. N. and Zacharioudakis, I. M. and Rebick, G. and Klein, E. and Reich, J. and Jones, S. and Rahimian, J. | Outcomes Among HIV-Positive Patients Hospitalized With COVID-19 | 2020 | n<50 |
| Karthik, K. and Senthilkumar, T. M. A. and Udhayavel, S. and Raj, G. D. | Role of antibody-dependent enhancement (ADE) in the virulence of SARS-CoV-2 and its mitigation strategies for the development of vaccines and immunotherapies to counter COVID-19 | 2020 | Incorrect study design |
| Kassanjee, R. and Davies, M. A. and Ngwenya, O. and Osei-Yeboah, R. and Jacobs, T. and Morden, E. and Timmerman, V. and Britz, S. and Mendelson, M. and Taljaard, J. and Riou, J. and Boulle, A. and Tiffin, N. and Zinyakatira, N. | COVID-19 among adults living with HIV: Correlates of mortality in a general population in a resource-limited setting | 2022 | Duplicate |
| Kaswa, R. | The impact of the COVID-19 pandemic on accessing HIV care: A case report | 2021 | n<50 |
| Kavanagh, M. M. and Agbla, S. C. and Joy, M. and Aneja, K. and Pillinger, M. and Case, A. and Erondu, N. A. and Erkkola, T. and Graeden, E. | Law, criminalisation and HIV in the world: have countries that criminalise achieved more or less successful pandemic response? | 2021 | Wrong outcome |
| Kavanagh, M. M. and Katz, I. T. and Holmes, C. B. | Reckoning with mortality: global health, HIV, and the politics of data | 2020 | Incorrect study design |
| Kelleni, M. T. | NSAIDs/nitazoxanide/azithromycin repurposed for COVID-19: potential mitigation of the cytokine storm interleukin-6 amplifier via immunomodulatory effects | 2022 | Wrong population |
| Kelly, S. and Waters, L. and Cevik, M. and Collins, S. and Lewis, J. and Wu, M. S. and Blanchard, T. J. and Geretti, A. M. | Pneumocystis pneumonia, a COVID-19 mimic, reminds us of the importance of HIV testing in COVID-19 | 2020 | Insufficient original data |
| Kerkhoff, A. D. and Havlir, D. V. | CROI 2021: Tuberculosis, Opportunistic Infections, and COVID-19 Among People with HIV | 2021 | Incorrect study design |
| Khaba, M. C. and Ngale, T. C. and Madala, N. | COVID-19 in an HIV-infected patient. Lessons learned from an autopsy case | 2020 | n<50 |
| Khairallah, Pascale and Aggarwal, Nidhi and Awan, Ahmed A and Vangala, Ch and an and Airy, Medha and Pan, Jenny S and Murthy, Bhamidipati VR and Winkelmayer, Wolfgang C and Ramanathan, Venkat | The impact of COVID-19 on kidney transplantation and the kidney transplant recipientâ€“One year into the pandemic | 2021 | Incorrect study design |
| Khan, K. and Lustig, G. and Bernstein, M. and Archary, D. and Cele, S. and Karim, F. and Smith, M. and Ganga, Y. and Jule, Z. and Reedoy, K. and Miya, Y. and Mthabela, N. and Magula, N. P. and Lessells, R. and de Oliveira, T. and Gosnell, B. I. and Abdool Karim, S. and Garrett, N. and Hanekom, W. and Bekker, L. G. and Gray, G. and Blackburn, J. M. and Moosa, M. S. and Sigal, A. | Immunogenicity of Severe Acute Respiratory Syndrome Coronavirus 2 (SARS-CoV-2) Infection and Ad26.CoV2.S Vaccination in People Living With Human Immunodeficiency Virus (HIV) | 2022 | Wrong outcome |
| Khezri, M. R. and Zolbanin, N. M. and Ghasemnejad-Berenji, M. and Jafari, R. | Azithromycin: Immunomodulatory and antiviral properties for SARS-CoV-2 infection | 2021 | Wrong population |
| Khokher, W. and Beran, A. and Iftikhar, S. and Malhas, S. E. and Srour, O. and Mhanna, M. and Bhuta, S. and Patel, D. and Kesireddy, N. and Burmeister, C. and Borchers, E. and Assaly, R. and Safi, F. | Pulse versus nonpulse steroid regimens in patients with coronavirus disease 2019: A systematic review and meta-analysis | 2022 | Wrong population |
| Kjeldsen, S. and Nielsen, J. and Mertz NÃ¸rgÃ¥rd, B. and Kjeldsen, J. | Mesalazine in Inflammatory Bowel Disease and COVID-19: Hospitalization and Adverse In-Hospital Outcomes Based on Nationwide Data | 2022 | Paediatric data |
| Koc, S. and Hanikoglu, F. and Dokur, M. and Polat, Y. and Celebi, S. and Koc, S. G. and Kupeli, I. and Uysal, H. | Comparison of Cytokine Hemadsorption as an Immunomodulator Therapy in COVID-19 Patients with and without Bacterial Sepsis | 2022 | Wrong population |
| Korsukewitz, C. and Reddel, S. W. and Bar-Or, A. and Wiendl, H. | Neurological immunotherapy in the era of COVID-19 - looking for consensus in the literature | 2020 | Wrong population |
| Koti, M. and Morales, A. and Graham, C. H. and Siemens, D. R. | BCG vaccine and COVID-19: implications for infection prophylaxis and cancer immunotherapy | 2020 | Wrong population |
| Kowalska, J. D. and BieÅ„kowski, C. and Fleischhans, L. and Antoniak, S. and Skrzat-KlapaczyÅ„ska, A. and Suchacz, M. and Bogdanic, N. and Gokengin, D. and Oprea, C. and Karpov, I. and Kase, K. and Matulionyte, R. and Papadopoulos, A. and Rukhadze, N. and Harxhi, A. and Jilich, D. and Lakatos, B. and Sedlacek, D. and Dragovic, G. and Vasylyev, M. and Verhaz, A. and Yancheva, N. and Begovac, J. and Horban, A. | The Presence of Either Typical or Atypical Radiological Changes Predicts Poor COVID-19 Outcomes in HIV-Positive Patients from a Multinational Observational Study: Data from Euroguidelines in Central and Eastern Europe Network Group | 2022 | Wrong outcome |
| Kuczaj, A. and Zakliczynski, M. and Przybylowski, P. and Zembala, M. and Hrapkowicz, T. | COVID-19 mortality in patients after orthotopic heart transplantation: A single-center one-year observational study | 2021 | Single centre |
| Kulkarni, Padmaj S and Das, Kunal and Agrawal, Nitika and Kala, Mansi and Kh and uri, Rakhee and Parikh, Purvish M | The Interplay between COVID-19 and Cancer: Challenges and Perspectives | 2020 | Incorrect study design |
| Kulkarni, S. and Fisk, M. and Kostapanos, M. and Banham-Hall, E. and Bond, S. and Hernan-Sancho, E. and Norton, S. and Cheriyan, J. and Cope, A. and Galloway, J. and Hall, F. and Jayne, D. and Wilkinson, I. B. | Repurposed immunomodulatory drugs for Covid-19 in pre-ICu patients - mulTi-Arm Therapeutic study in pre-ICu patients admitted with Covid-19 - Repurposed Drugs (TACTIC-R): A structured summary of a study protocol for a randomised controlled trial | 2020 | Wrong population |
| Kumar, A. and Sharma, A. and Tirpude, N. V. and Sharma, S. and Padwad, Y. S. and Kumar, S. | Pharmaco-immunomodulatory interventions for averting cytokine storm-linked disease severity in SARS-CoV-2 infection | 2022 | Wrong population |
| Kumar, V. | Toll-like receptors in sepsis-associated cytokine storm and their endogenous negative regulators as future immunomodulatory targets | 2020 | Wrong population |
| Kumric, M. and Ticinovic Kurir, T. and Martinovic, D. and Zivkovic, P. M. and Bozic, J. | Impact of the COVID-19 pandemic on inflammatory bowel disease patients: A review of the current evidence | 2021 | Incorrect study design |
| Kuno, T. and Miyamoto, Y. and Iwagami, M. and Ishimaru, M. and Takahashi, M. and Egorova, N. N. | The association of remdesivir and in-hospital outcomes for COVID-19 patients treated with steroids | 2021 | Wrong population |
| Kuno, T. and Sahashi, Y. and Kawahito, S. and Takahashi, M. and Iwagami, M. and Egorova, N. N. | Prediction of in-hospital mortality with machine learning for COVID-19 patients treated with steroid and remdesivir | 2021 | Incorrect study design |
| Kuno, T. and So, M. and Takahashi, M. and Egorova, N. N. | Prophylactic versus therapeutic anticoagulation for survival of patients with COVID-19 on steroid | 2022 | Wrong outcome |
| Lafont, E. and Pere, H. and Lebeaux, D. and Cheminet, G. and Thervet, E. and Guillemain, R. and Flahault, A. | Targeted SARS-CoV-2 treatment is associated with decreased mortality in immunocompromised patients with COVID-19 | 2022 | Single centre |
| Lai, Alvina G and Pasea, Laura and Banerjee, Amitava and Denaxas, Spiros and Katsoulis, Michail and Chang, Wai Hoong and Williams, Bryan and Pillay, Deenan and Noursadeghi, Mahdad and Linch, David | Estimating excess mortality in people with cancer and multimorbidity in the COVID-19 emergency | 2020 | Compound risk group |
| Lai, Y. J. and Chang, H. S. and Yang, Y. P. and Lin, T. W. and Lai, W. Y. and Lin, Y. Y. and Chang, C. C. | The role of micronutrient and immunomodulation effect in the vaccine era of COVID-19 | 2021 | Wrong population |
| Lamouche-Wilquin, P. and Souchard, J. and Pere, M. and Raymond, M. and Asfar, P. and Darreau, C. and Reizine, F. and Hourmant, B. and Colin, G. and Rieul, G. and Kergoat, P. and FrÃ©rou, A. and Lorber, J. and Auchabie, J. and La Combe, B. and Seguin, P. and Egreteau, P. Y. and Morin, J. and Fedun, Y. and Canet, E. and Lascarrou, J. B. and Delbove, A. | Early steroids and ventilator-associated pneumonia in COVID-19-related ARDS | 2022 | Wrong population |
| Laracy, J. and Zucker, J. and Castor, D. and McMahon, D. J. and Guo, T. W. and Yan, M. and Shalev, N. and Scherer, M. and Gordon, P. and Sobieszczyk, M. and Yin, M. T. | HIV-1 Infection Does Not Change Disease Course or Inflammatory Pattern of SARS-CoV-2-Infected Patients Presenting at a Large Urban Medical Center in New York City | 2021 | Single centre |
| Laurenge, A. and Ursu, R. and Houillier, C. and Abdi, B. and Tebano, G. and Quemeneur, C. and Choquet, S. and Di Blasi, R. and Lozano, F. and Morales, A. and DurÃ¡n-PeÃ±a, A. and Sirven-Villaros, L. and Mathon, B. and Mokhtari, K. and Bielle, F. and Martin-Duverneuil, N. and Delattre, J. Y. and Marcelin, A. G. and Pourcher, V. and Alentorn, A. and Idbaih, A. and Carpentier, A. F. and Leblond, V. and Hoang-Xuan, K. and Touat, M. | SARS-CoV-2 infection in patients with primary central nervous system lymphoma | 2021 | n<50 |
| Leach, D. A. and Brooke, G. N. and Bevan, C. L. | Roles of steroid receptors in the lung and COVID-19 | 2021 | Insufficient original data |
| Lee, K. Y. and Rhim, J. W. and Kang, J. H. | Immunopathogenesis of COVID-19 and early immunomodulators | 2020 | Insufficient original data |
| Lee, L. and Starkey, T. and Cazier, J. B. and Kerr, R. and Middleton, G. | COVID-19 mortality in hospitalized cancer patients is not significantly affected by chemotherapy or other anti-cancer treatments | 2020 | Insufficient original data |
| Li Marzi, V. and Campi, R. and Pecoraro, A. and Greco, I. and Caroti, L. and Cirami, C. L. and Giancane, S. and Lazzeri, C. and Tuccio, A. and Vignolini, G. and Migliaccio, M. L. and Peris, A. and Serni, S. | Feasibility and safety of a kidney transplantation program from donors after brain death during the COVID-19 pandemic: insights from an Italian academic centre | 2020 | Wrong population |
| Li, J. P. and Wu, K. H. and Chao, W. R. and Lee, Y. J. and Yang, S. F. and Chao, Y. H. | Immunomodulation of Mesenchymal Stem Cells in Acute Lung Injury: From Preclinical Animal Models to Treatment of Severe COVID-19 | 2022 | Insufficient original data |
| Lin, H. S. and Lin, X. H. and Wang, J. W. and Wen, D. N. and Xiang, J. and Fan, Y. Q. and Li, H. D. and Wu, J. and Lin, Y. and Lin, Y. L. and Sun, X. R. and Chen, Y. F. and Chen, C. J. and Lian, N. F. and Xie, H. S. and Lin, S. H. and Xie, Q. F. and Li, C. W. and Peng, F. Z. and Wang, N. and Lin, J. Q. and Chen, W. J. and Huang, C. L. and Fu, Y. | Exhausting T Cells During HIV Infection May Improve the Prognosis of Patients with COVID-19 | 2021 | Wrong population |
| Lin, H. Y. | The severe COVID-19: A sepsis induced by viral infection? And its immunomodulatory therapy | 2020 | Wrong population |
| Linares, L. and Cofan, F. and Diekmann, F. and Herrera, S. and Marcos, M. A. and Castel, M. A. and Farrero, M. and Colmenero, J. and Ruiz, P. and Crespo, G. and Llopis, J. and Garcia-Vidal, C. and Soriano, A. and Moreno, A. and Bodro, M. and Albiac, L. and Aguero, D. and Ambrosioni, J. and Blanco, J. L. and Cardozo, C. and Chumbita, M. and De La Mora, L. and Garcia-Alcaide, F. and Garcia-Pouton, N. and Gonzalez-Cordon, A. and Hern and ez-Meneses, M. and Inciarte, A. and Laguno, M. and Leal, L. and MacAya, I. and Mallolas, J. and Martinez, E. and Martinez, M. and Meira, F. and Miro, J. M. and Mensa, J. and Moreno-Martinez, A. and Moreno-Garcia, E. and Morata, L. and Martinez, J. A. and Puerta-Alcalde, P. and Rico, V. and Rojas, J. and Sola, M. and Torres, B. and Torres, M. and Garcia, A. and Perez-Villa, F. and Navasa, M. and Bayes, B. and Cucchiari, D. and Esforzado, N. and Guillen, E. and Molina, A. and Montagud-Marrahi, E. and Oppenheimer, F. and Pineiro, G. J. and Poch, E. and Revuelta, I. and Rodas, L. and Torregrosa, J. V. and Ugalde-Altamirano, J. and Ventura-Aguiar, P. and Hurtado, J. C. and Fern and ez, M. and Mosquera, M. M. | A propensity score-matched analysis of mortality in solid organ transplant patients with COVID-19 compared to non-solid organ transplant patients | 2021 | n<50 |
| Ling, K. L. and Hilmi, I. and Raja Ali, R. A. and Leong, R. W. L. and Leung, W. K. and Ng, S. C. and Wu, K. C. and Chen, M. H. and Ran, Z. H. and Hisamatsu, T. and Ahuja, V. and Makharia, G. K. and Banerjee, R. and Wei, S. C. and Wu, D. C. and Pisespongsa, P. and Ye, B. D. and Sollano, J. and Simadibrata, M. and Chuah, S. W. and Ooi, C. J. | Asian Pacific Association of Gastroenterology (APAGE) Inflammatory Bowel Disease (IBD) Working Party guidelines on IBD management during the COVID-19 pandemic | 2020 | Insufficient original data |
| Lipsa, A. and Prabhu, J. S. | Gender disparity in COVID-19: Role of sex steroid hormones | 2021 | Wrong population |
| Liu, N. and Jiang, C. and Cai, P. and Shen, Z. and Sun, W. and Xu, H. and Fang, M. and Yao, X. and Zhu, L. and Gao, X. and Fang, J. and Lin, J. and Guo, C. and Qu, K. | Single-cell analysis of COVID-19, sepsis, and HIV infection reveals hyperinflammatory and immunosuppressive signatures in monocytes | 2021 | Wrong outcome |
| Liu, Y. and Han, J. and Li, X. and Chen, D. and Zhao, X. and Qiu, Y. and Zhang, L. and Xiao, J. and Li, B. and Zhao, H. | COVID-19 Vaccination in People Living with HIV (PLWH) in China: A Cross Sectional Study of Vaccine Hesitancy, Safety, and Immunogenicity | 2021 | Wrong outcome |
| Llamas-Velasco, M. and Ovejero-Merino, E. and Salgado-Boquete, L. | [Obesity - A Risk Factor for Psoriasis and COVID-19] | 2021 | Incorrect study design |
| Logan, A. T. and Davis, N. and Delk, I. and Hassett, L. and Olson, S. and Patel, K. | Characterization of Lung Transplant COVID19+ Patients and Mortality Outcomes | 2022 | n<50 |
| Loke, X. Y. and Imran, S. A. M. and Tye, G. J. and Wan Kamarul Zaman, W. S. and Nordin, F. | Immunomodulation and Regenerative Capacity of MSCs for Long-COVID | 2021 | Wrong population |
| Lopez, V. and Casas-Gonzalez, C. and Vazquez, T. and Cabello, M. and Poveda, I. and Hern and ez, D. | Predictors of mortality in kidney transplant patients infected by SARS-CoV-2 in south of Spain | 2021 | Single centre |
| Lucchini, Giovanna and Cozma, Elena and Jackson, Aimee and Gilmour, Kimberly and Protheroe, Rachel Elizabeth and Tholouli, Eleni and Wynn, Robert F and Wilson, Keith and Peggs, Karl S and Potter, Victoria | COVID-19 Infection of HSCT Recipients Is Associated with High Mortality but No Detectable Cytokine Storm at Presentation | 2021 | Paediatric data |
| Ludvigsson, J. F. and Axelrad, J. and Halfvarson, J. and Khalili, H. and Larsson, E. and Lochhead, P. and Roelstraete, B. and Simon, T. G. and SÃ¶derling, J. and OlÃ©n, O. | Inflammatory bowel disease and risk of severe COVID-19: A nationwide population-based cohort study in Sweden | 2021 | Paediatric data |
| Luis, B. M. and Miguel, M. B. and Pedro, D. L. and David, I. P. and Itziar, A. and Ana, G. H. and Enrique, I. J. and MarÃ­a, L. V. and Noelia, T. F. and Julio CÃ©sar, B. B. and Marta, U. I. and Rodrigo, S. L. and MarÃ­a, C. B. and AndrÃ©s, L. M. and Javier, M. I. and Juan Pablo, G. M. and Gerardo, H. F. and Carolina, N. F. and Jorge, B. L. and MarÃ­a, F. R. and Fern and o, C. T. and Sergio, O. E. and Lourdes, F. C. and MarÃ­a, G. E. and Gregoria, M. L. and Adolfo, S. R. and JosÃ© Antonio, F. R. | Benefits of early aggressive immunomodulatory therapy (tocilizumab and methylprednisolone) in COVID-19: Single center cohort study of 685 patients | 2021 | Wrong population |
| Lund, L. C. and Kristensen, K. B. and Reilev, M. and Christensen, S. and Thomsen, R. W. and Christiansen, C. F. and StÃ¸vring, H. and Johansen, N. B. and Brun, N. C. and Hallas, J. and PottegÃ¥rd, A. | Adverse outcomes and mortality in users of non-steroidal anti-inflammatory drugs who tested positive for SARS-CoV-2: A Danish nationwide cohort study | 2020 | Wrong population |
| Luque-Paz, D. and Sesques, P. and Wallet, F. and Bachy, E. and Ader, F. | The burden of SARS-CoV-2 in patients receiving chimeric antigen receptor T cell immunotherapy: everything to lose | 2022 | Insufficient original data |
| Lustig, G. and Ganga, Y. and Rodel, H. and Tegally, H. and Jackson, L. and Cele, S. and Khan, K. and Jule, Z. and Reedoy, K. and Karim, F. and Bernstein, M. and Moosa, M. S. and Archary, D. and de Oliveira, T. and Lessells, R. and Abdool Karim, S. S. and Sigal, A. | SARS-CoV-2 evolves increased infection elicited cell death and fusion in an immunosuppressed individual | 2022 | Insufficient original data |
| Luzzati, R. and De Luca, M. and Sanson, G. and Borelli, M. and Biolo, G. and Giacomazzi, D. and Zerbato, V. and Di Bella, S. | Potential of outpatient steroid therapy in elderly patients with early COVID-19 | 2022 | Wrong population |
| Lyons-Weiler, J. | Pathogenic priming likely contributes to serious and critical illness and mortality in COVID-19 via autoimmunity | 2020 | Wrong population |
| M and ala, W. L. and Liu, M. K. P. | SARS-CoV-2 and HIV-1: Should HIV-1-Infected Individuals in Sub-Saharan Africa Be Considered a Priority Group for the COVID-19 Vaccines? | 2021 | Wrong population |
| Maddox, J. | Risk Factors and Outcomes of Patients with Haematological Malignancy and Covid-19: Ongoing Reduction in Mortality during the 3rd Wave of the Pandemic | 2022 | Insufficient original data |
| Madhi, S. A. and Moodley, D. and Hanley, S. and Archary, M. and Hoosain, Z. and Lalloo, U. and Louw, C. and Fairlie, L. and Fouche, L. F. and Masilela, M. S. L. and Singh, N. and Grobbelaar, C. and Ahmed, K. and BenadÃ©, G. and Bhikha, S. and Bhorat, A. E. and Bhorat, Q. and Joseph, N. and Dheda, K. and Esmail, A. and Foulkes, S. and Goga, A. and Oommen Jose, A. and Kruger, G. and Kalonji, D. J. and Lalloo, N. and Lombaard, J. J. and Lombard Koen, A. and Kany Luabeya, A. and Mngqibisa, R. and Petrick, F. G. and Pitsi, A. and Tameris, M. and Thombrayil, A. and Vollgraaff, P. L. and Cloney-Clark, S. and Zhu, M. and Bennett, C. and Albert, G. and Faust, E. and Plested, J. S. and Fries, L. and Robertson, A. and Neal, S. and Cho, I. and Glenn, G. M. and Shinde, V. | Immunogenicity and safety of a SARS-CoV-2 recombinant spike protein nanoparticle vaccine in people living with and without HIV-1 infection: a randomised, controlled, phase 2A/2B trial | 2022 | Wrong outcome |
| Madzima, B. and Makoni, T. and Mugurungi, O. and Mudariki, G. and Mpofu, A. and Dube, F. and Munangaidzwa, L. and Taramusi, I. | The impact of the COVID-19 pandemic on people living with HIV in Zimbabwe | 2022 | Insufficient original data |
| Maggi, P. and Ricci, E. and Messina, V. and Salzillo, A. and Simeone, F. and Iodice, A. and Socio, G. V. | Dangerous liaisons? The role of inflammation and comorbidities in HIV and SARS-CoV-2 infection | 2021 | Incorrect study design |
| Maggiolo, F. and Zoboli, F. and Arosio, M. and Valenti, D. and Guarneri, D. and Sangiorgio, L. and Ripamonti, D. and Callegaro, A. | SARS-CoV-2 infection in persons living with HIV: A single center prospective cohort | 2021 | Insufficient original data |
| Maggiore, U. and Riella, L. V. and Azzi, J. and Cravedi, P. | Mortality in solid organ transplant recipients with COVID-19: More than meets the eye | 2021 | Wrong population |
| Mahale, N. and Rajhans, P. and Godavarthy, P. and Narasimhan, V. L. and Oak, G. and Marreddy, S. and Bedekar, A. and Dhundi, U. and Pawar, H. S. and Akole, P. and Pawar, B. and Bhurke, B. and Chavan, S. and Prayag, P. and Pur and are, B. and Dalvi, P. and Telbhare, V. and Marudwar, P. and Diwane, D. and Shahane, M. and Prayag, A. and Gugale, S. and Bhor, S. and Jog, S. | A Retrospective Observational Study of Hypoxic COVID-19 Patients Treated with Immunomodulatory Drugs in a Tertiary Care Hospital | 2020 | Wrong population |
| Mahalingasivam, Viyaasan and Craik, Alison and Tomlinson, Laurie A and Ge, Long and Hou, Liangying and Wang, Qi and Yang, Kehu and Fogarty, Damian G and Keenan, Ciara | A systematic review of COVID-19 and kidney transplantation | 2021 | Insufficient original data |
| Mahase, E. | Covid-19: Low dose steroid cuts death in ventilated patients by one third, trial finds | 2020 | Wrong population |
| Mahendraraj, K. and Kim, I. and Todo, T. and Brennan, T. and Nissen, N. and Kosari, K. and Voidonikolas, G. and Ramzy, D. | Mortality from fulminant myocarditis in multi-organ transplant recipient with COVID-19 | 2021 | n<50 |
| Mahooti, M. and Miri, S. M. and Abdolalipour, E. and Ghaemi, A. | The immunomodulatory effects of probiotics on respiratory viral infections: A hint for COVID-19 treatment? | 2020 | Wrong population |
| Malek, Alex and re E and Adachi, Javier A and Mulanovich, Victor E and Sassine, Joseph and Raad, Issam I and McConn, Kelly and Seiler, Garret T and Dhal, Udit and Khawaja, Fareed and Chemaly, Roy F | Immune reconstitution and severity of COVID-19 among hematopoietic cell transplant recipients | 2021 | n<50 |
| Mallis, P. and Michalopoulos, E. and Chatzistamatiou, T. and Stavropoulos-Giokas, C. | Mesenchymal stromal cells as potential immunomodulatory players in severe acute respiratory distress syndrome induced by SARS-CoV-2 infection | 2020 | Wrong population |
| Mamode, Nizam and Ahmed, Zubir and Jones, Gareth and Banga, Neal and Motallebzadeh, Reza and Tolley, Hannah and Marks, Steve and Stojanovic, Jelena and Khurram, Muhammad A and Thuraisingham, Raj | Mortality rates in transplant recipients and transplantation candidates in a high-prevalence COVID-19 environment | 2021 | Paediatric data |
| Manganiello, C. F. and Basbus, L. and Callegari, M. S. and Cayol, F. and Amaral De Sousa, C. R. and Porta, R. E. | [Immunotherapy with anti-SARS-COV-2 neutralizing F(ab')2 antibodies from equine serum in the treatment of outpatients with bilateral COVID-19 pneumonia] | 2022 | Wrong population |
| Manna, S. and Chowdhury, T. and Chakraborty, R. and M and al, S. M. | Probiotics-Derived Peptides and Their Immunomodulatory Molecules Can Play a Preventive Role Against Viral Diseases Including COVID-19 | 2021 | Wrong population |
| MarÃ­n-JimÃ©nez, I. and Zabana, Y. and RodrÃ­guez-Lago, I. and MarÃ­n, L. and Barreiro-de Acosta, M. and Esteve, M. | COVID-19 and inflammatory bowel disease: questions arising from patient care and follow-up during the initial phase of the pandemic (February-April 2020) | 2020 | Wrong outcome |
| Market, M. and Angka, L. and Martel, A. B. and Bastin, D. and Olanubi, O. and Tennakoon, G. and Boucher, D. M. and Ng, J. and Ardolino, M. and Auer, R. C. | Flattening the COVID-19 Curve With Natural Killer Cell Based Immunotherapies | 2020 | Wrong population |
| Martinez-Lopez, J and Hern and ez-Ibarburu, G and Alonso, R and Sanchez-Pina, JM and Zamanillo, I and Lopez-MuÃ±oz, N and IÃ±iguez, Rodrigo and Cuellar, C and Calbacho, M and Paciello, ML | Impact of COVID-19 in patients with multiple myeloma based on a global data network | 2021 | Indirect mortality |
| Martinez-Urbistondo, M. and Gutierrez-Rojas, A. and Andres, A. and Gutierrez, I. and Escudero, G. and Garcia, S. and Gutierrez, A. and Sanchez, E. and Herraiz, J. and De La Fuente, S. and Callejas, A. and De Mendoza, C. and Moreno-Torres, V. | Severe Lymphopenia as a Predictor of COVID-19 Mortality in Immunosuppressed Patients | 2021 | Wrong population |
| Mashinchi, B. and Aryannejad, A. and Namazi, M. and Moradi, S. and Masoumi, Z. and Parsaei, A. and Masoumi, M. | A Case of C-ANCA Positive Systematic Lupus Erythematous and ANCA-Associated Vasculitis Overlap Syndrome Superimposed by COVID-19: A Fatal Trio | 2021 | n<50 |
| Mason, A. and Anver, H. and Lwin, M. and Holroyd, C. and Faust, S. N. and Edwards, C. J. | Lupus, vaccinations and COVID-19: What we know now | 2021 | Incorrect study design |
| Mason, A. and Rose, E. and Edwards, C. J. | Clinical management of Lupus patients during the COVID-19 pandemic | 2020 | Wrong outcome |
| Massarvva, T. | Clinical outcomes of COVID-19 amongst HIV patients: a systematic literature review | 2021 | Paediatric data |
| Matsuda, E. M. and Oliveira, I. P. and Campos, I. B. and Ahagon, C. M. and Castejon, M. J. and Silva, V. O. and Manzoni, F. M. and LÃ³pez-Lopes, G. I. and BrÃ­gido, L. F. M. | SARS-CoV-2 testing among patients and healthcare professionals in an HIV outpatient clinic in Brazil | 2022 | Wrong outcome |
| Matucci-Cerinic, M. and Bruni, C. and Allanore, Y. and Clementi, M. and Dagna, L. and Damjanov, N. S. and de Paulis, A. and Denton, C. P. and Distler, O. and Fox, D. and Furst, D. E. and Khanna, D. and Krieg, T. and Kuwana, M. and Lee, E. B. and Li, M. and Pillai, S. and Wang, Y. and Zeng, X. and Taliani, G. | Systemic sclerosis and the COVID-19 pandemic: World Scleroderma Foundation preliminary advice for patient management | 2020 | Incorrect study design |
| Mauvais-Jarvis, F. and Klein, S. L. and Levin, E. R. | Estradiol, Progesterone, Immunomodulation, and COVID-19 Outcomes | 2020 | Incorrect study design |
| Mazzitelli, M. and Trunfio, M. and Sasset, L. and Leoni, D. and Castelli, E. and Lo Menzo, S. and Gardin, S. and Putaggio, C. and Brundu, M. and Garzotto, P. and Cattelan, A. M. | Factors Associated with Severe COVID-19 and Post-Acute COVID-19 Syndrome in a Cohort of People Living with HIV on Antiretroviral Treatment and with Undetectable HIV RNA | 2022 | Wrong outcome |
| McAllister, M. J. and Kirkwood, K. and Chuah, S. C. and Thompson, E. J. and Cartwright, J. A. and Russell, C. D. and Dorward, D. A. and Lucas, C. D. and Ho, G. T. | Intestinal Protein Characterisation of SARS-CoV-2 Entry Molecules ACE2 and TMPRSS2 in Inflammatory Bowel Disease (IBD) and Fatal COVID-19 Infection | 2022 | Wrong outcome |
| Mehta, P. and Gasparyan, A. Y. and Zimba, O. and Kitas, G. D. | Systemic lupus erythematosus in the light of the COVID-19 pandemic: infection, vaccination, and impact on disease management | 2022 | Insufficient original data |
| Mehta, S. A. and Rana, M. M. and Motter, J. D. and Small, C. B. and Pereira, M. R. and Stosor, V. and Elias, N. and Haydel, B. and Florman, S. and Odim, J. and Morsheimer, M. and Robien, M. and Massie, A. B. and Brown, D. and Boyarsky, B. J. and Garonzik-Wang, J. and Tobian, A. A. R. and Werbel, W. A. and Segev, D. L. and Dur and , C. M. | Incidence and Outcomes of COVID-19 in Kidney and Liver Transplant Recipients With HIV: Report From the National HOPE in Action Consortium | 2021 | n<50 |
| MelgaÃ§o, J. G. and Brito, E. Cunha D. and Azamor, T. and da Silva, A. M. V. and TubarÃ£o, L. N. and GonÃ§alves, R. B. and Monteiro, R. Q. and Missailidis, S. and da Costa Neves, P. C. and Ano Bom, A. P. D. | Cellular and Molecular Immunology Approaches for the Development of Immunotherapies against the New Coronavirus (SARS-CoV-2): Challenges to Near-Future Breakthroughs | 2020 | Wrong outcome |
| Mendoza, Maria A and Raja, Mohammed and Villavicencio, Aasith and Anjan, Shweta and Natori, Yoichiro | Is the outcome of SARS-CoV-2 infection in solid organ transplant recipients really similar to that of the general population? | 2020 | Insufficient original data |
| Messika, Jonathan and Eloy, Philippine and Roux, Antoine and Hirschi, S and rine and Nieves, Ana and Le Pavec, JÃ©rÃ´me and SÃ©nÃ©chal, Agathe and Saint Raymond, Christel and Carlier, Nicolas and Demant, Xavier | COVID-19 in lung transplant recipients | 2021 | Insufficient original data |
| Meyerowitz, E. A. and Sen, P. and Schoenfeld, S. R. and Neilan, T. G. and Frigault, M. J. and Stone, J. H. and Kim, A. Y. and Mansour, M. K. | Immunomodulation as Treatment for Severe Coronavirus Disease 2019: A Systematic Review of Current Modalities and Future Directions | 2021 | Wrong population |
| Midha, I. K. and Kumar, N. and Kumar, A. and Madan, T. | Mega doses of retinol: A possible immunomodulation in Covid-19 illness in resource-limited settings | 2021 | Wrong population |
| Mihalopoulos, M. and Levine, A. C. and Marayati, N. F. and Chubak, B. M. and Archer, M. and Badani, K. K. and Tewari, A. K. and Mohamed, N. and Ferrer, F. and Kyprianou, N. | The Resilient Child: Sex-Steroid Hormones and COVID-19 Incidence in Pediatric Patients | 2020 | Wrong population |
| Mikulska, M. and Nicolini, L. A. and Signori, A. and Di Biagio, A. and Sepulcri, C. and Russo, C. and Dettori, S. and Berruti, M. and Sormani, M. P. and Giacobbe, D. R. and Vena, A. and De Maria, A. and Dentone, C. and Taramasso, L. and Mirabella, M. and Magnasco, L. and Mora, S. and Delfino, E. and Toscanini, F. and Balletto, E. and Aless and rini, A. I. and Baldi, F. and Briano, F. and Camera, M. and Dodi, F. and Ferrazin, A. and Labate, L. and Mazzarello, G. and Pincino, R. and Portunato, F. and Tutino, S. and Barisione, E. and Bruzzone, B. and Orsi, A. and Schenone, E. and Rosseti, N. and Sasso, E. and Da Rin, G. and Pelosi, P. and Beltramini, S. and Giacomini, M. and Icardi, G. and Gratarola, A. and Bassetti, M. | Tocilizumab and steroid treatment in patients with COVID-19 pneumonia | 2020 | Wrong population |
| Milic, J. and Novella, A. and Meschiari, M. and Menozzi, M. and Santoro, A. and Bedini, A. and Cuomo, G. and Franceschini, E. and Digaetano, M. and Carli, F. and Ciusa, G. and Volpi, S. and Bacca, E. and Franceschi, G. and Yaacoub, D. and Rogati, C. and Tutone, M. and Burastero, G. and Faltoni, M. and Iadisernia, V. and Dolci, G. and Cossarizza, A. and Mussini, C. and Pasina, L. and Guaraldi, G. | Darunavir/Cobicistat Is Associated with Negative Outcomes in HIV-Negative Patients with Severe COVID-19 Pneumonia | 2021 | Wrong outcome |
| Minkove, S. J. and Geiger, G. and Llibre, J. M. and Montgomery, M. W. and West, N. E. and Chida, N. M. and Antar, A. A. R. and D and achi, D. and Weld, E. D. | Clinical outcomes after IL-6 blockade in patients with COVID-19 and HIV: a case series | 2022 | n<50 |
| Mir and a, M. N. S. and Pingarilho, M. and Pimentel, V. and Torneri, A. and Seabra, S. G. and Libin, P. J. K. and Abecasis, A. B. | A Tale of Three Recent Pandemics: Influenza, HIV and SARS-CoV-2 | 2022 | Insufficient original data |
| Mirouse, A. and Darmon, M. and Zafrani, L. and Lengline, E. and Azoulay, E. | Impact of immunosuppression on mortality in critically ill COVID-19 patients | 2020 | Single centre |
| Mitchell, K. M. and Dimitrov, D. and Silhol, R. and Geidelberg, L. and Moore, M. and Liu, A. and Beyrer, C. and Mayer, K. H. and Baral, S. and Boily, M. C. | Estimating the potential impact of COVID-19-related disruptions on HIV incidence and mortality among men who have sex with men in the United States: a modelling study | 2020 | Wrong outcome |
| Mitchell, K. M. and Dimitrov, D. and Silhol, R. and Geidelberg, L. and Moore, M. and Liu, A. and Beyrer, C. and Mayer, K. H. and Baral, S. and Boily, M. C. | The potential effect of COVID-19-related disruptions on HIV incidence and HIV-related mortality among men who have sex with men in the USA: a modelling study | 2021 | Wrong outcome |
| Mohamed Khosroshahi, L. and Rokni, M. and Mokhtari, T. and Noorbakhsh, F. | Immunology, immunopathogenesis and immunotherapeutics of COVID-19; an overview | 2021 | Insufficient original data |
| Mohan, A. and Shaikh, M. T. A. and Wara, U. U. and Rackimuthu, S. and Costa, Acds and Lal, P. M. and Ahmad, S. and Essar, M. Y. | HIV/AIDS among children in Ratodero, Pakistan amidst the COVID-19 pandemic: Challenges, efforts, and recommendations | 2021 | Wrong population |
| Mohan, S. and King, K. and Husain, S. A. and Schold, J. | COVID-19-Associated Mortality among Kidney Transplant Recipients and Candidates in the United States | 2021 | Insufficient original data |
| Mohan, Sumit and King, Kristen and Husain, S Ali and Schold, Jesse | COVID-19Ã¢â‚¬â€œAssociated Mortality among Kidney Transplant Recipients and Candidates in the | 2021 | Duplicate |
| Mohanty, R. R. and Biswa Mohan, Padhy and Meher, B. R. | Effectiveness of pulse dose methyl prednisolone in management of COVID 19: A systematic review and meta-analysis of observational studies | 2022 | Wrong population |
| Mohanty, R. R. and Das, S. and Padhy, B. M. and Meher, B. R. | Comparison of Clinical Outcome between Dexamethasone and Methyl Prednisolone in Treatment of Moderate to Severe COVID-19: A Systematic Review and Meta-Analysis | 2022 | Wrong population |
| Mohr-Holl and , E. and Daniels, J. and Douglas-Jones, B. and Mema, N. and Scott, V. and Trivino-Duran, L. and Pfaff, C. and Furin, J. and Isaakidis, P. | A positive COVID-19 test is associated with high mortality in RR-TB-HIV patients | 2021 | Compound risk group |
| Monteiro, M. A. and Prates, G. S. and AP, R. Veiga and Magri, M. M. C. and Gascon, M. R. P. and Ferreira, M. D. and Tiberto, L. and Pereira, L. O. and de Lima Nascimento, N. A. and Polis, T. J. B. and Alves, W. and Fonseca, L. A. M. and Duarte, A. J. S. and Casseb, J. | SARS-CoV-2/COVID-19: Clinical course among subjects HIV-1-infected in Sao Paulo | 2022 | n<50 |
| Monteleone, G. and Ardizzone, S. | Are Patients with Inflammatory Bowel Disease at Increased Risk for Covid-19 Infection? | 2020 | Incorrect study design |
| Montero, Fern and o and MartÃ­nez-Barrio, Julia and Serrano-Benavente, BelÃ©n and GonzÃ¡lez, Teresa and Rivera, Javier and Molina Collada, Juan and CastrejÃ³n, Isabel and Ãlvaro-Gracia, Jose | Coronavirus disease 2019 (COVID-19) in autoimmune and inflammatory conditions: clinical characteristics of poor outcomes | 2020 | Single centre |
| Moosazadeh, M. and Mousavi, T. | Combination therapy of tocilizumab and steroid for COVID-19 patients: A meta-analysis | 2022 | Wrong population |
| Morani, Z. and Patel, S. and Ghosh, S. and Hassan, F. A. and Doreswamy, S. and Singh, S. and Kothapudi, V. N. and Desai, R. | COVID-19 in HIV: a Review of Published Case Reports | 2020 | n<50 |
| Moreno, A. and Vargas, C. and Azocar, F. and Villarroel, F. and CofrÃ©, M. and Oppliger, H. and RÃ­os, F. and Raijmakers, M. and Silva-Ayarza, I. and BeltrÃ¡n, C. and Zamora, F. | Steroids and mortality in non-critically ill COVID-19 patients: a propensity score-weighted study in a Chilean cohort | 2021 | Immunosuppression as treatment |
| Mugisa, B. and Sabry, A. and Hutin, Y. and Hermez, J. | HIV epidemiology in the WHO Eastern Mediterranean region: a multicountry programme review | 2022 | Incorrect study design |
| Mukherjee, A. P. | Hypothesis: Immunotherapy by Selective Convalescent Blood Engineering to Stifle Diseases like COVID-19 | 2021 | Wrong population |
| Munch, M. W. and Granholm, A. and Kjaer, M. N. and Aksnes, T. S. and SÃ¸lling, C. G. and Christensen, S. and Perner, A. | Long-term mortality and health-related quality of life in the COVID STEROID trial | 2022 | Immunosuppression as treatment |
| Munch, M. W. and Granholm, A. and Myatra, S. N. and Vijayaraghavan, B. and Cronhjort, M. and Wahlin, R. R. and Jakob, S. M. and Cioccari, L. and Kjaer, M. N. and Vesterlund, G. K. and Meyhoff, T. S. and Helleberg, M. and MÃ¸ller, M. H. and Benfield, T. and Venkatesh, B. and Hammond, N. and Micallef, S. and Bassi, A. and John, O. and Jha, V. and Kristiansen, K. T. and Ulrik, C. S. and JÃ¸rgensen, V. L. and Smitt, M. and Bestle, M. H. and Andreasen, A. S. and Poulsen, L. M. and Rasmussen, B. S. and BrÃ¸chner, A. C. and StrÃ¸m, T. and MÃ¸ller, A. and Khan, M. S. and Padmanaban, A. and Divatia, J. V. and Saseedharan, S. and Borawake, K. and Kapadia, F. and Dixit, S. and Chawla, R. and Shukla, U. and Amin, P. and Chew, M. S. and Gluud, C. and Lange, T. and Perner, A. | Higher vs lower doses of dexamethasone in patients with COVID-19 and severe hypoxia (COVID STEROID 2) trial: Protocol and statistical analysis plan | 2021 | Wrong population |
| Munch, M. W. and Meyhoff, T. S. and Helleberg, M. and Kjaer, M. N. and Granholm, A. and HjortsÃ¸, C. J. S. and Jensen, T. S. and MÃ¸ller, M. H. and Hjortrup, P. B. and Wetterslev, M. and Vesterlund, G. K. and Russell, L. and JÃ¸rgensen, V. L. and Kristiansen, K. T. and Benfield, T. and Ulrik, C. S. and Andreasen, A. S. and Bestle, M. H. and Poulsen, L. M. and Hildebr and t, T. and Knudsen, L. S. and MÃ¸ller, A. and SÃ¸lling, C. G. and BrÃ¸chner, A. C. and Rasmussen, B. S. and Nielsen, H. and Christensen, S. and StrÃ¸m, T. and Cronhjort, M. and Wahlin, R. R. and Jakob, S. M. and Cioccari, L. and Venkatesh, B. and Hammond, N. and Jha, V. and Myatra, S. N. and Jensen, M. Q. and Leistner, J. W. and Mikkelsen, V. S. and Svenningsen, J. S. and Laursen, S. B. and Hatley, E. V. and Kristensen, C. M. and Al-Alak, A. and Clapp, E. and Jonassen, T. B. and Bjerregaard, C. L. and Ã˜sterby, N. C. H. and Jespersen, M. M. and Abou-Kassem, D. and Lassen, M. L. and Zaabalawi, R. and Daoud, M. M. and Abdi, S. and Meier, N. and la Cour, K. and Derby, C. B. and Damlund, B. R. and Laigaard, J. and Andersen, L. L. and Mikkelsen, J. and Jensen, J. L. S. and Rasmussen, A. H. and ArnerlÃ¶v, E. and Lykke, M. and Holst-Hansen, M. Z. B. and TÃ¸stesen, B. W. and Schwab, J. and Madsen, E. K. and Gluud, C. and Lange, T. and Perner, A. | Low-dose hydrocortisone in patients with COVID-19 and severe hypoxia: The COVID STEROID randomised, placebo-controlled trial | 2021 | Wrong population |
| Munch, M. W. and Myatra, S. N. and Vijayaraghavan, B. K. T. and Saseedharan, S. and Benfield, T. and Wahlin, R. R. and Rasmussen, B. S. and Andreasen, A. S. and Poulsen, L. M. and Cioccari, L. and Khan, M. S. and Kapadia, F. and Divatia, J. V. and BrÃ¸chner, A. C. and Bestle, M. H. and Helleberg, M. and Michelsen, J. and Padmanaban, A. and Bose, N. and MÃ¸ller, A. and Borawake, K. and Kristiansen, K. T. and Shukla, U. and Chew, M. S. and Dixit, S. and Ulrik, C. S. and Amin, P. R. and Chawla, R. and Wamberg, C. A. and Shah, M. S. and Darfelt, I. S. and JÃ¸rgensen, V. L. and Smitt, M. and Granholm, A. and KjÃ¦r, M. N. and MÃ¸ller, M. H. and Meyhoff, T. S. and Vesterlund, G. K. and Hammond, N. E. and Micallef, S. and Bassi, A. and John, O. and Jha, A. and Cronhjort, M. and Jakob, S. M. and Gluud, C. and Lange, T. and Kadam, V. and Marcussen, K. V. and Hollenberg, J. and Hedman, A. and Nielsen, H. and SchjÃ¸rring, O. L. and Jensen, M. Q. and Leistner, J. W. and Jonassen, T. B. and Kristensen, C. M. and Clapp, E. C. and HjortsÃ¸, C. J. S. and Jensen, T. S. and Halstad, L. S. and Bak, E. R. B. and Zaabalawi, R. and Metcalf-Clausen, M. and Abdi, S. and Hatley, E. V. and Aksnes, T. S. and Gleipner-Andersen, E. and AlarcÃ³n, A. F. and Yamin, G. and Heymowski, A. and Berggren, A. and La Cour, K. and Weihe, S. and Pind, A. H. and EngstrÃ¸m, J. and Jha, V. and Venkatesh, B. and Perner, A. | Effect of 12 mg vs 6 mg of Dexamethasone on the Number of Days Alive Without Life Support in Adults With COVID-19 and Severe Hypoxemia: The COVID STEROID 2 Randomized Trial | 2021 | Wrong population |
| Murali, A. and Wong, P. and Gilbar, P. J. and Mangos, H. M. | Acquired Hemophilia A following Pfizer-BioNTech SARS CoV-2 mRNA vaccine, successfully treated with prednisolone and rituximab | 2022 | Wrong outcome |
| Murali, M. and Gowtham, H. G. and Ansari, M. A. and Alomary, M. N. and Alghamdi, S. and Almehmadi, M. and Singh, S. B. and Shilpa, N. and Aiyaz, M. and Kalegowda, N. and Ledesma, A. E. and Amruthesh, K. N. | Repositioning Therapeutics for SARS-CoV-2: Virtual Screening of Plant-based Anti-HIV Compounds as Possible Inhibitors against COVID-19 Viral RdRp | 2022 | Wrong population |
| Murphy, E. J. and Masterson, C. and Rezoagli, E. and O'Toole, D. and Major, I. and Stack, G. D. and Lynch, M. and Laffey, J. G. and Rowan, N. J. | Î²-Glucan extracts from the same edible shiitake mushroom Lentinus edodes produce differential in-vitro immunomodulatory and pulmonary cytoprotective effects - Implications for coronavirus disease (COVID-19) immunotherapies | 2020 | Wrong population |
| Murphy, L. | Systemic lupus erythematosus: overview, management and COVID-19 | 2022 | Insufficient original data |
| Murthy, S. K. and Kuenzig, M. E. and Windsor, J. W. and Ghia, J. E. and Griffiths, A. M. and Panaccione, R. and Seow, C. H. and Benchimol, E. I. and Bernstein, C. N. and Bitton, A. and Huang, J. G. and Jones, J. L. and Lee, K. and Kaplan, G. G. and Mukhtar, M. S. and T and on, P. and Targownik, L. E. and Gibson, D. L. | Crohn's and Colitis Canada's 2021 Impact of COVID-19 and Inflammatory Bowel Disease in Canada: COVID-19 Vaccines-Biology, Current Evidence and Recommendations | 2021 | Incorrect study design |
| Myers, Catherine N and Scott, John Harwood and Criner, Gerard J and Cordova, Francis C and Mamary, Albert James and Marchetti, Nathaniel and Shenoy, Kartik V and Galli, Jonathan A and Mulhall, Patrick D and Brown, James C | COVID-19 in lung transplant recipients | 2020 | Insufficient original data |
| Myers, L. C. and Murray, R. K. and Donato, B. M. K. and Liu, V. X. and Kipnis, P. and Kipnis, P. and Shaikh, A. and Franchino-Elder, J. | Persistent Steroid Exposure Before Coronavirus Disease 2019 Diagnosis and Risk of Hospitalization in Patients With Chronic Obstructive Pulmonary Disease | 2022 | Wrong population |
| Nachega, J. B. and Kapata, N. and Sam-Agudu, N. A. and Decloedt, E. H. and Katoto, Pdmc and Nagu, T. and Mwaba, P. and Yeboah-Manu, D. and Ch and a-Kapata, P. and Ntoumi, F. and Geng, E. H. and Zumla, A. | Minimizing the impact of the triple burden of COVID-19, tuberculosis and HIV on health services in sub-Saharan Africa | 2021 | Compound risk group |
| Nacif, Lucas Souto and Zanini, Leonardo Y and Waisberg, Daniel R and Pinheiro, Rafael S and GalvÃ£o, FlÃ¡vio and Andraus, Wellington and D'Albuquerque, Luiz Carneiro | COVID-19 in solid organ transplantation patients: A systematic review | 2020 | n<50 |
| Nagai, H. and Saito, M. and Adachi, E. and Sakai-Tagawa, Y. and Yamayoshi, S. and Kiso, M. and Kawamata, T. and Koga, M. and Kawaoka, Y. and Tsutsumi, T. and Yotsuyanagi, H. | Casirivimab/Imdevimab for Active COVID-19 Pneumonia Which Persisted for Nine Months in a Patient with Follicular Lymphoma during Anti-CD20 Therapy | 2022 | n<50 |
| Nagarakanti, S. R. and Okoh, A. K. and Grinberg, S. and Bishburg, E. | Clinical outcomes of patients with COVID-19 and HIV coinfection | 2021 | n<50 |
| Naidoo, N. and Moodley, J. and Naicker, T. | Maternal endothelial dysfunction in HIV-associated preeclampsia comorbid with COVID-19: a review | 2021 | Wrong population |
| Nair, K. and Mallick, S. and Veerankutty, F. and Yadav, A. and Srinivas Reddy, M. and Jamir, I. and Chaudhary, A. and Wadhawan, M. and Saraf, N. and Singh Soin, A. and Surendran, S. | COVID-19 in liver transplant recipients portend high mortality: A multicentric Indian experience | 2022 | Insufficient original data |
| Nair, V. and J and ovitz, N. and Abate, M. and Nair, G. D. and Bhaskaran, M. C. and Molmenti, E. P. | Risk factors for mortality in kidney transplant recipients with COVID-19 | 2020 | n<50 |
| Nair, Vinay and J and ovitz, Nicholas and Jhaveri, Kenar D and Molmenti, Ernesto | COVID-19 and solid organ transplant outcomes | 2020 | Insufficient original data |
| Nalubega, S. and Kyenkya, J. and Bagaya, I. and Nabukenya, S. and Ssewankambo, N. and Nakanjako, D. and Kiragga, A. N. | COVID-19 may exacerbate the clinical, structural and psychological barriers to retention in care among women living with HIV in rural and peri-urban settings in Uganda | 2021 | Wrong outcome |
| Narain, S. and Stefanov, D. G. and Chau, A. S. and Weber, A. G. and Marder, G. and Kaplan, B. and Malhotra, P. and Bloom, O. and Liu, A. and Lesser, M. L. and Hajizadeh, N. | Comparative Survival Analysis of Immunomodulatory Therapy for Coronavirus Disease 2019 Cytokine Storm | 2021 | Wrong population |
| Nargesi, S. and Bongomin, F. and Hedayati, M. T. | The impact of COVID-19 pandemic on AIDS-related mycoses and fungal neglected tropical diseases: Why should we worry? | 2021 | Wrong population |
| Naser Moghadasi, A. and Shabany, M. and Heidari, H. and Esk and arieh, S. | Can pulse steroid therapy increase the risk of infection by COVID-19 in patients with multiple sclerosis? | 2021 | Wrong population |
| Nasir, N. and Tajuddin, S. and Khaskheli, S. and Khan, N. and Niamatullah, H. and Nasir, N. | Clinical outcomes of immunomodulatory therapies in the management of COVID-19: A tertiary-care experience from Pakistan | 2022 | Wrong population |
| Navari, Y. and Bagheri, A. B. and Akhavan Rezayat, A. and SeyedAlinaghi, S. and Najafi, S. and Barzegary, A. and Asadollahi-Amin, A. | Mortality risk factors in kidney-transplanted patients with COVID-19: A systematic review and regression analysis | 2021 | Duplicate |
| Navari, Y. and Bagheri, A. B. and Akhavan Rezayat, A. and SeyedAlinaghi, S. and Najafi, S. and Barzegary, A. and Asadollahi-Amin, A. | Mortality risk factors in kidney-transplanted patients with COVID-19: A systematic review and regression analysis | 2021 | Wrong population |
| Neyens, T. | Did an effect of kidney transplantation on COVID-19 mortality go unnoticed due to selection bias? | 2021 | Insufficient original data |
| Ngamprasertchai, T. and Kajeekul, R. and Sivakorn, C. and Ruenroegnboon, N. and Luvira, V. and Siripoon, T. and Luangasanatip, N. | Efficacy and Safety of Immunomodulators in Patients with COVID-19: A Systematic Review and Network Meta-Analysis of Randomized Controlled Trials | 2022 | Wrong population |
| Ngouanom Kuate, M. P. and Bongomin, F. and Ndip, R. N. | SARS-CoV-2, HIV, and Mycobacterium tuberculosis triple co-infection | 2022 | Wrong population |
| Nguyen, M. T. and Sterris, J. and Woloszyn, J. and Regmi, S. and Rattanavich, R. and Rakoski, M. and Lin, M. and Mehta, P. and Kore, A. and Weissman, J. and Phan, T. and Jerez-Aguilar, B. and Robinson, M. and Wells, E. and Villicana, R. and De Vera, M. | Neutrophil-to-Lymphocyte Ratio at Time of COVID-19 Diagnosis Predicts Severity of Illness and Mortality in Solid Abdominal Organ Transplant Recipients | 2022 | Single centre |
| Nguyen, T. V. and Frost, S. A. | Effect of Steroids on Coronavirus Disease 2019 (COVID-19) Mortality Risk: A Bayesian Interpretation | 2021 | Wrong population |
| Nielsen, Travis B and Pantapalangkoor, Paul and Luna, Brian and Dekitani, Ken and Bruhn, Kevin and Tan, Br and on and Junus, Justin and Bonomo, Robert A and Schmidt, Ann Marie and Doherty, Terrence M | A Change in Paradigm: Diabetes Exacerbates Infection Severity by Hyperinflammation, Not Immunosuppression | 2016 | Incorrect date |
| Nierengarten, Mary Beth | Poor overall survival in bone marrow transplant patients with COVID-19 | 2021 | Incorrect study design |
| Nln, I. and Fern and ez-Ruiz, R. and Muskardin, T. L. W. and Paredes, J. L. and Blazer, A. D. and Tuminello, S. and Attur, M. and Iturrate, E. and Petrilli, C. M. and Abramson, S. B. and Chakravarti, A. and Niewold, T. B. | Interferon pathway lupus risk alleles modulate risk of death from acute COVID-19 | 2021 | Wrong outcome |
| Nomah, D. K. and Reyes-UrueÃ±a, J. and DÃ­az, Y. and Moreno, S. and Aceiton, J. and Bruguera, A. and Vivanco-Hidalgo, R. M. and Llibre, J. M. and Domingo, P. and FalcÃ³, V. and Imaz, A. and CortÃ©s, C. and Force, L. and Letang, E. and VilarÃ³, I. and Casabona, J. and Miro, J. M. | Sociodemographic, clinical, and immunological factors associated with SARS-CoV-2 diagnosis and severe COVID-19 outcomes in people living with HIV: a retrospective cohort study | 2021 | Wrong outcome |
| Obata, R. and Maeda, T. and Rizk, D. and Kuno, T. | Increased Secondary Infection in COVID-19 Patients Treated with Steroids in New York City | 2021 | Incorrect study design |
| O'Brien, E. R. and S and hu, J. K. | Sex differences in COVID-19 mortality: opportunity to develop HSP27 (HSPB1) immunotherapy to treat hyper-inflammation? | 2020 | Wrong population |
| ObriÈ™cÄƒ, B. and Vornicu, A. and JurubiÈ›Äƒ, R. and Mocanu, V. and Dimofte, G. and Andronesi, A. and Sorohan, B. and Achim, C. and Micu, G. and BobeicÄƒ, R. and Dina, C. and Ismail, G. | Characteristics of SARS-CoV-2 Infection in an Actively Monitored Cohort of Patients with Lupus Nephritis | 2022 | n<50 |
| Ochoa-Ramirez, L. A. and Ramos-Payan, R. and Jimenez-Gastelum, G. R. and Rodriguez-Millan, J. and Aguilar-Medina, M. and Rios-Tostado, J. J. and Ayala-Ham, A. and Bermudez, M. and Osuna-Ramos, J. F. and Olimon-Andalon, V. and Velarde-Felix, J. S. | The Chemokine MIG is Associated with an Increased Risk of COVID-19 Mortality in Mexican Patients | 2022 | Wrong population |
| Ofori-Asenso, Richard and Ogundipe, Oyepeju and Agyeman, Akosua Adom and Chin, Ken Lee and Mazidi, Mohsen and Ademi, Zanfina and De Bruin, Marie Louise and Liew, Danny | Cancer is associated with severe disease in COVID-19 patients: a systematic review and meta-analysis | 2020 | Wrong outcome |
| Oh, S. M. and Ham, S. Y. and Suh, H. J. and Lee, E. and Park, S. W. | Clinical Characteristics of COVID-19: Use of Steroids in Mostly Unvaccinated COVID-19 Patients Before the Omicron Variant | 2022 | Wrong outcome |
| Okumura, K. and Nishida, S. and Dh and , A. | Trends in COVID-19 Mortality among Solid Organ Transplant Recipients: Implications for Prevention | 2022 | Wrong outcome |
| Olivera, M. J. | Dexamethasone and COVID-19: Strategies in Low- and Middle-Income Countries to Tackle Steroid-Related Strongyloides Hyperinfection | 2021 | Wrong population |
| Omran, H. M. and Almaliki, M. S. | Influence of NAD+ as an ageing-related immunomodulator on COVID 19 infection: A hypothesis | 2020 | Wrong population |
| Ortiz-MartÃ­nez, Y. and LÃ³pez-LÃ³pez, MÃ and Ruiz-GonzÃ¡lez, C. E. and Turbay-Caballero, V. and Sacoto, D. H. and Caldera-Caballero, M. and Bravo, H. and Sarmiento, J. and Rodriguez-Morales, A. J. | Willingness to receive COVID-19 vaccination in people living with HIV/AIDS from Latin America | 2022 | Wrong outcome |
| Osmanodja, B. and Mayrdorfer, M. and Halleck, F. and Choi, M. and Budde, K. | Undoubtedly, kidney transplant recipients have a higher mortality due to COVID-19 disease compared to the general population | 2021 | Incorrect study design |
| Ouyang, J. and Bajracharya, S. D. and Yap, E. and Mallappallil, M. C. | Mortality of AKI in human immunodeficiency virus with and without co-infection with COVID-19 | 2020 | Compound risk group |
| Owaidah, T. and Saleh, M. and Aguilos, A. M. and Amri, A. A. and Maghrabi, K. and Owaidah, M. and Siddiqui, K. and Alsaleh, K. and Alnounou, R. | Incidence of lupus anticoagulant in hospitalized covid-19 patients | 2021 | Wrong population |
| PÃ©rez-Ortega, JoaquÃ­n and Almanza-Ortega, Nelva Nely and Torres-Poveda, Kirvis and MartÃ­nez-GonzÃ¡lez, Gerardo and Zavala-DÃ­az, JosÃ© CrispÃ­n and Pazos-Rangel, Rodolfo | Application of Data Science for Cluster Analysis of COVID-19 Mortality According to Sociodemographic Factors at Municipal Level in Mexico | 2022 | Wrong population |
| Pahalyants, V. and Murphy, W. S. and Klebanov, N. and Lu, C. and Theodosakis, N. and Klevens, R. M. and Estir, H. and Lilly, E. and Asgari, M. and Semenov, Y. R. | Immunosuppressive biologics did not increase the risk of COVID-19 or subsequent mortality: A retrospective matched cohort study from Massachusetts | 2022 | Wrong outcome |
| Parekh, R. and Zhang, X. and Ungaro, R. C. and Brenner, E. J. and Agrawal, M. and Colombel, J. F. and Kappelman, M. D. | Presence of Comorbidities Associated with Severe Coronavirus Infection in Patients with Inflammatory Bowel Disease | 2022 | Compound risk group |
| Park, D. E. and Higdon, M. M. and Prosperi, C. and Baggett, H. C. and Brooks, W. A. and Feikin, D. R. and Hammitt, L. L. and Howie, S. R. C. and Kotloff, K. L. and Levine, O. S. and Madhi, S. A. and Murdoch, D. R. and O'Brien, K. L. and Scott, J. A. G. and Thea, D. M. and Antonio, M. and Awori, J. O. and Baillie, V. L. and Bunthi, C. and Kwenda, G. and Mackenzie, G. A. and Moore, D. P. and Morpeth, S. C. and Mwanany and a, L. and Paveenkittiporn, W. and Ziaur Rahman, M. and Rahman, M. and Rhodes, J. and Sow, S. O. and Tapia, M. D. and Deloria Knoll, M. | Upper Respiratory Tract Co-detection of Human Endemic Coronaviruses and High-density Pneumococcus Associated With Increased Severity Among HIV-Uninfected Children Under 5 Years Old in the PERCH Study | 2021 | Wrong population |
| Park, J. and Lee, S. H. and You, S. C. and Kim, J. and Yang, K. | Non-steroidal anti-inflammatory agent use may not be associated with mortality of coronavirus disease 19 | 2021 | Wrong population |
| Park, J. N. and Owczarzak, J. and Urquhart, G. and Morris, M. and Weicker, N. P. and Rouhani, S. and Sherman, S. G. | HIV Risk Among Urban and Suburban People Who Inject Drugs: Elevated Risk Among Fentanyl and Cocaine Injectors in Maryland | 2022 | Wrong population |
| Park, S. H. and Kim, H. J. and Lee, C. K. and Song, E. M. and Kang, S. B. and Jang, B. I. and Kim, E. S. and Kim, K. O. and Lee, Y. J. and Kim, E. Y. and Jung, Y. J. and Park, S. K. and Park, D. I. and Ye, B. D. and Jung, S. A. and Yang, S. K. | Safety and Optimal Timing of BCG Vaccination in Infants Born to Mothers Receiving Anti-TNF Therapy for Inflammatory Bowel Disease | 2020 | Wrong population |
| Parker, A. and Koegelenberg, C. F. N. and Moolla, M. S. and Louw, E. H. and Mowlana, A. and NortjÃ©, A. and Ahmed, R. and Brittain, N. and Lalla, U. and Allwood, B. W. and Prozesky, H. and Schrueder, N. and Taljaard, J. J. | High HIV prevalence in an early cohort of hospital admissions with COVID-19 in Cape Town, South Africa | 2020 | Single centre |
| Pascale, M. M. and Frongillo, F. and Bianco, G. and Gali and ro, F. and Nure, E. and Agnes, S. and Giovinazzo, F. | COVID-19 Syndrome After Liver Transplant: Immunosuppression Role and Risk Factors for Mortality | 2022 | Incorrect study design |
| Pascual, J. and Melilli, E. and Jimenez-Martin, C. and Gonzalez-Monte, E. and Zarraga, S. and Gutierrez-Dalmau, A. and Lopez-Jimenez, V. and Juega, J. and Munoz-Cepeda, M. and Lorenzo, I. and Facundo, C. and Ruiz-Fuentes, M. D. C. and Mazuecos, A. and Sanchez-Alvarez, E. and Crespo, M. and Spanish Society of Nephrology, Covid-Group | COVID-19-related Mortality During the First 60 Days After Kidney Transplantation | 2020 | n<50 |
| Pastor, D. M. and Lee-Wisdom, K. and Arai, A. E. and Sirajuddin, A. and Rosing, D. R. and Korchin, B. and Gulley, J. L. and Bilusic, M. | Fast Clearance of the SARS-CoV-2 Virus in a Patient Undergoing Vaccine Immunotherapy for Metastatic Chordoma: A Case Report | 2020 | n<50 |
| Patel, V. V. and Felsen, U. R. and Fisher, M. and Fazzari, M. J. and Ginsberg, M. S. and Beil, R. and Akiyama, M. J. and Anastos, K. and Hanna, D. B. | Clinical Outcomes and Inflammatory Markers by HIV Serostatus and Viral Suppression in a Large Cohort of Patients Hospitalized With COVID-19 | 2021 | Single centre |
| Peach, E. and Rutter, M. and Lanyon, P. and Grainge, M. J. and Hubbard, R. and Aston, J. and Bythell, M. and Stevens, S. and Pearce, F. | Risk of death among people with rare autoimmune diseases compared with the general population in England during the 2020 COVID-19 pandemic | 2021 | Wrong outcome |
| Pereira, Marcus R and Arcasoy, Selim and Farr, Maryjane A and Mohan, Sumit and Emond, Jean C and Tsapepas, Demetra S and Shi, Qiuhu and Purpura, Lawrence and Uhlemann, Anneâ€Catrin and Zucker, Jason | Outcomes of COVID-19 in solid organ transplant recipients: a matched cohort study | 2021 | Single centre |
| Permpalung, Nitipong and Bazemore, Katrina and Chiang, Teresa Po-Yu and Mathew, Joby and Barker, Lindsay and Nematollahi, Saman and Cochran, Willa and Sait, Afrah S and Avery, Robin K and Shah, Pali D | Impact of COVID-19 on lung allograft and clinical outcomes in lung transplant recipients: a case-control study | 2021 | n<50 |
| Perrotta, Cristina M | Evaluation of HIV Status as a Risk Factor for COVID-19 Infection: A Combined Cohort Study | 2021 | Incorrect study design |
| Peters, L. L. and Raymer, D. S. and Pal, J. D. and Ambardekar, A. V. | Association of COVID-19 Vaccination With Risk of COVID-19 Infection, Hospitalization, and Death in Heart Transplant Recipients | 2022 | Single centre |
| Petersen, M. W. and Meyhoff, T. S. and Helleberg, M. and Kjaer, M. N. and Granholm, A. and HjortsÃ¸, C. J. S. and Jensen, T. S. and MÃ¸ller, M. H. and Hjortrup, P. B. and Wetterslev, M. and Vesterlund, G. K. and Russell, L. and JÃ¸rgensen, V. L. and Tjelle, K. and Benfield, T. and Ulrik, C. S. and Andreasen, A. S. and Mohr, T. and Bestle, M. H. and Poulsen, L. M. and Hitz, M. F. and Hildebr and t, T. and Knudsen, L. S. and MÃ¸ller, A. and SÃ¸lling, C. G. and BrÃ¸chner, A. C. and Rasmussen, B. S. and Nielsen, H. and Christensen, S. and StrÃ¸m, T. and Cronhjort, M. and Wahlin, R. R. and Jakob, S. and Cioccari, L. and Venkatesh, B. and Hammond, N. and Jha, V. and Myatra, S. N. and Gluud, C. and Lange, T. and Perner, A. | Low-dose hydrocortisone in patients with COVID-19 and severe hypoxia (COVID STEROID) trial-Protocol and statistical analysis plan | 2020 | Wrong population |
| PiÃ±ana, JosÃ© Luis and Martino, Rodrigo and GarcÃ­a-GarcÃ­a, Irene and Parody, RocÃ­o and Morales, MarÃ­a Dolores and Benzo, Gonzalo and GÃ³mez-Catalan, Irene and Coll, Rosa and De La Fuente, Ignacio and Luna, Alej and ro | Risk factors and outcome of COVID-19 in patients with hematological malignancies | 2020 | Paediatric data |
| Pinna, G. | Sex and COVID-19: A Protective Role for Reproductive Steroids | 2021 | Wrong population |
| Pistor, M. and Hoepner, A. G. F. and Lin, Y. and Jung, S. and Bassetti, C. L. and Chan, A. and Salmen, A. and Hoepner, R. | Immunotherapies and COVID-19 mortality: a multidisciplinary open data analysis based on FDA's Adverse Event Reporting System | 2021 | Wrong population |
| Pistor, M. and Hoepner, R. and Hoepner, A. G. F. and Lin, Y. and Jung, S. and Bassetti, C. L. and Chan, A. and Salmen, A. | Multiple Sclerosis immunotherapies and COVID-19 mortality: an analysis of the FDA Adverse Event Reporting System | 2022 | Wrong population |
| Plummer, M. M. and Pavia, C. S. | COVID-19 Vaccines for HIV-Infected Patients | 2021 | Incorrect study design |
| Ponsford, M. J. and Ward, T. J. C. and Stoneham, S. M. and Dallimore, C. M. and Sham, D. and Osman, K. and Barry, S. M. and Jolles, S. and Humphreys, I. R. and Farewell, D. | A Systematic Review and Meta-Analysis of Inpatient Mortality Associated With Nosocomial and Community COVID-19 Exposes the Vulnerability of Immunosuppressed Adults | 2021 | Wrong outcome |
| Pouladzadeh, M. and Safdarian, M. and Eshghi, P. and Abolghasemi, H. and Bavani, A. G. and Sheibani, B. and Moradi Choghakabodi, P. and Feghhi, A. and Ghafourian Boroujerdnia, M. and Forouzan, A. and Jalali Far, M. A. and Kaydani, G. A. and Rajaei, E. and Amin, M. and Torabizadeh, M. and Yousefi, F. and Hadaddezfuli, R. | A randomized clinical trial evaluating the immunomodulatory effect of convalescent plasma on COVID-19-related cytokine storm | 2021 | Wrong outcome |
| Prabhu, S. and Poongulali, S. and Kumarasamy, N. | Impact of COVID-19 on people living with HIV: A review | 2020 | Incorrect study design |
| Prashantha, C. N. and Gouthami, K. and Lavanya, L. and Bhavanam, S. and Jakhar, A. and Shakthiraju, R. G. and Suraj, V. and Sahana, K. V. and Sujana, H. S. and Guruprasad, N. M. and Ramach and ra, R. | Molecular screening of antimalarial, antiviral, anti-inflammatory and HIV protease inhibitors against spike glycoprotein of coronavirus | 2021 | Wrong outcome |
| Prower, E. and Hadfield, S. and Saha, R. and Woo, T. and Ang, K. M. and Metaxa, V. | A critical care outreach team under strain - Evaluation of the service provided to patients with haematological malignancy during the Covid-19 pandemic | 2022 | Insufficient original data |
| Pujari, S. and Gaikwad, S. and Chitalikar, A. and Dabhade, D. and Joshi, K. and Bele, V. | Short Communication: Coronavirus Disease 19 Among People Living with HIV in Western India: An Observational Cohort Study | 2021 | Single centre |
| Qasim, A. and Mansour, M. and Kousa, O. and Awad, D. and Abuhazeem, B. and Millner, P. and Velagapudi, M. | A case of coronavirus disease 2019 in acquired immunodeficiency syndrome patient: a case report and review of the literature | 2020 | Insufficient original data |
| Queiroz, N. S. F. and Teixeira, F. V. and Motta, M. P. and Chebli, L. A. and Hino, A. A. F. and Martins, C. A. and Quaresma, A. B. and da Silva, A. A. P. and DamiÃ£o, Aomc and Saad-Hossne, R. and Kotze, P. G. | Risk stratification and geographical mapping of Brazilian inflammatory bowel disease patients during the COVID-19 outbreak: Results from a nationwide survey | 2021 | Wrong outcome |
| QuirÃ³s-GonzÃ¡lez, V. and Rubio, R. and Pulido, F. and Rial-Crestelo, D. and MartÃ­n-Jurado, C. and HernÃ¡ndez-Ros, MÃ and JimÃ©nez, E. A. L. and Miguel Ferrari, J. and Manuel Caro-Teller, J. and Pinar, Ã“ and Pedrera-JimÃ©nez, M. and GarcÃ­a-Barrio, N. and Serrano, P. and Luis Bernal, J. | Healthcare Outcomes In Patients With Hiv Infection At A Tertiary Hospital During The COVID-19 Pandemic | 2021 | Single centre |
| Quitadamo, P. A. and Comegna, L. and Cristalli, P. | Anti-Infective, Anti-Inflammatory, and Immunomodulatory Properties of Breast Milk Factors for the Protection of Infants in the Pandemic From COVID-19 | 2020 | Wrong population |
| Rabaan, A. A. and Al-Ahmed, S. H. and Muhammad, J. and Khan, A. and Sule, A. A. and Tirupathi, R. and Mutair, A. A. and Alhumaid, S. and Al-Omari, A. and Dhawan, M. and Tiwari, R. and Sharun, K. and Mohapatra, R. K. and Mitra, S. and Bilal, M. and Alyami, S. A. and Emran, T. B. and Moni, M. A. and Dhama, K. | Role of Inflammatory Cytokines in COVID-19 Patients: A Review on Molecular Mechanisms, Immune Functions, Immunopathology and Immunomodulatory Drugs to Counter Cytokine Storm | 2021 | Wrong population |
| Ramos, L. and Carrillo-Palau, M. and Alonso-Abreu, I. and Reygosa, C. and HernÃ¡ndez-Alvarez, N. and Amaral, C. and HernÃ¡ndez, A. and BenÃ­tez-Zafra, F. and PÃ©rez-GonzÃ¡lez, F. and Quintana-DÃ­az, H. and Hern and ez-Guerra, M. | COVID-19 vaccination rate and willingness of an additional dose among inflammatory bowel disease patients receiving biologic therapy: Fearless and with desire | 2022 | Wrong outcome |
| RÃºa-Figueroa, I. and RÃºa-Figueroa, D. and PÃ©rez-Veiga, N. and Anzola, A. M. and Galindo-Izquierdo, M. and Calvo-AlÃ©n, J. and FernÃ¡ndez-Nebro, A. and SangÃ¼esa, C. and Menor-Almagro, R. and Tomero, E. and Del Val, N. and Uriarte-Isazelaya, E. and Blanco, R. and Andreu, J. L. and Boteanu, A. and NarvÃ¡ez, J. and Cobo, T. and BohÃ³rquez, C. and Montilla, C. and Salas, E. and Toyos, F. J. and Bernal, J. A. and Salgado, E. and Freire, M. and Mas, A. J. and ExpÃ³sito, L. and HernÃ¡ndez-Beriain, J. A. and Ibarguengoitia, O. and Velloso-Feijoo, M. L. and Lozano-Rivas, N. and Bonilla, G. and Moreno, M. and JimÃ©nez, I. and Quevedo-Vila, V. and PecondÃ³n, A. and Aurrecoechea, E. and Valls, E. and MouriÃ±o, C. and VÃ¡zquez-RodrÃ­guez, T. and Pego-Reigosa, J. M. | Antimalarials exert a cardioprotective effect in lupus patients: Insights from the Spanish Society of Rheumatology Lupus Register (RELESSER) analysis of factors associated with heart failure | 2022 | Wrong outcome |
| Ravanan, R. and Mumford, L. and Ushiro-Lumb, I. and Callaghan, C. and Pettigrew, G. and Thorburn, D. and Gardiner, D. and Forsythe, J. and Otdt Clinical, Team | Two Doses of SARS-CoV-2 Vaccines Reduce Risk of Death Due to COVID-19 in Solid Organ Transplant Recipients: Preliminary Outcomes From a UK Registry Linkage Analysis | 2021 | Wrong outcome |
| Razia, D. and Olson, M. T. and Walia, R. and Bremner, R. M. and Smith, M. A. and Tokman, S. | A Comparison of Short-Term Morbidity and Mortality Among Inpatient Lung Transplant Recipients Transplanted for COVID-19 and Other Restrictive Lung Diseases | 2022 | n<50 |
| Razia, D. and Omar, A. and Grief, K. and Walia, R. and Tokman, S. | COVID-19 Has a High Mortality Rate in Lung Transplant Recipients: A Large Single-Center Experience | 2022 | Single centre |
| Reece, M. D. and Taylor, R. R. and Song, C. and Gavegnano, C. | Targeting Macrophage Dysregulation for Viral Infections: Novel Targets for Immunomodulators | 2021 | Wrong outcome |
| Rentsch, C. T. and DeVito, N. J. and MacKenna, B. and Morton, C. E. and Bhaskaran, K. and Brown, J. P. and Schultze, A. and Hulme, W. J. and Croker, R. and Walker, A. J. and Williamson, E. J. and Bates, C. and Bacon, S. and Mehrkar, A. and Curtis, H. J. and Evans, D. and Wing, K. and Inglesby, P. and Mathur, R. and Drysdale, H. and Wong, A. Y. S. and McDonald, H. I. and Cockburn, J. and Forbes, H. and Parry, J. and Hester, F. and Harper, S. and Smeeth, L. and Douglas, I. J. and Dixon, W. G. and Evans, S. J. W. and Tomlinson, L. and Goldacre, B. | Effect of pre-exposure use of hydroxychloroquine on COVID-19 mortality: a population-based cohort study in patients with rheumatoid arthritis or systemic lupus erythematosus using the OpenSAFELY platform | 2021 | Immunosuppression as treatment |
| Revelo-Garcia, R. and Danguilan, R. and De leon, D. F. | Pos-655 Risk Factors for in-Hospital Mortality in Chronic Peritoneal Dialysis Patients Admitted with Covid-19 at the National Kidney and Transplant Institute of the Philippines | 2021 | Wrong population |
| Rezende, R. P. V. and Mendonca de Santana, F. and Figueiredo, C. P. | Unchanged trend in mortality from systemic lupus erythematosus during the 2020 COVID-19 pandemic: A nationwide population-based study | 2022 | Wrong outcome |
| Ricciuto, A. and Lamb, C. A. and Benchimol, E. I. and Walker, G. J. and Kennedy, N. A. and Kuenzig, M. E. and Kaplan, G. G. and Kappelman, M. D. and Ungaro, R. C. and Colombel, J. F. and Brenner, E. J. and Agrawal, M. and Reinisch, W. and Griffiths, A. M. and Sebastian, S. | Inflammatory Bowel Disease Clinical Activity is Associated with COVID-19 Severity Especially in Younger Patients | 2022 | Wrong outcome |
| Riches, John Charles | Impact of COVID-19 in patients with lymphoid malignancies | 2021 | Incorrect study design |
| Rinaldi, Matteo and Bartoletti, Michele and Bussini, Linda and Pancaldi, Livia and Pascale, Renato and Comai, Giorgia and Morelli, Mariacristina and Ravaioli, Matteo and Cescon, Matteo and Cristini, Francesco | COVID-19 in solid organ transplant recipients: No difference in survival compared to general population | 2021 | n<50 |
| Ringer, M. and Azmy, V. and Kaman, K. and Tang, D. and Cheung, H. and Azar, M. M. and Price, C. and Malinis, M. | A retrospective matched cohort single-center study evaluating outcomes of COVID-19 and the impact of immunomodulation on COVID-19-related cytokine release syndrome in solid organ transplant recipients | 2021 | Single centre |
| Rivera, J. C. H. H. and Mendoza, M. S. and Covarrubias, L. G. and Ramirez, A. L. Q. and Reyes, L. C. and Hern and ez, M. B. and Martinez, J. R. and Contla, Y. P. and Duran, J. M. V. and Rivas, A. T. and Flores, S. L. R. and Murillo, W. A. Q. and Sierra, J. R. P. | Mortality Due to COVID-19 in Renal Transplant Recipients, Related to Variants of SARS-CoV-2 and Vaccination in Mexico | 2022 | Wrong outcome |
| Rizk, J. G. and Kalantar-Zadeh, K. and Mehra, M. R. and Lavie, C. J. and Rizk, Y. and Forthal, D. N. | Pharmaco-Immunomodulatory Therapy in COVID-19 | 2020 | Wrong population |
| Rodriguez-Cubillo, B. and Moreno de la Higuera, M. A. and Perez-Flores, I. and Calvo Romero, N. and Aiffil, A. S. and Arribi Vilela, A. and Peix, B. and Huertas, S. and Juez, A. and Sanchez-Fructuoso, A. I. | Clinical Effectiveness of SARS-CoV-2 Vaccination in Renal Transplant Recipients. Antibody Levels Impact in Pneumonia and Death | 2022 | Duplicate |
| Rodriguez-Cubillo, B. and Moreno de la Higuera, M. A. and Perez-Flores, I. and Calvo Romero, N. and Aiffil, A. S. and Arribi Vilela, A. and Peix, B. and Huertas, S. and Juez, A. and Sanchez-Fructuoso, A. I. | Clinical Effectiveness of SARS-CoV-2 Vaccination in Renal Transplant Recipients. Antibody Levels Impact in Pneumonia and Death | 2022 | Duplicate |
| Rodriguez-Cubillo, B. and Moreno De La Higuera, M. A. and Perez-Flores, I. and Calvo Romero, N. and Aiffil, A. S. and Arribi Vilela, A. and Peix, B. and Huertas, S. and Juez, A. and Sanchez-Fructuoso, A. I. | Clinical Effectiveness of SARS-CoV-2 Vaccination in Renal Transplant Recipients. Antibody Levels Impact in Pneumonia and Death | 2022 | Single centre |
| Rodríguez-Cubillo, B., de la Higuera, M.A.M., Pérez-Flores, I., Romero, N.C., Aiffil, A.S., Vilela, A.A., Peix, B., Huertas, S., Juez, A. and Sanchez-Fructuoso, A.I | Clinical Effectiveness of SARS-CoV-2 Vaccination in Renal Transplant Recipients. Antibody Levels Impact in Pneumonia and Death: Erratum | 2022 | Wrong population |
| Rodriguez-Peralvarez, M. and Colmenero, J. and Salcedo, M. and Spanish Society of Liver, Transplantation | Reply to: "Age and comorbidity are central to the risk of death from COVID-19 in liver transplant recipients" | 2021 | Incorrect study design |
| Roldan, M. K. | POS-493 Predictors of Mortality in Chronic Hemodialysis Patients with COVID-19 at National Kidney and Transplant Institute: A Retrospective Cohort Study | 2021 | Single centre |
| Romero-HernÃ¡ndez, B. and MartÃ­nez-GarcÃ­a, L. and RodrÃ­guez-Dominguez, M. and MartÃ­nez-Sanz, J. and VÃ©lez-DÃ­az-PallarÃ©s, M. and PÃ©rez Mies, B. and Muriel, A. and Gea, F. and PÃ©rez-ElÃ­as, M. J. and GalÃ¡n, J. C. | The Negative Impact of COVID-19 in HCV, HIV, and HPV Surveillance Programs During the Different Pandemic Waves | 2022 | Wrong outcome |
| Rommasi, F. and Nasiri, M. J. and Mirsaeidi, M. | Immunomodulatory agents for COVID-19 treatment: possible mechanism of action and immunopathology features | 2022 | Wrong population |
| Roncati, L. and Corsi, L. and Barbolini, G. | Abnormal immunothrombosis and lupus anticoagulant in a catastrophic COVID-19 recalling Asherson's syndrome | 2021 | n<50 |
| Routray, P. and Samal, S. and Mishra, D. | Long term morbidity and mortality in covid patients discharged from hospital with or without steroid as discharge medication | 2021 | Wrong population |
| Roy, K. and Himelfarb, A. and Karrah, K. and Porterfield, L. and Paremoer, L. and Serag, H. and Lee, W. C. | The Social, Behavioral, and Ethical Modalities of COVID-19 on HIV Care in South Africa: A Systematic Review | 2022 | Wrong outcome |
| Ruiz-AntorÃ¡n, B. and Sancho-LÃ³pez, A. and Torres, F. and FernÃ¡ndez-Cruz, A. | A Response to: Letter to the Editor Regarding Combination of Tocilizumab and Steroids to Improve Mortality in Patients with Severe COVID-19 Infection: A Spanish, Multicenter, Cohort | 2021 | Incorrect study design |
| Ruiz-AntorÃ¡n, B. and Sancho-LÃ³pez, A. and Torres, F. and Moreno-Torres, V. and de Pablo-LÃ³pez, I. and GarcÃ­a-LÃ³pez, P. and Abad-Santos, F. and Rosso-FernÃ¡ndez, C. M. and Aldea-Perona, A. and MontanÃ©, E. and Aparicio-HernÃ¡ndez, R. M. and Llop-Rius, R. and PedrÃ³s, C. and GijÃ³n, P. and HernÃ¡ndez-Carballo, C. and Pedrosa-MartÃ­nez, M. J. and RodrÃ­guez-JimÃ©nez, C. and Prada-Ramallal, G. and Cabrera-GarcÃ­a, L. and Aguilar-GarcÃ­a, J. A. and Sanjuan-Jimenez, R. and Ortiz-Barraza, E. I. and SÃ¡nchez-Chica, E. and FernÃ¡ndez-Cruz, A. | Combination of Tocilizumab and Steroids to Improve Mortality in Patients with Severe COVID-19 Infection: A Spanish, Multicenter, Cohort Study | 2021 | Wrong population |
| Ruiz-Irastorza, G. and MartÃ­n-Iglesias, D. and Soto-Peleteiro, A. | Update on antimalarials and systemic lupus erythematosus | 2020 | Wrong outcome |
| Ruiz-Irastorza, G. and Pijoan, J. I. and Bereciartua, E. and Dunder, S. and Dominguez, J. and Garcia-Escudero, P. and Rodrigo, A. and Gomez-Carballo, C. and Varona, J. and Guio, L. and Ibarrola, M. and Ugarte, A. and Martinez-Berriotxoa, A. | Second week methyl-prednisolone pulses improve prognosis in patients with severe coronavirus disease 2019 pneumonia: An observational comparative study using routine care data | 2020 | Wrong outcome |
| Ruscitti, P. and Berardicurti, O. and Di Benedetto, P. and Cipriani, P. and Iagnocco, A. and Shoenfeld, Y. and Giacomelli, R. | Severe COVID-19, Another Piece in the Puzzle of the Hyperferritinemic Syndrome. An Immunomodulatory Perspective to Alleviate the Storm | 2020 | Wrong outcome |
| Ryan, S. and Campbell, P. and Paskins, Z. and Hider, S. and Manning, F. and Rule, K. and Brooks, M. and Hassell, A. | Exploring the physical, psychological and social well-being of people with rheumatoid arthritis during the coronavirus pandemic: a single-centre, longitudinal, qualitative interview study in the UK | 2022 | Wrong population |
| Ryu, J. K. and Sozmen, E. G. and Dixit, K. and Montano, M. and Matsui, Y. and Liu, Y. and Helmy, E. and Deerinck, T. J. and Yan, Z. and Schuck, R. and Acevedo, R. M. and Spencer, C. M. and Thomas, R. and Pico, A. R. and Zamvil, S. S. and Lynch, K. L. and Ellisman, M. H. and Greene, W. C. and Akassoglou, K. | SARS-CoV-2 spike protein induces abnormal inflammatory blood clots neutralized by fibrin immunotherapy | 2021 | Wrong population |
| Rzeniewicz, K. and Larkin, J. and Menzies, A. M. and Turajlic, S. | Immunotherapy use outside clinical trial populations: never say never? | 2021 | Wrong population |
| S, F. A. and Madhu, M. and Udaya Kumar, V. and Dhingra, S. and Kumar, N. and Singh, S. and Ravich and iran, V. and Murti, K. | Nutritional Aspects of People Living with HIV (PLHIV) Amidst COVID-19 Pandemic: an Insight | 2022 | Wrong outcome |
| Saezâ€GimÃ©nez, Berta and Berastegui, Cristina and Barrecheguren, Miriam and Revillaâ€LÃ³pez, Eva and Los Arcos, Ibai and Alonso, Rodrigo and Aguilar, Myriam and Mora, VÃ­ctor M and Otero, Isabel and Reig, Juan P | COVID-19 in lung transplant recipients: a multicenter study | 2021 | Insufficient original data |
| Sagris, D. and Florentin, M. and Tasoudis, P. and Korompoki, E. and Gatselis, N. and Giamarellos-Bourboulis, E. J. and Milionis, H. and Douketis, J. and Spyropoulos, A. C. and Dalekos, G. and Ntaios, G. | Immunomodulation and Reduction of Thromboembolic Risk in Hospitalized COVID-19 Patients: Systematic Review and Meta-Analysis of Randomized Trials | 2021 | Wrong population |
| Sahilu, T. and Sheleme, T. and Melaku, T. | Severity and Mortality Associated with Steroid Use among Patients with COVID-19: A Systematic Review and Meta-Analysis | 2021 | Wrong population |
| Sahoo, A. and Swain, S. S. and Paital, B. and P and a, M. | Combinatorial approach of vitamin C derivative and anti-HIV drug-darunavir against SARS-CoV-2 | 2022 | Wrong population |
| Sahu, A. K. and Mathew, R. and Bhat, R. and Malhotra, C. and Nayer, J. and Aggarwal, P. and Galwankar, S. | Steroids use in non-oxygen requiring COVID-19 patients: a systematic review and meta-analysis | 2021 | Wrong population |
| Sahu, Kamal Kant and Siddiqui, Ahmad Daniyal | A review on recipients of hematopoietic stem cell transplantation patients with COVID-19 infection | 2021 | Incorrect study design |
| Sakthiswary, R. and Chuah, H. Y. and Chiang, K. S. and Liew, Y. S. and Muhammad Aizat, N. A. | COVID-19 in systemic lupus erythematosus: A pooled analysis and systematic review of case reports and series | 2021 | n<50 |
| Sanchezâ€Pina, JosÃ© MarÃ­a and RodrÃ­guez Rodriguez, Mario and Castro Quismondo, Nerea and Gil Manso, Rodrigo and Colmenares, Rafael and Gil Alos, Daniel and Paciello, Mari Liz and Zafra, Denis and Garciaâ€Sanchez, Cristina and Villegas, Carolina | Clinical course and risk factors for mortality from COVID-19 in patients with haematological malignancies | 2020 | n<50 |
| Santeusanio, A. D. and Menon, M. C. and Liu, C. and Bhansali, A. and Patel, N. and Mahir, F. and Rana, M. and Tedla, F. and Mahamid, A. and Fenig, Y. and Zendel, A. and Delaney, V. and De Boccardo, G. and Farouk, S. S. and Sehgal, V. and Khaim, R. and Jacobs, S. E. and Dunn, D. and Sullivan, T. and Taimur, S. and Baneman, E. and Florman, S. and Shapiro, R. | Influence of patient characteristics and immunosuppressant management on mortality in kidney transplant recipients hospitalized with coronavirus disease 2019 (COVID-19) | 2021 | n<50 |
| Saponara, M. and Pala, L. and Conforti, F. and Rubatto, M. and De Risi, I. and Spagnolo, F. and Guida, M. and Bossi, P. and Quaglino, P. and Queirolo, P. | Patients with locally advanced and metastatic cutaneous squamous cell carcinoma treated with immunotherapy in the era of COVID-19: stop or go? Data from five Italian referral cancer centers | 2020 | Insufficient original data |
| Sari, A. and Aslan, M. and Ekinci, O. | The effect of steroids used in the treatment of coronavirus disease 2019 on infections in intensive care | 2022 | Wrong population |
| Sarkar, I. and Sen, A. | In silico screening predicts common cold drug Dextromethorphan along with Prednisolone and Dexamethasone can be effective against novel Coronavirus disease (COVID-19) | 2022 | Wrong population |
| Sarkar, S. and Khanna, P. and Soni, K. D. | Are the steroids a blanket solution for COVID-19? A systematic review and meta-analysis | 2021 | Wrong population |
| Sarma, P. and Bhattacharyya, A. and Kaur, H. and Prajapat, M. and Prakash, A. and Kumar, S. and Bansal, S. and Kirubakaran, R. and Reddy, D. H. and Muktesh, G. and Kaushal, K. and Sharma, S. and Shekhar, N. and Avti, P. and Thota, P. and Medhi, B. | Efficacy and safety of steroid therapy in COVID-19: A rapid systematic review and Meta-analysis | 2020 | Wrong population |
| Sattui, S. E. and Crow, M. K. and Navarro-MillÃ¡n, I. | The role of immunomodulatory medications in the treatment of COVID-19 | 2021 | Wrong population |
| Sawalha, A. H. and Zhao, M. and Coit, P. and Lu, Q. | Epigenetic dysregulation of ACE2 and interferon-regulated genes might suggest increased COVID-19 susceptibility and severity in lupus patients | 2020 | Wrong population |
| Saxena, H. M. | Immunotherapy of COVID-19 with Bacille Calmette - Guerin: Where is the missing red herring? | 2021 | Wrong population |
| Sbikina, E. and Ivanov, A. and Khaimenova, T. and Vinnitskaya, E. and Solovyeva, O. and Akhmedianov, A. and Alikhanov, R. | Impact of COVID-19 to mortality in waiting list for liver transplantation. Single center study | 2022 | Wrong population |
| Schaenman, J. and Byford, H. and Grogan, T. R. and Motwani, Y. and Beaird, O. and Kamath, M. and Lum, E. and Meneses, K. and Sayah, D. and Vucicevic, D. and Saab, S. | Risk Factors Associated with ICU Admission and Death in Patients Hospitalized with COVID-19 Differ Between Solid Organ Transplant and Non-Solid Organ Transplant Patients | 2022 | Single centre |
| Schmalzle, S. A. and Viviano, N. A. and Mohanty, K. and Palmeiro, R. M. and Hoffmann, J. D. and Sheth-P and it, N. and Gruber-Baldini, A. and Stafford, K. A. | People aging with HIV - protecting a population vulnerable to effects of COVID-19 and its control measures | 2021 | Wrong population |
| Schmidt-Lauber, C. and Gunster, C. and Huber, T. B. and Spoden, M. and Grahammer, F. | Collateral Effects and Mortality of Kidney Transplant Recipients during the COVID-19 Pandemic | 2021 | Wrong outcome |
| Schold, J. D. and King, K. L. and Husain, S. A. and Poggio, E. D. and Buccini, L. D. and Mohan, S. | COVID-19 mortality among kidney transplant candidates is strongly associated with social determinants of health | 2021 | Wrong population |
| Scolari, Fern and o Luis and Hastenteufel, Laura Carolina Tavares and Einsfeld, LÃ­dia and Bueno, Julia and Orl and in, LetÃ­cia and Clausell, Nadine and Goldraich, Livia A | Impact of COVID-19 infection among heart transplant recipients: a Southern Brazilian experience | 2022 | n<50 |
| Seijo, L. L. and Perez, A. and Shah, R. J. and Kolaitis, N. A. and Venado, A. and Leard, L. E. and Kleinhenz, M. and Singer, J. P. and Golden, J. A. and Kukreja, J. and Trinh, B. and Hays, S. | Respiratory disease, and treatment / thematic poster session mortality rate in lung transplant recipients infected with COVID-19 | 2021 | Single centre |
| Sekar, N. and Sundaresan, K. T. | The Co-infection of Mild COVID-19 and Rhinocerebral Mucormycosis in a Patient Without Diabetes or Prior Steroid Use | 2022 | n<50 |
| Sellier, P. and Alex and re-Castor, G. and Bouchaud, O. and Diamantis, S. and Hamet, G. and Brun, A. and Chabrol, A. and Machado, M. and Bouldouyre, M. A. and Ponscarme, D. and De Castro, N. and Leroy, P. and Turpault, I. and Rozenbaum, W. and Molina, J. M. | Causes of death among HIV-infected patients during the COVID-19 pandemic: A multicenter prospective study in the Paris Region | 2021 | Wrong outcome |
| Sen, A. | Repurposing prolactin as a promising immunomodulator for the treatment of COVID-19: Are common Antiemetics the wonder drug to fight coronavirus? | 2020 | Wrong population |
| Sendic, S. and Mansouri, L. and Havervall, S. and Thalin, C. and Lundahl, J. and Jacobson, S. H. | Impact of monocyte chemoattractants on inhospital mortality in relation to kidney function in patients with COVID-19 | 2021 | Wrong population |
| Sengar, Manju and Chinnaswamy, Girish and Ranganathan, Priya and Ashok, Apurva and Bhosale, Shilpushp and Biswas, Sanjay and Chaturvedi, Pankaj and Dhamne, Chetan and Divatia, Jigeeshu and Dsa, Karishma | Outcomes of COVID-19 and risk factors in patients with cancer-a cohort study | 2021 | Single centre |
| SeyedAlinaghi, S. A. and Karimi, A. and Barzegary, A. and Mojdeganlou, H. and Vahedi, F. and Mirghaderi, S. P. and Shobeiri, P. and Ramezani, M. and Yousefi Konjdar, P. and Mirzapour, P. and Tantuoyir, M. M. and Mehraeen, E. and Dadras, O. and Voltarelli, F. | COVID-19 mortality in patients with immunodeficiency and its predictors: a systematic review | 2022 | Incorrect study design |
| Shadmanfar, S. and Jonaidi-Jafari, N. and Jafari, R. and Rastgar-Moqaddam, Z. and Saburi, A. | COVID-19 in rheumatoid arthritis cases: an Iranian referral center experience | 2021 | Insufficient original data |
| Shafiekhani, M. and Shahabinezhad, F. and Niknam, T. and Tara, S. A. and Haem, E. and Mardani, P. and Zare, Z. and Jafarian, S. and Mirzad Jahromi, K. and Arabsheybani, S. and Moeini, Y. S. and Alavi, J. and Jalali, S. S. and Salimi, M. and Shahriarirad, R. and Malekhosseini, S. A. | Evaluation of the therapeutic regimen in COVID-19 in transplant patients: where do immunomodulatory and antivirals stand? | 2021 | Single centre |
| Shah, H. and Busquets, A. C. | Psoriasis Flares in Patients With COVID-19 Infection or Vaccination: A Case Series | 2022 | n<50 |
| Shahzad, Moazzam and Chaudhary, Sibgha Gull and Zafar, Muhammad U and Hassan, Maha A and Hussain, Ali and Ali, Fatima and Anwar, Iqra and Ahmed, Mamoon and Ahmed, Nausheen and Khurana, Sharad | Impact of COVID-19 in hematopoietic stem cell transplant recipients: A systematic review and metaâ€analysis | 2022 | Insufficient original data |
| Shareef, M. A. and Bashaiwth, H. M. and AlAkbari, A. O. and Bahamran, M. S. and AlAmodi, M. O. and Albaiti, S. H. and Ali, M. A. and Eshaq, A. M. and Alkattan, K. and Alamodi, A. A. | A systematic review of contemporary evidence on SARS-CoV-2 and HIV coinfection: What does it look like up to date? | 2020 | n<50 |
| Sharma, A. and Choudhary, D. and Kenwar, D. and Singh, S. and Ramch and ran, R. and Kumar Sp, S. and Chauhan, P. | Transplant related risk factors analysis of mortality in renal transplant recipients with covid-19-a single center-based retrospective study | 2021 | Single centre |
| Sharma, N. K. and P and e, R. and Bag, P. | Prolonged COVID RT-PCR and Higher Mortality in COVID-19 Patients with Rituximab Treated Non-Hodgkin-Lymphoma: A Case Series | 2022 | n<50 |
| Sharma, Pratima and Chen, Vincent and Fung, Christopher M and Troost, Jonathan P and Patel, Vaiibhav N and Combs, Michael and Norman, Silas and Garg, Puneet and Colvin, Monica and Aaronson, Keith | COVID-19 outcomes among solid organ transplant recipients: a case-control study | 2021 | n<50 |
| Sharmeen, Saika and Elghawy, Ahmed and Zarlasht, Fnu and Yao, Qingping | COVID-19 in rheumatic disease patients on immunosuppressive agents | 2020 | n<50 |
| Shaw, B. and Shortt, J. and Low, M. and Rogers, B. and Kaplan, Z. and Fedele, P. and Gregory, G. and Vilcassim, S. and Gilbertson, M. and Grigoriadis, G. and Opat, S. | Low mortality in vaccinated immunocompromised haematology patients infected with SARS-CoV-2 | 2022 | Duplicate |
| Shaya, S. and Choudhuri, S. | COVID-19 Mortality in concurrent multiple myeloma (mm) - A prospective and comparative case series through the first and second wave of the sars-cov-2 pandemic in a uk tertiary haematology centre | 2021 | Single centre |
| Shehab, M. and Alrashed, F. and Alfadhli, A. | COVID-19 Vaccine Booster Dose Willingness among Patients with Inflammatory Bowel Disease on Infliximab and Vedolizumab: A Cross-Sectional Study | 2022 | Wrong outcome |
| Shehab, M. and Zurba, Y. and Al Abdulsalam, A. and Alfadhli, A. and Elouali, S. | COVID-19 Vaccine Hesitancy among Patients with Inflammatory Bowel Disease Receiving Biologic Therapies in Kuwait: A Cross-Sectional Study | 2021 | Wrong outcome |
| Shen, Y. and Freeman, J. A. and Holl and , J. and Solterbeck, A. and Naidu, K. and Soosapilla, A. and Downe, P. and Tang, C. and Kerridge, I. and Wallman, L. and Van Bilsen, N. and Milogiannakis, V. and Akerman, A. and Martins Costa Gomes, G. and S and gren, K. and Cunningham, A. L. and Turville, S. and Mulligan, S. P. | COVID-19 vaccine failure in chronic lymphocytic leukaemia and monoclonal B-lymphocytosis; humoural and cellular immunity | 2022 | Wrong outcome |
| Sherer, R. and Lehmann, C. and Zhu, M. and Lio, J. | Cancer, transplantation, and other immunocompromising conditions were not significantly associated with severe COVID-19 or death in hospitalized COVID-19 patients in Chicago | 2020 | Wrong outcome |
| Shingare, Ashay and Bahadur, Madan M and Raina, Shailesh | COVID-19 in recent kidney transplant recipients | 2020 | Single centre |
| Shionoya, Y. and Taniguchi, T. and Kasai, H. and Sakuma, N. and Imai, S. and Shikano, K. and Takayanagi, S. and Yahaba, M. and Nakada, T. A. and Igari, H. and Sakao, S. and Suzuki, T. | Possibility of deterioration of respiratory status when steroids precede antiviral drugs in patients with COVID-19 pneumonia: A retrospective study | 2021 | Wrong population |
| Shoemaker, M. E. and Huynh, L. M. and Smith, C. M. and Mustad, V. A. and Duarte, M. O. and Cramer, J. T. | Immunomodulatory Effects of Vitamin D and Prevention of Respiratory Tract Infections and COVID-19 | 2022 | Wrong population |
| Siegmund-Schultze, N. | COVID-19 after heart transplant: Risk of severe progressions and mortality are probably significantly increased. [German] | 2020 | Foreign language |
| Siewe Fodjo, J. N. and Faria de Moura Villela, E. and Van Hees, S. and Vanholder, P. and Reyntiens, P. and Colebunders, R. | Follow-Up Survey of the Impact of COVID-19 on People Living with HIV during the Second Semester of the Pandemic | 2021 | Wrong outcome |
| Sigel, K. and Swartz, T. and Golden, E. and Paranjpe, I. and Somani, S. and Richter, F. and De Freitas, J. K. and Miotto, R. and Zhao, S. and Polak, P. and Mutetwa, T. and Factor, S. and Meh and ru, S. and Mullen, M. and Cossarini, F. and Bottinger, E. and Fayad, Z. and Merad, M. and Gnjatic, S. and Aberg, J. and Charney, A. and Nadkarni, G. and Glicksberg, B. S. | Coronavirus 2019 and People Living With Human Immunodeficiency Virus: Outcomes for Hospitalized Patients in New York City | 2020 | Duplicate |
| Silva, E. C. G. E. and Schmitt, A. C. B. and Godoy, C. G. D. and Gambeta, A. C. and Carvalho, C. R. F. D. and Fu, C. and Tanaka, C. and Junior, C. T. and Carvalho, C. R. R. D. and Pompeu, J. E. | Ambulation capacity, age, immunosuppression, and mechanical ventilation are risk factors of in-hospital death in severe COVID-19: a cohort study | 2022 | Single centre |
| Silva, M. and Blamey, R. and Ceballos, M. E. and Araya, X. and BastÃ­as, C. and Twele, L. and MuÃ±oz, R. and Sciaraffia, A. and PiÃ±era, C. | [SARS-CoV-2 infection in people living with HIV. Topics on the global panorama and in Chile] | 2022 | Foreign language |
| Simms, E. L. and Chung, H. and Oberding, L. and Muruve, D. A. and McDonald, B. and Bromley, A. and Pillai, D. R. and Chun, J. | Post-mortem molecular investigations of SARS-CoV-2 in an unexpected death of a recent kidney transplant recipient | 2021 | n<50 |
| Siserman, C. V. and Jeican, II and Gheban, D. and Anton, V. and Mironescu, D. and È˜uÈ™man, S. and VicÄƒ, M. L. and LazÄƒr, M. and AluaÈ™, M. and Toader, C. and Albu, S. | Fatal Form of COVID-19 in a Young Male Bodybuilder Anabolic Steroid Using: The First Autopsied Case | 2022 | n<50 |
| Slimani, Y. and Abbassi, R. and El Fatoiki, F. Z. and Barrou, L. and Chiheb, S. | Systemic lupus erythematosus and varicella-like rash following COVID-19 in a previously healthy patient | 2021 | n<50 |
| Sloan, M. and Gordon, C. and Lever, E. and Harwood, R. and Bosley, M. A. and Pilling, M. and Brimicombe, J. and Naughton, F. and Blane, M. and Walia, C. and D'Cruz, D. | COVID-19 and shielding: experiences of UK patients with lupus and related diseases | 2021 | Wrong outcome |
| Smith, M. K. and Chow, J. and Huang, R. and Omar, M. and Ebadi, M. and Wong, P. and Huard, G. and Yoshida, E. M. and Peretz, D. and Brahmania, M. and Montano-Loza, A. J. and Bhanji, R. | Covid-19 Infection in Liver Transplant Recipients: Clinical Features, Hospitalization, and Mortality from a Canadian Multicentre Cohort | 2022 | n<50 |
| Smith, M. L. and Sharma, S. and Singh, T. P. | Comments on the Discussion Forum: Oromucosal immunomodulation as clinical spectrum mitigating factor in SARS-CoV-2 infection | 2022 | Wrong outcome |
| Snow, T. A. C. and Longobardo, A. and Brealey, D. and Down, J. and Satta, G. and Singer, M. and Arulkumaran, N. | Beneficial ex vivo immunomodulatory and clinical effects of clarithromycin in COVID-19 | 2022 | Wrong outcome |
| So, J. M. and Umeh, C. and Noriega, S. and Stratton, E. and Aseri, M. and Gupta, R. C. | Use of Baricitinib in Combination With Remdesivir and Steroid in COVID-19 Treatment: A Multicenter Retrospective Study | 2021 | Wrong outcome |
| Song, N. and Wakimoto, H. and Rossignoli, F. and Bhere, D. and Ciccocioppo, R. and Chen, K. S. and Khalsa, J. K. and Mastrolia, I. and Samarelli, A. V. and Dominici, M. and Shah, K. | Mesenchymal stem cell immunomodulation: In pursuit of controlling COVID-19 related cytokine storm | 2021 | Wrong population |
| Soni, S. and Mebratu, Y. A. | B-cell lymphoma-2 family proteins-activated proteases as potential therapeutic targets for influenza A virus and severe acute respiratory syndrome coronavirus-2: Killing two birds with one stone? | 2022 | Wrong population |
| Spiera, E. and Ungaro, R. C. and Kornbluth, A. | Effectiveness and Safety of COVID-19 Vaccines in Patients With Inflammatory Bowel Disease | 2022 | Incorrect study design |
| Spihlman, A. P. and Gadi, N. and Wu, S. C. and Moulton, V. R. | COVID-19 and Systemic Lupus Erythematosus: Focus on Immune Response and Therapeutics | 2020 | Insufficient original data |
| Sponga, S. and Benedetti, G. and Ferrara, V. and Nalli, C. and C, D. I. Nora and Lechiancole, A. and De Manna, N. D. and Dralov, A. and Vendramin, I. and Livi, U. | Are heart transplant patients at higher risk for mortality following SARS-CoV-2 infection single centre experience | 2021 | Single centre |
| Spottiswoode, N. and Bloomstein, J. D. and Caldera, S. and Sessolo, A. and McCauley, K. and Byanyima, P. and Zawedde, J. and Kalantar, K. and Kaswabuli, S. and Rutishauser, R. L. and Lieng, M. K. and Davis, J. L. and Moore, J. and Jan, A. and Iwai, S. and Shenoy, M. and Sanyu, I. and DeRisi, J. L. and Lynch, S. V. and Worodria, W. and Huang, L. and Langelier, C. R. | Pneumonia surveillance with culture-independent metatranscriptomics in HIV-positive adults in Uganda: a cross-sectional study | 2022 | Wrong outcome |
| Squillace, N. and Ricci, E. and Colella, E. and Bonfanti, P. | HIV and SARS-CoV-2 Co-Infection: What are the Risks? | 2021 | n<50 |
| Stoeckle, K. and Johnston, C. D. and Jannat-Khah, D. P. and Williams, S. C. and Ellman, T. M. and Vogler, M. A. and Gulick, R. M. and Glesby, M. J. and Choi, J. J. | COVID-19 in Hospitalized Adults With HIV | 2020 | n<50 |
| Stover, J. and Glaubius, R. and Kassanjee, R. and Dugdale, C. M. | Updates to the Spectrum/AIM model for the UNAIDS 2020 HIV estimates | 2021 | Insufficient original data |
| Strykowski, R. K. and Poonawalla, M. and Tyker, A. and Bauer Ventura, I. and Lee, C. and Jablonski, R. and Vij, R. and Chung, J. and Strek, M. and Adegunsoye, A. | Mortality Risk From COVID-19 Among Unvaccinated Subjects With Autoimmune Phenotypes of Interstitial Lung Disease | 2022 | n<50 |
| Stutz, M. R. and Dylla, N. P. and Pearson, S. D. and Lecompte-Osorio, P. and Nayak, R. and Khalid, M. and Adler, E. and Boissiere, J. and Lin, H. and Leiter, W. and Little, J. and Rose, A. and Moran, D. and Mullowney, M. W. and Wolfe, K. S. and Lehmann, C. and Odenwald, M. and De La Cruz, M. and Giurcanu, M. and Pohlman, A. S. and Hall, J. B. and Chaubard, J. L. and Sundararajan, A. and Sidebottom, A. and Kress, J. P. and Pamer, E. G. and Patel, B. K. | Immunomodulatory fecal metabolites are associated with mortality in COVID-19 patients with respiratory failure | 2022 | Wrong population |
| Sultan, Almetwaly M and Mahmoud, Hossam K and Fathy, Gamaleldin Mohamed and Abdelfattah, Nabil M | The outcome of hematopoietic stem cell transplantation patients with COVID-19 infection | 2021 | n<50 |
| Sultan, K. and Mone, A. and Durbin, L. and Khuwaja, S. and Swaminath, A. | Review of inflammatory bowel disease and COVID-19 | 2020 | Incorrect study design |
| Sun, M. and Shankar, R. and Ko, M. and Chang, C. D. and Yeh, S. J. and Li, S. and Liu, K. and Zhou, G. and Xing, J. and VanVelsen, A. and VanVelsen, T. and Paithankar, S. and Feng, B. Y. and Young, K. and Strug, M. and Turco, L. and Wang, Z. and Schadt, E. and Chen, R. and Li, X. and Oskotsky, T. and Sirota, M. and Glicksberg, B. S. and Nadkarni, G. N. and Moeser, A. J. and Li, L. and Kim, S. and Zhou, J. and Chen, B. | Sex differences in viral entry protein expression, host responses to SARS-CoV-2, and in vitro responses to sex steroid hormone treatment in COVID-19 | 2020 | Incorrect study design |
| Sung, K. Y. and Chang, T. E. and Wang, Y. P. and Lin, C. C. and Chang, C. Y. and Hou, M. C. and Lu, C. L. | SARS-CoV-2 vaccination in patients with inflammatory bowel disease: A systemic review and meta-analysis | 2022 | Wrong outcome |
| Suwanwongse, K. and Shabarek, N. | Variation in mortality of HIV/SARS-CoV-2 coinfected patients in the Bronx, New York City | 2021 | n<50 |
| TÃ¶rÃ¼ner, M. and Kalkan Ä°, H. and AkyÃ¼z, F. and Tezel, A. and Ã‡elik, A. F. | Turkish IBD Organization's Position Statement on Inflammatory Bowel Disease Management Recommendations During COVID-19 Pandemic | 2021 | Wrong population |
| Tabesh, E. and Soheilipour, M. and Rezaeisadrabadi, M. and Zare-Farashb and i, E. and Mousavi-Roknabadi, R. S. | Comparison the effects and side effects of Covid-19 vaccination in patients with inflammatory bowel disease (IBD): a systematic scoping review | 2022 | Wrong outcome |
| Talamonti, M. and Galluzzo, M. and Chiricozzi, A. and Quaglino, P. and Fabbrocini, G. and Gisondi, P. and Marzano, A. V. and Potenza, C. and Conti, A. and Parodi, A. and Piaserico, S. and Bardazzi, F. and Argenziano, G. and Rongioletti, F. and Stingeni, L. and Micali, G. and Loconsole, F. and Rossi, M. T. and Bongiorno, M. R. and Feliciani, C. and Rubegni, P. and Amerio, P. and Fargnoli, M. C. and Pigatto, P. and Savoia, P. and NisticÃ², S. P. and Giustini, S. and Carugno, A. and Cannavo, S. P. and Rech, G. and Prignano, F. and Offidani, A. and Lombardo, M. and Zalaudek, I. and Bianchi, L. and Peris, K. and Balestri, R. and Bernardini, N. and Belloni Fortini, A. and Burl and o, M. and Caldarola, G. and Campione, E. and Cattaneo, A. and Dapavo, P. and Dastoli, S. and De Simone, C. and Di Nuzzo, S. and Diotallevi, F. and Fierro, M. T. and Franchi, C. and Esposito, M. and Foti, C. and Gambini, D. M. and Gambardella, A. and Girolomoni, G. and Giunta, A. and Guarneri, C. and Gualdi, G. and Hansel, K. and Megna, M. and Mugheddu, C. and Musumeci, M. L. and Patrizi, A. and Pellacani, G. and Richetta, A. G. and Rosi, E. and Sacchelli, L. and Tiberio, R. and Tilotta, G. and Trovato, E. and Venturini, M. and Vezzoni, R. | Characteristic of chronic plaque psoriasis patients treated with biologics in Italy during the COVID-19 Pandemic: Risk analysis from the PSO-BIO-COVID observational study | 2021 | n<50 |
| Tam, C. S. and Brown, J. R. and Kahl, B. S. and Ghia, P. and Giannopoulos, K. and Jurczak, W. and Å imkoviÄ, M. and Shadman, M. and Ã–sterborg, A. and Laurenti, L. and Walker, P. and Opat, S. and Chan, H. and Ciepluch, H. and Greil, R. and Tani, M. and TrnÄ›nÃ½, M. and Br and er, D. M. and Flinn, I. W. and Grosicki, S. and Verner, E. and Tedeschi, A. and Li, J. and Tian, T. and Zhou, L. and Marimpietri, C. and Paik, J. C. and Cohen, A. and Huang, J. and Robak, T. and Hillmen, P. | Zanubrutinib versus bendamustine and rituximab in untreated chronic lymphocytic leukaemia and small lymphocytic lymphoma (SEQUOIA): a randomised, controlled, phase 3 trial | 2022 | Wrong population |
| Tamuzi, J. L. and Ayele, B. T. and Shumba, C. S. and Adetokunboh, O. O. and Uwimana-Nicol, J. and Haile, Z. T. and Inugu, J. and Nyasulu, P. S. | Implications of COVID-19 in high burden countries for HIV/TB: A systematic review of evidence | 2020 | Compound risk group |
| Tan, E. H. and Sena, A. G. and Prats-Uribe, A. and You, S. C. and Ahmed, W. U. and Kostka, K. and Reich, C. and Duvall, S. L. and Lynch, K. E. and Matheny, M. E. and Duarte-Salles, T. and Bertolin, S. F. and Hripcsak, G. and Natarajan, K. and Falconer, T. and Spotnitz, M. and Ostropolets, A. and Blacketer, C. and Alshammari, T. M. and Alghoul, H. and Alser, O. and Lane, J. C. E. and Dawoud, D. M. and Shah, K. and Yang, Y. and Zhang, L. and Areia, C. and Golozar, A. and Relcade, M. and Casajust, P. and Jonnagaddala, J. and Subbian, V. and Vizcaya, D. and Lai, L. Y. and Nyberg, F. and Morales, D. R. and Posada, J. D. and Shah, N. H. and Gong, M. and Vivekanantham, A. and Abend, A. and Minty, E. P. and Suchard, M. and Rijnbeek, P. and Ryan, P. B. and Prieto-Alhambra, D. | Characteristics, outcomes, and mortality amongst 133,589 patients with prevalent autoimmune diseases diagnosed with, and 48,418 hospitalised for COVID-19: a multinational distributed network cohort analysis | 2020 | Paediatric data |
| Tan, Y. and Tang, F. | SARS-CoV-2-mediated immune system activation and potential application in immunotherapy | 2021 | Wrong outcome |
| Tanner, T. and Wahezi, D. M. | Hyperinflammation and the utility of immunomodulatory medications in children with COVID-19 | 2020 | Wrong population |
| Targownik, L. E. and Bernstein, C. N. and Lakatos, P. L. and Murthy, S. K. and Benchimol, E. I. and Bitton, A. and Huang, J. G. and Kuenzig, M. E. and Jones, J. L. and Kaplan, G. G. and Lee, K. and Mukhtar, M. S. and T and on, P. and Windsor, J. W. and Panaccione, R. | Crohn's and Colitis Canada's 2021 Impact of COVID-19 and Inflammatory Bowel Disease in Canada: Risk Factors and Medications | 2021 | Incorrect study design |
| Taylor, M. F. | Author reply: Mortality in patients with kidney transplantation and SARS-CoV-2 infection. [Spanish] | 2022 | Incorrect study design |
| Tesoriero, J. M. and Swain, C. E. and Pierce, J. L. and Zamboni, L. and Wu, M. and Holtgrave, D. R. and Gonzalez, C. J. and Udo, T. and Morne, J. E. and Hart-Malloy, R. and Rajulu, D. T. and Leung, S. J. and Rosenberg, E. S. | Elevated COVID-19 outcomes among persons living with diagnosed HIV infection in New York State: Results from a population-level match of HIV, COVID-19, and hospitalization databases | 2020 | Duplicate |
| Thaunat, O. and Legeai, C. and Anglicheau, D. and Couzi, L. and Blancho, G. and Hazzan, M. and Pastural, M. and Savoye, E. and Bayer, F. and Morelon, E. and Le Meur, Y. and Bastien, O. and Caillard, S. and French nationwide Registry of Solid Organ Transplant Recipients with, Covid | IMPact of the COVID-19 epidemic on the moRTAlity of kidney transplant recipients and candidates in a French Nationwide registry sTudy (IMPORTANT) | 2020 | Wrong outcome |
| Thomas, G. and Frederick, E. and Hausburg, M. and Goldberg, L. and Hoke, M. and Roshon, M. and Mains, C. and Bar-Or, D. | The novel immunomodulatory biologic LMWF5A for pharmacological attenuation of the "cytokine storm" in COVID-19 patients: a hypothesis | 2020 | Wrong population |
| Thurm, C. and Reinhold, A. and Borucki, K. and Kahlfuss, S. and Feist, E. and Schreiber, J. and Reinhold, D. and Schraven, B. | Homologous and Heterologous Anti-COVID-19 Vaccination Does Not Induce New-Onset Formation of Autoantibodies Typically Accompanying Lupus Erythematodes, Rheumatoid Arthritis, Celiac Disease and Antiphospholipid Syndrome | 2022 | Wrong outcome |
| Tien, A. and Bhatnagar, M. S. and Sahota, A. K. | Incidence and Mortality of Covid-19 in Post-Liver Transplant Patients in a Large Integrated Healthcare System | 2021 | n<50 |
| TiendrÃ©bÃ©ogo, Wendlassida JoÃ«lle StÃ©phanie and KaborÃ©, Fulgence and DiendÃ©rÃ©, Eric Arnaud and Ouedraogo, Dieu-DonnÃ© | Case series of chronic inflammatory rheumatic disease patients infected by coronavirus disease 2019 (COVID-19) | 2020 | Insufficient original data |
| Toombs, J. M. and Van den Abbeele, K. and Democratis, J. and Merricks, R. and M and al, A. K. J. and Missouris, C. G. | COVID-19 in three people living with HIV in the United Kingdom | 2021 | n<50 |
| Torreggiani, M. and Fessi, H. and Piccoli, G. B. | On the need to better protect hemodialysis patients: a comment on "IMPact of the COVID-19 epidemic on the moRTAlity of kidney transplant recipients and candidates in a French Nationwide registry sTudy (IMPORTANT)" | 2021 | Incorrect study design |
| Torres, A. and Cilloniz, C. | Steroid therapy and antiviral treatment in SARS-CoV-2 pneumonia: clinical contexts and indications | 2022 | Wrong population |
| Torres, A. and Motos, A. and CillÃ³niz, C. and Ceccato, A. and FernÃ¡ndez-Barat, L. and GabarrÃºs, A. and Bermejo-Martin, J. and Ferrer, R. and Riera, J. and PÃ©rez-Arnal, R. and GarcÃ­a-Gasulla, D. and PeÃ±uelas, O. and Lorente, JÃ and de Gonzalo-Calvo, D. and Almansa, R. and MenÃ©ndez, R. and Palomeque, A. and Villar, R. A. and AÃ±Ã³n, J. M. and Balan MariÃ±o, A. and BarberÃ , C. and BarberÃ¡n, J. and Bl and ino Ortiz, A. and Boado, M. V. and Bustamante-Munguira, E. and Caballero, J. and CantÃ³n-Bulnes, M. L. and Carbajales PÃ©rez, C. and Carbonell, N. and CatalÃ¡n-GonzÃ¡lez, M. and de Frutos, R. and Franco, N. and GalbÃ¡n, C. and Gumucio-Sanguino, V. D. and de la Torre, M. D. C. and DÃ­az, E. and Estella, Ã and Gallego, E. and GarcÃ­a Garmendia, J. L. and GÃ³mez, J. M. and Huerta, A. and GarcÃ­a, R. N. J. and Loza-VÃ¡zquez, A. and Marin-Corral, J. and Martin Delgado, M. C. and MartÃ­nez de la GÃ¡ndara, A. and MartÃ­nez Varela, I. and LÃ³pez Messa, J. and Albaiceta, G. M. and Nieto, M. and Novo, M. A. and PeÃ±asco, Y. and PÃ©rez-GarcÃ­a, F. and Pozo-Laderas, J. C. and Ricart, P. and Sagredo, V. and SÃ¡nchez-Miralles, A. and Sancho Chinesta, S. and Serra-Fortuny, M. and Socias, L. and SolÃ©-Violan, J. and Suarez-Sipmann, F. and Tamayo Lomas, L. and Trenado, J. and Ãšbeda, A. and Valdivia, L. J. and Vidal, P. and BarbÃ©, F. | Major candidate variables to guide personalised treatment with steroids in critically ill patients with COVID-19: CIBERESUCICOVID study | 2022 | Wrong population |
| Torun, S. and Karaman, I. | Acute Exacerbation of Rheumatoid Arthritis Misdiagnosed as COVID-19: A Case Report | 2022 | n<50 |
| Traish, A. M. | Sex steroids and COVID-19 mortality in women | 2021 | Insufficient original data |
| Tripathy, S. and Alvarez, N. and Jaiswal, S. and Williams, R. and Al-Khadimi, M. and Hackman, S. and Phillips, W. and Kaur, S. and Cervantez, S. and Kelly, W. and Taverna, J. | Hypermetabolic lymphadenopathy following the administration of COVID-19 vaccine and immunotherapy in a lung cancer patient: a case report | 2022 | n<50 |
| Truchetet, Marie-Elise and Drumez, Elodie and Barnetche, Thomas and Martin, Claire and Devaux, Mathilde and Goulenok, Tiphaine and Maria, Alex and re and Schmidt, Jean and Abdallah, Nassim Ait and Melki, Isabelle | Outcome of COVID-19 in patients with rheumatic and inflammatory diseases treated with mycophenolic acid: data from the French RMD COVID-19 cohort | 2022 | Wrong outcome |
| Turkmen, M. and Dogan, S. | Certolizumab pegol in the treatment of psoriasis: Real-life data | 2021 | Wrong outcome |
| Tveita, A. and Murphy, S. L. and Holter, J. C. and Kildal, A. B. and Michelsen, A. E. and Lerum, T. V. and Kaarbo, M. and Heggelund, L. and Holten, A. R. and Finbraten, A. K. and Muller, K. E. and Mathiessen, A. and Boe, S. and Fevang, B. and Granerud, B. K. and Tonby, K. and Lind, A. and Dudman, S. G. and Henriksen, K. N. and Muller, F. and Skjonsberg, O. H. and Troseid, M. and Barratt-Due, A. and Dyrhol-Riise, A. M. and Aukrust, P. and Halvorsen, B. and Dahl, T. B. and Uel and , T. and consortium, Nor-Solidarity and Norwegian, Sars-CoV-Study Group investigators | High circulating levels of the homeostatic chemokines CCL19 and CCL21 predict mortality and disease severity in Covid-19 | 2022 | Wrong population |
| Udomkarnjananun, S. and Kerr, S. J. | Mortality risk factors of covid-19 infection in kidney transplantation recipients: A systematic review and meta-analysis of cohorts and clinical registries | 2021 | Duplicate |
| Vagonis, A. M. and MacMillan, A. C. and Kashiouris, M. G. and Jackson, P. D. and Mahmud, H. and Uber, R. K. and Mahashabde, M. and Nana-Sinkam, P. | Hospitalized COVID-19 patients with active lymphoma have eight times higher risk of death than COVID-19 controls | 2021 | Single centre |
| van Bremen, K. and Monin, M. and Schlabe, S. and Bischoff, J. and Rieke, G. J. and Schwarze-Z and er, C. and Wasmuth, J. C. and Rockstroh, J. K. and Boesecke, C. | Impact of COVID-19 on HIV late diagnosis in a specialized German centre | 2022 | Wrong outcome |
| van Wyngaard, A. and Whiteside, A. | AIDS and COVID-19 in southern Africa | 2021 | Insufficient original data |
| van Zeggeren, I. E. and Boelen, A. and van de Beek, D. and Heijboer, A. C. and Vlaar, A. P. J. and Brouwer, M. C. | Sex steroid hormones are associated with mortality in COVID-19 patients: Level of sex hormones in severe COVID-19 | 2021 | Wrong population |
| Várnai, C., Palles, C., Arnold, R., Curley, H.M., Purshouse, K., Cheng, V.W., Booth, S., Campton, N.A., Collins, G.P., Hughes, D.J. and Kulasekararaj, A.G | Erratum: Mortality among adults with cancer undergoing chemotherapy or immunotherapy and infected with COVID-19 (JAMA Netw Open. (2022) 5:2 (e220130) DOI: 10.1001/jamanetworkopen.2022.0130) | 2022 | Insufficient original data |
| Varshney, K. and Ghosh, P. and Stiles, H. and Iriowen, R. | Risk Factors for COVID-19 Mortality Among People Living with HIV: A Scoping Review | 2022 | Incorrect study design |
| Venditto, V. J. and Haydar, D. and Abdel-Latif, A. and Gensel, J. C. and Anstead, M. I. and Pitts, M. G. and Creameans, J. and Kopper, T. J. and Peng, C. and Feola, D. J. | Immunomodulatory Effects of Azithromycin Revisited: Potential Applications to COVID-19 | 2021 | Wrong outcome |
| Venturas, J. and Zamparini, J. and Shaddock, E. and Stacey, S. and Murray, L. and Richards, G. A. and Kalla, I. and Mahomed, A. and Mohamed, F. and Mer, M. and Maposa, I. and Feldman, C. | Comparison of outcomes in HIV-positive and HIV-negative patients with COVID-19 | 2021 | Single centre |
| Vergori, A. and Boschini, A. and Notari, S. and Lorenzini, P. and Castilletti, C. and Colavita, F. and Matusali, G. and Tartaglia, E. and Gagliardini, R. and Boschi, A. and Cimini, E. and Maeurer, M. and Piselli, P. and Angeli, L. and Antinori, A. and Agrati, C. and Girardi, E. | SARS-CoV-2 Specific Immune Response and Inflammatory Profile in Advanced HIV-Infected Persons during a COVID-19 Outbreak | 2022 | n<50 |
| Vetrano, Davide Liborio and Tazzeo, Clare and Palmieri, Luigi and Marengoni, Aless and ra and Zucchelli, Alberto and Lo Noce, Cinzia and Onder, Graziano | Comorbidity status of deceased COVID-19 in-patients in Italy | 2021 | Compound risk group |
| ViganÃ², C. and Mulinacci, G. and Palermo, A. and Barisani, D. and Pirola, L. and Fichera, M. and Invernizzi, P. and Massironi, S. | Impact of COVID-19 on inflammatory bowel disease practice and perspectives for the future | 2021 | Insufficient original data |
| Vinson, Am and a J and Agarwal, Gaurav and Dai, Ran and Anzalone, Alfred J and Lee, Stephen B and French, Evan and Olex, Amy and Madhira, Vithal and Mannon, Roslyn B | COVID-19 in solid organ transplantation: results of the national COVID cohort collaborative | 2021 | Wrong outcome |
| Virata, M. D. and Shenoi, S. V. and Ladines-Lim, J. and Villanueva, M. S. and Barakat, L. A. | Cumulative burden of non-communicable diseases predicts COVID hospitalization among people with HIV: A one-year retrospective cohort study | 2021 | Single centre |
| Vita, S. and Centanni, D. and Lanini, S. and Piselli, P. and Rosati, S. and Giancola, M. L. and Mondi, A. and Pinnetti, C. and Topino, S. and Chinello, P. and Mosti, S. and Gualano, G. and Faraglia, F. and Iacomi, F. and Marchioni, L. and Maritti, M. and Girardi, E. and Ippolito, G. and Nicastri, E. and On Behalf Of The Re, COVeRI Study Group | Benefits of Steroid Therapy in COVID-19 Patients with Different PaO(2)/FiO(2) Ratio at Admission | 2021 | Wrong outcome |
| Vivekanantham, A. and Burn, E. and Fern and ez-Bertolin, S. and Aragon, M. and Duarte-Salles, T. and Prieto-Alhambra, D. | Rheumatoid arthritis and the risk of COVID-19 diagnosis, hospitalisation and death: A population-based multi-state cohort analysis including 5,586,565 people in Catalonia, Spain | 2021 | Insufficient original data |
| von Hentig, N. | Repositioning HIV protease inhibitors and nucleos(t)ide RNA polymerase inhibitors for the treatment of SARS-CoV-2 infection and COVID-19 | 2021 | Wrong outcome |
| Wang, W. and Snell, L. B. and Ferrari, D. and Goodman, A. L. and Price, N. M. and Wolfe, C. D. and Curcin, V. and Edgeworth, J. D. and Wang, Y. | Real-world effectiveness of steroids in severe COVID-19: a retrospective cohort study | 2022 | Wrong population |
| Wang, X. S. | Angiotensin-converting enzyme 2 connects COVID-19 with cancer and cancer immunotherapy | 2021 | Wrong population |
| Wang, Z. P. and Hua, M. and Jiu, T. and Ge, R. L. and Bai, Z. | Biofunctional roles of estrogen in coronavirus disease 2019: Beyond a steroid hormone | 2022 | Wrong population |
| Wanyuan Cui, Dr and Yousaf, Nadia and Bhosle, Jaishree and Minchom, Anna and Nicholson, Andrew G and Merina Ahmed, Dr and McDonald, Fiona and Locke, Imogen and Lee, Richard and O'Brien, Mary | Real-world outcomes in thoracic cancer patients with severe acute respiratory syndrome Coronavirus 2 (COVID-19): single UK institution experience | 2020 | n<50 |
| Waterer, G. W. and Rello, J. | Steroids and COVID-19: We Need a Precision Approach, Not One Size Fits All | 2020 | Wrong population |
| Waters, Laura J and Pozniak, Anton L | COVID-19 death in people with HIV: interpret cautiously | 2021 | Incorrect study design |
| Webb, G. J. and Moon, A. M. and Barnes, E. and Barritt, A. S. and Marjot, T. | Determining risk factors for mortality in liver transplant patients with COVID-19 | 2020 | n<50 |
| Webb, G. J. and Moon, A. M. and Barnes, E. and Barritt, A. S. and Marjot, T. | Age and comorbidity are central to the risk of death from COVID-19 in liver transplant recipients | 2021 | Insufficient original data |
| Wetwittayakhlang, P. and Albader, F. and Golovics, P. A. and Hahn, G. D. and Bessissow, T. and Bitton, A. and Afif, W. and Wild, G. and Lakatos, P. L. | Clinical Outcomes of COVID-19 and Impact on Disease Course in Patients with Inflammatory Bowel Disease | 2021 | Single centre |
| Williams, S. V. and Whitaker, H. J. and Mumford, L. and Callaghan, C. and Curtis, R. M. K. and Stowe, J. and Kirsebom, F. and Thomas, J. and Ushiro-Lumb, I. and Ravanan, R. and Lopez-Bernal, J. | Effectiveness of COVID-19 Vaccines Against Hospitalization and Death with the SARS-CoV-2 Delta Variant in Solid Organ and Islet Transplant Recipients | 2022 | Wrong outcome |
| Wong, A. Y. and MacKenna, B. and Morton, C. E. and Schultze, A. and Walker, A. J. and Bhaskaran, K. and Brown, J. P. and Rentsch, C. T. and Williamson, E. and Drysdale, H. and Croker, R. and Bacon, S. and Hulme, W. and Bates, C. and Curtis, H. J. and Mehrkar, A. and Evans, D. and Inglesby, P. and Cockburn, J. and McDonald, H. I. and Tomlinson, L. and Mathur, R. and Wing, K. and Forbes, H. and Eggo, R. M. and Parry, J. and Hester, F. and Harper, S. and Evans, S. J. and Smeeth, L. and Douglas, I. J. and Goldacre, B. | Use of non-steroidal anti-inflammatory drugs and risk of death from COVID-19: an OpenSAFELY cohort analysis based on two cohorts | 2021 | Wrong population |
| Woo, S. and Kim, B. and Heo, N. H. and Kim, M. S. and Yoon, Y. A. and Choi, Y. J. | Association of Lupus Anticoagulant status with Disease Course in SARS-CoV-2 (COVID-19) Infection | 2022 | Wrong population |
| Wu, Julie Tsu-Yu and Kwon, Daniel H and Glover, Michael J and Henry, Solomon and Wood, Douglas and Rubin, Daniel L and Koshkin, Vadim S and Schapira, Lidia and Shah, Sumit A | Changes in cancer management due to COVID-19 illness in patients with cancer in Northern California | 2021 | Indirect mortality |
| Wu, Q. and Hu, Y. | Integrated network pharmacology and molecular docking strategy to explore the mechanism of medicinal and edible Astragali Radix-Atractylodis Macrocephalae Rhizoma acting on pneumonia via immunomodulation | 2020 | Incorrect study design |
| Xie, B. and Zhang, J. and Li, Y. and Yuan, S. and Shang, Y. | COVID-19: Imbalanced Immune Responses and Potential Immunotherapies | 2020 | Insufficient original data |
| Yan, C. and Niu, Y. and Wang, X. | Blood transcriptome analysis revealed the crosstalk between COVID-19 and HIV | 2022 | Wrong outcome |
| Yanes, R. R. and Malijan, G. M. B. and Escora-Garcia, L. K. and Ricafrente, S. A. M. and Salazar, M. J. and Suzuki, S. and Smith, C. and Ariyoshi, K. and Solante, R. M. and Edrada, E. M. and Takahashi, K. | Detection of SARS-CoV-2 and HHV-8 from a large pericardial effusion in an HIV-positive patient with COVID-19 and clinically diagnosed Kaposi sarcoma: a case report | 2022 | n<50 |
| Yang, D. and Li, H. and Chen, Y. and Ren, W. and Dong, M. and Li, C. and Jiao, Q. | Immunomodulatory mechanisms of abatacept: A therapeutic strategy for COVID-19 | 2022 | Wrong outcome |
| Yang, J. and Yu, M. and Fu, G. and Lan, G. and Li, L. and Qiao, Y. and Zhao, J. and Qian, H. Z. and Zhang, X. and Liu, X. and Jin, X. and Chen, G. and Fang, Y. and Wang, Z. and Xu, J. | COVID-19 Vaccination Uptake Among a Nationwide Sample of People Living With HIV During the Early Phase of Vaccine Rollout in China | 2022 | Wrong outcome |
| Yang, M. and Li, A. and Wang, Y. and Tran, C. and Zhao, S. and Ao, G. | Monoclonal antibody therapy improves severity and mortality of COVID-19 in organ transplant recipients: A meta-analysis | 2022 | Incorrect study design |
| Yarkan TuÄŸsal, H. and Ä°zdeÅŸ, S. and KÃ¼Ã§Ã¼kÅŸahin, O. | 20-Year-old female with fever, cough, and dyspnea: Acute lupus pneumonitis during the pandemic of coronavirus disease 2019 (COVID-19) | 2021 | n<50 |
| Yarlagadda, K. and Mi, K. and Sendil, S. and Koons, C. L. and Kom and uri, S. and Cinicola, J. T. | A 31-Year-Old Man with COVID-19-Associated Empyema and Lupus Anticoagulant | 2020 | n<50 |
| Yessayan, L. T. and Neyra, J. A. and Westover, A. J. and Szamosfalvi, B. and Humes, H. D. | Extracorporeal Immunomodulation Treatment and Clinical Outcomes in ICU COVID-19 Patients | 2022 | Wrong population |
| Yilmaz, F. and Yasar, S. and Tuncali, M. C. and Akin, S. | Complete response in a frail patient with high-grade B-cell lymphoma to only one cycle of R-CHOP or to prolonged COVID-19? | 2021 | Insufficient original data |
| Yilmaz, Gulay and Ebru, Ozdemir and Ibrahim, Berber and Ulkem, Cakir | Assessment of clinical outcomes in renal transplant recipients with COVID-19 | 2021 | Single centre |
| Yin, S. and Song, T. and Zhong, Q. and Lin, T. | Mortality, risk factors, and treatment of COVID-19 infection in solid organ transplants: A systematic review and meta-analysis | 2021 | Insufficient original data |
| Yousefifard, M. and Zali, A. and Zarghi, A. and Madani Neishaboori, A. and Hosseini, M. and Safari, S. | Non-steroidal anti-inflammatory drugs in management of COVID-19; A systematic review on current evidence | 2020 | Wrong population |
| Yuan, Q. and Haque, O. and Coe, T. M. and Markmann, J. F. | The Heterogenous Effect of COVID-19 on Liver Transplantation Activity and Waitlist Mortality in the United States | 2021 | Wrong outcome |
| Yusuf, A. S. and Cheong, X. K. and Rozita, M. and Periyasamy, P. and Ruslinda, M. | A case of lupus nephritis flare-up in severe COVID-19 infection | 2021 | n<50 |
| Zanetta, C. and Rocca, M. A. and Filippi, M. | Impact of immunotherapies on COVID-19 outcomes in multiple sclerosis patients | 2022 | Wrong population |
| Zargaran, M. and Movassaghi, S. and Seyyedsalehi, M. S. and Zendehdel, K. and Rostamian, A. | Outcomes of coronavirus disease 19 patients with a history of rheumatoid arthritis: A retrospective registry-based study in Iran | 2022 | n<50 |
| Zarifkar, P and Kamath, A and Robinson, C and Morgulchik, N and Shah, SFH and Cheng, TKM and Dominic, C and Fehintola, AO and Bhalla, G and Ahillan, T | Clinical characteristics and outcomes in patients with COVID-19 and cancer: a systematic review and meta-analysis | 2021 | Insufficient original data |
| Zayed, M. and Iohara, K. | Immunomodulation and Regeneration Properties of Dental Pulp Stem Cells: A Potential Therapy to Treat Coronavirus Disease 2019 | 2020 | Wrong population |
| Zhang, J. and Sheng, H. and Tang, X. and Xia, P. and Li, Z. and Xu, M. and Ma, J. and Shen, Y. and Yu, P. and Liu, X. | Non-steroidal anti-inflammatory drugs and clinical outcomes in patients with COVID-19 | 2022 | Wrong population |
| Zhou, Q. and Zhao, S. and Gan, L. and Wang, Z. and Peng, S. and Li, Q. and Liu, H. and Liu, X. and Wang, Z. and Shi, Q. and Estill, J. and Luo, Z. and Wang, X. and Liu, E. and Chen, Y. | Use of non-steroidal anti-inflammatory drugs and adverse outcomes during the COVID-19 pandemic: A systematic review and meta-analysis | 2022 | Wrong population |
| Zhou, Yangzhong and Ren, Qidong and Chen, Gang and Jin, Qiao and Cui, Quexuan and Luo, Huiting and Zheng, Ke and Qin, Yan and Li, Xuemei | Chronic kidney diseases and acute kidney injury in patients with COVID-19: evidence from a meta-analysis | 2020 | Wrong population |
| Zizzo, G. and Tamburello, A. and Castelnovo, L. and Laria, A. and Mumoli, N. and Faggioli, P. M. and Stefani, I. and Mazzone, A. | Immunotherapy of COVID-19: Inside and Beyond IL-6 Signalling | 2022 | Wrong population |
| Zuniga, M., Gomes, C., Carsons, S.E., Bender, M.T., Cotzia, P., Miao, Q.R., Lee, D.C. and Rodriguez, A. | Autoimmunity to annexin A2 predicts mortality among hospitalised COVID-19 patients | 2021 | Wrong population |

1. **Phase 2 Exclusion Justifications**

| **Authors** | **Title** | **Year** | **Exclusion Factor** |
| --- | --- | --- | --- |
| Abuhelwa, Z., Alsughayer, A., Abuhelwa, A.Y., Beran, A., Sayeh, W., Khokher, W., Sajdeya, O., Khuder, S. and Assaly, R. | Â In-Hospital Mortality and Morbidity in Cancer Patients with COVID-19: A Nationwide Analysis from the United States | 2022 | All-cause mortality |
| Akiyama, Shintaro and Hamdeh, Shadi and Micic, Dejan and Sakuraba, Atsushi | Prevalence and clinical outcomes of COVID-19 in patients with autoimmune diseases: a systematic review and meta-analysis | 2022 | Systematic Review |
| Alpert, N., Rapp, J.L., Marcellino, B., Lieberman-Cribbin, W., Flores, R. and Taioli, E | Clinical Course of Cancer Patients with COVID-19: A Retrospective Cohort Study | 2021 | All-cause mortality |
| Alpert, N., Rapp, J.L., Marcellino, B., Lieberman-Cribbin, W., Flores, R. and Taioli, E. | Clinical course of cancer patients with COVID-19: a retrospective cohort study | 2021 | Duplication |
| Alrashed, F. and Alasfour, H. and Shehab, M. | Impact of biologics and small molecules for inflammatory bowel disease on COVID-19-related hospitalization and mortality: A systematic review and meta-analysis | 2021 | Systematic Review |
| Alrashed, F. and Battat, R. and Abdullah, I. and Charabaty, A. and Shehab, M. | Impact of medical therapies for inflammatory bowel disease on the severity of COVID-19: a systematic review and meta-analysis | 2021 | Systematic Review |
| Alshaqaq, A., Al Abadi, A., Altheaby, A., Bukhari, M.A., Nasrallah, B., Alamoudi, A., Arabi, Z., Alhejaili, F., Saad, K.B., Busbaih, A. and Idris, M.A., | Coronavirus disease 2019 and kidney transplantation in Saudi Arabia: outcomes and future opportunities | 2021 | Insufficient data |
| Altuntas, Fevzi and Ata, Naim and Yigenoglu, Tugce Nur and BascÄ±, Semih and Dal, Mehmet Sinan and Korkmaz, Serdal and Namdaroglu, Sinem and Basturk, Abdulkadir and HacÄ±bekiroglu, Tuba and Dogu, Mehmet Hilmi | COVID-19 in hematopoietic cell transplant recipients | 2021 | n<50 |
| Anushiravani, A., Saberzadeh-Ardestani, B., Vahedi, H., Fakheri, H., Mansour-Ghanaei, F., Maleki, I., Nasseri-Moghaddam, S., Vosoghinia, H., Ghadir, M.R., Hormati, A. and Kasaeian, A | Susceptibility of Patients with Inflammatory Bowel Disease to COVID-19 Compared with Their Households | 2022 | Insufficient data |
| Ao, G. and Wang, Y. and Qi, X. and Nasr, B. and Bao, M. and Gao, M. and Sun, Y. and Xie, D. | The association between severe or death COVID-19 and solid organ transplantation: A systematic review and meta-analysis | 2021 | Systematic Review |
| Ascierto, P.A., Secrest, M.H., Lambert, P., Sarsour, K., Tan, A., Walls, R., Reddy, J., Seetasith, A., Shenison, D., Ngwa, I. and Yun, C. | Mortality of 1,636 COVID-19 cancer patients (pts) and associated prognostic factors | 2021 | Insufficient data |
| Avery, Robin K and Chiang, Teresa Poâ€Yu and Marr, Kieren A and Brennan, Daniel C and Sait, Afrah S and Garibaldi, Brian T and Shah, Pali and Ostr and er, Darin and Steinke, Seema Mehta and Permpalung, Nitipong | Inpatient COVID-19 outcomes in solid organ transplant recipients compared to nonâ€solid organ transplant patients: a retrospective cohort | 2021 | n<50 |
| Axelrad, J.E., Malter, L., Hong, S., Chang, S., Bosworth, B. and Hudesman, D. | From the American epicenter: coronavirus disease 2019 in patients with inflammatory bowel disease in the New York City metropolitan area.Â | 2021 | Single Centre |
| Ayala Gutierrez, M.D.M., Rubio-Rivas, M., Romero Gómez, C., Montero Saez, A., Pérez de Pedro, I., Homs, N., Ayuso García, B., Cuenca Carvajal, C., Arnalich Fernández, F., Beato Perez, J.L. and Vargas Nunez, J.A. | On Behalf of The Semi-COVID-Network. Autoimmune Diseases and COVID-19 as Risk Factors for Poor Outcomes: Data on 13,940 Hospitalized Patients from the Spanish Nationwide SEMI-COVID-19 Registry | 2021 | Systematic Review |
| Bamias, G. and Kokkotis, G. and Christidou, A. and Christodoulou, D. K. and Delis, V. and Diamantopoulou, G. and Fessatou, S. and Gatopoulou, A. and Giouleme, O. and Kafritsa, P. and Kalantzis, C. and Kapsoritakis, A. and Karatzas, P. and Karmiris, K. and Katsanos, K. and Kevrekidou, P. and Kosmidis, C. and Mantaka, A. and Mathou, N. and Michalopoulos, G. and Michopoulos, S. and Papaconstantinou, I. and Papatheodoridis, G. and Polymeros, D. and Potamianos, S. and Poulopoulos, G. and Protopapas, A. and Sklavaina, M. and Soufleris, K. and Theocharis, G. and Theodoropoulou, A. and Triantafillidis, J. K. and Triantafyllou, K. and Tsiolakidou, G. and Tsironi, E. and Tzouvala, M. and Viazis, N. and Xourgias, V. and Zacharopoulou, E. and Zampeli, E. and Mantzaris, G. J. | The natural history of COVID-19 in patients with inflammatory bowel disease: a nationwide study by the Hellenic Society for the study of IBD | 2021 | Insufficient data |
| Banjongjit, A. and Larpparisuth, N. and Phonphok, K. and Homkrailas, P. and Traitanon, O. and Inkong, P. and Wongpraphairot, S. and Lumpaopong, A. and Supaporn, T. and Pongskul, C. | Prognostic Factors for Mortality of COVID-19 Infection in Kidney Transplant Recipients in Thailand: A Nationwide Study | 2022 | Insufficient data |
| Banna, G. L. and Cantale, O. and Friedlaender, A. and Yusof, H. and Acharige, S. and Addeo, A. | Risk of SARS-CoV2-Related Mortality in Non-Small Cell Lung Cancer Patients Treated with First-Line Immunotherapy Alone or in Combination with Chemotherapy | 2022 | Insufficient data |
| Barlesi, F., Foulon, S., Bayle, A., Gachot, B., Pommeret, F., Willekens, C., Stoclin, A., Merad, M., GriscelliI, F., Micol, J.B. and Sun, R | Outcome of cancer patients infected with COVID-19, including toxicity of cancer research. | 2020 | Single Centre |
| Bazgir, N., Taghinezhad, F., Nourmohammadi, H., Azami, G., Ahmadi, I. and Mozafari, A. | Comparing the COVID-19 Mortality Rate in Cancer Patients with and Without a History of Chemotherapy | 2022 | Single Centre |
| Bennett, C.L., Ogele, E., Pettit, N.R., Bischof, J.J., Meng, T., Govindarajan, P., Camargo Jr, C.A., Nordenholz, K., Kline, J.A. and RECOVER Investigators | Â Multicenter Study of Outcomes Among Persons With HIV Who Presented to US Emergency Departments With Suspected SARS-CoV-2 | 2021 | Duplication |
| Berger, B. and Hazzan, M. and Kamar, N. and Francois, H. and Matignon, M. and Greze, C. and Gatault, P. and Frimat, L. and Westeel, P. F. and Goutaudier, V. and Snanoudj, R. and Colosio, C. and Sicard, A. and Bertr and , D. and Mousson, C. and Bamoulid, J. and Thierry, A. and Anglicheau, D. and Couzi, L. and Chemouny, J. M. and Duveau, A. and Moal, V. and Le Meur, Y. and Blancho, G. and Tourret, J. and Malvezzi, P. and Mariat, C. and Rerolle, J. P. and Bouvier, N. and Caillard, S. and Thaunat, O. and French Solid Organ Transplant, Covid Registry | Absence of Mortality Differences Between the First and Second COVID-19 Waves in Kidney Transplant Recipients | 2022 | Insufficient data |
| Berger, J.M., Wohlfarth, P., Königsbrügge, O., Knaus, H.A., Porpaczy, E., Kaufmann, H., Schreiber, J., Mrva-Ghukasyan, T., Winder, T., Severgnini, L. and Wolf, D., | SARS-CoV-2-related mortality and treatment delays for cancer patients in Austria: Findings of a multicentric nationwide study | 2022 | Insufficient data |
| Bertagnolio, S. and Thwin, S. S. and Silva, R. and Nagarajan, S. and Jassat, W. and Fowler, R. and Haniffa, R. and Reveiz, L. and Ford, N. and Doherty, M. and Diaz, J. | Clinical features of, and risk factors for, severe or fatal COVID-19 among people living with HIV admitted to hospital: analysis of data from the WHO Global Clinical Platform of COVID-19 | 2022 | Paediatric |
| Bertoglio, I.M., Valim, J.M.D.L., Daffre, D., Aikawa, N.E., Silva, C.A., Bonfá, E. and Ugolini‐Lopes, M.R. | Poor prognosis of COVID-19 acute respiratory distress syndrome in lupus erythematosus: nationwide cross-sectional population study of 252 119 patients. | 2021 | Duplication |
| Bezzio, C. and Armuzzi, A. and Furfaro, F. and Ardizzone, S. and Milla, M. and Carparelli, S. and Orl and o, A. and Caprioli, F. A. and Castiglione, F. and ViganÃ², C. and Ribaldone, D. G. and Zingone, F. and Monterubbianesi, R. and Imperatore, N. and Festa, S. and Daperno, M. and Scucchi, L. and Ferronato, A. and Pastorelli, L. and Balestrieri, P. and Ricci, C. and Cappello, M. and Felice, C. and Fiorino, G. and Saibeni, S. | Therapies for inflammatory bowel disease do not pose additional risks for adverse outcomes of SARS-CoV-2 infection: an IG-IBD study | 2021 | Insufficient data |
| Bhogal, T. and Khan, U. T. and Lee, R. and Stockdale, A. and Hesford, C. and Potti-Dhananjaya, V. and Jathanna, A. and Rahman, S. and Tivey, A. and Shotton, R. and Sundar, R. and Valerio, C. and Norouzi, A. and Walker, P. and Suckling, R. and Armstrong, A. and Brearton, G. and Pettitt, A. and Kalakonda, N. and Palmer, D. H. and Jackson, R. and Turtle, L. and Palmieri, C. | Haematological malignancy and nosocomial transmission are associated with an increased risk of death from COVID-19: results of a multi-center UK cohort | 2021 | Insufficient data |
| Bondeson, L., Thulin, A., Ny, L., Levin, M., Svensson, J., Lindh, M. and Zhao, Z. | Clinical outcomes in cancer patients with COVID-19 in Sweden | 2021 | Single Centre |
| Booth, S. and Curley, H. M. and Varnai, C. and Arnold, R. and Lee, L. Y. W. and Campton, N. A. and Cook, G. and Purshouse, K. and Aries, J. and Innes, A. and Cook, L. B. and Tomkins, O. and Oram, H. S. and Tilby, M. and Kulasekararaj, A. and Wrench, D. and Dolly, S. and Newsom-Davies, T. and Pettengell, R. and Gault, A. and Moody, S. and Mittal, S. and Altohami, M. and Tillet, T. and Illingworth, J. and Mukherjee, L. and Apperly, J. and Ashcroft, J. and Rabin, N. and Carmichael, J. and Cazier, J. B. and Kerr, R. and Middleton, G. and Collins, G. P. and Palles, C. | Key findings from the UKCCMP cohort of 877 patients with haematological malignancy and COVID-19: disease control as an important factor relative to recent chemotherapy or anti-CD20 therapy | 2022 | Insufficient data |
| Booth, S. and Willan, J. and Wong, H. and Khan, D. and Farnell, R. and Hunter, A. and Eyre, T. and Katz, H. and Dungarwalla, M. and Chen, L. and Browning, J. and Polzella, P. and Gray, N. and Neelakantan, P. and Dhillon, E. K. and Dutton, D. and Sternberg, A. and Prideaux, S. and Collins, G. P. and Peniket, A. | Regional outcomes of severe acute respiratory syndrome coronavirus 2 infection in hospitalised patients with haematological malignancy | 2020 | Insufficient data |
| Braunstein, S.L., Lazar, R., Wahnich, A., Daskalakis, D.C. and Blackstock, O.J. | Coronavirus Disease 2019 (COVID-19) Infection Among People With Human Immunodeficiency Virus in New York City: A Population-Level Analysis of Linked Surveillance Data | 2021 | Paediatric |
| Breccia, M. and Abruzzese, E. and Accurso, V. and Attolico, I. and Barulli, S. and Bergamaschi, M. and Binotto, G. and Bocchia, M. and Bonifacio, M. and Caocci, G. and Capodanno, I. and Castagnetti, F. and Cavazzini, F. and CrisÃ , E. and Crugnola, M. and Stella De C and ia, M. and Elena, C. and Fava, C. and Galimberti, S. and Gozzini, A. and Gugliotta, G. and Intermesoli, T. and Iurlo, A. and La Barba, G. and Latagliata, R. and Leonetti Crescenzi, S. and Levato, L. and Loglisci, G. and Lucchesi, A. and Luciano, L. and Lunghi, F. and Luzi, D. and Malato, A. and Cristina Miggiano, M. and Pizzuti, M. and Pregno, P. and Rapezzi, D. and Rege-Cambrin, G. and Rosti, G. and Russo, S. and Sancetta, R. and Rita Scortechini, A. and SorÃ , F. and Sportoletti, P. and Stagno, F. and Tafuri, A. and Tiribelli, M. and FoÃ , R. and Saglio, G. | COVID-19 infection in chronic myeloid leukaemia after one year of the pandemic in Italy. A Campus CML report | 2023 | Insufficient data |
| Brown, A.E., Croxford, S.E., Nash, S., Khawam, J., Kirwan, P., Kall, M., Bradshaw, D., Sabin, C., Miller, R.F., Post, F.A. and Harding, R. | COVID-19 mortality among people with diagnosed HIV compared to those without during the first wave of the COVID-19 pandemic in England.Â | 2022 | Paediatric |
| Bruera, S., Lei, X., Zhao, H., Yazdany, J., Chavez-MacGregor, M., Giordano, S.H. and Suarez-Almazor, M.E. | Risks of mortality and severe coronavirus disease 19 (COVID-19) outcomes in patients with or without systemic lupus erythematosus | 2023 | 2023 |
| Cabello, A., Zamarro, B., Nistal, S., Victor, V., Hernández, J., Prieto-Pérez, L., Carrillo, I., Álvarez, B., Fernández-Roblas, R., Hernández-Segurado, M. and Becares, J. | COVID-19 in people living with HIV: A multicenter case-series study | 2021 | Duplication |
| Caillard, S. and Anglicheau, D. and Matignon, M. and Durrbach, A. and Greze, C. and Frimat, L. and Thaunat, O. and Legris, T. and Moal, V. and Westeel, P. F. and Kamar, N. and Gatault, P. and Snanoudj, R. and Sicard, A. and Bertr and , D. and Colosio, C. and Couzi, L. and Chemouny, J. M. and Masset, C. and Blancho, G. and Bamoulid, J. and Duveau, A. and Bouvier, N. and Chavarot, N. and Grimbert, P. and Moulin, B. and Le Meur, Y. and Hazzan, M. and French, S. O. T. Covid Registry | An initial report from the French SOT COVID Registry suggests high mortality due to Covid-19 in recipients of kidney transplants | 2020 | Insufficient data |
| Caillard, S. and Chavarot, N. and Francois, H. and Matignon, M. and Snanoudj, R. and Tourret, J. and Greze, C. and Thaunat, O. and Frimat, L. and Westeel, P. F. and Gatault, P. and Masset, C. and Blancho, G. and Legris, T. and Moal, V. and Kamar, N. and Jdidou, M. and Colosio, C. and Mousson, C. and Goutadier, V. and Sicard, A. and Bertr and , D. and Bamoulid, J. and Malvezzi, P. and Couzi, L. and Chemouny, J. M. and Duveau, A. and Mariat, C. and Rerolle, J. P. and Thierry, A. and Bouvier, N. and Anglicheau, D. and Le Meur, Y. and Hazzan, M. and Moulin, B. and Fafi-Kremer, S. and Hertig, A. and Barrou, B. and Morelon, E. and Merville, P. and Westeel, P. F. and Jaureguy, M. and Ducloux, D. and Tsimaratos, M. and Garaix-Gilardo, F. and Dumortier, J. and Mussot, S. and Roux, A. and Sebbag, L. and Rondeau, E. and Buchler, M. and Augusto, J. F. and Vigneau, C. and Morin, M. C. and Chemouny, J. and Golbin, L. and Grimbert, P. and Durrbach, A. and Schvartz, B. and Le Quintrec, M. and Rerolle, J. P. and Heng, A. E. and Garrouste, C. and Coponat, H. V. and Epailly, E. and Brugiere, O. and Dharancy, S. and Salame, E. and Saliba, F. | Clinical Utility of Biochemical Markers for the Prediction of COVID-19-Related Mortality in Kidney Transplant Recipients | 2021 | Insufficient data |
| Caillard, S., Thaunat, O. and Hazzan, M. | Covid-19 in kidney transplantation: Lessons from the French Registry | 2021 | Duplication |
| Calderón-Parra, J., Cuervas-Mons, V., Moreno-Torres, V., Rubio-Rivas, M., Agudo-de Blas, P., Pinilla-Llorente, B., Helguera-Amezua, C., Jiménez-García, N., Pesqueira-Fontan, P.M., Méndez-Bailón, M. and Artero, A | Influence of chronic use of corticosteroids and calcineurin inhibitors on COVID-19 clinical outcomes: analysis of a nationwide registry | 2022 | All-cause mortality |
| Candellier, A. and Jean Goffin, E. and Vart, P. and Noordzij, M. and Arnol, M. and Covic, A. and Lentini, P. and Malik, S. and Reichert, L. and Sever, M. S. and Watschinger, B. and Jager, K. J. and Gansevoort, R. | COVID-19 related mortality in kidney transplant and dialysis patients: A comparative, prospective registry based study | 2020 | Insufficient data |
| Cao, C. and Gan, X. and Hu, X. and Su, Y. and Zhang, Y. and Peng, X. | Association of active immunotherapy with outcomes in cancer patients with COVID-19: a systematic review and meta-analysis | 2022 | Systematic Review |
| Chanda, D., Minchella, P.A., Kampamba, D., Itoh, M., Hines, J.Z., Fwoloshi, S., Boyd, M.A., Hamusonde, K., Chirwa, L., Nikoi, K. and Chirwa, R. | COVID-19 severity and COVID-19-associated deaths among hospitalized patients with HIV infectionâ€“Zambia, | 2021 | Paediatric |
| Chauhan, S., Meshram, H.S., Kute, V.B., Patel, H., Deshmukh, S., Desai, S., Dave, R. and Banerjee, S. | Comparative Analysis of Kidney Transplant Recipients with Severe Acute Respiratory Syndrome Coronavirus 2 Compared with Nonkidney Transplant | 2022 | Single Centre |
| Creemers, R.H., Rezazadeh Ardabili, A., Jonkers, D.M., Leers, M.P., Romberg-Camps, M.J., Pierik, M.J. and van Bodegraven, A.A | Severe COVID-19 in inflammatory bowel disease patients in a population-based setting | 2021 | Insufficient data |
| D’Silva, K.M., Jorge, A., Cohen, A., McCormick, N., Zhang, Y., Wallace, Z.S. and Choi, H.K | COVID-19 outcomes in patients with systemic autoimmune rheumatic diseases compared to the general population: a US multicenter, comparative cohort study. | 2021 | Duplication |
| Dachi, D. and Geiger, G. and Montgomery, M. W. and Karmen-Tuohy, S. and Golzy, M. and Antar, A. A. R. and Llibre, J. M. and Camazine, M. and DÃ­az-De Santiago, A. and Carlucci, P. M. and Zacharioudakis, I. M. and Rahimian, J. and Wanjalla, C. N. and Slim, J. and Arinze, F. and Kratz, A. M. P. and Jones, J. L. and Patel, S. M. and Kitchell, E. and Francis, A. and Ray, M. and Koren, D. E. and Baddley, J. W. and Hill, B. and Sax, P. E. and Chow, J. | Characteristics, Comorbidities, and Outcomes in a Multicenter Registry of Patients With Human Immunodeficiency Virus and Coronavirus Disease 2019 | 2021 | Insufficient data |
| Damiani, G., Pacifico, A., Bragazzi, N.L. and Malagoli, P | Biologics increase the risk of SARS-CoV-2 infection and hospitalization, but not ICU admission and death: Real-life data from a large cohort during red-zone declaration | 2020 | Single Centre |
| Davies, M.A. | HIV and risk of COVID-19 death: a population cohort study from the Western Cape Province, South Africa. | 2020 | Duplication |
| de Medeiros, K. S. and da Silva, L. A. S. and MacÃªdo, L. T. A. and Sarmento, A. C. and Costa, A. P. F. and EleutÃ©rio, J., Jr. and GonÃ§alves, A. K. | Potential impact of the COVID-19 in HIV-infected individuals: a systematic review Impact of the COVID-19 in HIV-Infected Individuals | 2021 | Systematic Review |
| Del Amo, J. and Polo, R. and Moreno, S. and DÃ­az, A. and MartÃ­nez, E. and Arribas, J. R. and JarrÃ­n, I. and HernÃ¡n, M. A. | Incidence and Severity of COVID-19 in HIV-Positive Persons Receiving Antiretroviral Therapy : A Cohort Study | 2022 | Insufficient data |
| Del Amo, J. and Polo, R. and Moreno, S. and MartÃ­nez, E. and Cabello, A. and Iribarren, J. A. and Curran, A. and MacÃ­as, J. and Montero, M. and DueÃ±as, C. and MariÃ±o, A. I. and de la CÃ¡mara, S. P. and DÃ­az, A. and Arribas, J. R. and JarrÃ­n, I. and HernÃ¡n, M. A. | Tenofovir disoproxil fumarate/emtricitabine and severity of coronavirus disease 2019 in people with HIV infection | 2022 | Insufficient data |
| Derikx, L.A., Lantinga, M.A., de Jong, D.J., van Dop, W.A., Creemers, R.H., Römkens, T.E., Jansen, J.M., Mahmmod, N. | Clinical Outcomes of Covid-19 in Patients With Inflammatory Bowel Disease: A Nationwide Cohort Study | 2021 | Insufficient data |
| Desai, Aakash and Gupta, Rohit and Advani, Shailesh and Ouellette, Lara and Kuderer, Nicole M and Lyman, Gary H and Li, Ang | Mortality in hospitalized patients with cancer and coronavirus disease 2019: a systematic review and metaâ€analysis of cohort studies | 2021 | Systematic Review |
| Dulery, R. and Lamure, S. and Delord, M. and Di Blasi, R. and Chauchet, A. and Hueso, T. and Rossi, C. and Drenou, B. and Deau Fischer, B. and Soussain, C. and Feugier, P. and NoÃ«l, N. and Choquet, S. and Bologna, S. and Joly, B. and Philippe, L. and Kohn, M. and Malak, S. and Fouquet, G. and Daguindau, E. and Taoufik, Y. and Lacombe, K. and Cartron, G. and ThiÃ©blemont, C. and Besson, C. | Prolonged in-hospital stay and higher mortality after Covid-19 among patients with non-Hodgkin lymphoma treated with B-cell depleting immunotherapy | 2021 | Insufficient data |
| Durstenfeld, M.S., Sun, K., Ma, Y., Rodriguez, F., Secemsky, E.A., Parikh, R.V. and Hsue, P.Y | Impact of HIV Infection on COVID-19 Outcomes Among Hospitalized Adults in the U.S. | 2021 | Duplication |
| Elias, Michelle and Pievani, Daniele and R and oux, Christine and Louis, Kevin and Denis, Bl and ine and Delion, Alex and ra and Le Goff, OcÃ©ane and Antoine, Corinne and Greze, Clarisse and Pillebout, Evangeline | COVID-19 infection in kidney transplant recipients: disease incidence and clinical outcomes | 2020 | Insufficient data |
| Engl and , B. R. and Roul, P. and Yang, Y. and Kalil, A. C. and Michaud, K. and Thiele, G. M. and Sauer, B. C. and Baker, J. F. and Mikuls, T. R. | Risk of COVID-19 in Rheumatoid Arthritis: A National Veterans Affairs Matched Cohort Study in At-Risk Individuals | 2021 | Compound outcome |
| England, B.R., Roul, P., Yang, Y., Kalil, A.C., Michaud, K., Thiele, G.M., Sauer, B.C., Baker, J.F. and Mikuls, T.R | Risk of COVID-19 in rheumatoid arthritis: a national veterans affairs matched cohort study in at-risk individuals.Â | 2021 | Compound outcome |
| Eybpoosh, S., Afshari, M., Haghdoost, A.A., Kazerooni, P.A., Gouya, M.M. and Tayeri, K. | Severity and mortality of COVID-19 infection in HIV -infected individuals: Preliminary findings from Iran. | 2021 | Insufficient data |
| Favara, G. and Barchitta, M. and Maugeri, A. and Faro, G. and Agodi, A. | HIV infection does not affect the risk of death of COVID-19 patients: A systematic review and meta-analysis of epidemiological studies | 2022 | Systematic Review |
| Faye, A.S., Lee, K.E., Laszkowska, M., Kim, J., Blackett, J.W., McKenney, A.S., Krigel, A., Giles, J.T., Wang, R., Bernstein, E.J. and Green, P.H | Risk of adverse outcomes in hospitalized patients with autoimmune disease and COVID-19: a matched cohort study from New York City | 2021 | Single Centre |
| Ferri, C. and Raimondo, V. and Gragnani, L. and Giuggioli, D. and Dagna, L. and Tavoni, A. and Ursini, F. and L'Andolina, M. and Caso, F. and Ruscitti, P. and Caminiti, M. and Foti, R. and Riccieri, V. and Guiducci, S. and Pellegrini, R. and Zanatta, E. and Varcasia, G. and Olivo, D. and Gigliotti, P. and Cuomo, G. and Murdaca, G. and Cecchetti, R. and De Angelis, R. and Romeo, N. and Ingegnoli, F. and Cozzi, F. and Codullo, V. and Cavazzana, I. and Colaci, M. and Abignano, G. and De Santis, M. and Lubrano, E. and Fusaro, E. and Spinella, A. and Lumetti, F. and De Luca, G. and Bell and o R and one, S. and Visalli, E. and Dal Bosco, Y. and Amato, G. and Giannini, D. and Bilia, S. and Masini, F. and Pellegrino, G. and Pigatto, E. and Generali, E. and Pagano Mariano, G. and Pettiti, G. and Zanframundo, G. and Brittelli, R. and Aiello, V. and Caminiti, R. and Scorpiniti, D. and Ferrari, T. and Campochiaro, C. and Brusi, V. and Fredi, M. and Moschetti, L. and Cacciapaglia, F. and Ferrari, S. M. and Di Cola, I. and Vadacca, M. and Lorusso, S. and Monti, M. and Lorini, S. and Paparo, S. R. and Ragusa, F. and Elia, G. and Mazzi, V. and Aprile, M. L. and Tasso, M. and Miccoli, M. and Bosello, S. L. and D'Angelo, S. and Doria, A. and Franceschini, F. and Meliconi, R. and Matucci-Cerinic, M. and Iannone, F. and Giacomelli, R. and Salvarani, C. and Zignego, A. L. and Fallahi, P. and Antonelli, A. | LONG-TERM SURVEY STUDY of the IMPACT of COVID-19 on SYSTEMIC AUTOIMMUNE DISEASES. LOW DEATH RATE DESPITE the INCREASED PREVALENCE of SYMPTOMATIC INFECTION. ROLE of PRE-EXISTING INTERSTITIAL LUNG DISEASE and ONGOING TREATMENTS | 2022 | Insufficient data |
| Fisher, A.M., Schlauch, D., Mulloy, M., Dao, A., Reyad, A.I., Correll, M., Fromell, G.J., Pittman, J., Bingaman, A.W., Sankarapandian, B. and Allam, S.R. | Outcomes of COVID-19 in hospitalized solid organ transplant recipients compared to a matched cohort of non-transplant pa- tients at a national healthcare system in the United States | 2021 | Duplication |
| Flannery, S., Schwartz, R., Rasul, R., Hirschwerk, D.A., Wallach, F., Hirsch, B. and McGowan, J., | A comparison of COVID-19 inpatients by HIV status. | 2021 | Duplication |
| Gagliardini, R., Vergori, A., Lorenzini, P., Cicalini, S., Pinnetti, C., Mazzotta, V., Mondi, A., Mastrorosa, I., Camici, M., Lanini, S. and Fusto, M. | Characteristics and outcomes of COVID-19-related hospitalization among PLWH | 2022 | Insufficient data |
| GarcÃ­a-SuÃ¡rez, Julio and De La Cruz, Javier and Cedillo, Ãngel and Llamas, Pilar and Duarte, Rafael and JimÃ©nez-Yuste, VÃ­ctor and HernÃ¡ndez-Rivas, JosÃ© Ãngel and Gil-Manso, Rodrigo and Kwon, Mi and SÃ¡nchez-Godoy, Pedro | Impact of hematologic malignancy and type of cancer therapy on COVID-19 severity and mortality: lessons from a large population-based registry study | 2020 | Insufficient data |
| Garnica, M. and De Queiroz Crusoe, E. and Ribeiro, G. and Bittencourt, R. and Magalhaes, R. J. P. and Zanella, K. R. and Neto, A. E. H. and Lima, J. and Sola, C. and Souza, E. G. and Magalhaes, A. and Maiolino, A. and Hungria, V. | COVID-19 in multiple myeloma patients: Frequencies and risk factors for hospitalization, ventilatory support, intensive care admission and mortality -cooperative registry from grupo brasileiro de mieloma multiplo (GBRAM) | 2023 | 2023 |
| Genuardi, Michael V and Moss, Noah and Najjar, Samer S and Houston, Brian A and Shore, Supriya and Vorovich, Esther and Atluri, Pavan and Molina, Maria and Chambers, Susan and Sharkoski, Tiffany | Coronavirus disease 2019 in heart transplant recipients: Risk factors, immunosuppression, and outcomes | 2021 | Insufficient data |
| Gerard, A. O. and Barbosa, S. and Anglicheau, D. and Couzi, L. and Hazzan, M. and Thaunat, O. and Blancho, G. and Caillard, S. and Sicard, A. | Association Between Maintenance Immunosuppressive Regimens and COVID-19 Mortality in Kidney Transplant Recipients | 2020 | Insufficient data |
| Giannakoulis, V.G., Papoutsi, E. and Siempos, I.I. | Effect of cancer on clinical outcomes of patients with COVID‐19: a meta‐analysis of patient data' - meta analysis. | 2020 | Systematic Review |
| Giannakoulis, Vassilis G and Papoutsi, Eleni and Siempos, Ilias I | Effect of cancer on clinical outcomes of patients with COVID-19: a meta-analysis of patient data | 2020 | Duplication |
| Gisondi, P., Zaza, G., Del Giglio, M., Rossi, M., Iacono, V. and Girolomoni, G. | Risk of hospitalization and death from COVID-19 infection in patients with chronic plaque psoriasis receiving a biologic treatment and renal transplant recipients in maintenance immunosuppressive treatment. | 2020 | Single Centre |
| Goffin, Eric and C and ellier, Alex and re and Vart, Priya and Noordzij, Marlies and Arnol, Miha and Covic, Adrian and Lentini, Paolo and Malik, Shafi and Reichert, Louis J and Sever, Mehmet S | COVID-19-related mortality in kidney transplant and haemodialysis patients: a comparative, prospective registry-based study | 2021 | Insufficient data |
| Hadi, Y.B., Naqvi, S.F., Kupec, J.T. and Sarwari, A.R | Characteristics and outcomes of COVID-19 in patients with HIV: a multicentre research network study | 2020 | Duplication |
| Han, X. and Hou, H. and Xu, J. and Ren, J. and Li, S. and Yang, H. and Wang, Y. | Significant association between HIV infection and increased risk of COVID-19 mortality: a meta-analysis based on adjusted effect estimates | 2021 | Systematic Review |
| Hariyanto, T. I. and Kurniawan, A. | MO33-5 The impact of chemotherapy for cancer patients with COVID-19 on severity and mortality outcomes: A meta-analysis | 2021 | Insufficient data |
| Hariyanto, T. I. and Rosalind, J. and Christian, K. and Kurniawan, A. | Human immunodeficiency virus and mortality from coronavirus disease 2019: A systematic review and meta-analysis | 2021 | Systematic Review |
| Hasseli, R. and Pfeil, A. and Hoyer, B. F. and Krause, A. and Lorenz, H. M. and Richter, J. G. and Schmeiser, T. and Voll, R. E. and Schulze-Koops, H. and Specker, C. and MÃ¼ller-Ladner, U. | Do patients with rheumatoid arthritis show a different course of COVID-19 compared to patients with spondyloarthritis? | 2021 | Insufficient data |
| He, W., Chen, L., Chen, L., Yuan, G., Fang, Y., Chen, W., Wu, D., Liang, B., Lu, X., Ma, Y. and Li, L | COVID-19 in persons with haematological cancers | 2020 | Insufficient data |
| Heidary, M. and Asadi, A. and Noorbakhsh, N. and Dashtbin, S. and Asadollahi, P. and Dranb and i, A. and Navidifar, T. and Ghanavati, R. | COVID-19 in HIV-positive patients: A systematic review of case reports and case series | 2022 | Systematic Review |
| Heldman, M. R. and Kates, O. S. and Rakita, R. M. and Lease, E. D. and Limaye, A. P. and Fisher, C. E. | Delayed Mortality among Solid Organ Transplant Recipients Hospitalized for Covid-19: An International Multicenter Study | 2021 | Insufficient data |
| Heldman, Madeleine R and Kates, Olivia S and Safa, Kassem and Kotton, Camille N and Georgia, Sarah J and Steinbrink, Julie M and Alex and er, Barbara D and Hemmersbachâ€Miller, Marion and Blumberg, Emily A and Multani, Ashrit | Changing trends in mortality among solid organ transplant recipients hospitalized for COVID-19 during the course of the pandemic | 2022 | Insufficient data |
| Hilbr and s, L. B. and Duivenvoorden, R. and Vart, P. and Franssen, C. F. M. and Hemmelder, M. H. and Jager, K. J. and Kieneker, L. M. and Noordzij, M. and Pena, M. J. and de Vries, H. and Arroyo, D. and Covic, A. and Crespo, M. and Goffin, E. and Islam, M. and Massy, Z. A. and Montero, N. and Oliveira, J. P. and Munoz, A. R. and Sanchez, J. E. and Sridharan, S. and Winzeler, R. and Gansevoort, R. T. and van der Net, J. B. and Essig, M. and du Buf-Vereijken, P. W. G. and van Ginneken, B. and Maas, N. and Vogt, L. and van Jaarsveld, B. C. and Bemelman, F. J. and Klingenberg-Salahova, F. and Heenan-Vos, F. and Vervloet, M. G. and Nurmohamed, A. and Abramowicz, D. and Verhofstede, S. and Maoujoud, O. and Fialova, J. and Melilli, E. and Fava, A. and Cruzado, J. M. and Lips, J. and Hengst, M. and Gellert, R. and Rydzewski, A. and Alferes, D. G. and Rychlik, I. and Zakharova, E. V. and Ambuehl, P. M. and Lepeytre, F. and Rabate, C. and Rostoker, G. and Marques, S. and Azasevac, T. and Katicic, D. and ten Dam, M. and Kruger, T. and Logtenberg, S. J. J. and Fricke, L. and van Zanen, A. L. and Slebe, J. J. P. and Kemlin, D. and van de Wetering, J. and Eiselt, J. and Kielberger, L. and El-Wakil, H. S. and ElHafeez, S. A. and Canal, C. and Facundo, C. and Ramos, A. M. and Debska-Slizien, A. and Veldhuizen, N. M. H. and Panagoutsos, S. and Matceac, I. and Nistor, I. and Cordos, M. and Groeneveld, J. H. M. and van Buren, M. and Diekmann, F. and Ferreira, A. C. and Santos, A. C. S. and Arias-Cabrales, C. and Llinas-Mallol, L. and Buxeda, A. and Tarrega, C. B. and Redondo-Pachon, D. and Jimenez, M. D. A. and Hofstra, J. M. and Franco, A. and Rodriguez-Ferrero, M. L. and Manzanos, S. B. and de Arriba, G. and Barrios, R. H. S. and Bartelet, K. and Demir, E. and Holl and er, D. A. M. J. and Kerckhoffs, A. and Buttner, S. and de Vries, A. P. J. and Meziyerh, S. and van der Helm, D. and Reinders, M. and Bouwsma, H. and Petruliene, K. and Maloney, S. and Verberk, I. and Di Luca, M. and Tuglular, S. Z. and Beerenhout, C. and Luik, P. T. and Kerschbaum, J. and Tiefenthaler, M. and Watschinger, B. and Adema, A. Y. and Stepanov, V. A. and Zulkarnaev, A. B. and Turkmen, K. and Decenzio, B. and Fliedner, A. and Miyasato, H. and Asberg, A. and Mjoen, G. and Pini, S. and de Biase, C. and van de Logt, A. E. and Maas, R. and Lebedeva, O. and Lopez, V. and Reichert, L. J. M. and Verhave, J. and Titov, D. and Parshina, E. V. and van Gils-Verrij, L. E. A. and de Bruin, C. J. R. and Harty, J. C. and Meurs, M. and Myslak, M. and Battaglia, Y. and Lentini, P. and den Deurwaarder, E. and Rahimzadeh, H. and Schouten, M. and Cabezas-Reina, C. J. and Diaz-Mareque, A. and Coca, A. and Meijers, B. K. I. and Naesens, M. and Kuypers, D. and Desschans, B. and Tonnerlier, A. and Wissing, K. M. and Dedinska, I. and Pessolano, G. and van der S and e, F. M. and Christiaans, M. H. L. and G and olfini, I. and Maggiore, U. and Kanaan, N. and Labriola, L. and Devresse, A. and Malik, S. and Berger, S. P. and Meijer, E. and S and ers, J. S. F. and Ponikvar, J. B. and Abrahams, A. C. and Molenaar, F. M. and van Zuilen, A. D. and Meijvis, S. C. A. and Dolmans, H. and Zanoli, L. and Marcantoni, C. and Esposito, P. and Krzesinski, J. M. and Barahira, J. D. and Gallieni, M. and Sabiu, G. and Martin-Moreno, P. L. and Guglielmetti, G. and Guzzo, G. and Luik, A. J. and van Kuijk, W. H. M. and Stikkelbroeck, L. W. H. and Hermans, M. M. H. and Rimsevicius, L. and Righetti, M. and Heitink-Ter Braak, N. | COVID-19-related mortality in kidney transplant and dialysis patients: Results of the ERACODA collaboration | 2020 | Insufficient data |
| Hoffmann, C. and Casado, J. L. and HÃ¤rter, G. and Vizcarra, P. and Moreno, A. and Cattaneo, D. and Meraviglia, P. and Spinner, C. D. and Schabaz, F. and Grunwald, S. and Gervasoni, C. | Immune deficiency is a risk factor for severe COVID-19 in people living with HIV | 2021 | Insufficient data |
| Ilonze, Onyedika J and Ballut, Kareem and Rao, Roopa S and Jones, Mark A and Guglin, Maya | SARS-CoV-2 infection in heart transplant recipients: a systematic literature review of clinical outcomes and immunosuppression strategies | 2021 | Systematic Review |
| Jager, K. J. and Kramer, A. and Chesnaye, N. C. and Couchoud, C. and Sanchez-Alvarez, J. E. and Garneata, L. and Collart, F. and Hemmelder, M. H. and Ambuhl, P. and Kerschbaum, J. and Legeai, C. and Del Pino, Y. Pino M. D. and Mircescu, G. and Mazzoleni, L. and Hoekstra, T. and Winzeler, R. and Mayer, G. and Stel, V. S. and Wanner, C. and Zoccali, C. and Massy, Z. A. | Results from the ERA-EDTA Registry indicate a high mortality due to COVID-19 in dialysis patients and kidney transplant recipients across Europe | 2020 | Paediatric |
| Jassat, W., Mudara, C., Ozougwu, L., Welch, R., Arendse, T., Masha, M., Blumberg, L., Kufa, T., Puren, A., Groome, M. and Govender, N. | Trends in COVID-19 Admissions and Deaths Among People Living with HIV in South Africa | 2023 | 2023 |
| Jering, K. S. and McGrath, M. M. and Mc Causl and , F. R. and Claggett, B. and Cunningham, J. W. and Solomon, S. D. | Excess mortality in solid organ transplant recipients hospitalized with COVID-19: A large-scale comparison of SOT recipients hospitalized with or without COVID-19 | 2022 | Insufficient data |
| Johnston, R. | The first 6 months of HIV-SARS-CoV-2 coinfection: outcomes for 6947 individuals | 2021 | Systematic Review |
| Jung, Y., Kwon, M. and Choi, H.G., 2021. Association between previous rheumatoid arthritis and COVID-19 and its severity: a nationwide cohort study in South Korea. | Association between previous rheumatoid arthritis and COVID-19 and its severity: a nationwide cohort study in South Korea | 2021 | Paediatric |
| Kamal, M. and Baudo, M. and Shmushkevich, S. and Geng, Y. and Rahouma, M. | Early mortality following COVID-19 infection among cancer patients who received radiotherapy: A meta-analysis | 2023 | 2023 |
| Kaplan-Lewis, E. and Banga, J. and Khan, M. and Casey, E. and Mazumdar, M. and Bratu, S. and Abdallah, M. and Pitts, R. and Leider, J. and Hennessey, K. and Cohen, G. M. and Clel and , C. M. and Salama, C. | HIV Diagnosis and the Clinical Course of COVID-19 Among Patients Seeking Care Within the New York City Public Hospital System During the Initial Pandemic Peak | 2021 | All-cause mortality |
| Kaplan-Lewis, E., Banga, J., Khan, M., Casey, E., Mazumdar, M., Bratu, S., Abdallah, M., Pitts, R., Leider, J., Hennessey, K. and Cohen, G.M. | .HIV Diagnosis and the Clinical Course of COVID-19 Among Patients Seeking Care Within the New York City Public Hospital System During the Initial Pandemic Peak.Â | 2021 | Duplication |
| Kates, Olivia S and Haydel, Br and y M and Florman, S and er S and Rana, Meenakshi M and Chaudhry, Zohra S and Ramesh, Mayur S and Safa, Kassem and Kotton, Camille Nelson and Blumberg, Emily A and Besharatian, Behdad D | Coronavirus disease 2019 in solid organ transplant: a multicenter cohort study | 2021 | Insufficient data |
| Khusid, J.A., Becerra, A.Z., Gallante, B., Sadiq, A.S., Atallah, W.M., Badani, K.K. and Gupta, M. | Cancer, mortality, and acute kidney injury among hospitalized patients with SARS-CoV-2 infection | 2021 | All-cause mortality |
| Kjeldsen, S., Nielsen, J., Mertz Nørgård, B. and Kjeldsen, J., | Mesalazine in inflammatory bowel disease and COVID-19: hospitalization and adverse in-hospital outcomes based on nationwide data | 2022 | Insufficient data |
| Klebanov, N., Pahalyants, V., Murphy, W.S., Theodosakis, N., Zubiri, L., Klevens, R.M., Kwatra, S.G., Lilly, E., Reynolds, K.L. and Semenov, Y.R. | Risk of COVID-19 in patients with cancer receiving immune checkpoint inhibitors | 2021 | Insufficient data |
| Koren, J. and Steinerova, K. and Janikova, A. and Belada, D. and Hajkova, B. and Krcmeryova, M. and Hanackova, V. and Vackova, B. and Jindra, P. and Osovska, M. and Svobodova, E. and Dlouha, L. and Vodicka, P. and Trneny, M. | Multicenter retrospective analysis of risk factors for mortality of COVID-19 infection in patients with lymphoma | 2021 | Insufficient data |
| Kowalska, J. D. and Lara, M. and Hlebowicz, M. and Mularska, E. and JabÅ‚onowska, E. and Siwak, E. and W and aÅ‚owicz, A. and Witak-JÄ™dra, M. and Olczak, A. and BociÄ…ga-Jasik, M. and Suchacz, M. and Stempkowska-Rejek, J. and Wasilewski, P. and Parczewski, M. | Non-HIV-related comorbidities and uncontrolled HIV replication are independent factors increasing the odds of hospitalization due to COVID-19 among HIV-positive patients in Poland | 2023 | 2023 |
| Kremer, Daan and Pieters, Tobias T and Verhaar, Marianne C and Berger, Stefan P and Bakker, Stephan JL and van Zuilen, Arjan D and Joles, Jaap A and Vernooij, Robin WM and van Balkom, Bas WM | A systematic review and metaâ€analysis of COVID-19 in kidney transplant recipients: lessons to be learned | 2021 | Systematic Review |
| Kridin, K. and Schonmann, Y. and Damiani, G. and Peretz, A. and Onn, E. and Bitan, D. T. and Cohen, A. D. | Tumor necrosis factor inhibitors are associated with a decreased risk of COVID-19-associated hospitalization in patients with psoriasis-A population-based cohort study | 2021 | Insufficient data |
| Kridin, K. and Schonmann, Y. and Solomon, A. and Damiani, G. and Tzur Bitan, D. and Onn, E. and Weinstein, O. and Cohen, A. D. | Risk of COVID-19 infection, hospitalization, and mortality in patients with psoriasis treated by interleukin-17 inhibitors | 2022 | Insufficient data |
| Kridin, K. and Schonmann, Y. and Tzur Bitan, D. and Damiani, G. and Peretz, A. and Weinstein, O. and Cohen, A. D. | Coronavirus Disease 2019 (COVID-19)-Associated Hospitalization and Mortality in Patients with Psoriasis: A Population-Based Study | 2021 | Insufficient data |
| Kridin, K., Schonmann, Y., Solomon, A., Damiani, G., Tzur Bitan, D., Onn, E., Weinstein, O. and Cohen, A.D. | Risk of covid-19 infection, hospitalization, and mortality in patients with psoriasis treated by interleukin-17 inhibitors. | 2022 | Insufficient data |
| Lamure, S. and DulÃ©ry, R. and Di Blasi, R. and Chauchet, A. and Laureana, C. and Deau-Fischer, B. and Drenou, B. and Soussain, C. and Rossi, C. and NoÃ«l, N. and Choquet, S. and Bologna, S. and Joly, B. and Kohn, M. and Malak, S. and Fouquet, G. and Daguindau, E. and Bernard, S. and ThiÃ©blemont, C. and Cartron, G. and Lacombe, K. and Besson, C. | Determinants of outcome in Covid-19 hospitalized patients with lymphoma: A retrospective multicentric cohort study | 2020 | Insufficient data |
| Lee, K. W. and Yap, S. F. and Ngeow, Y. F. and Lye, M. S. | COVID-19 in People Living with HIV: A Systematic Review and Meta-Analysis | 2021 | Systematic Review |
| Lee, L. Y. W. and Cazier, J. B. and Angelis, V. and Arnold, R. and Bisht, V. and Campton, N. A. and Chackathayil, J. and Cheng, V. W. T. and Curley, H. M. and Fittall, M. W. and Freeman-Mills, L. and Gennatas, S. and Goel, A. and Hartley, S. and Hughes, D. J. and Kerr, D. and Lee, A. J. X. and Lee, R. J. and McGrath, S. E. and Middleton, C. P. and Murugaesu, N. and Newsom-Davis, T. and Okines, A. F. C. and Olsson-Brown, A. C. and Palles, C. and Pan, Y. and Pettengell, R. and Powles, T. and Protheroe, E. A. and Purshouse, K. and Sharma-Oates, A. and Sivakumar, S. and Smith, A. J. and Starkey, T. and Turnbull, C. D. and Varnai, C. and Yousaf, N. and Kerr, R. and Middleton, G. | COVID-19 mortality in patients with cancer on chemotherapy or other anticancer treatments: A prospective cohort study | 2020 | Insufficient data |
| Lee, M.J., Snell, L.B., Douthwaite, S.T., Fidler, S., Fitzgerald, N., Goodwin, L., Hamzah, L., Kulasegaram, R., Lawrence, S., Lwanga, J. and Marchant, R. | Clinical outcomes of patients with and without HIV hospitalized with COVID-19 in EnglandÂ | 2022 | Duplication |
| Lee, M.J., Snell, L.B., Douthwaite, S.T., Fidler, S., Fitzgerald, N., Goodwin, L., Hamzah, L., Kulasegaram, R., Lawrence, S., Lwanga, J. and Marchant, R. | Clinical outcomes of patients with and without HIV hospitalized with COVID-19 in England during the early stages of the pandemic: a matched retrospective multi-centre analysis (RECEDE-C19 study) | 2022 | Duplication |
| Legeai, C. and Jasseron, C. and Cantrelle, C. and Varnous, S. and Para, M. and Epailly, E. and Guendouz, S. and Sebbag, L. and Guillemain, R. and Vermes, E. and Kerbaul, F. and Dorent, R. | Death Rate in Heart Transplant Recipients during the COVID-19 Outbreak in France | 2021 | Insufficient data |
| Lerner, A. H. and Arvanitis, P. and Vieira, K. and Klein, E. J. and Farmakiotis, D. | mRNA Vaccination Decreases COVID-19-Associated Morbidity and Mortality Among Organ Transplant Recipients: A Contemporary Cohort Study | 2022 | Insufficient data |
| Lerner, A. H. and Klein, E. and Farmakiotis, D. | Mortality in organ transplant recipients with COVID-19 compared to non-transplant or waitlisted patients: A meta-analysis | 2021 | Insufficient data |
| Leuva, H., Zhou, M., Brau, N., Brown, S.T., Mundi, P., Rosenberg, T.C.M., Luhrs, C., Bates, S.E., Park, Y.H.A. and Fojo, T., | Influence of cancer on COVID-19 incidence, outcomes, and vaccine effectiveness: A Prospective Cohort Study of US Veterans | 2022 | All-cause mortality |
| Li, H. and Wallace, Z. S. and Sparks, J. A. and Lu, N. and Wei, J. and Xie, D. and Wang, Y. and Zeng, C. and Lei, G. and Zhang, Y. | Risk of COVID-19 Among Unvaccinated and Vaccinated Patients With Rheumatoid Arthritis: A General Population Study | 2023 | 2023 |
| Li, J., Tian, A., Yang, D., Zhang, M., Chen, L., Wen, J. and Chen, P. | Celiac disease and the susceptibility of COVID-19 and the risk of severe COVID-19: a Mendelian randomization study | 2022 | Insufficient data |
| Liang, M. and Luo, N. and Chen, M. and Chen, C. and Singh, S. and Singh, S. and Tan, S. | Prevalence and Mortality due to COVID-19 in HIV Co-Infected Population: A Systematic Review and Meta-Analysis | 2021 | Systematic Review |
| Liang, W., Guan, W., Chen, R., Wang, W., Li, J., Xu, K., Li, C., Ai, Q., Lu, W., Liang, H. and Li, S | Cancer patients in SARS-CoV-2 infection: a nationwide analysis in China. | 2020 | Insufficient data |
| Liu, M. and Wang, H. and Liu, L. and Cui, S. and Huo, X. and Xiao, Z. and Zhao, Y. and Wang, B. and Zhang, G. and Wang, N. | Risk of COVID-19 infection, hospitalization and mortality in psoriasis patients treated with interleukin-17 inhibitors: A systematic review and meta-analysis | 2022 | Systematic Review |
| Lopez, V. and Vazquez, T. and Casas, C. and Hibrovo, R. and Hern and ez, D. | Risk factors for mortality in kidney transplant patients infected by sars-cov-2 in south of Spain | 2021 | Insufficient data |
| Lova Sun | Rates of COVID-19-related Outcomes in Cancer compared to non-Cancer Patients | 2020 | Duplication |
| Ludvigsson, J.F., Axelrad, J., Halfvarson, J., Khalili, H., Larsson, E., Lochhead, P., Roelstraete, B., Simon, T.G., Söderling, J. and Olen, O. | Inflammatory bowel disease and risk of severe COVID-19: a nationwide population-based cohort study in Sweden | 2021 | Paediatric |
| Lukin, D.J., Kumar, A., Hajifathalian, K., Sharaiha, R.Z., Scherl, E.J., Longman, R.S., Funez-dePagnier, G., Duenas-Bianchi, L., Jacob, V., Battat, R.J. and Krisko, T. | Baseline disease activity and steroid therapy stratify risk of COVID-19 in patients with inflammatory bowel disease. | 2020 | Insufficient data |
| Macaluso, F. S. and Giuliano, A. and Fries, W. and Viola, A. and Abbruzzese, A. and Cappello, M. and Giuffrida, E. and Carrozza, L. and Privitera, A. C. and Magnano, A. and Ferracane, C. and Scalisi, G. and Minissale, M. G. and Giangreco, E. and Garufi, S. and Bertolami, C. and Cucinotta, U. and Graziano, F. and CasÃ , A. and Renna, S. and Teresi, G. and Rizzuto, G. and Mannino, M. and Maida, M. and Orl and o, A. | Severe Activity of Inflammatory Bowel Disease is a Risk Factor for Severe COVID-19 | 2023 | 2023 |
| Mageau, A., Papo, T., Ruckly, S., Strukov, A., van Gysel, D., Sacre, K. and Timsit, J.F | Survival after COVID-19-associated organ failure among inpatients with systemic lupus erythematosus in France: a nationwide study | 2022 | Insufficient data |
| Mapahla, Lovemore and Abdelmaksoud, Asmaa and Arif, Rida and Islam, Nazmul and Chinhenzva, Albert and Doi, Suhail AR and Chivese, Taw and a | The risk of severe COVID-19 and mortality from COVID-19 in people living with HIV compared to individuals without HIV-a systematic review and meta-analysis of 1 268 676 individuals | 2021 | Systematic Review |
| Marinaki, Smaragdi and Tsiakas, Stathis and Korogiannou, Maria and Grigorakos, Konstantinos and Papalois, Vassilios and Boletis, Ioannis | A systematic review of COVID-19 infection in kidney transplant recipients: a universal effort to preserve patientsâ€™ lives and allografts | 2020 | Systematic Review |
| Martinez-Lopez, J. and Mateos, M. V. and Lopez-Munoz, N. and Magan, M. A. S. and De Miguel, M. D. and De la Guia, A. L. and Sureda, A. and Encinas, C. and Royo, D. C. and Rosinol, L. and Tomas, L. G. and Hern and ez-Rivas, J. A. and Rojas, J. A. and Alegre, A. and Blanchard, M. J. and Barrigon, F. E. and Garcia, E. G. and Inigo, B. and Barahona, P. B. and Penalver, F. J. and De La Rubia, J. and Fern and ez-Escalada, N. and Alonso, J. M. A. and Blade, J. and Lahuerta, J. J. and De La Cruz, J. and San-Miguel, J. F. | Severity of COVID-19 clinical outcomes and mortality in multiple myeloma patients over year 1 of the pandemic | 2021 | Insufficient data |
| Martínez-López, J., Mateos, M.V., Encinas, C., Sureda, A., Hernández-Rivas, J.Á., Lopez de la Guia, A., Conde, D., Krsnik, I., Prieto, E., Riaza Grau, R. and Gironella, M. | Multiple myeloma and SARS-CoV-2 infection: clinical characteristics and prognostic factors of inpatient mortality | 2020 | Single Centre |
| Masclioni, M. | COVID-19 rate no higher with HIV in largest US HIV+/HIV- cohort. | 2020 | Duplication |
| McKeigue, P.M., Porter, D., Hollick, R.J., Ralston, S.H., McAllister, D.A. and Colhoun, H.M. | Risk of severe COVID-19 in patients with inflammatory rheumatic diseases treated with immunosuppressive therapy in Scotland | 2023 | 2023 |
| Medina-Pestana, J., Cristelli, M.P., Foresto, R.D., Tedesco-Silva, H. and Requião-Moura, L.R | The higher COVID-19 fatality rate among kidney transplant recipients calls for further action | 2022 | Single Centre |
| Mellor, M. M. and Bast, A. C. and Jones, N. R. and Roberts, N. W. and OrdÃ³Ã±ez-Mena, J. M. and Reith, A. J. M. and Butler, C. C. and Matthews, P. C. and Dorward, J. | Risk of adverse coronavirus disease 2019 outcomes for people living with HIV | 2021 | Systematic Review |
| Meng, Y., Lu, W., Guo, E., Liu, J., Yang, B., Wu, P., Lin, S., Peng, T., Fu, Y., Li, F. and Wang, Z | Cancer history is an independent risk factor for mortality in hospitalized COVID-19 patients: a propensity scoreâ€matched analysi | 2020 | Single Centre |
| Modelli de Andrade, L. G. and de S and es-Freitas, T. V. and Requiao-Moura, L. R. and Viana, L. A. and Cristelli, M. P. and Garcia, V. D. and Alcantara, A. L. C. and Esmeraldo, R. M. and Abbud Filho, M. and Pacheco-Silva, A. and de Lima Carneiro, E. C. R. and Manfro, R. C. and Costa, Kmah and Simao, D. R. and de Sousa, M. V. and Santana, Vbbm and Noronha, I. L. and Romao, E. A. and Zanocco, J. A. and Arimatea, G. G. Q. and De Boni Monteiro de Carvalho, D. and Tedesco-Silva, H. and Medina-Pestana, J. and Brazil, Covid-Kt | Development and validation of a simple web-based tool for early prediction of COVID-19-associated death in kidney transplant recipients | 2022 | Insufficient data |
| Mollaeian, A., Kim, D.S. and Haas, C.J | COVID-19 Prevalence and Outcomes among Individuals with Rheumatoid Arthritis and Systemic Lupus ErythematosusÂ | 2021 | Insufficient data |
| Möller, I.K., Gisslén, M., Wagner, P., Sparén, P. and Carlander, C. | COVID-19 hospitalization outcomes in adults by HIV status; a nationâ€wide registerâ€based study | 2023 | 2023 |
| Molnar, M.Z., Bhalla, A., Azhar, A., Tsujita, M., Talwar, M., Balaraman, V., Sodhi, A., Kadaria, D., Eason, J.D., Hayek, S.S. and Coca, S.G | Outcomes of critically ill solid organ transplant patients with COVID-19 in the United States. | 2020 | Duplication |
| Moradi, Y. and Soheili, M. and Dehghanbanadaki, H. and Moradi, G. and Moradpour, F. and Mahdavi Mortazavi, S. M. and Gilzad Kohan, H. and Zareie, M. | The Effect of HIV/AIDS Infection on the Clinical Outcomes of COVID-19: A Meta-Analysis | 2022 | Systematic Review |
| Muthuka, J. K. and Francis, M. W. and Oluoch, K. J. and Nzioki, J. M. | Effect of HIV disease and the associated moderators on COVID-19 Mortality | 2022 | Systematic Review |
| Okumura, K. and Dh and , A. and Misawa, R. and Sogawa, H. and Bodin, R. and Wolf, D. C. and Nishida, S. | Impact of COVID-19 on Increased Mortality in Liver Transplant Recipients in United States | 2022 | Systematic Review |
| Overvad, M., Koch, A., Jespersen, B., Gustafsson, F., Krause, T.G., Hansen, C.H., Ethelberg, S. and Obel, N | Outcomes following SARS-CoV-2 infection in individuals with and without solid organ transplantation—A Danish nationwide cohort study | 2022 | Insufficient data |
| Oyelade, T. and Alqahtani, J. S. and Hjazi, A. M. and Li, A. and Kamila, A. and Raya, R. P. | Global and Regional Prevalence and Outcomes of COVID-19 in People Living with HIV: A Systematic Review and Meta-Analysis | 2022 | Insufficient data |
| Ozturk, S., Turgutalp, K., Arici, M., Odabas, A.R., Altiparmak, M.R., Aydin, Z., Cebeci, E., Basturk, T., Soypacaci, Z., Sahin, G. and Elif Ozler, T. | Mortality analysis of COVID-19 infection in chronic kidney disease, haemodialysis and renal transplant patients compared with patients without kidney disease: a nationwide analysis from Turkey. | 2020 | Duplication |
| Pahalyants, V. and Murphy, W. S. and Klebanov, N. and Theodosakis, N. and Klevens, R. M. and Lilly, E. and Asgari, M. and Semenov, Y. R. | 28552 Risk of COVID-19 and subsequent mortality among patients receiving immunosuppressive biologic therapy: A retrospective matched cohort study | 2021 | Systematic Review |
| Peach, E., Rutter, M., Lanyon, P., Grainge, M.J., Hubbard, R., Aston, J., Bythell, M., Stevens, S. and Pearce, F. | Risk of death among people with rare autoimmune diseases compared with the general population in England during the 2020 COVID-19 pandemic | 2021 | All-cause mortality |
| Penso, L. and Dray-Spira, R. and Weill, A. and Zureik, M. and Sbidian, E. | Psoriasis-related treatment exposure and hospitalization or in-hospital mortality due to COVID-19 during the first and second wave of the pandemic: cohort study of 1 326 312 patients in France* | 2022 | All-cause mortality |
| RÃ¼thrich, Maria Madeleine and Giessen-Jung, C and Borgmann, S and Classen, AY and Dolff, S and GrÃ¼ner, B and Hanses, F and Isberner, N and KÃ¶hler, P and Lanznaster, J | COVID-19 in cancer patients: clinical characteristics and outcomeâ€”an analysis of the LEOSS registry | 2021 | Paediatric |
| Raiker, R., Pakhchanian, H., DeYoung, C., Gupta, L., Kardeş, S., Ahmed, S. and Kavadichanda, C. | Short term outcomes of COVID-19 in lupus: Propensity score matched analysis from a nationwide multi-centric research network | 2021 | All-cause mortality |
| Raiker, R., Pakhchanian, H., DeYoung, C., Gupta, L., Kardeş, S., Ahmed, S. and Kavadichanda, C., | Short term outcomes of COVID-19 in lupus: propensity score matched analysis from a nationwide multi-centric research network. | 2021 | All-cause mortality |
| Raiker, R., Pakhchanian, H., Hussain, A. and Deng, M. | Outcomes of COVID‐19 in patients with skin cancer | 2021 | All-cause mortality |
| Raja, Mohammed A and Mendoza, Maria A and Villavicencio, Aasith and Anjan, Shweta and Reynolds, John M and Kittipibul, Veraprapas and Fern and ez, Anmary and Guerra, Giselle and Camargo, Jose F and Simkins, Jacques | COVID-19 in solid organ transplant recipients: a systematic review and meta-analysis of current literature | 2021 | Systematic Review |
| Ravanan, R. and Callaghan, C. J. and Mumford, L. and Ushiro-Lumb, I. and Thorburn, D. and Casey, J. and Friend, P. and Parameshwar, J. and Currie, I. and Burnapp, L. and Baker, R. and Dudley, J. and Oniscu, G. C. and Berman, M. and Asher, J. and Harvey, D. and Manara, A. and Manas, D. and Gardiner, D. and Forsythe, J. L. R. | SARS-CoV-2 infection and early mortality of waitlisted and solid organ transplant recipients in England: A national cohort study | 2020 | Insufficient data |
| Regalado-Artamendi, I. and Jimenez-Ubieto, A. and Hern and ez-Rivas, J. A. and Navarro, B. and Nunez, L. and Alaez, C. and Cordoba, R. and Penalver, F. J. and Cannata, J. and Estival, P. and Quiroz-Cervantes, K. and Riaza Grau, R. and Velasco, A. and Martos, R. and Domingo-Gonzalez, A. and Benito-Parra, L. and Gomez-Sanz, E. and Lopez-Jimenez, J. and Matilla, A. and Herraez, M. R. and Penalva, M. J. and Garcia-Suarez, J. and Diez-Martin, J. L. and Bastos-Oreiro, M. | Risk Factors and Mortality of COVID-19 in Patients With Lymphoma: A Multicenter Study | 2021 | Insufficient data |
| Requiao-Moura, L. R. and Modelli de Andrade, L. G. and de S and es-Freitas, T. V. and Cristelli, M. P. and Viana, L. A. and Nakamura, M. R. and Garcia, V. D. and Manfro, R. C. and Simao, D. R. and Almeida, Ramb and Ferreira, G. F. and Costa, Kmah and de Lima, P. R. and Pacheco-Silva, A. and Charpiot, Immf and Deboni, L. M. and Ferreira, T. C. A. and Mazzali, M. and Calazans, C. A. C. and Oria, R. B. and Tedesco-Silva, H. and Medina-Pestana, J. and Group*, Covid-Kt Brazil Study | The Mycophenolate-based Immunosuppressive Regimen Is Associated With Increased Mortality in Kidney Transplant Patients With COVID-19 | 2022 | Insufficient data |
| Rocha, S. Q. and Avelino-Silva, V. I. and Tancredi, M. V. and Jamal, L. F. and Ferreira, P. R. A. and Tayra, A. and Ferreira, P. M. and Carvalhanas, T. and Domingues, C. S. B. and Souza, R. A. and Gianna, M. C. and Kalichman, A. O. and Leite, O. H. M. and Souza, T. N. L. and Gomes, E. Costa D. A. and Furtado, J. J. D. and Costa, A. F. | COVID-19 and HIV/AIDS in a cohort study in Sao Paulo, Brazil: outcomes and disparities by race and schooling | 2022 | Insufficient data |
| Russell, Beth and Moss, Charlotte L and Shah, Vallari and Ko, Thinzar Ko and Palmer, Kieran and Sylva, Rushan and George, Gincy and Monroy-Iglesias, Maria J and Patten, Piers and Ceesay, Muhammed Mansour | Risk of COVID-19 death in cancer patients: an analysis from Guyâ€™s Cancer Centre and Kingâ€™s College Hospital in London | 2021 | Insufficient data |
| Rutter, M., Lanyon, P.C., Grainge, M.J., Hubbard, R., Bythell, M., Stilwell, P., Aston, J., McPhail, S., Stevens, S. and Pearce, F.A. | COVID-19 infection, admission and death and the impact of corticosteroids amongst people with rare autoimmune rheumatic disease during the second wave | 2023 | 2023 |
| SÃ¸ftel and , John M and Friman, Gustav and von Zurâ€MÃ¼hlen, Bengt and Ericzon, Boâ€GÃ¶ran and Wallquist, Carin and Karason, Kristjan and Friman, V and a and Ekelund, Jan and Felldin, Marie and Magnusson, Jesper | COVID-19 in solid organ transplant recipients: a national cohort study from Sweden | 2021 | Insufficient data |
| Sachdev, D., Mara, E., Hsu, L., Scheer, S., Rutherford, G., Enanoria, W. and Gandhi, M. | COVID-19 susceptibility and outcomes among people living with HIV in San Francisco | 2021 | Insufficient data |
| Sahota, A., Tien, A., Yao, J., Dong, E., Herald, J., Javaherifar, S., Neyer, J., Hwang, J., Lee, R. and Fong, T.L. | Incidence, risk factors, and outcomes of COVID-19 infection in a large cohort of solid organ transplant recipients | 2022 | Duplication |
| Saini, Kamal S and Tagliamento, Marco and Lambertini, Matteo and McNally, Richard and Romano, Marco and Leone, Manuela and Curigliano, Giuseppe and de Azambuja, Ev and ro | Mortality in patients with cancer and coronavirus disease 2019: a systematic review and pooled analysis of 52 studies | 2020 | Systematic Review |
| Sales, T.L.S., Souza-Silva, M.V.R., Delfino-Pereira, P., Neves, J.V.B., Sacioto, M.F., de Assis, V.C.M., Duani, H., de Oliveira, N.R., Sampaio, N.D.C.S., Ramos, L.E.F. and Schwarzbold, A.V | COVID-19 outcomes in people living with HIV: peering through the waves | 2023 | 2023 |
| Salto-Alej and re, Sonsoles and Jimenez-Jorge, Silvia and SabÃ©, Nuria and Ramos-MartÃ­nez, Antonio and Linares, Laura and Valerio, Maricela and Martin-Davila, Pilar and FernÃ¡ndez-Ruiz, Mario and FariÃ±as, MarÃ­a Carmen and Blanes-Julia, Marino | Risk factors for unfavorable outcome and impact of early post-transplant infection in solid organ recipients with COVID-19: A prospective multicenter cohort study | 2021 | Insufficient data |
| Sánchez‐Velázquez A, Bauer‐Alonso A, Estrach T, Vega‐Díez D, Garcia‐Muret P, Haya L, Peñate Y, Acebo E, Fernández de Misa R, Blanes M, Suh‐Oh HJ | Â Patients with primary cutaneous lymphoma are at risk for severe COVID-19. Data from the Spanish primary cutaneous lymphoma registry. | 2021 | Insufficient data |
| Schaffrath, J. and Brummer, C. and Wolff, D. and Holtick, U. and Kroger, N. and Bornhauser, M. and Kraus, S. and Hilgendorf, I. and Blau, I. W. and Penack, O. and Wittke, C. and Steiner, N. and Nachbaur, D. and Thurner, L. and Hindahl, H. and Zeiser, R. and Maier, C. P. and Bethge, W. and Muller, L. P. | High Mortality of COVID-19 Early after Allogeneic Stem Cell Transplantation: A Retrospective Multicenter Analysis on Behalf of the German Cooperative Transplant Study Group | 2022 | Insufficient data |
| Serraino, D., Zucchetto, A., Dal Maso, L., Del Zotto, S., Taboga, F., Clagnan, E., Fratino, L., Tosolini, F. and Burba, I. | Prevalence , determinants, and outcomes of SARS‐COV‐2 infection among cancer patients. A population‐based study in northern Italy | 2021 | All-cause mortality |
| Shapiro, A. E. and Bender Ignacio, R. A. and Whitney, B. M. and Delaney, J. A. and Nance, R. M. and Bamford, L. and Wooten, D. and Keruly, J. C. and Burkholder, G. and Napravnik, S. and Mayer, K. H. and Webel, A. R. and Kim, H. N. and Van Rompaey, S. E. and Christopoulos, K. and Jacobson, J. and Karris, M. and Smith, D. and Johnson, M. O. and Willig, A. and Eron, J. J. and Hunt, P. and Moore, R. D. and Saag, M. S. and Mathews, W. C. and Crane, H. M. and Cachay, E. R. and Kitahata, M. M. | Factors Associated With Severity of COVID-19 Disease in a Multicenter Cohort of People With HIV in the United States, March-December 2020 | 2022 | Insufficient data |
| Sharafeldin, Noha and Bates, Benjamin and Song, Qianqian and Madhira, Vithal and Yan, Yao and Dong, Sharlene and Lee, Eileen and Kuhrt, Nathaniel and Shao, Yu Raymond and Liu, Feifan | Outcomes of COVID-19 in patients with cancer: report from the National COVID Cohort Collaborative (N3C) | 2021 | Insufficient data |
| Shaw, B. and Shortt, J. and Low, M. and Rogers, B. and Kaplan, Z. and Fedele, P. and Gregory, G. and Vilcassim, S. and Gilbertson, M. and Grigoriadis, G. and Opat, S. | Low mortality in vaccinated immunocompromised haematology patients infected with SARS-CoV-2 | 2022 | Insufficient data |
| Shields, A. M. and Tadros, S. and Al-Hakim, A. and Nell, J. M. and Lin, M. M. N. and Chan, M. and Goddard, S. and Dempster, J. and Dziadzio, M. and Patel, S. Y. and Elkalifa, S. and Huissoon, A. and Duncan, C. J. A. and Herwadkar, A. and Khan, S. and Bethune, C. and Elcombe, S. and Thaventhiran, J. and Klenerman, P. and Lowe, D. M. and Savic, S. and Burns, S. O. and Richter, A. G. | Impact of vaccination on hospitalization and mortality from COVID-19 in patients with primary and secondary immunodeficiency: The United Kingdom experience | 2022 | Insufficient data |
| Singh, A. K. and Jena, A. and Kumar, M. P. and Sharma, V. and Sebastian, S. | Risk and outcomes of coronavirus disease in patients with inflammatory bowel disease: A systematic review and meta-analysis | 2021 | Systematic Review |
| Singh, S., Khan, A., Chowdhry, M., Bilal, M., Kochhar, G.S. and Clarke, K. | Â Risk of severe coronavirus disease 2019 in patients with inflammatory bowel disease in the United States: a Multicenter Research Network Study.Â | 2020 | Compound outcome |
| Sonaglia, A., Comoretto, R., Pasut, E., Treppo, E., Del Frate, G., Colatutto, D., Zabotti, A., De Vita, S. and Quartuccio, L. | Safety of Biologic-DMARDs in Rheumatic Musculoskeletal Disorders: A Population-Based Study over the First Two Waves of COVID-19 Outbreak | 2022 | Insufficient data |
| Sparks, J. A. and Wallace, Z. S. and Seet, A. M. and Gianfrancesco, M. A. and Izadi, Z. and Hyrich, K. L. and Strangfeld, A. and Gossec, L. and Carmona, L. and Mateus, E. F. and Lawson-Tovey, S. and Trupin, L. and Rush, S. and Katz, P. and Schmajuk, G. and Jacobsohn, L. and Wise, L. and Gilbert, E. L. and Duarte-GarcÃ­a, A. and Valenzuela-Almada, M. O. and Pons-Estel, G. J. and Isnardi, C. A. and Berbotto, G. A. and Hsu, T. Y. and D'Silva, K. M. and Patel, N. J. and Kearsley-Fleet, L. and SchÃ¤fer, M. and Ribeiro, S. L. E. and Al Emadi, S. and Tidblad, L. and ScirÃ¨, C. A. and Raffeiner, B. and Thomas, T. and Flipo, R. M. and Avouac, J. and Seror, R. and Bernardes, M. and Cunha, M. M. and Hasseli, R. and Schulze-Koops, H. and MÃ¼ller-Ladner, U. and Specker, C. and Souza, V. A. and Mota, Lmhd and Gomides, A. P. M. and DieudÃ©, P. and Nikiphorou, E. and Kronzer, V. L. and Singh, N. and Ugarte-Gil, M. F. and Wallace, B. and Akpabio, A. and Thomas, R. and Bhana, S. and Costello, W. and Grainger, R. and Hausmann, J. S. and Liew, J. W. and Sirotich, E. and Sufka, P. and Robinson, P. C. and Machado, P. M. and Yazdany, J. | Associations of baseline use of biologic or targeted synthetic DMARDs with COVID-19 severity in rheumatoid arthritis: Results from the COVID-19 Global Rheumatology Alliance physician registry | 2021 | Insufficient data |
| Spila Alegiani, S., Crisafulli, S., Giorgi Rossi, P., Mancuso, P., Salvarani, C., Atzeni, F., Gini, R., Kirchmayer, U., Belleudi, V., Kurotschka, P.K. and Leoni, O | Risk of coronavirus disease 2019 hospitalization and mortality in rheumatic patients treated with hydroxychloroquine or other conventional disease-modifyingÂ | 2021 | Insufficient data |
| Ssentongo, P. and Heilbrunn, E. S. and Ssentongo, A. E. and Advani, S. and Chinchilli, V. M. and Nunez, J. J. and Du, P. | Epidemiology and outcomes of COVID-19 in HIV-infected individuals: a systematic review and meta-analysis | 2021 | Systematic Review |
| Ssentongo, P. and Ssentongo, A. E. and Heilbrunn, E. S. and Du, P. | Prevalence of HIV in patients hospitalized for COVID-19 and associated mortality outcomes: A systematic review and meta-analysis | 2020 | Systematic Review |
| Steffanoni, S. and Calimeri, T. and Laurenge, A. and Fox, C. P. and Soussain, C. and Grommes, C. and Tisi, M. C. and Boot, J. and Crosbie, N. and Visco, C. and Arcaini, L. and Chaganti, S. and Sassone, M. C. and Alencar, A. and Armiento, D. and Romano, I. and Dietrich, J. and Itchaki, G. and Bruna, R. and Fracchiolla, N. S. and Arletti, L. and Venditti, A. and Booth, S. and Musto, P. and Hoang Xuan, K. and Batchelor, T. T. and Cwynarski, K. and Ferreri, A. J. M. | Impact of severe acute respiratory syndrome coronavirus-2 infection on the outcome of primary central nervous system lymphoma treatment: A study of the International PCNSL Collaborative Group | 2022 | Systematic Review |
| Sultan, K. and Durbin, L. and Bhardwaj, R. and Mackey, J. and Becher, N. and Abureesh, M. and Lakhani, K. and Mone, A. and Abergel, J. and Trindade, A. and Korelitz, B. I. and Swaminath, A. | Corticosteroid and Biologic Use Not Associated With Adverse Outcomes for Inflammatory Bowel Disease Patients Hospitalized With COVID-19 | 2021 | Insufficient data |
| Sun, J., Patel, R., Madhira, V., Olex, A.L., French, E., Islam, J.Y., Moffitt, R., Franceschini, N., Mannon, R.B. and Kirk, G.D | COVID-19 HOSPITALIZATION AMONG PEOPLE WITH HIV OR SOLID ORGAN TRANSPLANT IN THE US. | 2021 | Duplication |
| Sun, Y., Miller, D.C., Akpandak, I., Chen, E.M., Arnold, B.F. and Acharya, N.R | Association between immunosuppressive drugs and coronavirus disease 2019 outcomes in patients with noninfectious uveitis in a large US claims database | 2022 | Insufficient data |
| Swan, J. T. and Rizk, E. and Jones, S. L. and Nwana, N. and Nicolas, J. C. and Tran, A. and Nisar, T. and Menser, T. and Yi, S. G. and Moore, L. W. and Gaber, A. O. and Knight, R. J. | Propensity matched analysis of death and non-favorable discharge among hospitalized transplant recipients with covid-19 | 2021 | Insufficient data |
| Taxonera, C., Sagastagoitia, I., Alba, C., Manas, N., Olivares, D. and Rey, E | 2019 novel coronavirus disease [COVID-19] in patients with inflammatory bowel diseases. | 2020 | Insufficient data |
| Tesoriero, J.M., Swain, C.A.E., Pierce, J.L., Zamboni, L., Wu, M., Holtgrave, D.R., Gonzalez, C.J., Udo, T., Morne, J.E., Hart-Malloy, R. and Rajulu, D.T. | COVID-19 outcomes among persons living with or without diagnosed HIV infection in New York state | 2021 | Duplication |
| Tilch, M. K. and Visco, C. and Kinda, S. and Hermine, O. and Kohn, M. and Besson, C. and Lamure, S. and DulÃ©ry, R. and Ragaini, S. and Eyre, T. A. and Van Meerten, T. and Ohler, A. and Eckerle, S. and Dreyling, M. and Hess, G. and GinÃ©, E. and Gomes da Silva, M. | Outcome of COVID-19 in Patients With Mantle Cell Lymphoma-Report From the European MCL Registry | 2022 | Insufficient data |
| Trapani, S., Masiero, L., Puoti, F., Rota, M.C., Del Manso, M., Lombardini, L., Riccardo, F., Amoroso, A., Pezzotti, P., Grossi, P.A. and Brusaferro, S | Incidence and outcome of SARS-CoV-2 infection on solid organ transplantation recipients: a nationwide population-based study | 2021 | Paediatric |
| Tripathi, K. and Godoy Brewer, G. and Thu Nguyen, M. and Singh, Y. and Saleh Ismail, M. and Sauk, J. S. and Parian, A. M. and Limketkai, B. N. | COVID-19 and Outcomes in Patients With Inflammatory Bowel Disease: Systematic Review and Meta-Analysis | 2022 | Systematic Review |
| Turk, M. and Alhuneafat, L. and Rizvi, A. W. and Gupta, N. and Barnett, K. and Gutta, R. and Mealy, S. and Rana, T. and Shah, A. and Sharma, A. and Osman, O. and Sureshkumar, K. K. | COVID-19 Mortality in Kidney Transplant Recipients: Analysis of Risk Factors | 2022 | Insufficient data |
| Ugarte-Gil, M. F. and AlarcÃ³n, G. S. and Izadi, Z. and Duarte-GarcÃ­a, A. and ReÃ¡tegui-Sokolova, C. and Clarke, A. E. and Wise, L. and Pons-Estel, G. J. and Santos, M. J. and Bernatsky, S. and Ribeiro, S. L. E. and Al Emadi, S. and Sparks, J. A. and Hsu, T. Y. and Patel, N. J. and Gilbert, E. L. and Valenzuela-Almada, M. O. and JÃ¶nsen, A. and L and olfi, G. and Fredi, M. and Goulenok, T. and Devaux, M. and Mariette, X. and Queyrel, V. and RomÃ£o, V. C. and Sequeira, G. and Hasseli, R. and Hoyer, B. and Voll, R. E. and Specker, C. and Baez, R. and Castro-Coello, V. and Maldonado Ficco, H. and Reis Neto, E. T. and Ferreira, G. A. A. and Monticielo, O. A. A. and Sirotich, E. and Liew, J. and Hausmann, J. and Sufka, P. and Grainger, R. and Bhana, S. and Costello, W. and Wallace, Z. S. and Jacobsohn, L. and Taylor, T. and Ja, C. and Strangfeld, A. and Mateus, E. F. and Hyrich, K. L. and Carmona, L. and Lawson-Tovey, S. and Kearsley-Fleet, L. and SchÃ¤fer, M. and Machado, P. M. and Robinson, P. C. and Gianfrancesco, M. and Yazdany, J. | Characteristics associated with poor COVID-19 outcomes in individuals with systemic lupus erythematosus: data from the COVID-19 Global Rheumatology Alliance | 2022 | Insufficient data |
| Ugarte-Gil, M. F. and AlarcÃ³n, G. S. and Seet, A. M. and Izadi, Z. and Montgomery, A. D. and Duarte-GarcÃ­a, A. and Gilbert, E. L. and Valenzuela-Almada, M. O. and Wise, L. and Sparks, J. A. and Hsu, T. Y. and D'Silva, K. M. and Patel, N. J. and Sirotich, E. and Liew, J. W. and Hausmann, J. S. and Sufka, P. and Grainger, R. and Bhana, S. and Wallace, Z. and Jacobsohn, L. and Strangfeld, A. and Mateus, E. F. and Hyrich, K. L. and Gossec, L. and Carmona, L. and Lawson-Tovey, S. and Kearsley-Fleet, L. and Schaefer, M. and Machado, P. M. and Robinson, P. C. and Gianfrancesco, M. and Yazdany, J. | Association Between Race/Ethnicity and COVID-19 Outcomes in Systemic Lupus Erythematosus Patients From the United States: Data From the COVID-19 Global Rheumatology Alliance | 2023 | 2023 |
| Varnai, C. and Palles, C. and Arnold, R. and Curley, H. M. and Purshouse, K. and Cheng, V. W. T. and Booth, S. and Campton, N. A. and Collins, G. P. and Hughes, D. J. and Kulasekararaj, A. G. and Lee, A. J. X. and Olsson-Brown, A. C. and Sharma-Oates, A. and Van Hemelrijck, M. and Lee, L. Y. W. and Kerr, R. and Middleton, G. and Cazier, J. B. | Mortality among Adults with Cancer Undergoing Chemotherapy or Immunotherapy and Infected with COVID-19 | 2022 | Insufficient data |
| Vart, P. and Jager, K. J. and Arnol, M. and Duivenvoorden, R. and Franssen, C. F. M. and Groeneveld, M. and Hemmelder, M. H. and Lepeytre, F. and Malfait, T. and Midtvedt, K. and Mitra, S. and Facundo, C. and Noordzij, M. and Reina, C. C. and Safak, S. and Toapanta, N. and Hilbr and s, L. B. and Gansevoort, R. T. | A Comparative Study of Patient Mortality during First and Second Waves of Covid-19 Pandemic in Dialysis Patients and Kidney Transplant Recipients | 2022 | Insufficient data |
| Venturas, J., Zamparini, J., Shaddock, E., Stacey, S., Murray, L., Richards, G.A., Kalla, I., Mahomed, A., Mohamed, F., Mer, M. and Maposa, I. | Comparison of outcomes in HIV-positive and HIV-negative patients with COVID-19 | 2021 | Single Centre |
| Vieira, M. and Comarmond, C. and Labreuche, J. and Mirouse, A. and Saadoun, D. and Richez, C. and Flipo, R. M. and Hachulla, E. and Drumez, E. and Cacoub, P. | COVID-19 outcomes in giant cell arteritis and polymyalgia rheumatica versus rheumatoid arthritis: A national, multicenter, cohort study | 2022 | Insufficient data |
| Vijenthira, Abi and Gong, Inna Y and Fox, Thomas A and Booth, Stephen and Cook, Gordon and Fattizzo, Bruno and MartÃ­n-Moro, Fern and o and Razanamahery, Jerome and Riches, John C and Zwicker, Jeff | Outcomes of patients with hematologic malignancies and COVID-19: a systematic review and meta-analysis of 3377 patients | 2020 | Systematic Review |
| Visco, C. and Marcheselli, L. and Mina, R. and Sassone, M. and Guidetti, A. and Penna, D. and Cattaneo, C. and Bonuomo, V. and Busca, A. and Ferreri, A. J. M. and Bruna, R. and Petrucci, L. and Cairoli, R. and Salvini, M. and BertÃ¹, L. and Ladetto, M. and Pilerci, S. and Pinto, A. and Ramadan, S. and Marchesi, F. and Cavo, M. and Arcaini, L. and Coviello, E. and Romano, A. and Musto, P. and Massaia, M. and Fracchiolla, N. and Marchetti, M. and Scattolin, A. and Tisi, M. C. and Cuneo, A. and Della Porta, M. and Trentin, L. and Turrini, M. and Gherlinzoni, F. and Tafuri, A. and Galimberti, S. and Bocchia, M. and Cardinali, V. and Cilloni, D. and Corso, A. and Armiento, D. and Rigacci, L. and La Barbera, E. O. and Gambacorti-Passerini, C. and Visani, G. and Vallisa, D. and Venditti, A. and Selleri, C. and Conconi, A. and Tosi, P. and Lanza, F. and C and oni, A. and Krampera, M. and Corradini, P. and Passamonti, F. and Merli, F. | A prognostic model for patients with lymphoma and COVID-19: aÂ multicentre cohort study | 2022 | Insufficient data |
| Wagner, Michael J and Hennessy, Cass and ra and Beeghly, Alicia and French, Benjamin and Shah, Dimpy P and Croessmann, Sarah and Vilar-Compte, Diana and Ruiz-Garcia, Erika and Ingham, Matthew and Schwartz, Gary K | Demographics, Outcomes, and Risk Factors for Patients with Sarcoma and COVID-19: A CCC19-Registry Based Retrospective Cohort Study | 2022 | Insufficient data |
| Waleed, M. and Arshad, M. A. and Abdallah, M. and Younossi, Z. M. and Singal, A. K. | Disease severity and time since transplantation determine patient mortality among liver transplant recipients with COVID-19 infection: A meta-analysis | 2020 | Systematic Review |
| Wang, B. and Huang, Y. | Immunotherapy or other anti-cancer treatments and risk of exacerbation and mortality in cancer patients with COVID-19: a systematic review and meta-analysis | 2020 | Systematic Review |
| Wang, Q., Berger, N.A. and Xu, R. | Â When hematologic malignancies meet COVID-19 in the United States: infections, death and disparities. | 2021 | Insufficient data |
| Wang, Y. and Feng, R. and Xu, J. and Shi, L. and Feng, H. and Yang, H. | An updated meta-analysis on the association between HIV infection and COVID-19 mortality | 2021 | Systematic Review |
| Wang, Y. and Xie, Y. and Hu, S. and Ai, W. and Tao, Y. and Tang, H. and Jing, F. and Tang, W. | Systematic Review and Meta-Analyses of The Interaction Between HIV Infection And COVID-19: Two Years' Evidence Summary | 2022 | Systematic Review |
| Weissman, S. and Aziz, M. and Smith, W. L. and Elias, S. and Swaminath, A. and Feuerstein, J. D. | Safety of biologics in inflammatory bowel disease patients with COVID-19 | 2021 | Systematic Review |
| Yang, H., Xu, J., Liang, X., Shi, L. and Wang, Y | Autoimmune diseases are independently associated with COVID-19 severity: Evidence based on adjusted effect estimates | 2021 | Systematic Review |
| Yendewa, G.A., Perez, J.A., Schlick, K., Tribout, H. and McComsey, G.A. | Clinical features and outcomes of COVID-19 among people living with HIV in the United States: A multicenter study from a large global health research networkÂ â€¦ | 2021 | Duplication |
| Yendewa, G.A., Perez, J.A., Schlick, K., Tribout, H. and McComsey, G.A. | Clinical Features and Outcomes of Coronavirus Disease 2019 Among People With Human Immunodeficiency Virus in the United States: A Multicenter Study From a Large Global Health Research Network (TriNetX). | 2021 | Duplication |
| Yilmaz, C. and Demir, I | Mortality Rate and Related Factors in Patients with Active Oncological Malignancies Hospitalized for Covid-19. | 2022 | Single Centre |
| Yue, X., Ye, Y., Choi, Y.C., Zhang, D. and Krueger, W.S. | Risk of severe COVID-19 outcomes among patients with immune-mediated inflammatory diseases or malignancies: a retrospective analysis of real-world dataÂ â€¦ | 2022 | Paediatric |
| Yunihastuti, E. and Karjadi, T. H. and Widhani, A. and Mahdi, H. I. S. and Sundari, S. and Hapsari, A. F. and Koesnoe, S. and Djauzi, S. | Incidence and severity prediction score of COVID-19 in people living with HIV (SCOVHIV): experience from the first and second waves of the pandemic in Indonesia | 2022 | Insufficient data |
| Zabana, Y. and MarÃ­n-JimÃ©nez, I. and RodrÃ­guez-Lago, I. and Vera, I. and MartÃ­n-Arranz, M. D. and Guerra, I. and Gisbert, J. P. and Mesonero, F. and BenÃ­tez, O. and Taxonera, C. and Ponferrada-DÃ­az, Ã and Piqueras, M. and Lucendo, A. J. and Caballol, B. and MaÃ±osa, M. and MartÃ­nez-Montiel, P. and Bosca-Watts, M. and Gordillo, J. and Buj and a, L. and ManceÃ±ido, N. and MartÃ­nez-PÃ©rez, T. and LÃ³pez, A. and RodrÃ­guez-GutiÃ©rrez, C. and GarcÃ­a-LÃ³pez, S. and Vega, P. and Rivero, M. and Melcarne, L. and Calvo, M. and Iborra, M. and Barreiro de-Acosta, M. and Sicilia, B. and Barrio, J. and PÃ©rez, J. L. and Busquets, D. and PÃ©rez-MartÃ­nez, I. and Navarro-Llavat, M. and HernÃ¡ndez, V. and ArgÃ¼elles-Arias, F. and RamÃ­rez Esteso, F. and Meijide, S. and Ramos, L. and GomollÃ³n, F. and MuÃ±oz, F. and Suris, G. and de Zarate, J. O. and Huguet, J. M. and LlaÃ³, J. and GarcÃ­a-Sepulcre, M. F. and Sierra, M. and DurÃ , M. and Estrecha, S. and Fuentes Coronel, A. and Hinojosa, E. and Olivan, L. and Iglesias, E. and GutiÃ©rrez, A. and Varela, P. and Rull, N. and Gilabert, P. and HernÃ¡ndez-Camba, A. and Brotons, A. and Ginard, D. and SesÃ©, E. and Carpio, D. and Aceituno, M. and Cabriada, J. L. and GonzÃ¡lez-Lama, Y. and JimÃ©nez, L. and Chaparro, M. and LÃ³pez-San RomÃ¡n, A. and Alba, C. and Plaza-Santos, R. and Mena, R. and Tamarit-SebastiÃ¡n, S. and Ricart, E. and Calafat, M. and Olivares, S. and Navarro, P. and Bertoletti, F. and Alonso-GalÃ¡n, H. and Pajares, R. and Olcina, P. and Manzano, P. and DomÃ¨nech, E. and Esteve, M. and On Behalf Of The Eneida Registry Of, Geteccu | Nationwide COVID-19-EII Study: Incidence, Environmental Risk Factors and Long-Term Follow-Up of Patients with Inflammatory Bowel Disease and COVID-19 of the ENEIDA Registry | 2022 | Insufficient data |
| Zanetti, A., Carrara, G., Landolfi, G., Rozza, D., Chighizola, C.B., Alunno, A., Andreoli, L., Caporali, R., Gerli, R., Sebastiani, G.D. and Valesini, G. | Increased COVID-19 mortality in patients with rheumatic diseases: results from the CONTROL-19 study by the Italian Society for Rheumatology | 2022 | Duplication |
| Zhang, M. and Bai, X. and Cao, W. and Ji, J. and Wang, L. and Yang, Y. and Yang, H. | | 2021 | Systematic Review |
